# Supplementary material for: Overlapping community detection in networks based on link partitioning and partitioning around medoids
Source: PLoS One. 2021 Aug 25;16(8):e0255717. doi: 10.1371/journal.pone.0255717 (PMC8386890; doi:10.1371/journal.pone.0255717)
Supplement: S1 Appendix — The computational results and the clustering of the exact LPAM method. (PDF) [file pone.0255717.s001.pdf]

LPAM-Exact-Amplified-Commute-Distance

April 27, 2020

Link Partitioning by Partioning Around Medoids – Exact
Distance function: Amplified Commute Distance

In [30]:
```
import numpy as np
import random
random.seed = 108
from tqdm import tqdm_notebook as tqdm
import seaborn as sns
import matplotlib.pyplot as plt
from mpl_toolkits import mplot3d
import pandas as pd
from PIL import Image
from io import BytesIO
%matplotlib inline
```

In [31]:
```
!ls ../lp_solve_5-2.5_java/lib/ux64
```

```
liblpsolve55j.so
```

In [60]:
```
!java -jar ../lpam/target/Clustering-1.5-jar-with-dependencies.jar
```

```
Missing required options: i, k
usage: PMPClustering <-i input_file> <-k number_of_clusters> [-ftbcd]
 -a,--algorithm <arg>       Specifies algorithm that will be used to find
                            disjoint edge clusters. Possible values:
                            pmp (p-median exact algorithm).
                            kmd (k-medoids heuristic)
                            fkmd (fast k-medioids heuristic)
                            kmn (k-means heuristic).
                            If this option is omitted, the P-Median
                            algorithm will be used.
 -b,--benchmark             use benchmark format (.dat file)
 -d,--distance <arg>        the type of function to measure the distance
                            between nodes.
                            Possible values:
                            sp (shortest path)
                            gd (Generalize Degree)
                            cm (Commute Distance)
                            acm (Amplified Commute Distance).
                            If this option is omitted, the amplified
                            commute distance function will be used.
 -f,--force                 The previous founded solution by lp_solver
                            will not be used. lp_solver will be started
                            forcibly.
                            The flag effects only the PMP exact edge
                            clustering algorithm.
```

```
                              By default algorithm will try to get previous
                              founded solution
 -gt,--groundTruth <arg>      load info about ground truth community file
 -i,--input <arg>             path to the input file in GML format
 -k <arg>                     Number of clusters to detect (int)
 -l,--linegraph               produce line graph as output
 -mn,--maxNeighbors <arg>     How many maximum number of neighbors will be
                              used by CLARANS heuristic
                              Default value is 100
 -o,--outputDir <arg>         the name of the output file. If option was
                              omitted, the name of the output file
                              will be composed automatically as
                              "{suffix}_{distanceName}_{clusterNumber}_out.g
                              exf"
 -s,--solver <arg>            solver type: cplex or lpsolve
 -t,--threshold <arg>         Vertex i belongs to cluster C, if node i has
                              fraction of edges in cluster C greater then
                              this value
                              Default value is 0.
```

In [123]:
```
import re
err = None
def lp_experiment(clustersNumber, algorithm, distance, inputFile, groundTruth, params =
{}, vertexNumerationShift=0, benchmarkFormat=False, verbose=False, lineGraph=False,
force=True):
    lineGraphParam = ""
#     path_to_jar = "-Djava.library.path=../lib ../lpam/target/Clustering-1.5-jar-with-
dependencies.jar"
    path_to_jar = "-Djava.library.path=/home/latna/cplex/cplex/bin/x86-64_linux
../lpam/target/Clustering-1.5-jar-with-dependencies.jar"
    #      path_to_jar = "-Djava.library.path=../lp_solve_5-2.5_java/lib/ux64:../lib
../lpam/target/Clustering-1.0-SNAPSHOT-jar-with-dependencies.jar"
    if (lineGraph == True):
        lineGraphParam = " -l"
    datasetName = inputFile.split('/')[-2]
    sufix = inputFile.split('/')[-1].split('.')[0]
    outputDir = "../Results/lp_{}_{}_{}".format(algorithm, distance, datasetName)
    outputFile = outputDir +'/' +"pmp_{3}_{2}_{0}_{1}.dat".format(algorithm,
clustersNumber, distance.upper(), sufix)
    print("Output dir name: {}".format(outputDir) )
    print("Output file name: {}".format(outputFile) )
    if force:
        print("Force flag detected. Remove *.ser and *.lp files from output dir.")
        !rm {outputDir}/*.ser {outputDir}/*.lp
    _all_results = {}
    bestParam = "not found"
    nmi_best = 0;
    param_list = list(generate_params(params))
    tmp = None
    for param in tqdm(param_list):
        if benchmarkFormat:
            tmp=!java -Xmx20G -jar {path_to_jar} -b -a {algorithm} -o {outputDir} -i
{inputFile} -k {clustersNumber} -d {distance} -gt {groundTruth} {param} -s cplex
            tmpFile = outputFile
        else:
            tmp=!java -Xmx20G -jar {path_to_jar} -a {algorithm} -o {outputDir} -i
{inputFile} -k {clustersNumber} -d {distance} -gt {groundTruth} {param} -s cplex
            if verbose:
                err = tmp
                print(tmp)
            lines=[]
            with open(outputFile) as f:
                lines = f.readlines()
            tmpFile="tmpFile.dat"
```

```
                with open(tmpFile, 'w') as the_file:
                    for line in lines:
                        the_file.write(" ".join([str(int(a)+vertexNumerationShift) for a in
line.split()]) + "\n")
            clustering_time = int(re.search(r"Clustering work time: (\w+)"
,'\n'.join(tmp)).groups()[0])
            matrix_time = int(re.search(r"Matrix work time: (\w+)"
,'\n'.join(tmp)).groups()[0])

            output=!../Overlapping-NMI/onmi {groundTruth} {tmpFile}
            nmi=float(output[0].split()[1])
            _all_results[param] = (nmi, matrix_time, clustering_time)
            if nmi >  nmi_best:
                bestParam = param
                nmi_best = nmi
        #restorign solution for the best parameters
        if benchmarkFormat:
            tmp=!java -Xmx24G -jar {path_to_jar} -b -a {algorithm} -o {outputDir} -i
{inputFile} -gt {groundTruth} -k {clustersNumber} -d {distance} -gt {groundTruth}
{bestParam} {lineGraphParam} -s cplex
            tmpFile = outputFile
        else:
            tmp=!java -Xmx24G -jar {path_to_jar} -a {algorithm} -o {outputDir} -i
{inputFile} -gt {groundTruth} -k {clustersNumber} -d {distance} -gt {groundTruth}
{bestParam} {lineGraphParam} -s cplex
            lines=[]
            with open(outputFile) as f:
                lines = f.readlines()
            tmpFile="tmpFile.dat"
            with open(tmpFile, 'w') as the_file:
                for line in lines:
                    the_file.write(" ".join([str(int(a)+vertexNumerationShift) for a in
line.split()]) + "\n")

        if verbose:
            print(tmp)
        print("Matrix time: {} Clustering time: {} Best ONMI: {} params: '{}'".format(
_all_results[param_list[0]][1],_all_results[param_list[0]][2], nmi_best, bestParam) )
        return _all_results
```

In [87]:
```
def generate_params(params):
    keys = list(params.keys())
    if len(keys) == 1:
        for value in params[keys[0]]:
            yield ( keys[0] + " " + str(value) )
    if len( keys ) > 1:
        for value in params[keys[0]]:
            for remain_params in generate_params({k:params[k] for k in keys[1:]}):
                yield ( keys[0] + " " + str(value) + " " + remain_params )
```

In [88]:
```
def plot_all_params(algorithm, dataset, all_results, fName=None ):
    xdata=[]
    ydata=[]
    df = pd.DataFrame()
    for param, res in all_results.items():
        nmi = res[0]
        splited = param.split()
        xdata.append(float(splited[1]))
        ydata.append(nmi)
        df = df.append({'x': float(splited[1]), 'y': nmi}, ignore_index=True)

    plt.plot(xdata, ydata, 'C3', zorder=1, lw=3)

    plt.scatter(xdata, ydata,s=70,zorder=2)
    plt.xlabel('threshold')
    plt.ylabel('onmi value');
    plt.title('Algorithm: {}\nDataset: {}'.format(algorithm, dataset));
#     if (fName is not None):
```

```
#         png1 = BytesIO()
#         plt.savefig(png1, format='png', dpi=400)
#         png2 = Image.open(png1)
#         ffName = '../figures/{}.tiff'.format(fName)
#         png2.save(ffName)
#         png1.close()
#         print("figure saved to: " + ffName)
        plt.show()
```

```
In [124]: params={}
          all_results = {}
          params["-t"] = np.arange(0.05, 1.0, 0.05)
```

```
In [134]: !echo hello!
```

```
hello!
```

1 School friendship network

```
In [147]: all_results['school'] = lp_experiment(clustersNumber=6,
                      algorithm = "pmp",
                      distance = "acm",
                      inputFile =  "../datasets/school_friendship/school-2.gml",
                      groundTruth = "../datasets/school_friendship/truth-school.dat",
                      params = params,
                      vertexNumerationShift=-1,
                      benchmarkFormat=False, verbose=False, lineGraph=True, force=False)
```

```
Output dir name: ../Results/lp_pmp_acm_school_friendship
Output file name: ../Results/lp_pmp_acm_school_friendship/pmp_school-2_ACM_pmp_6.dat
```

```
HBox(children=(IntProgress(value=0, max=19), HTML(value='')))
```

```
Matrix time: 3358 Clustering time: 29918 Best ONMI: 0.697792 params: '-t
0.35000000000000003'
```

```
In [146]: all_results['school'] = lp_experiment(clustersNumber=7,
                      algorithm = "pmp",
                      distance = "acm",
                      inputFile =  "../datasets/school_friendship/school-2.gml",
                      groundTruth = "../datasets/school_friendship/truth-school.dat",
                      params = params,
                      vertexNumerationShift=-1,
                      benchmarkFormat=False, verbose=False, lineGraph=True, force=False)
```

```
Output dir name: ../Results/lp_pmp_acm_school_friendship
Output file name: ../Results/lp_pmp_acm_school_friendship/pmp_school-2_ACM_pmp_7.dat
```

```
HBox(children=(IntProgress(value=0, max=19), HTML(value='')))
```

```
Matrix time: 25 Clustering time: 23 Best ONMI: 0.748707 params: '-t 0.55'
```

```
In [142]: !ls -lahtr ../Results/lp_pmp_acm_school_friendship
```

```
total 42M
-rw-r--r--   1 latna docker 3.3M Mar  7 02:16 solution_school-2.gml.lp_2
drwxr-xr-x 145 latna docker  12K Mar 23 14:39 ..
-rw-r--r--   1 latna docker 297K Mar 23 18:44 d_matrix_school-2_ACM_pmp_7.ser
-rw-r--r--   1 latna docker  12M Mar 23 18:44 model_school-2.gml.lp
-rw-r--r--   1 latna docker  23M Mar 23 18:44 cplex_model_school-2.gml.lp
-rw-r--r--   1 latna docker 3.3M Mar 23 18:45 solution_school-2.gml.lp
drwxr-xr-x   2 latna docker 4.0K Mar 23 18:45 .
-rw-r--r--   1 latna docker 2.6K Mar 23 18:45
cluster_edges_hmap_school-2_ACM_pmp_7.ser
-rw-r--r--   1 latna latna  1.1K Mar 23 18:45 network_formatschool-2_ACM_pmp_7.dat
-rw-r--r--   1 latna docker  183 Mar 23 18:45 pmp_school-2_ACM_pmp_7.dat
-rw-r--r--   1 latna latna  136K Mar 23 18:45 school-2_ACM_pmp_7_out_line.gexf
-rw-r--r--   1 latna latna   67K Mar 23 18:45 school-2_ACM_pmp_7_out.gexf
```

```
In [ ]: plot_all_params(algorithm = "LPAM-Exact + acm",
                        dataset = "School Friendship", all_results = all_results['school'])
```

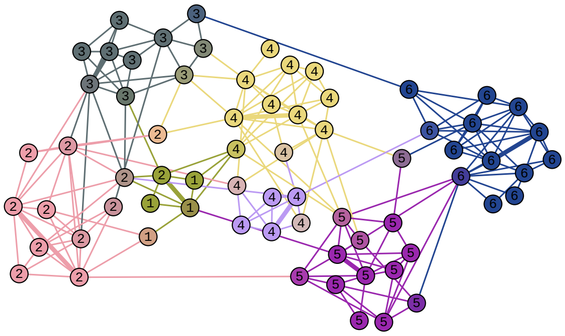

Clustering results for School Frienship Netwrok for the best parameters

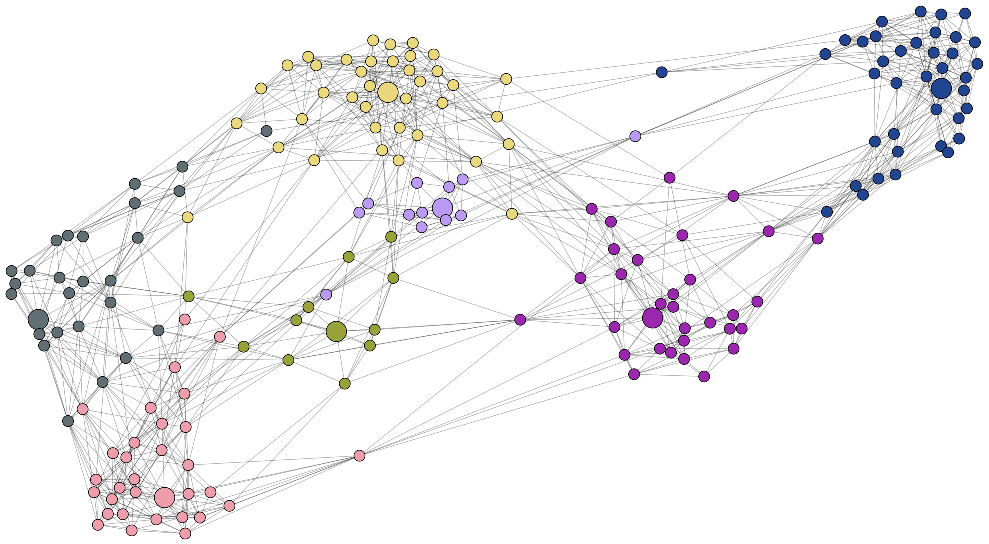

Clustering results for School Frienship Netwrok for the best parameters – Line graph

1.1 Karate Club

In [126]:
```
params={}
params["-t"] = np.arange(0.05, 1.0, 0.05)
all_results['karate'] = lp_experiment(clustersNumber=2,
              algorithm = "pmp",
              distance = "acm",
              inputFile =  "../datasets/karate/karate.gml",
              groundTruth = "../datasets/karate/truth_karate.dat",
              params = params,
              vertexNumerationShift=0,
              benchmarkFormat=False,
              lineGraph=True, force=True)
```

```
Output dir name: ../Results/lp_pmp_acm_karate
Output file name: ../Results/lp_pmp_acm_karate/pmp_karate_ACM_pmp_2.dat
Force flag detected. Remove *.ser and *.lp files from output dir.
```

```
HBox(children=(IntProgress(value=0, max=19), HTML(value='')))
```

```
Matrix time: 181 Clustering time: 1495 Best ONMI: 0.91796 params: '-t 0.45'
```

In [55]:
```
plot_all_params(algorithm = "LPAM-Exact+acm", dataset = "Karate Club ", all_results =
all_results['karate'])
```

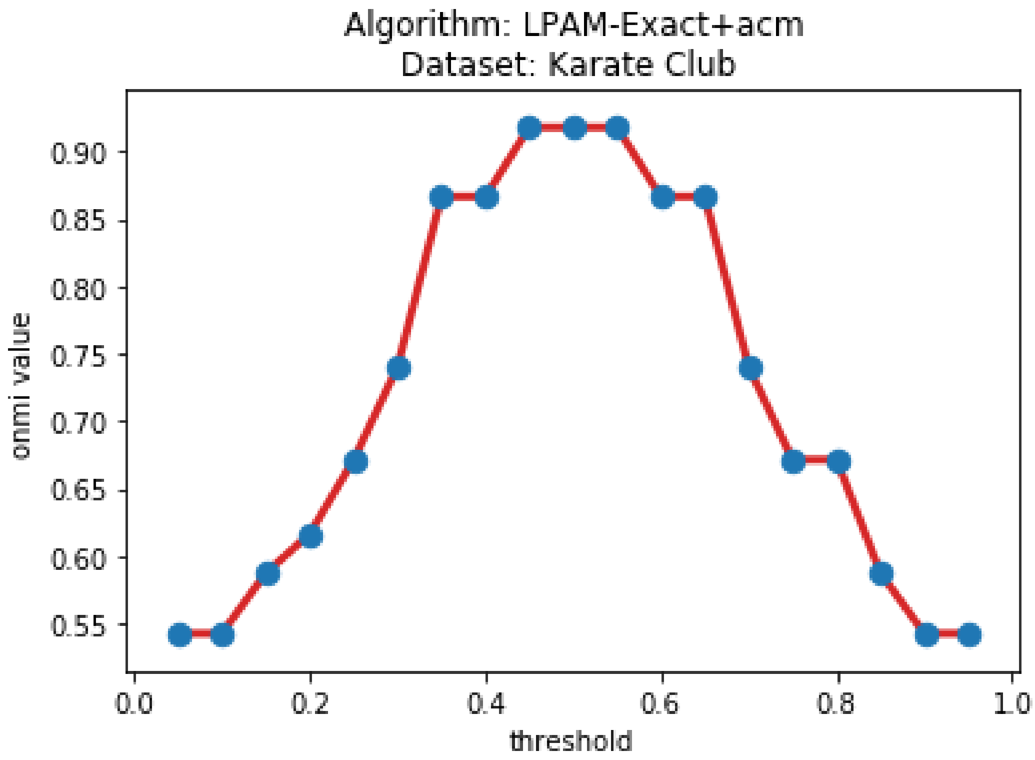

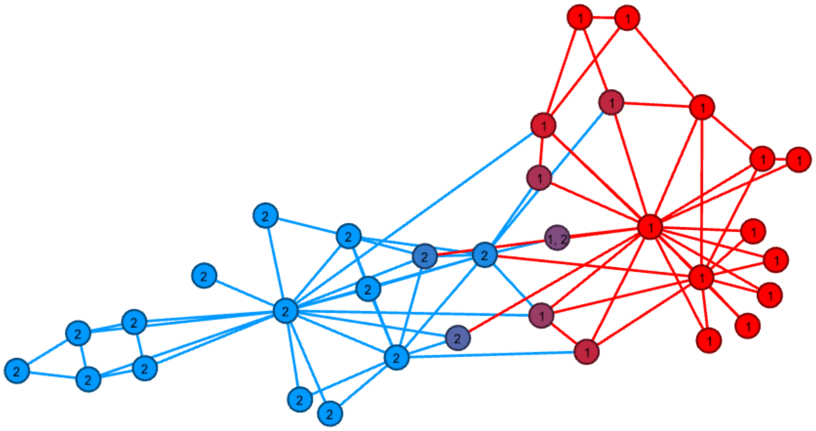

Karate Club

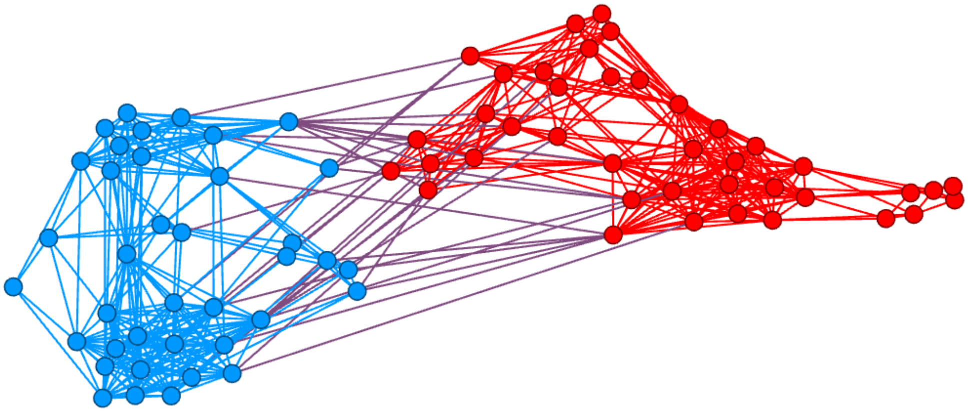

Karate Club - Line graph

2 American Football League c = 12

```
In [152]: params={}
          params["-t"] = np.arange(0.05, 1.0, 0.05)
          all_results['Fooball League'] = lp_experiment(clustersNumber=12,
                        algorithm = "pmp",
                        distance = "acm",
                        inputFile =  "../datasets/football/footballTSEinput.gml",
                        groundTruth = "../datasets/football/truth_footballTSEinput.dat",
                        params = params,
                        vertexNumerationShift=-1,
                        verbose = True,
                        benchmarkFormat=False)
```

```
In [146]: plot_all_params(algorithm = "LPAM-Heuristic-Clarance + acm", dataset = "American
          Foolball League", all_results = all_results)
```

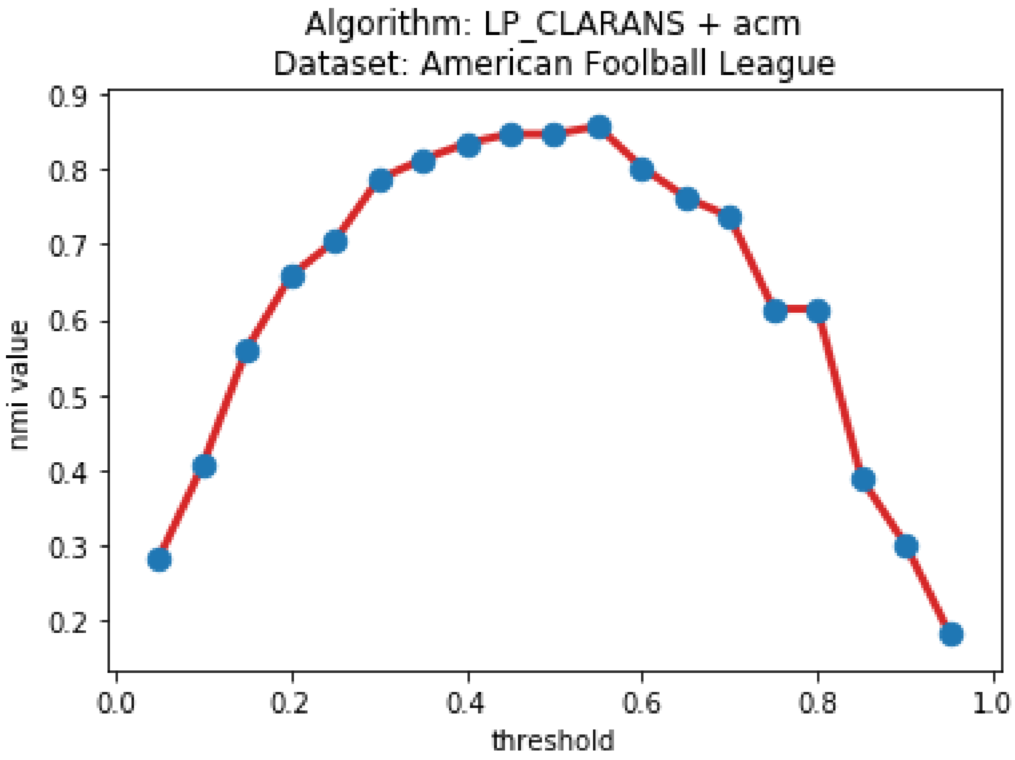

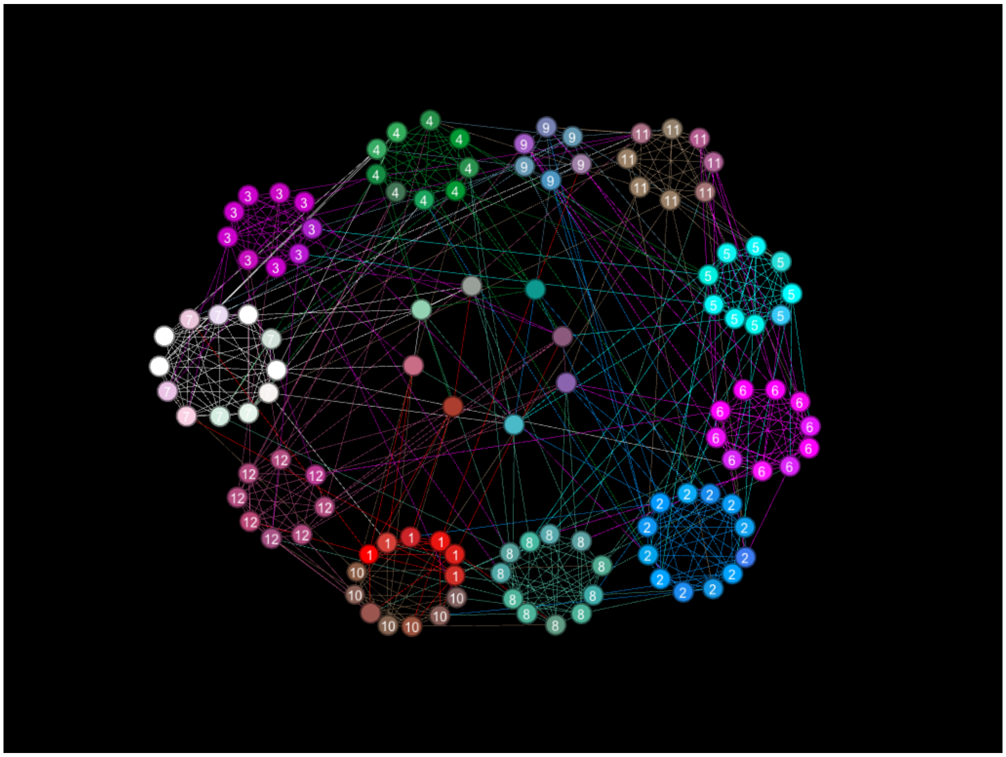

Americal Football League. onmi: 0.856281; thresould: 0.55

3 Adj-noun

In [127]:
```
params={}
params["-t"] = np.arange(0.05, 1.0, 0.05)
all_results['adj-noun'] = lp_experiment(clustersNumber=2,
              algorithm = "pmp",
              distance = "acm",
              inputFile =  "../datasets/adjnoun/adjnoun.dat",
              groundTruth = "../datasets/adjnoun/truth_adjnoun.dat",
              params = params,
              vertexNumerationShift=0,
              benchmarkFormat=True)
```

```
Output dir name: ../Results/lp_pmp_acm_adjnoun
Output file name: ../Results/lp_pmp_acm_adjnoun/pmp_adjnoun_ACM_pmp_2.dat
Force flag detected. Remove *.ser and *.lp files from output dir.
```

```
HBox(children=(IntProgress(value=0, max=19), HTML(value='')))
```

```
Matrix time: 110554 Clustering time: 1786141 Best ONMI: 0.00490969 params: '-t 0.1'
```

In [17]:
```
plot_all_params(algorithm = "LP + PAM-Exact + acm", dataset = "Adj noun", all_results =
all_results['adj-noun'] )
```

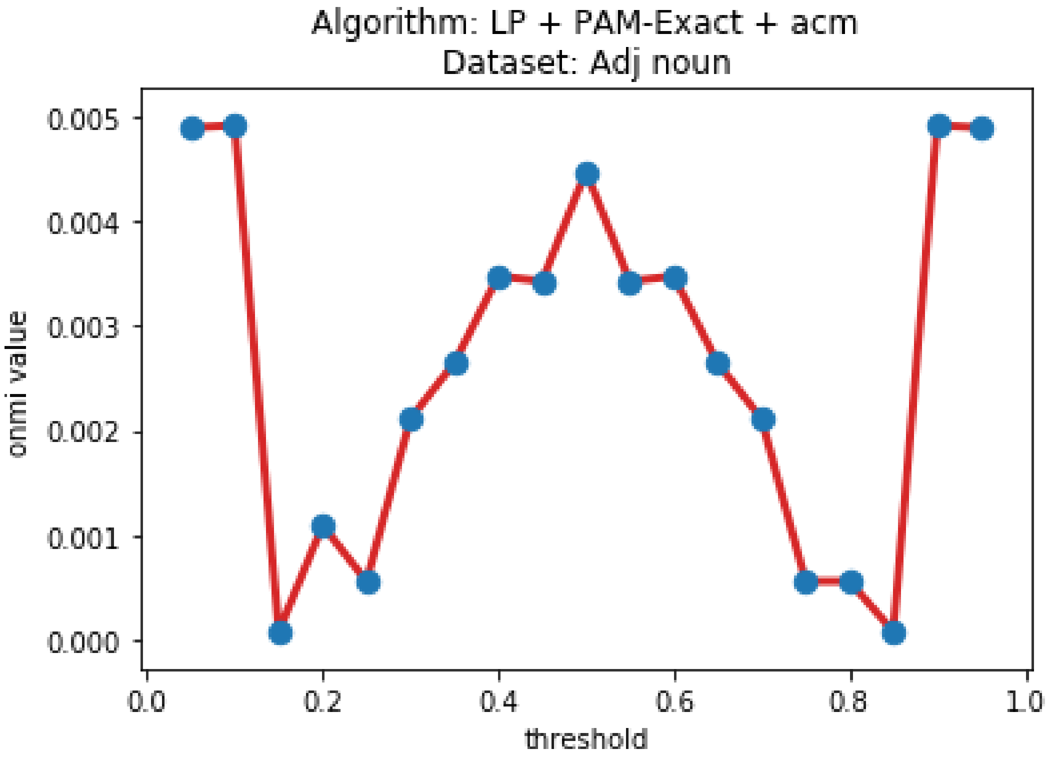

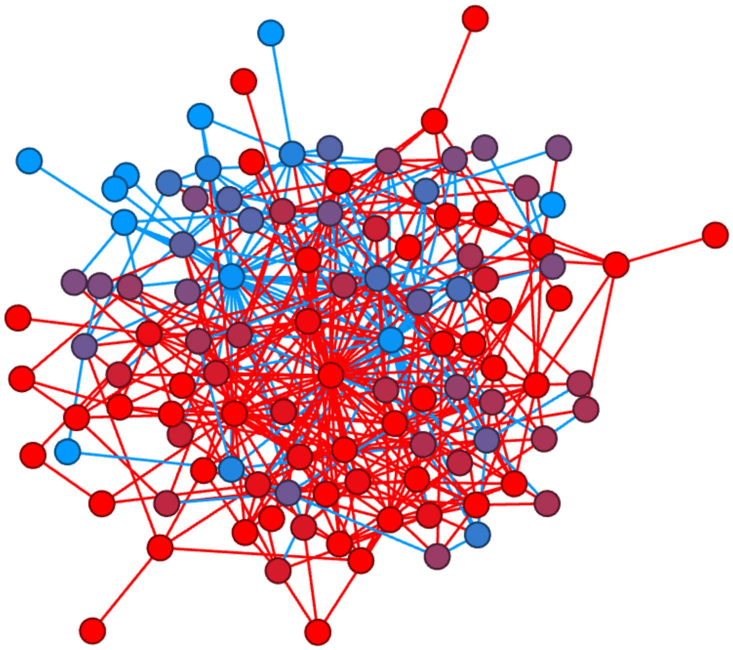

adj-noun onmi: 0.00490969; thresould: 0.1

4 Political Books

```
In [128]: params={}
          params["-t"] = np.arange(0.05, 1.0, 0.05)
          all_results['Politics Books'] = lp_experiment(clustersNumber=2,
                         algorithm = "pmp",
                         distance = "acm",
                         inputFile = "../datasets/polbooks/polbooks.dat",
                         groundTruth = "../datasets/polbooks/truth_polbooks.dat",
                         params = params,
                         vertexNumerationShift=0,
                         benchmarkFormat=True, verbose=False, lineGraph=True)
```

```
Output dir name: ../Results/lp_pmp_acm_polbooks
Output file name: ../Results/lp_pmp_acm_polbooks/pmp_polbooks_ACM_pmp_2.dat
Force flag detected. Remove *.ser and *.lp files from output dir.
```

```
HBox(children=(IntProgress(value=0, max=19), HTML(value='')))
```

```
Matrix time: 130428 Clustering time: 642852 Best ONMI: 0.464154 params: '-t 0.55'
```

```
In [19]: plot_all_params(algorithm = "LPAM-exact + acm", dataset = "Politics Books", all_results
         = all_results['Politics Books'])
```

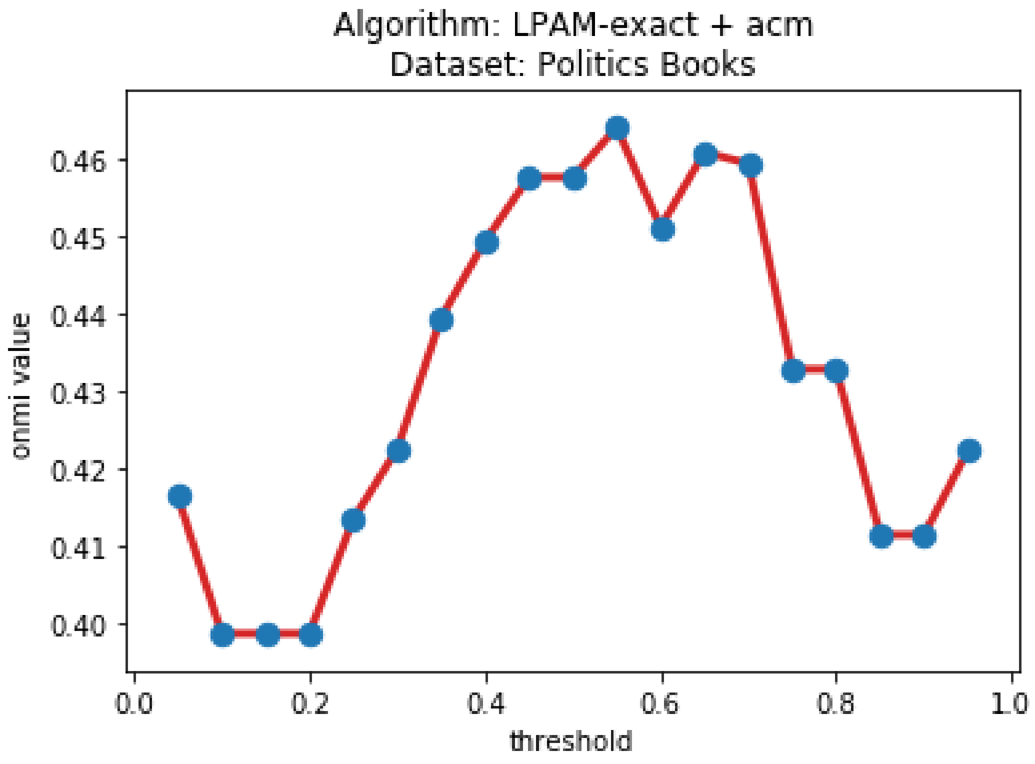

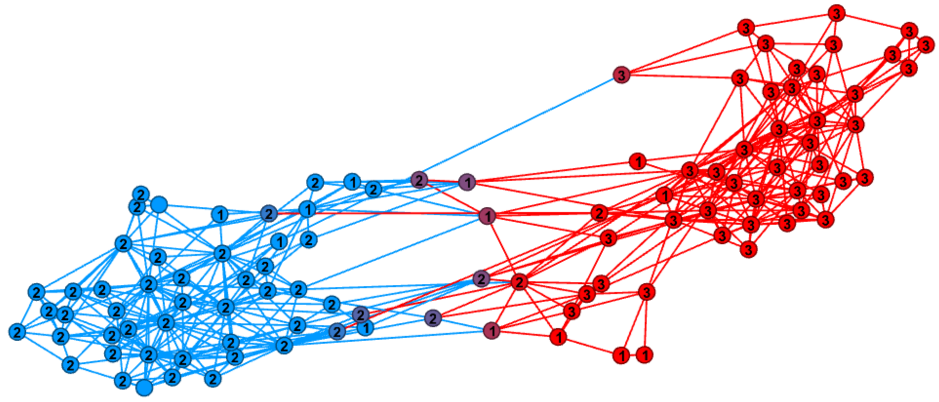

Political books

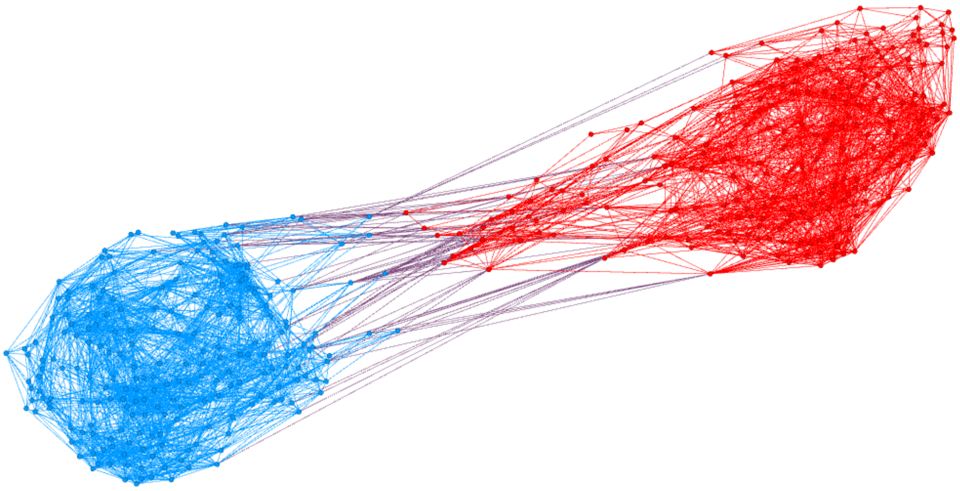

Political books - Line graph

5 Syntetic Datasets

5.0.1 bench_30

```
In [129]: params={}
          params["-t"] = np.arange(0.05, 1.0, 0.05)
          all_results['bench_30'] = lp_experiment(clustersNumber=3,
                        algorithm = "pmp",
                        distance = "acm",
                        inputFile = "../datasets/bench_30/bench_30_network.dat",
                        groundTruth = "../datasets/bench_30/bench_30_truth.dat",
                        params = params,
                        vertexNumerationShift=0,
                        benchmarkFormat=True, lineGraph=True, force=True)
```

```
Output dir name: ../Results/lp_pmp_acm_bench_30
Output file name: ../Results/lp_pmp_acm_bench_30/pmp_bench_30_network_ACM_pmp_3.dat
Force flag detected. Remove *.ser and *.lp files from output dir.
```

```
HBox(children=(IntProgress(value=0, max=19), HTML(value='')))
```

```
Matrix time: 169 Clustering time: 913 Best ONMI: 0.931866 params: '-t
0.35000000000000003'
```

```
In [45]: plot_all_params(algorithm = "LPAM-exact + Amplified Commute Distance", dataset =
         "bench_30", all_results = all_results['bench_30'])
```

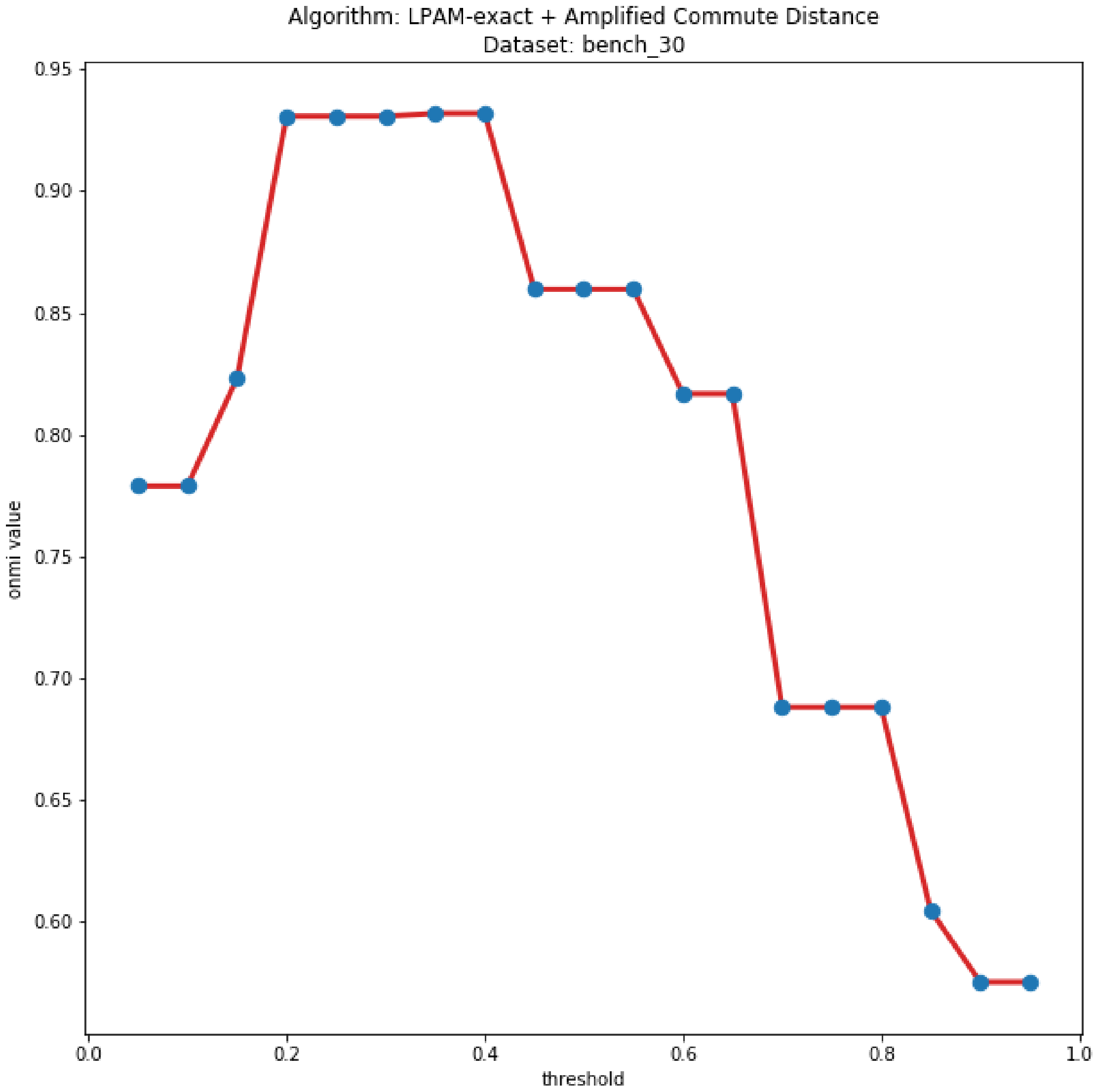
Algorithm: LPAM-exact + Amplified Commute Distance
Dataset: bench_30
onmi value
threshold
0.95
0.90
0.85
0.80
0.75
0.70
0.65
0.60
0.0
0.2
0.4
0.6
0.8
1.0

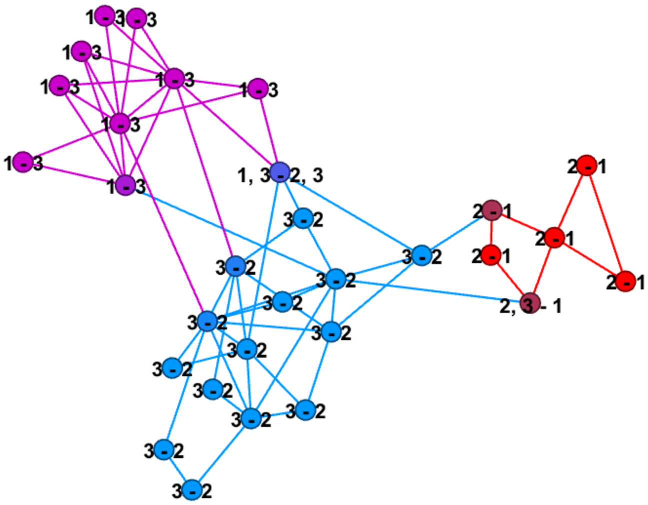

bench_30

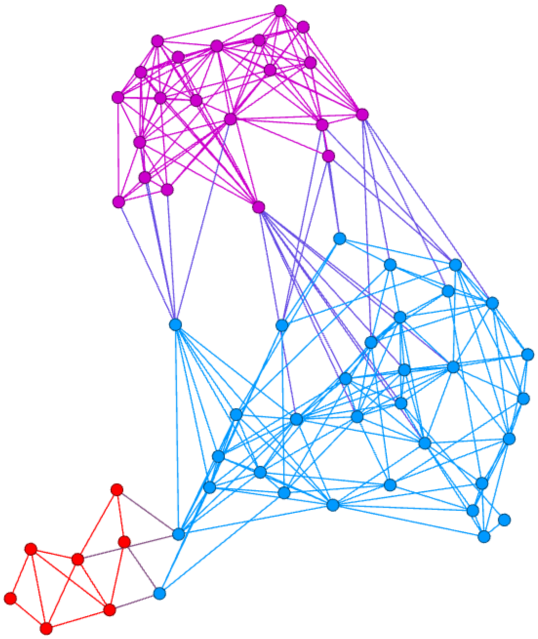

bench_30 - Line graph

bench_40

```
In [130]: params={}
          params["-t"] = np.arange(0.05, 1.0, 0.05)
          all_results['bench_40'] = lp_experiment(clustersNumber=4,
                        algorithm = "pmp",
                        distance = "acm",
                        inputFile = "../datasets/bench_40/bench_40_network.dat",
                        groundTruth = "../datasets/bench_40/bench_40_truth.dat",
                        params = params,
                        vertexNumerationShift=0,
                        benchmarkFormat=True, lineGraph=True)
```

```
Output dir name: ../Results/lp_pmp_acm_bench_40
Output file name: ../Results/lp_pmp_acm_bench_40/pmp_bench_40_network_ACM_pmp_4.dat
Force flag detected. Remove *.ser and *.lp files from output dir.
```

```
HBox(children=(IntProgress(value=0, max=19), HTML(value='')))
```

```
Matrix time: 395 Clustering time: 6074 Best ONMI: 0.346589 params: '-t
0.35000000000000003'
```

```
In [81]: plot_all_params(algorithm = "LPAM-exact + Amplified Commute Distance", dataset =
         "bench_40", all_results = all_results)
```

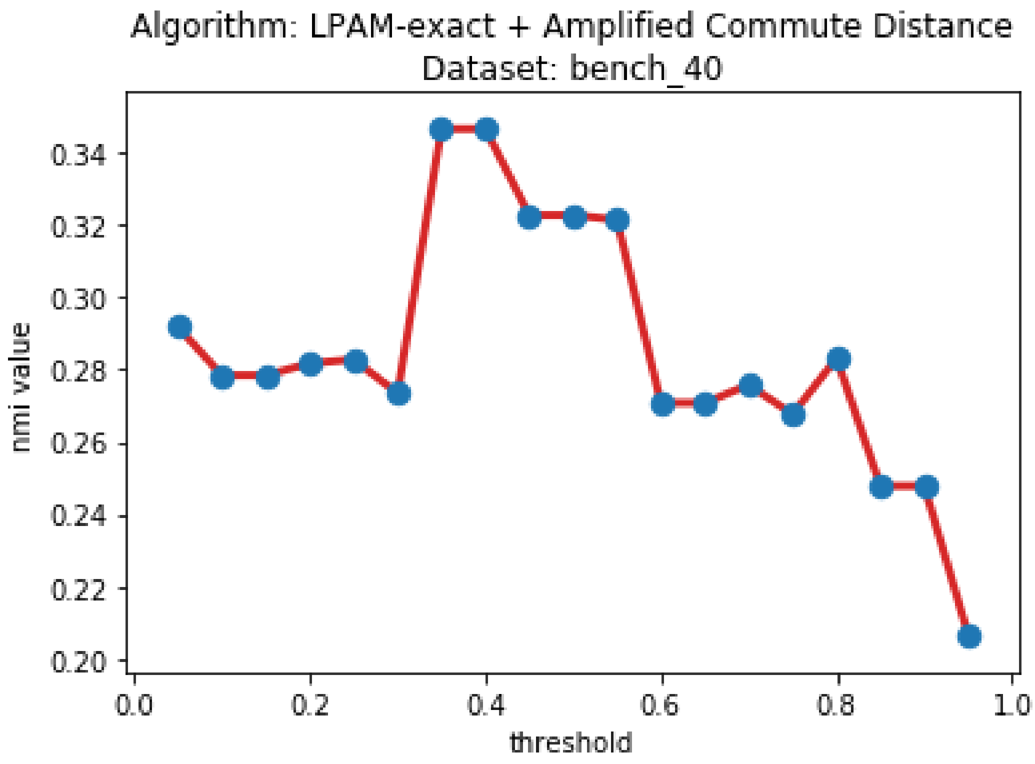

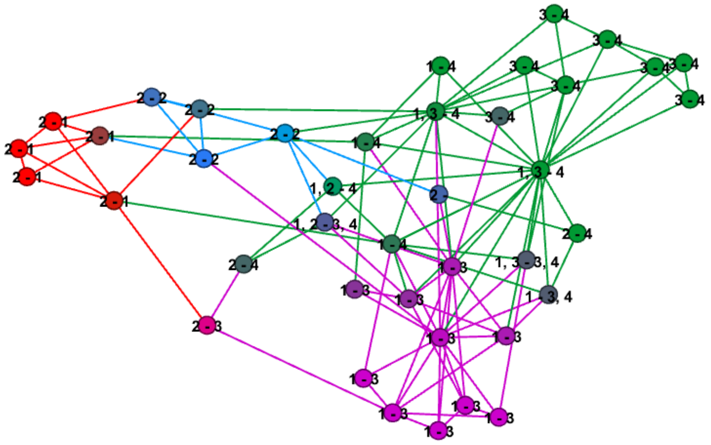

bench_50

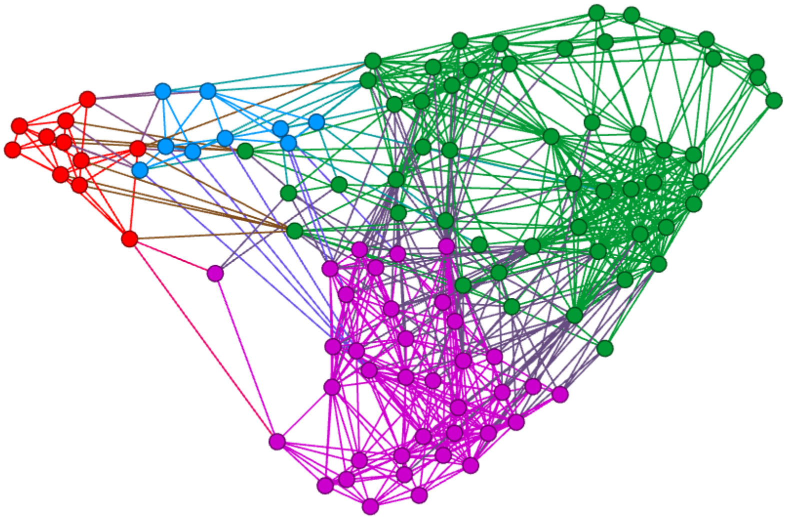

bench_50 - Line graph

6 bench_50

```
In [131]: params={}
          params["-t"] = np.arange(0.05, 1.0, 0.05)
          all_results['bench_50'] = lp_experiment(clustersNumber=4,
                        algorithm = "pmp",
                        distance = "acm",
                        inputFile = "../datasets/bench_50/bench_50_network.dat",
                        groundTruth = "../datasets/bench_50/bench_50_truth.dat",
                        params = params,
                        vertexNumerationShift=0,
                        benchmarkFormat=True, lineGraph=True)
```

```
Output dir name: ../Results/lp_pmp_acm_bench_50
Output file name: ../Results/lp_pmp_acm_bench_50/pmp_bench_50_network_ACM_pmp_4.dat
Force flag detected. Remove *.ser and *.lp files from output dir.
```

```
HBox(children=(IntProgress(value=0, max=19), HTML(value='')))
```

```
Matrix time: 881 Clustering time: 11662 Best ONMI: 0.845306 params: '-t
0.35000000000000003'
```

```
In [31]: plot_all_params(algorithm = "LPAM-exact + Amplified Commute Distance", dataset =
         "bench_50", all_results = all_results)
```

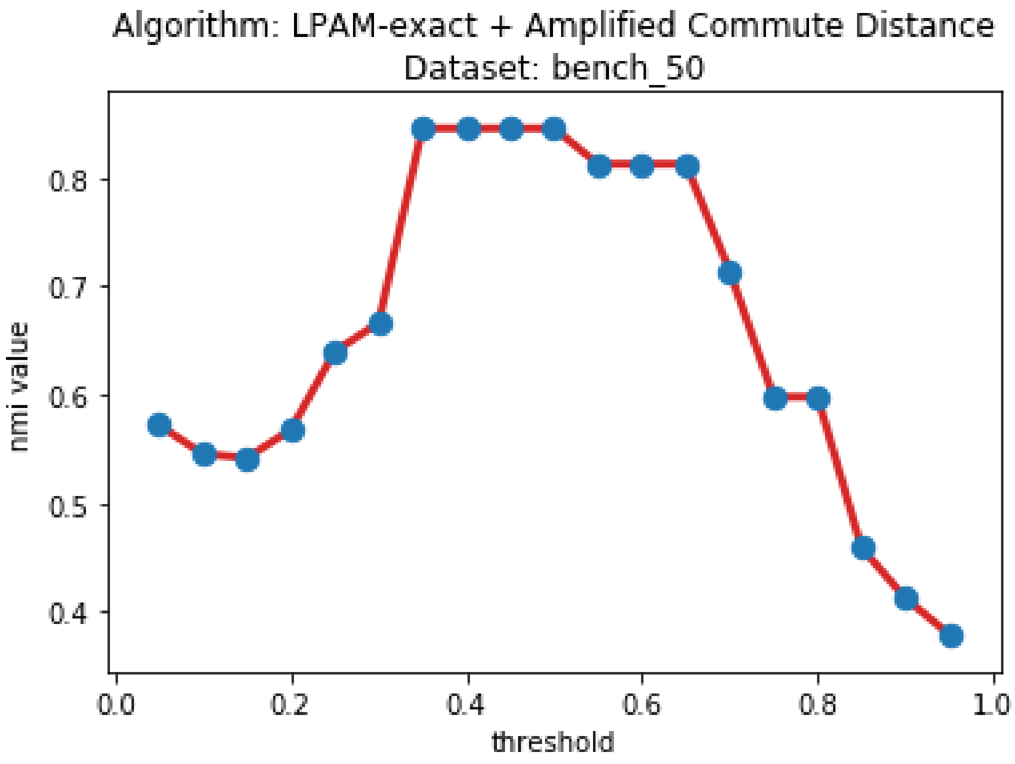

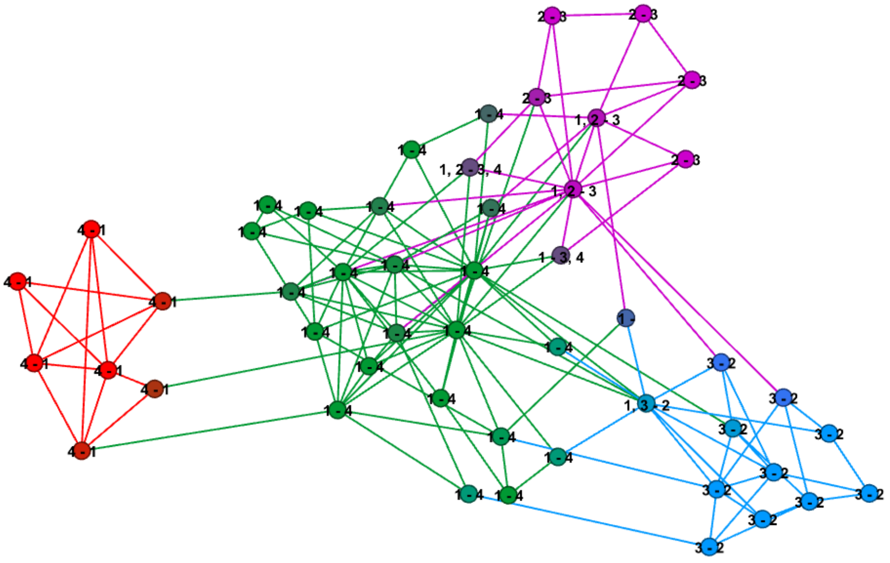

bench_50

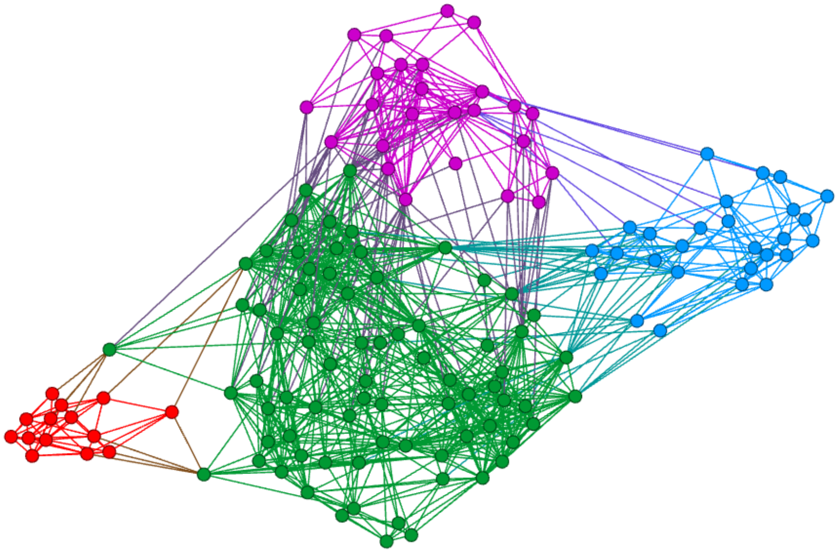

bench_50 - line graph

7 bench_60

In [132]:
```
params={}
params["-t"] = np.arange(0.05, 1.0, 0.05)
all_results['bench_60']  = lp_experiment(clustersNumber=6,
              algorithm = "pmp",
              distance = "acm",
              inputFile = "../datasets/bench_60/bench_60_network.dat",
              groundTruth = "../datasets/bench_60/bench_60_truth.dat",
              params = params,
              vertexNumerationShift=0,
              benchmarkFormat=True, lineGraph=True)
```

```
Output dir name: ../Results/lp_pmp_acm_bench_60
Output file name: ../Results/lp_pmp_acm_bench_60/pmp_bench_60_network_ACM_pmp_6.dat
Force flag detected. Remove *.ser and *.lp files from output dir.
```

```
HBox(children=(IntProgress(value=0, max=19), HTML(value='')))
```

```
Matrix time: 626 Clustering time: 4894 Best ONMI: 0.60211 params: '-t 0.55'
```

In [29]:
```
plot_all_params(algorithm = "LPAM-exact + Amplified Commute Distance", dataset =
"bench_60", all_results = all_results)
```

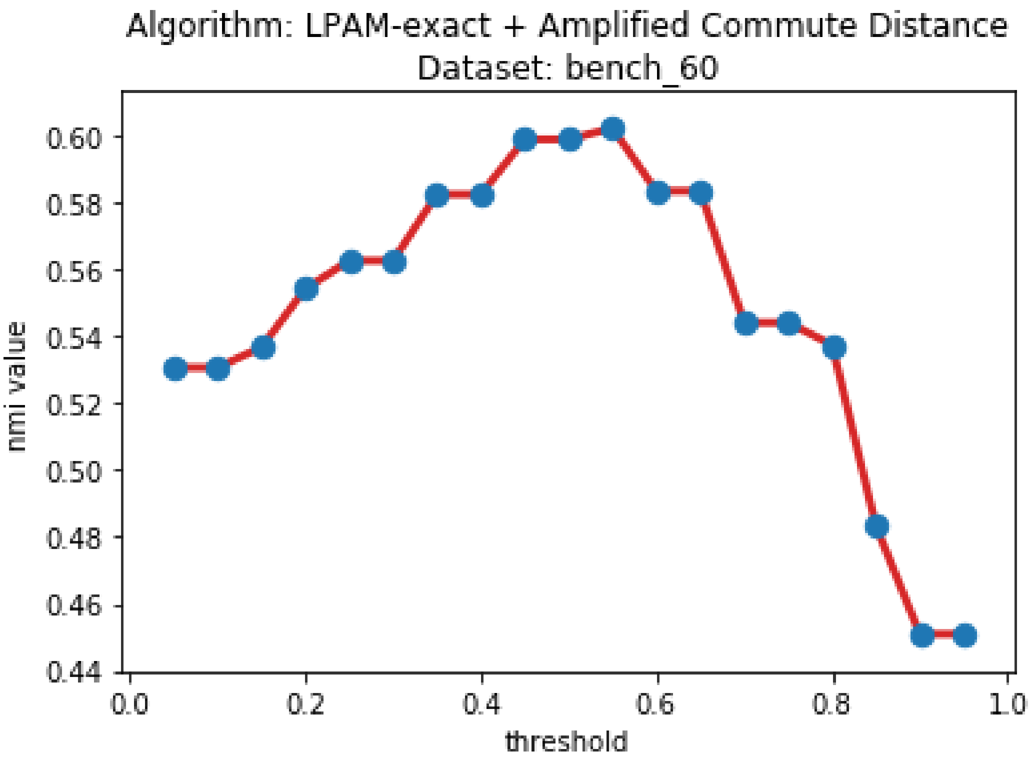

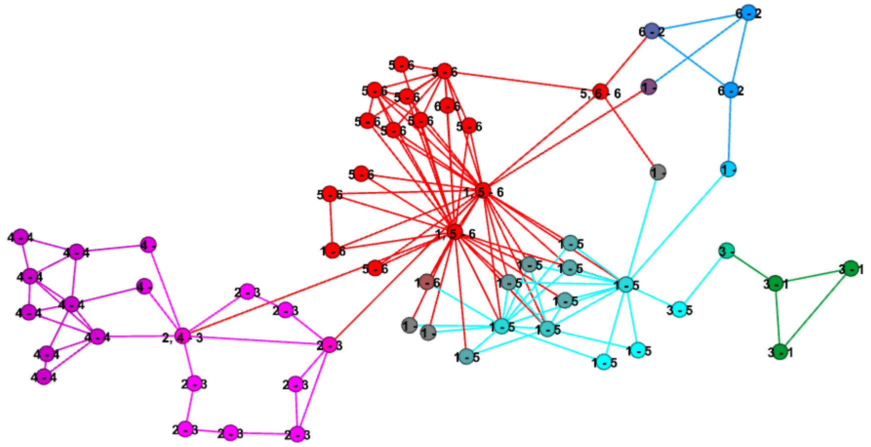

bench_60

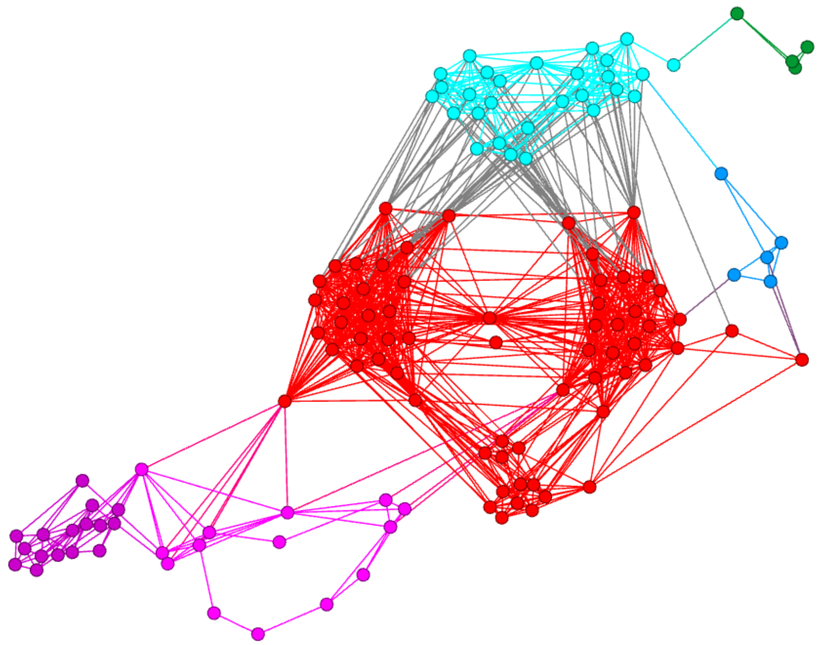

bench_60 - line graph

8 bench_60 dense (mu = 0.25)

```
In [133]: params={}
          params["-t"] = np.arange(0.05, 1.0, 0.05)
          all_results['bench_60_dense'] = lp_experiment(clustersNumber=6,
                        algorithm = "pmp",
                        distance = "acm",
                        inputFile = "../datasets/bench_60_dense/bench_60_dense_network.dat",
                        groundTruth = "../datasets/bench_60_dense/bench_60_dense_truth.dat",
                        params = params,
                        vertexNumerationShift=0,
                        benchmarkFormat=True, lineGraph=True)
```

```
Output dir name: ../Results/lp_pmp_acm_bench_60_dense
Output file name:
../Results/lp_pmp_acm_bench_60_dense/pmp_bench_60_dense_network_ACM_pmp_6.dat
Force flag detected. Remove *.ser and *.lp files from output dir.
```

```
HBox(children=(IntProgress(value=0, max=19), HTML(value='')))
```

```
Matrix time: 653 Clustering time: 7258 Best ONMI: 0.466922 params: '-t 0.2'
```

```
In [27]: plot_all_params(algorithm = "LPAM-exact + Amplified Commute Distance", dataset =
         "bench_60_dense", all_results = all_results)
```

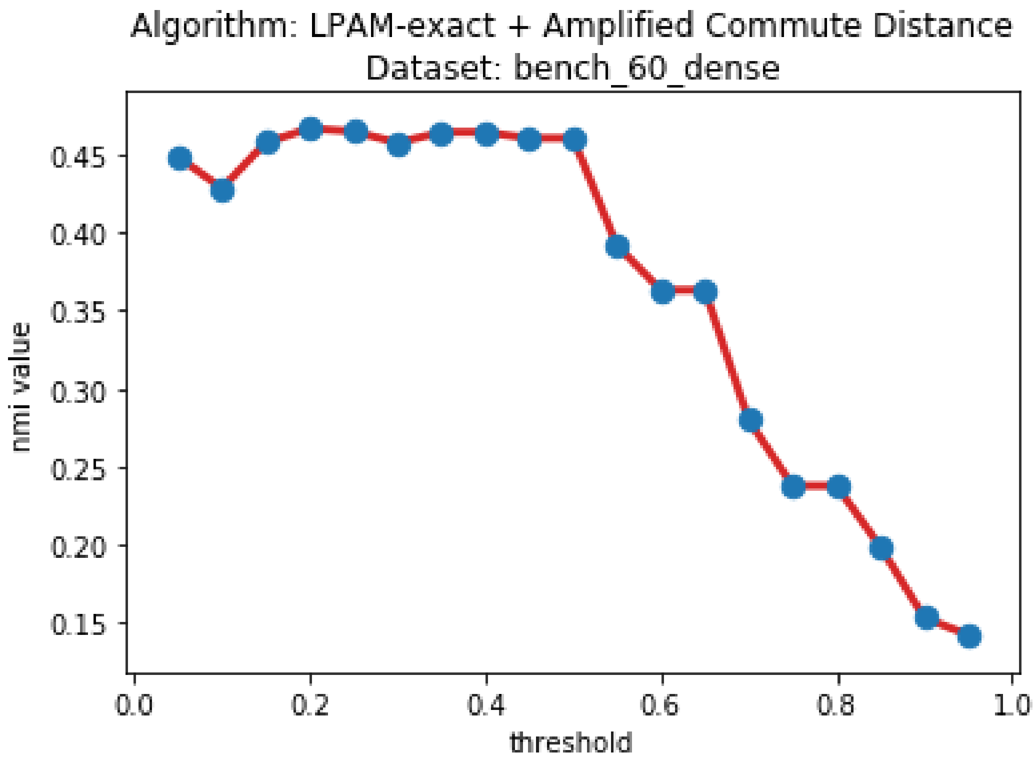

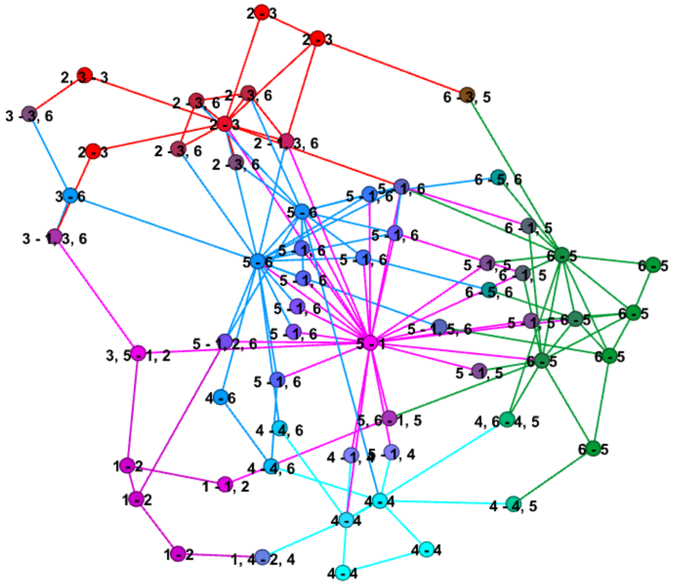

bench_60_dense

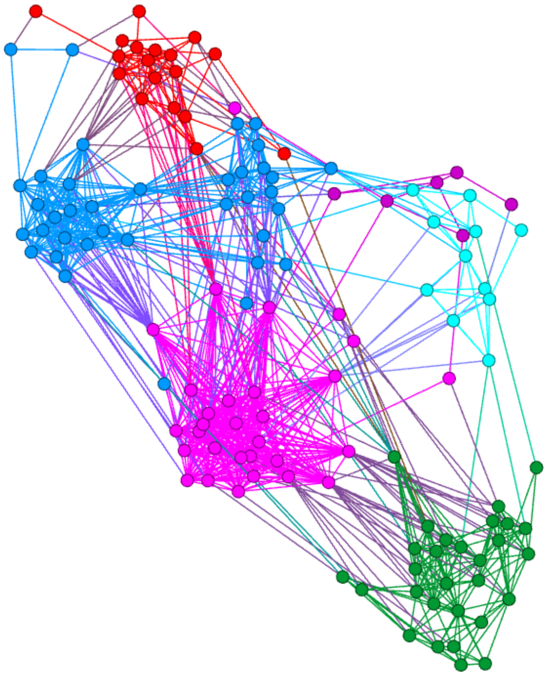

bench_60_dense - line graph

```
In [140]: plt.rcParams["figure.figsize"] = (10,10)
          fName = "lpam_exact_acm_onmi"
          algorithm = "lpam + exact + acm"
          for dataset_name, results in all_results.items():
              xdata=[]
              ydata=[]
              df = pd.DataFrame()
              for param, (nmi, time_m, time_c) in results.items():
                  splited = param.split()
                  xdata.append(float(splited[1]))
                  ydata.append(nmi)
                  df = df.append({'x': float(splited[1]), 'y': nmi}, ignore_index=True)

              plt.plot(xdata, ydata,  zorder=1)
              plt.scatter(xdata, ydata,s=30,zorder=2)


          plt.legend(list(all_results.keys()))
          plt.xlabel('threshold')
          plt.ylabel('onmi value')
          plt.title('Algorithm: {}'.format(algorithm))

          if (fName is not None):
              png1 = BytesIO()
              plt.savefig(png1, format='png', dpi=400)
              png2 = Image.open(png1)
              ffName = '../figures/{}.tiff'.format(fName)
          #     png2.save(ffName)
              png1.close()
              print("figure saved to: " + ffName)
          plt.show()
```

```
figure saved to: ../figures/lpam_exact_acm_onmi.tiff
```

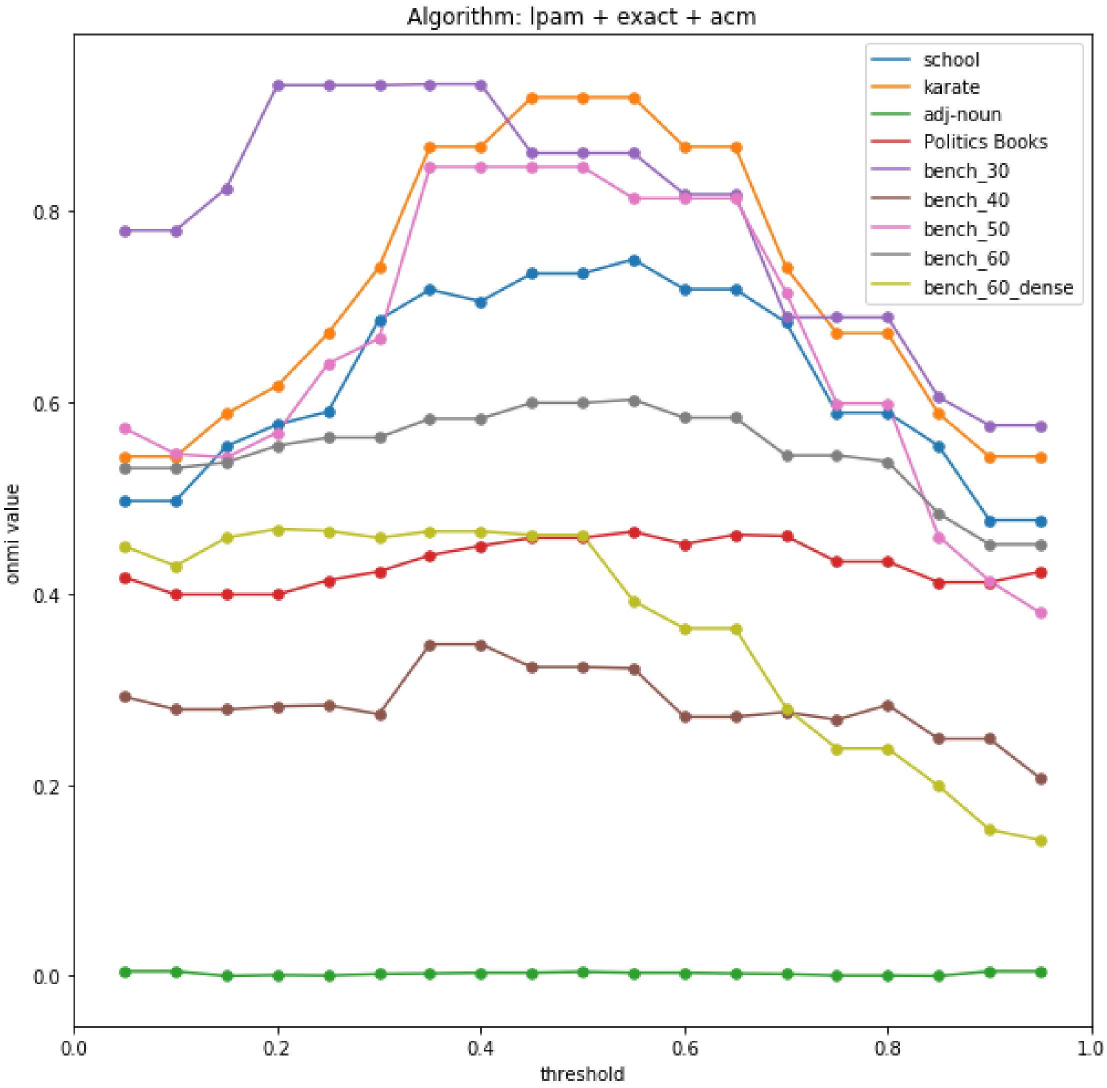

9 Latice 8x8 with 2 clustres

```
In [2]: !java -Djava.library.path=../lib -jar \
        ../lpam/target/Clustering-1.0-SNAPSHOT-jar-with-dependencies.jar \
        -a pmp  -k 2 -d acm -t 0.5 \
        -o ../Results/lp_pmp_acm_lattice_8x8/ \
        -i ../datasets/lattice_8x8/lattice_8x8.gml
```

```
Suffix: lattice_8x8
# Nodes loaded: 64
# Edges loaded: 112
Checking file /home/latna/aponom/lpam-clustering/Scripts/../Results/lp_pmp_acm_lattice
_8x8/d_matrix_lattice_8x8_ACM_pmp_2.ser...not found
Starting calculation of the distance map

Distance map between edges has been calculated.
```

```
Matrix is saved in /home/latna/aponom/lpam-clustering/Scripts/../Results/lp_pmp_acm_la
ttice_8x8/d_matrix_lattice_8x8_ACM_pmp_2.serChecking file /home/latna/aponom/lpam-clus
tering/Scripts/../Results/lp_pmp_acm_lattice_8x8/d_matrix_lattice_8x8_ACM_pmp_2.ser...
not found
Starting calculation of the distance map

Matrix dimensions rows: 112 columns: 112
Model ready
LPSOLVE READ MODEL

Model name:  'PMP' - run #1
Objective:   Minimize(R0)

SUBMITTED
Model size:     9475 constraints,    9586 variables,       475180 non-zeros.
Sets:                                   0 GUB,                  0 SOS.

Using DUAL simplex for phase 1 and PRIMAL simplex for phase 2.
The primal and dual simplex pricing strategy set to 'Devex'.


Relaxed solution       50.7083438606 after       8796 iter is B&B base.

Feasible solution      50.7083438606 after       8796 iter,         0 nodes (gap 0.0%)

Optimal solution       50.7083438606 after       8796 iter,         0 nodes (gap
0.0%).

Relative numeric accuracy ||*|| = 1.18905e-13

 MEMO: lp_solve version 5.5.2.5 for 64 bit OS, with 64 bit REAL variables.
      In the total iteration count 8796, 916 (10.4%) were bound flips.
      There were 34 refactorizations, 0 triggered by time and 4 by density.
       ... on average 231.8 major pivots per refactorization.
      The largest [LUSOL v2.2.1.0] fact(B) had 269777 NZ entries, 1.0x largest basis.
      The maximum B&B level was 1, 0.0x MIP order, 1 at the optimal solution.
      The constraint matrix inf-norm is 1, with a dynamic range of 1.
      Time to load data was 0.083 seconds, presolve used 0.109 seconds,
       ... 25.221 seconds in simplex solver, in total 25.413 seconds.
lp_solve_exit_code: 0
lp_solve_objective_function: 50.70834386062533
LPSOLVE SOLVED MODEL
Distance map between edges has been calculated.

Matrix is saved in /home/latna/aponom/lpam-clustering/Scripts/../Results/lp_pmp_acm_la
ttice_8x8/cluster_edges_hmap_lattice_8x8_ACM_pmp_2.serSorted clusters indices: [[57,
64]]
PMP clusters: [{0={1=2}, 1={1=3}, 2={1=3}, 3={1=3}, 4={1=1, 2=2}, 5={2=3}, 6={2=3},
7={2=2}, 9={1=3}, 11={1=4}, 12={1=4}, 13={1=4}, 14={1=1, 2=3}, 15={2=4}, 16={2=4},
17={2=3}, 19={1=3}, 20={1=4}, 21={1=4}, 22={1=4}, 23={1=1, 2=3}, 25={2=4}, 26={2=4},
27={2=3}, 29={1=3}, 30={1=4}, 31={1=4}, 32={1=4}, 33={1=1, 2=3}, 34={2=4}, 35={2=4},
37={2=3}, 40={1=3}, 42={1=4}, 43={1=4}, 44={1=3, 2=1}, 45={2=4}, 47={2=4}, 49={2=4},
50={2=3}, 52={1=3}, 53={1=4}, 54={1=4}, 55={1=3, 2=1}, 56={2=4}, 57={2=4}, 58={2=4},
59={2=3}, 63={1=3}, 65={1=4}, 67={1=4}, 68={1=3, 2=1}, 69={2=4}, 71={2=4}, 72={2=4},
73={2=3}, 76={1=2}, 78={1=3}, 79={1=3}, 81={1=2, 2=1}, 83={2=3}, 84={2=3}, 85={2=3},
86={2=2}}]
Node: 0 1 100.00%
Node: 1 1 100.00%
```

```
Node: 2 1 100.00%
Node: 3 1 100.00%
Node: 4 1 33.33% 2 66.67%
Node: 5 2 100.00%
Node: 6 2 100.00%
Node: 7 2 100.00%
Node: 9 1 100.00%
Node: 11 1 100.00%
Node: 12 1 100.00%
Node: 13 1 100.00%
Node: 14 1 25.00% 2 75.00%
Node: 15 2 100.00%
Node: 16 2 100.00%
Node: 17 2 100.00%
Node: 19 1 100.00%
Node: 20 1 100.00%
Node: 21 1 100.00%
Node: 22 1 100.00%
Node: 23 1 25.00% 2 75.00%
Node: 25 2 100.00%
Node: 26 2 100.00%
Node: 27 2 100.00%
Node: 29 1 100.00%
Node: 30 1 100.00%
Node: 31 1 100.00%
Node: 32 1 100.00%
Node: 33 1 25.00% 2 75.00%
Node: 34 2 100.00%
Node: 35 2 100.00%
Node: 37 2 100.00%
Node: 40 1 100.00%
Node: 42 1 100.00%
Node: 43 1 100.00%
Node: 44 1 75.00% 2 25.00%
Node: 45 2 100.00%
Node: 47 2 100.00%
Node: 49 2 100.00%
Node: 50 2 100.00%
Node: 52 1 100.00%
Node: 53 1 100.00%
Node: 54 1 100.00%
Node: 55 1 75.00% 2 25.00%
Node: 56 2 100.00%
Node: 57 2 100.00%
Node: 58 2 100.00%
Node: 59 2 100.00%
Node: 63 1 100.00%
Node: 65 1 100.00%
Node: 67 1 100.00%
Node: 68 1 75.00% 2 25.00%
Node: 69 2 100.00%
Node: 71 2 100.00%
Node: 72 2 100.00%
Node: 73 2 100.00%
Node: 76 1 100.00%
Node: 78 1 100.00%
Node: 79 1 100.00%
Node: 81 1 66.67% 2 33.33%
Node: 83 2 100.00%
```

```
Node: 84 2 100.00%
Node: 85 2 100.00%
Node: 86 2 100.00%
Clusters: [{1=[0, 1, 2, 3, 9, 11, 12, 13, 19, 20, 21, 22, 29, 30, 31, 32, 40, 42, 43,
44, 52, 53, 54, 55, 63, 65, 67, 68, 76, 78, 79, 81], 2=[4, 5, 6, 7, 14, 15, 16, 17,
23, 25, 26, 27, 33, 34, 35, 37, 45, 47, 49, 50, 56, 57, 58, 59, 69, 71, 72, 73, 83,
84, 85, 86]}]
The work has been done successfully!
```

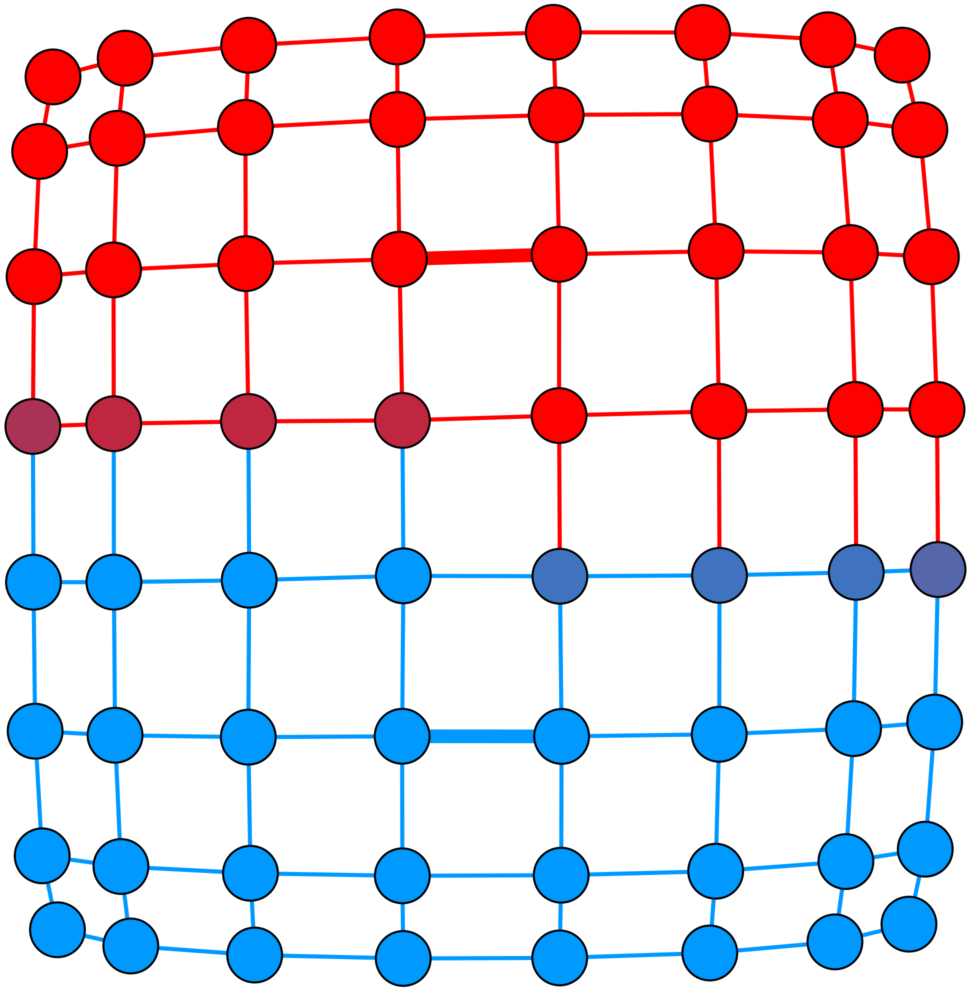

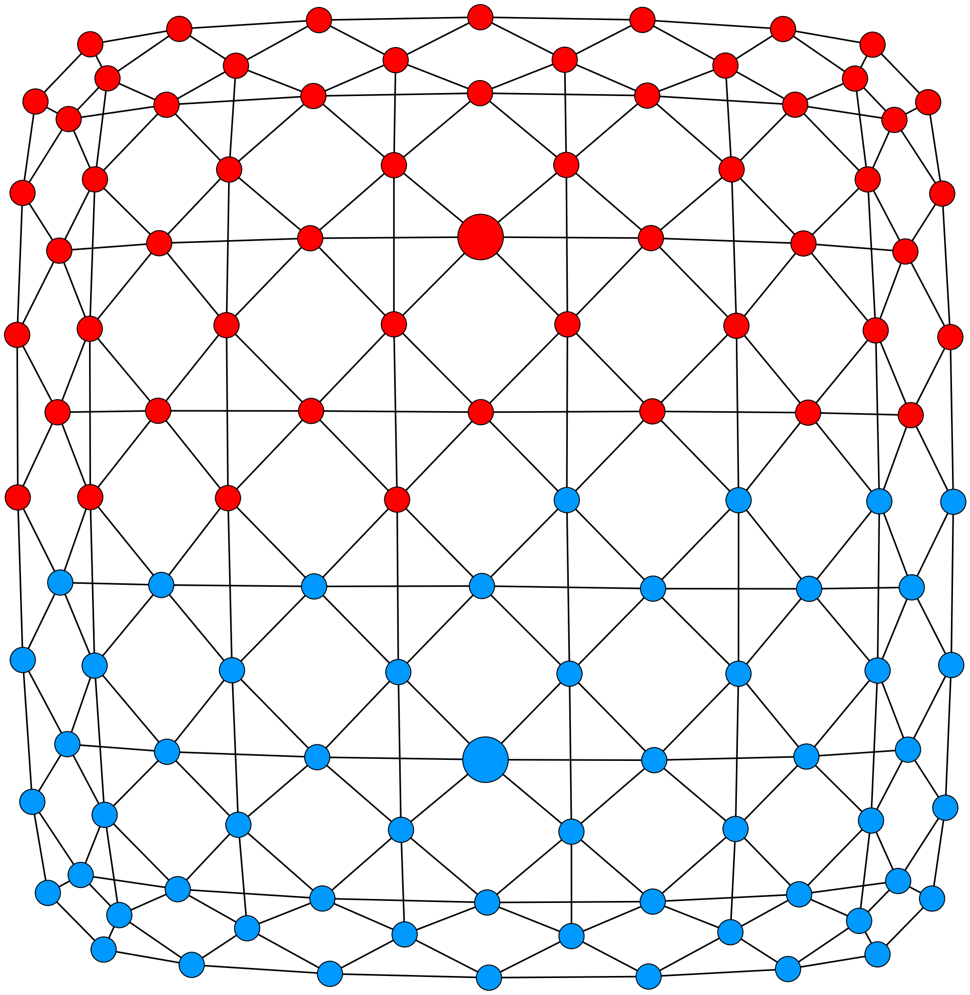

10 Latice 8x8 with 4 clustres

In [75]: !ls ../datasets/lattice_8x8/lattice_8x8.gml

```
lattice_8x8.gephi  lattice_8x8.gml
```

In [2]: !java -Djava.library.path=../lib -jar ../lpam/target/Clustering-1.1-jar-with-dependencies.jar -a pmp -o ../Results/lp_pmp_acm_lattice_8x8/ -i ../datasets/lattice_8x8/lattice_8x8.gml -k 4 -d acm -t 0.5

```
Suffix: lattice_8x8
# Nodes loaded: 64
# Edges loaded: 112
Checking file /home/latna/aponom/lpam-clustering/Scripts/../Results/lp_pmp_acm_lattice
_8x8/d_matrix_lattice_8x8_ACM_pmp_4.ser...found
Distance map between edges has been loaded.

Checking file /home/latna/aponom/lpam-clustering/Scripts/../Results/lp_pmp_acm_lattice
_8x8/cluster_edges_hmap_lattice_8x8_ACM_pmp_4.ser...found
Clustering map has been loaded.
```

```
Sorted clusters indices: [[27, 34, 71, 87]]
PMP clusters: [{0={1=2}, 1={1=3}, 2={1=3}, 3={1=3}, 4={1=1, 2=2}, 5={2=3}, 6={2=3},
7={2=2}, 9={1=3}, 11={1=4}, 12={1=4}, 13={1=4}, 14={1=1, 2=3}, 15={2=4}, 16={2=4},
17={2=3}, 19={1=3}, 20={1=4}, 21={1=4}, 22={1=4}, 23={1=1, 2=3}, 25={2=4}, 26={2=4},
27={2=3}, 29={1=2, 3=1}, 30={1=3, 3=1}, 31={1=3, 3=1}, 32={1=3, 3=1}, 33={1=1, 2=3},
34={2=4}, 35={2=4}, 37={2=3}, 40={3=3}, 42={3=4}, 43={3=4}, 44={3=3, 4=1}, 45={2=1,
4=3}, 47={2=1, 4=3}, 49={2=1, 4=3}, 50={2=1, 4=2}, 52={3=3}, 53={3=4}, 54={3=4},
55={3=3, 4=1}, 56={4=4}, 57={4=4}, 58={4=4}, 59={4=3}, 63={3=3}, 65={3=4}, 67={3=4},
68={3=3, 4=1}, 69={4=4}, 71={4=4}, 72={4=4}, 73={4=3}, 76={3=2}, 78={3=3}, 79={3=3},
81={3=2, 4=1}, 83={4=3}, 84={4=3}, 85={4=3}, 86={4=2}}]
Node: 0 1 100.00%
Node: 1 1 100.00%
Node: 2 1 100.00%
Node: 3 1 100.00%
Node: 4 1 33.33% 2 66.67%
Node: 5 2 100.00%
Node: 6 2 100.00%
Node: 7 2 100.00%
Node: 9 1 100.00%
Node: 11 1 100.00%
Node: 12 1 100.00%
Node: 13 1 100.00%
Node: 14 1 25.00% 2 75.00%
Node: 15 2 100.00%
Node: 16 2 100.00%
Node: 17 2 100.00%
Node: 19 1 100.00%
Node: 20 1 100.00%
Node: 21 1 100.00%
Node: 22 1 100.00%
Node: 23 1 25.00% 2 75.00%
Node: 25 2 100.00%
Node: 26 2 100.00%
Node: 27 2 100.00%
Node: 29 1 66.67% 3 33.33%
Node: 30 1 75.00% 3 25.00%
Node: 31 1 75.00% 3 25.00%
Node: 32 1 75.00% 3 25.00%
Node: 33 1 25.00% 2 75.00%
Node: 34 2 100.00%
Node: 35 2 100.00%
Node: 37 2 100.00%
Node: 40 3 100.00%
Node: 42 3 100.00%
Node: 43 3 100.00%
Node: 44 3 75.00% 4 25.00%
Node: 45 2 25.00% 4 75.00%
Node: 47 2 25.00% 4 75.00%
Node: 49 2 25.00% 4 75.00%
Node: 50 2 33.33% 4 66.67%
Node: 52 3 100.00%
Node: 53 3 100.00%
Node: 54 3 100.00%
Node: 55 3 75.00% 4 25.00%
Node: 56 4 100.00%
Node: 57 4 100.00%
Node: 58 4 100.00%
Node: 59 4 100.00%
Node: 63 3 100.00%
```

```
Node: 65 3 100.00%
Node: 67 3 100.00%
Node: 68 3 75.00% 4 25.00%
Node: 69 4 100.00%
Node: 71 4 100.00%
Node: 72 4 100.00%
Node: 73 4 100.00%
Node: 76 3 100.00%
Node: 78 3 100.00%
Node: 79 3 100.00%
Node: 81 3 66.67% 4 33.33%
Node: 83 4 100.00%
Node: 84 4 100.00%
Node: 85 4 100.00%
Node: 86 4 100.00%
Clusters: [{1=[0, 1, 2, 3, 9, 11, 12, 13, 19, 20, 21, 22, 29, 30, 31, 32], 2=[4, 5, 6,
7, 14, 15, 16, 17, 23, 25, 26, 27, 33, 34, 35, 37], 3=[40, 42, 43, 44, 52, 53, 54, 55,
63, 65, 67, 68, 76, 78, 79, 81], 4=[45, 47, 49, 50, 56, 57, 58, 59, 69, 71, 72, 73,
83, 84, 85, 86]}]
The work has been done successfully!
```

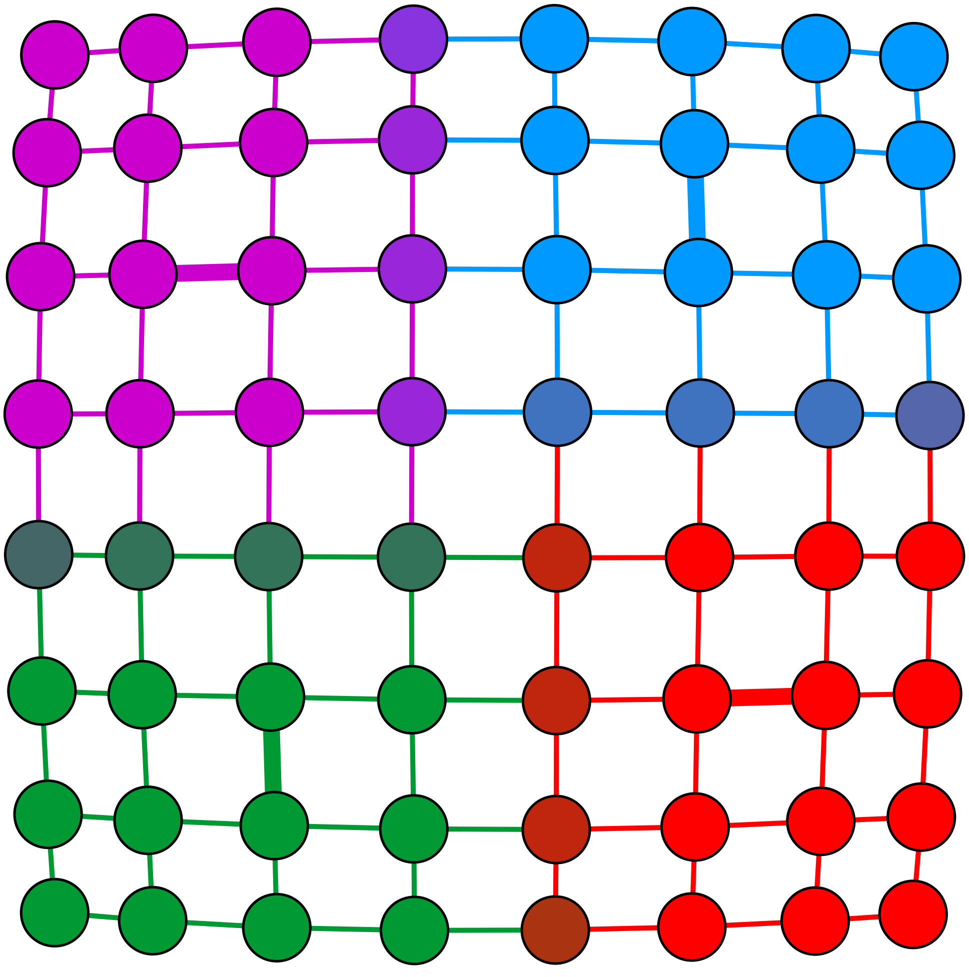

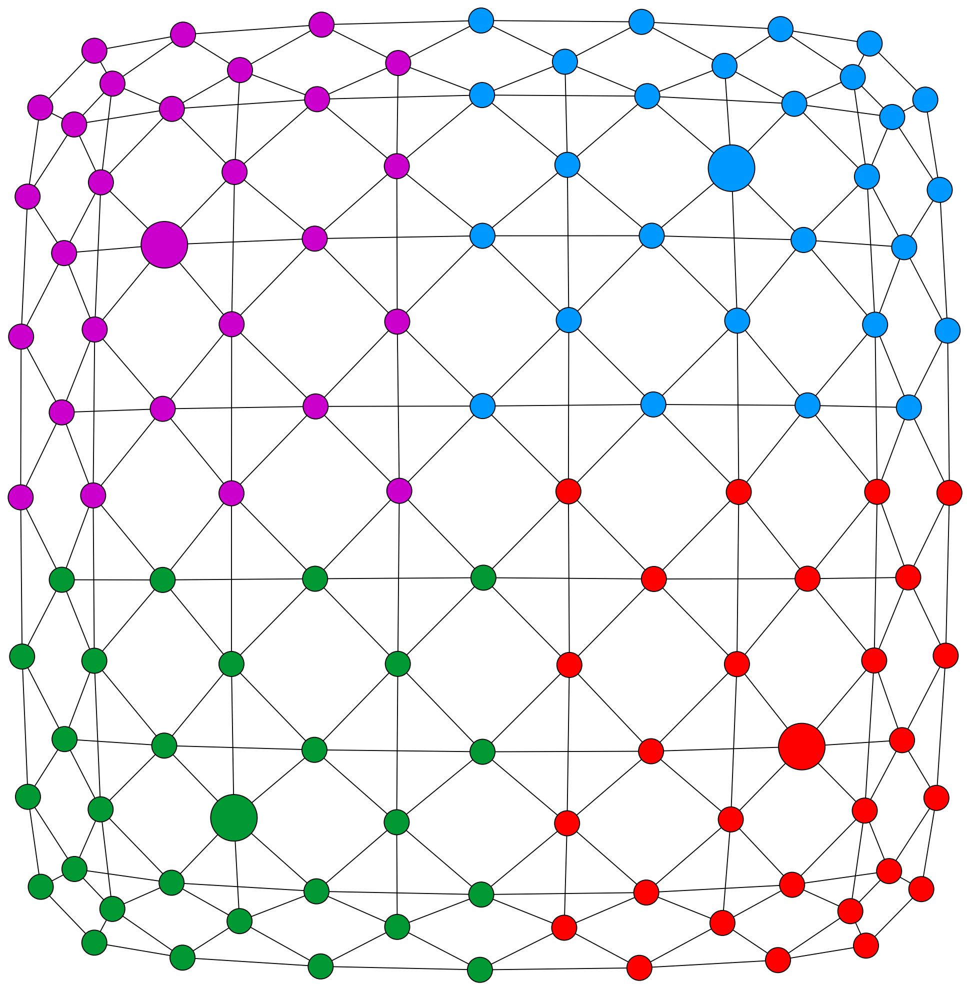

11 FARZ

```
In [25]: params={}
         params["-t"] = np.arange(0.05, 1.0, 0.05)
         all_results = lp_experiment(clustersNumber=2,
                        algorithm = "pmp",
                        distance = "acm",
                        inputFile = "../datasets/FARZ_n_30_m_4_k_2_beta_0.75/network.dat",
                        groundTruth = "../datasets/FARZ_n_30_m_4_k_2_beta_0.75/network.lgt",
                        params = params,
                        vertexNumerationShift=0,
                        benchmarkFormat=True)
```

```
Output dir name: ../Results/lp_pmp_acm_FARZ_n_30_m_4_k_2_beta_0.75
Output file name:
../Results/lp_pmp_acm_FARZ_n_30_m_4_k_2_beta_0.75/pmp_network_ACM_pmp_2.dat


Best ONMI: 0.190791 params: '-t 0.45'
```

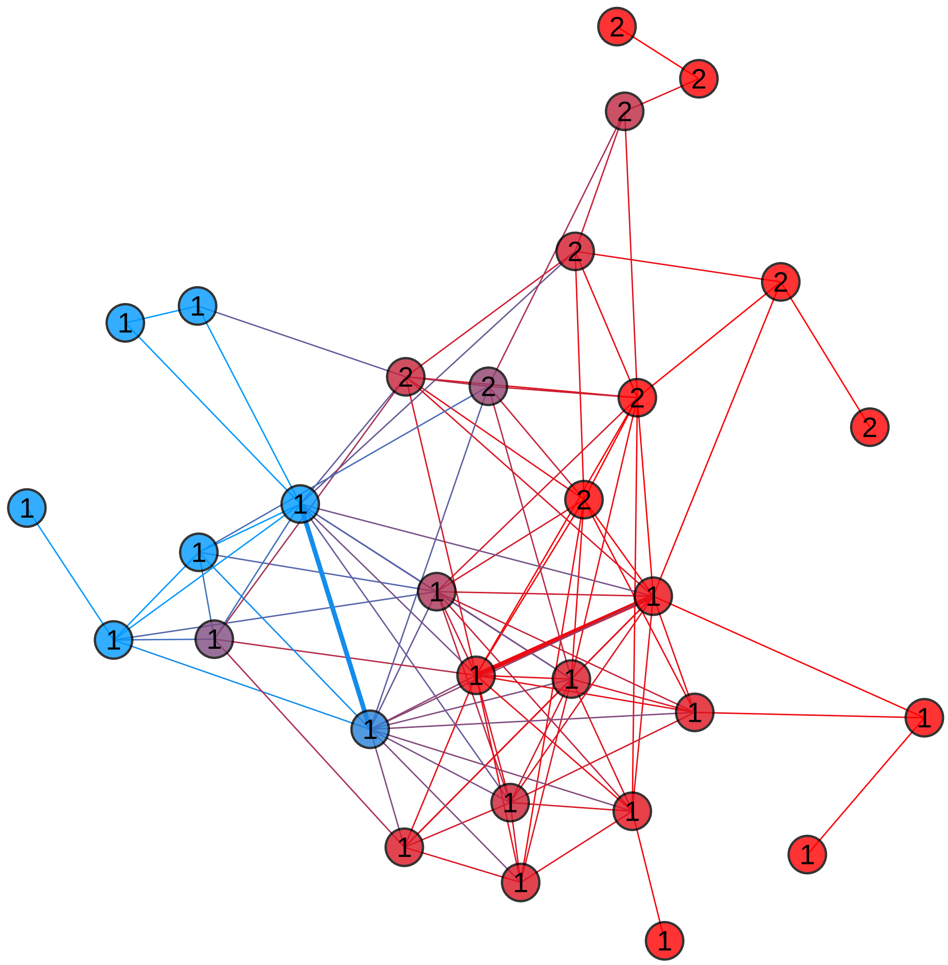

FARZ_n_60_m_5_k_2_beta_0.9

11.1 FARZ_n_60_m_5_k_4_beta_0.75

```
In [111]: params={}
          params["-t"] = np.arange(0.05, 1.0, 0.05)
          all_results = lp_experiment(clustersNumber=2,
                        algorithm = "pmp",
                        distance = "acm",
                        inputFile = "../datasets/FARZ_n_60_m_5_k_4_beta_0.75/network.dat",
```

```
                      groundTruth = "../datasets/FARZ_n_60_m_5_k_4_beta_0.75/network.lgt",
                      params = params,
                      vertexNumerationShift=0,
                      benchmarkFormat=True)
```

```
Output dir name: ../Results/lp_pmp_acm_FARZ_n_60_m_5_k_4_beta_0.75
Output file name:
../Results/lp_pmp_acm_FARZ_n_60_m_5_k_4_beta_0.75/pmp_network_ACM_pmp_2.dat
Force flag detected. Remove *.ser and *.lp files from output dir.
rm: cannot remove '../Results/lp_pmp_acm_FARZ_n_60_m_5_k_4_beta_0.75/*.ser': No such
file or directory
rm: cannot remove '../Results/lp_pmp_acm_FARZ_n_60_m_5_k_4_beta_0.75/*.lp': No such
file or directory
```

```
HBox(children=(IntProgress(value=0, max=19), HTML(value='')))
```

```
Matrix time: 11955 Clustering time: 204947 Best ONMI: 0.729291 params: '-t
0.6000000000000001'
```

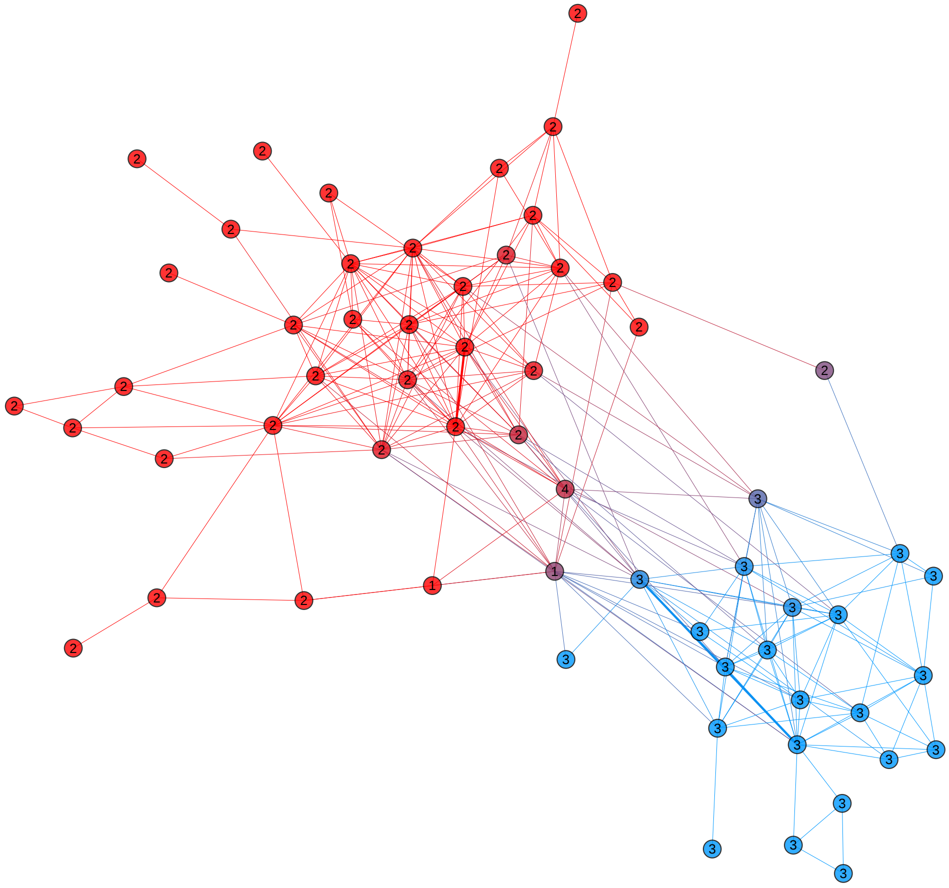

FARZ_n_60_m_5_k_2_beta_0.9

```
In [155]: plot_all_params(algorithm = "LPAM-exact + Amplified Commute Distance", dataset =
          "bench_40", all_results = all_results)
```

11.2 FARZ_n_60_m_5_k_2_beta_0.9

```
In [23]: params={}
         params["-t"] = np.arange(0.05, 1.0, 0.05)
```

```
all_results = lp_experiment(clustersNumber=2,
              algorithm = "pmp",
              distance = "acm",
              inputFile = "../datasets/FARZ_n_60_m_5_k_2_beta_0.9/network.dat",
              groundTruth = "../datasets/FARZ_n_60_m_5_k_2_beta_0.9/network.lgt",
              params = params,
              vertexNumerationShift=0,
              benchmarkFormat=True)
```

```
Output dir name: ../Results/lp_pmp_acm_FARZ_n_60_m_5_k_2_beta_0.9
Output file name:
../Results/lp_pmp_acm_FARZ_n_60_m_5_k_2_beta_0.9/pmp_network_ACM_pmp_2.dat


Best ONMI: 0.561627 params: '-t 0.25'
```

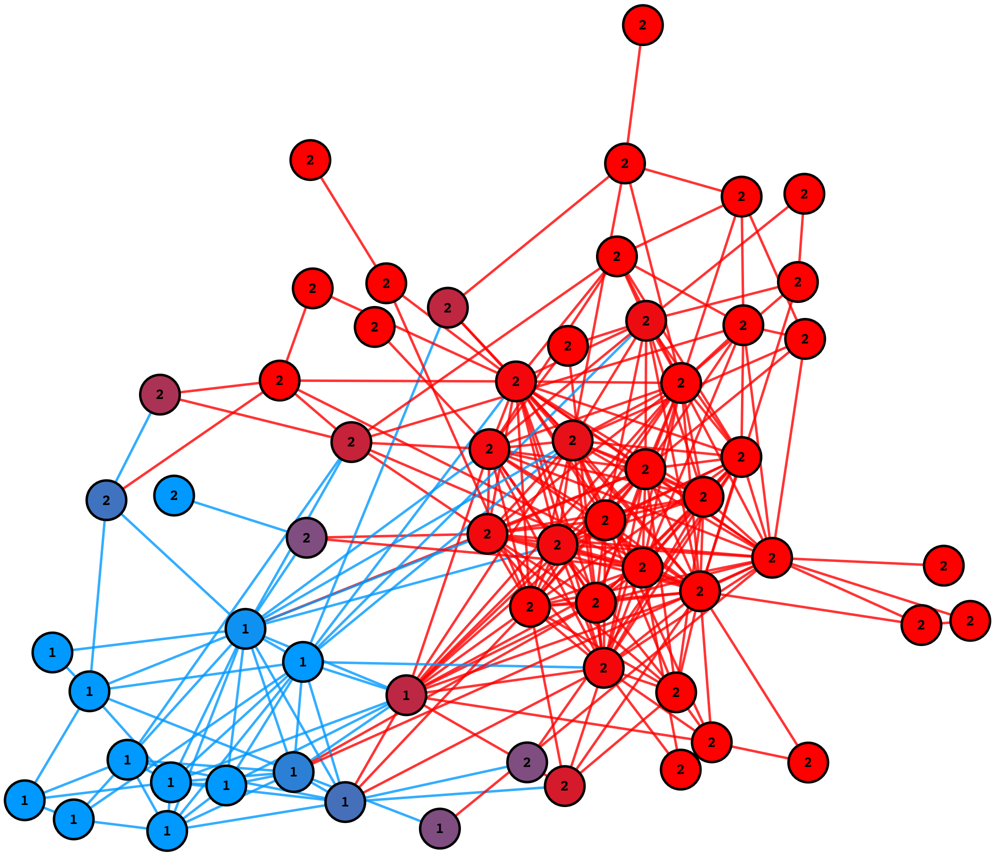

FARZ_n_60_m_5_k_2_beta_0.9

11.3 FARZ_n_200_m_5_k_4_beta_1

```
In [ ]: params={}
        params["-t"] = np.arange(0.05, 1.0, 0.05)
        all_results = lp_experiment(clustersNumber=2,
                      algorithm = "pmp",
                      distance = "acm",
                      inputFile = "../datasets/FARZ_n_200_m_5_k_4_beta_1/network.dat",
```

```
groundTruth = "../datasets/FARZ_n_200_m_5_k_4_beta_1/network.lgt",
params = params,
vertexNumerationShift=0,
benchmarkFormat=True)
```
