# Supplementary material for: Overlapping community detection in networks based on link partitioning and partitioning around medoids
Source: PLoS One. 2021 Aug 25;16(8):e0255717. doi: 10.1371/journal.pone.0255717 (PMC8386890; doi:10.1371/journal.pone.0255717)
Supplement: S2 Appendix — The computational results and the clustering of the heuristic LPAM method with amplified commute distance using the CLARANCE heuristic to solve the k-median problem. (PDF) [file pone.0255717.s002.pdf]

LPAM-Heuristic-Clarans-Amplified-Commute-Distance

April 27, 2020

Link Partitioning Around Medoids with Clarans Heuristic
Distance function: Amplified Commute Distance

In [10]:
```
import numpy as np
import random
random.seed = 108
from tqdm import tqdm_notebook as tqdm
import seaborn as sns
import matplotlib.pyplot as plt
from mpl_toolkits import mplot3d
import pandas as pd
import re
%matplotlib inline
```

In [1]:
```
!java -jar ../lpam/target/Clustering-1.5-jar-with-dependencies.jar
```

```
Missing required options: i, k
usage: PMPClustering <-i input_file> <-k number_of_clusters> [-ftbcd]
 -a,--algorithm <arg>       Specifies algorithm that will be used to find
                            disjoint edge clusters. Possible values:
                            pmp (p-median exact algorithm).
                            kmd (k-medoids heuristic)
                            fkmd (fast k-medioids heuristic)
                            kmn (k-means heuristic).
                            If this option is omitted, the P-Median
                            algorithm will be used.
 -b,--benchmark             use benchmark format (.dat file)
 -d,--distance <arg>        the type of function to measure the distance
                            between nodes.
                            Possible values:
                            sp (shortest path)
                            gd (Generalize Degree)
                            cm (Commute Distance)
                            acm (Amplified Commute Distance).
                            If this option is omitted, the amplified
                            commute distance function will be used.
 -f,--force                 The previous founded solution by lp_solver
                            will not be used. lp_solver will be started
                            forcibly.
                            The flag effects only the PMP exact edge
                            clustering algorithm.
                            By default algorithm will try to get previous
                            founded solution
 -gt,--groundTruth <arg>    load info about ground truth community file
 -i,--input <arg>           path to the input file in GML format
 -k <arg>                   Number of clusters to detect (int)
 -l,--linegraph             produce line graph as output
```

```
-mn,--maxNeighbors <arg>   How many maximum number of neighbors will be
                           used by CLARANS heuristic
                           Default value is 100
-o,--outputDir <arg>       the name of the output file. If option was
                           omitted, the name of the output file
                           will be composed automatically as
                           "{suffix}_{distanceName}_{clusterNumber}_out.g
                           exf"
-s,--solver <arg>          solver type: cplex or lpsolve
-t,--threshold <arg>       Vertex i belongs to cluster C, if node i has
                           fraction of edges in cluster C greater then
                           this value
                           Default value is 0.
```

In [75]:
```
err = None
def lp_experiment(clustersNumber, algorithm, distance, inputFile, groundTruth, params =
{}, vertexNumerationShift=0, benchmarkFormat=False, verbose=False, force=True):
#     path_to_jar = "../lpam/target/Clustering-1.0-SNAPSHOT-jar-with-dependencies.jar"
    path_to_jar = "../lpam/target/Clustering-1.5-jar-with-dependencies.jar"
    datasetName = inputFile.split('/')[-2]
    sufix = inputFile.split('/')[-1].split('.')[0]
    outputDir = "../Results/lp_{}_{}_{}".format(algorithm, distance, datasetName)
    outputFile = outputDir +'/' +"pmp_{3}_{2}_{0}_{1}.dat".format(algorithm,
clustersNumber, distance.upper(), sufix)
    print("Output dir name: {}".format(outputDir) )
    print("Output file name: {}".format(outputFile) )
    if force:
        print("Force flag detected. Remove *.ser and *.lp files from output dir.")
        !rm {outputDir}/*.ser {outputDir}/*.lp
    all_results = {}
    bestParam = "not found"
    nmi_best = 0;
    param_list = list(generate_params(params))
    tmp = None
    for param in tqdm(param_list):
        if benchmarkFormat:
            if verbose:
                !echo java -jar {path_to_jar} -b -a {algorithm} -o {outputDir} -i
{inputFile} -k {clustersNumber} -d {distance} {param}
            tmp=!java -jar {path_to_jar} -b -a {algorithm} -o {outputDir} -i {inputFile}
-k {clustersNumber} -d {distance} {param}
            tmpFile = outputFile
        else:
            tmp=!java -jar {path_to_jar} -a {algorithm} -o {outputDir} -i {inputFile} -k
{clustersNumber} -d {distance} {param}
            if verbose:
                !echo java -jar {path_to_jar} -a {algorithm} -o {outputDir} -i
{inputFile} -k {clustersNumber} -d {distance} {param}
            lines=[]
            with open(outputFile) as f:
                lines = f.readlines()
            tmpFile="tmpFile.dat"
            with open(tmpFile, 'w') as the_file:
                for line in lines:
                    the_file.write(" ".join([str(int(a)+vertexNumerationShift) for a in
line.split()]) + "\n")
        if verbose:
            print(tmp)
        clustering_time = int(re.search(r"Clustering work time: (\w+)"
,'\n'.join(tmp)).groups()[0])
        matrix_time = int(re.search(r"Matrix work time: (\w+)"
,'\n'.join(tmp)).groups()[0])
        output=!../Overlapping-NMI/onmi {groundTruth} {tmpFile}
        nmi=float(output[0].split()[1])
        all_results[param] = (nmi, matrix_time, clustering_time)
```

```
        if nmi >  nmi_best:
            bestParam = param
            nmi_best = nmi
    #restorign solution for the best parameters
    if benchmarkFormat:
        tmp=!java -jar {path_to_jar} -b -a {algorithm} -o {outputDir} -i {inputFile} -gt
{groundTruth} -k {clustersNumber} -d {distance} {bestParam}
        tmpFile = outputFile
    else:
        tmp=!java -jar {path_to_jar} -a {algorithm} -o {outputDir} -i {inputFile} -gt
{groundTruth} -k {clustersNumber} -d {distance} {bestParam}
        lines=[]
        with open(outputFile) as f:
            lines = f.readlines()
        tmpFile="tmpFile.dat"
        with open(tmpFile, 'w') as the_file:
            for line in lines:
                the_file.write(" ".join([str(int(a)+vertexNumerationShift) for a in
line.split()]) + "\n")

    print("Matrix time: {} Clustering time: {} Best ONMI: {} params: '{}'".format(
all_results[param_list[0]][1],all_results[param_list[0]][2], nmi_best, bestParam) )
    return all_results
```

In [53]:
```
def generate_params(params):
    keys = list(params.keys())
    if len(keys) == 1:
        for value in params[keys[0]]:
            yield ( keys[0] + " " + str(value) )
    if len( keys ) > 1:
        for value in params[keys[0]]:
            for remain_params in generate_params({k:params[k] for k in keys[1:]}):
                yield ( keys[0] + " " + str(value) + " " + remain_params )
```

In [58]:
```
def plot_all_params(algorithm, dataset, all_results ):
    xdata=[]
    ydata=[]
    df = pd.DataFrame()
    for param, res in all_results.items():
        nmi = res[0]
        splited = param.split()
        xdata.append(float(splited[1]))
        ydata.append(nmi)
        df = df.append({'x': float(splited[1]), 'y': nmi}, ignore_index=True)

    plt.plot(xdata, ydata, 'C3', zorder=1, lw=3)
    # ax = plt.axes(projection='3d')
    plt.scatter(xdata, ydata,s=70,zorder=2)
    plt.xlabel('threshold')
    plt.ylabel('nmi value');
    plt.title('Algorithm: {}\nDataset: {}'.format(algorithm, dataset));
    plt.show()
```

1 School friendship network

In [55]:
```
params={}
params["-t"] = np.arange(0.05, 1.0, 0.05)
```

In [125]:
```
all_results = lp_experiment(clustersNumber=7,
               algorithm = "kmd",
               distance = "acm",
               inputFile =  "../datasets/school_friendship/school-2.gml",
               groundTruth = "../datasets/school_friendship/truth-school.dat",
               params = params,
               vertexNumerationShift=-1,
               benchmarkFormat=False, verbose = False, force = True)
```

```
Output dir name: ../Results/lp_kmd_acm_school_friendship
Output file name: ../Results/lp_kmd_acm_school_friendship/pmp_school-2_ACM_kmd_7.dat
Force flag detected. Remove *.ser and *.lp files from output dir.
rm: cannot remove '../Results/lp_kmd_acm_school_friendship/*.lp': No such file or
directory
```

```
HBox(children=(IntProgress(value=0, max=19), HTML(value='')))
```

```
Matrix time: 3215 Clustering time: 397 Best ONMI: 0.757913 params: '-t 0.45'
```

```
In [59]: plot_all_params(algorithm = "LPAM-Heuristic-CLARANS + acm", dataset = "School
         Friendship", all_results = all_results)
```

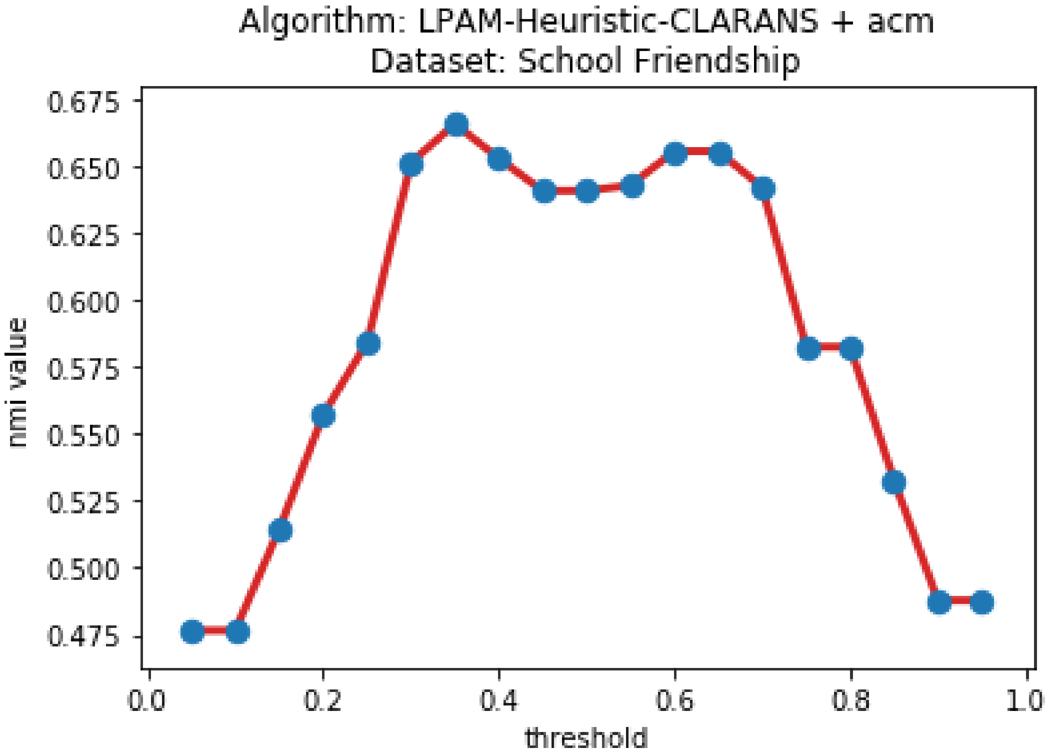

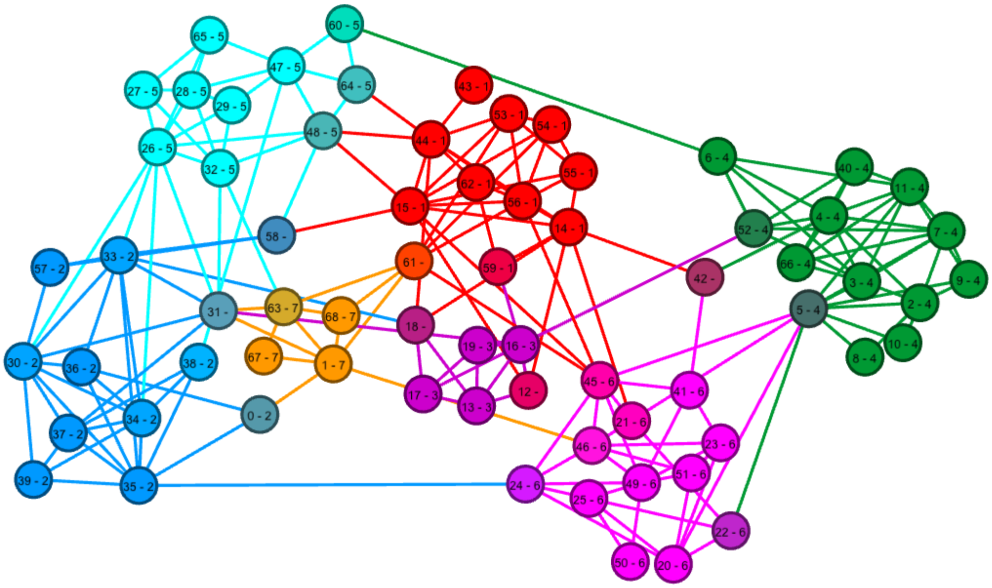

School friendship network

2 Karate Club

```
In [136]: params={}
          params["-t"] = np.arange(0.05, 1.0, 0.05)
          all_results = lp_experiment(clustersNumber=2,
                        algorithm = "kmd",
                        distance = "acm",
                        inputFile =  "../datasets/karate/karate.gml",
                        groundTruth = "../datasets/karate/truth_karate.dat",
                        params = params,
                        vertexNumerationShift=0,
                        benchmarkFormat=False, force=True)
```

```
Output dir name: ../Results/lp_kmd_acm_karate
Output file name: ../Results/lp_kmd_acm_karate/pmp_karate_ACM_kmd_2.dat
Force flag detected. Remove *.ser and *.lp files from output dir.
rm: cannot remove '../Results/lp_kmd_acm_karate/*.lp': No such file or directory
```

```
HBox(children=(IntProgress(value=0, max=19), HTML(value='')))
```

```
Matrix time: 180 Clustering time: 227 Best ONMI: 0.91796 params: '-t 0.45'
```

```
In [137]: plot_all_params(algorithm = "LPAM-Heuristic_CLARANS + acm", dataset = "Karate Club ",
          all_results = all_results)
```

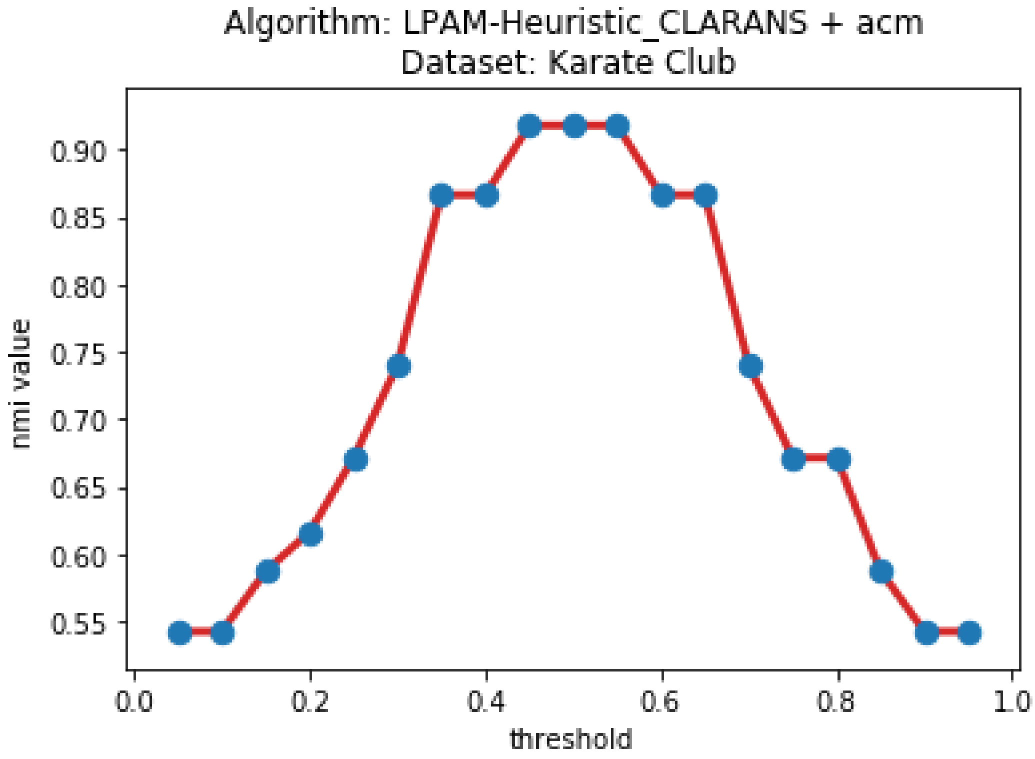
Algorithm: LPAM-Heuristic_CLARANS + acm
Dataset: Karate Club
nmi value
threshold
0.90
0.85
0.80
0.75
0.70
0.65
0.60
0.55
0.0
0.2
0.4
0.6
0.8
1.0

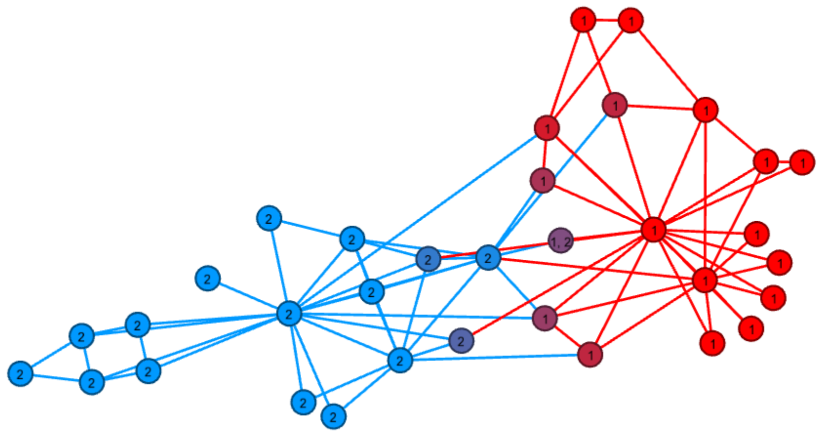

Karate Club

3 American Football League c = 11

```
In [90]: plot_all_params(algorithm = "fkmd + acm; c = 11", dataset = "American Foolball League",
         all_results = all_results)
```

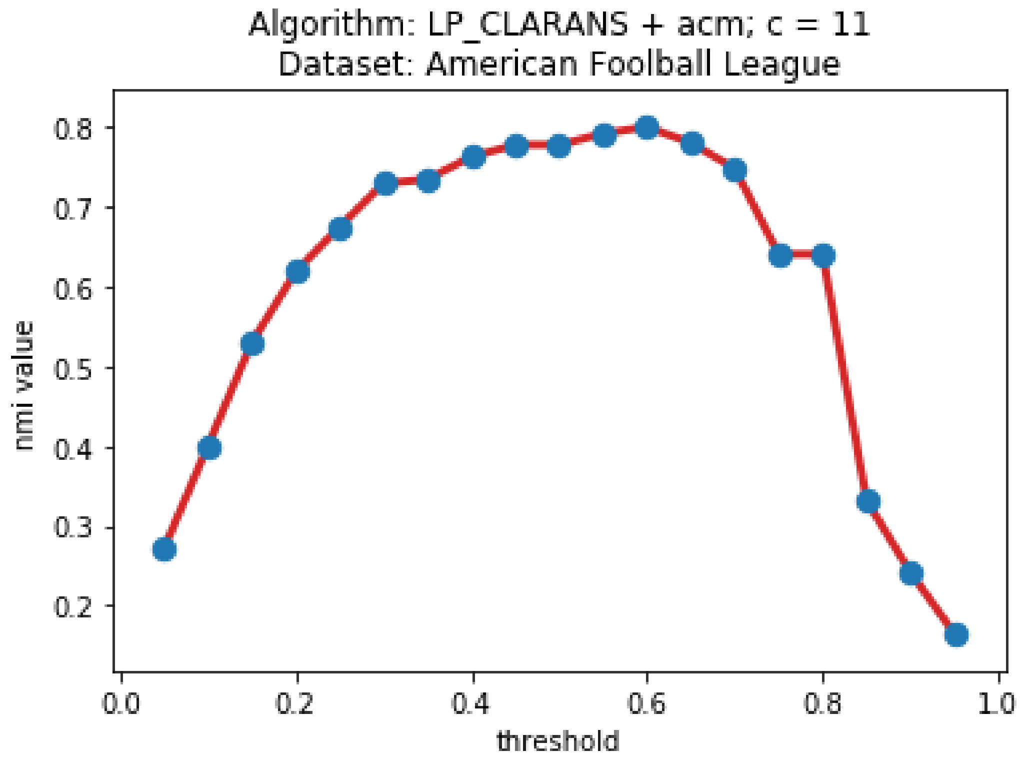


```
In [105]: params={}
          params["-t"] = np.arange(0.05, 1.0, 0.05)
          all_results = lp_experiment(clustersNumber=11,
                        algorithm = "kmd",
                        distance = "acm",
                        inputFile =  "../datasets/football/footballTSEinput.gml",
                        groundTruth = "../datasets/football/truth_footballTSEinput.dat",
                        params = params,
                        vertexNumerationShift=-1,
                        benchmarkFormat=False, verbose = False, force = True)
```

```
Output dir name: ../Results/lp_kmd_acm_football
Output file name: ../Results/lp_kmd_acm_football/pmp_footballTSEinput_ACM_kmd_11.dat
Force flag detected. Remove *.ser and *.lp files from output dir.
rm: cannot remove '../Results/lp_kmd_acm_football/*.lp': No such file or directory
```

```
HBox(children=(IntProgress(value=0, max=19), HTML(value='')))
```

```
Matrix time: 886405 Clustering time: 712 Best ONMI: 0.916974 params: '-t 0.55'
```

```
In [106]: plot_all_params(algorithm = "kmd + acm; c = 11", dataset = "American Foolball League",
          all_results = all_results)
```

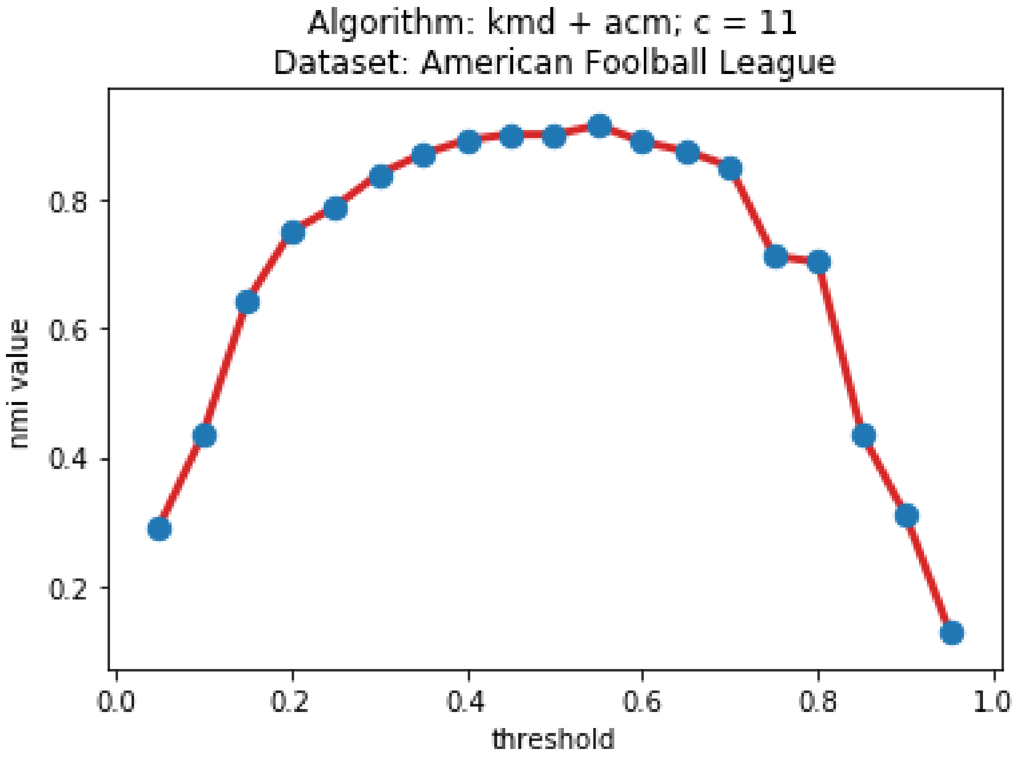

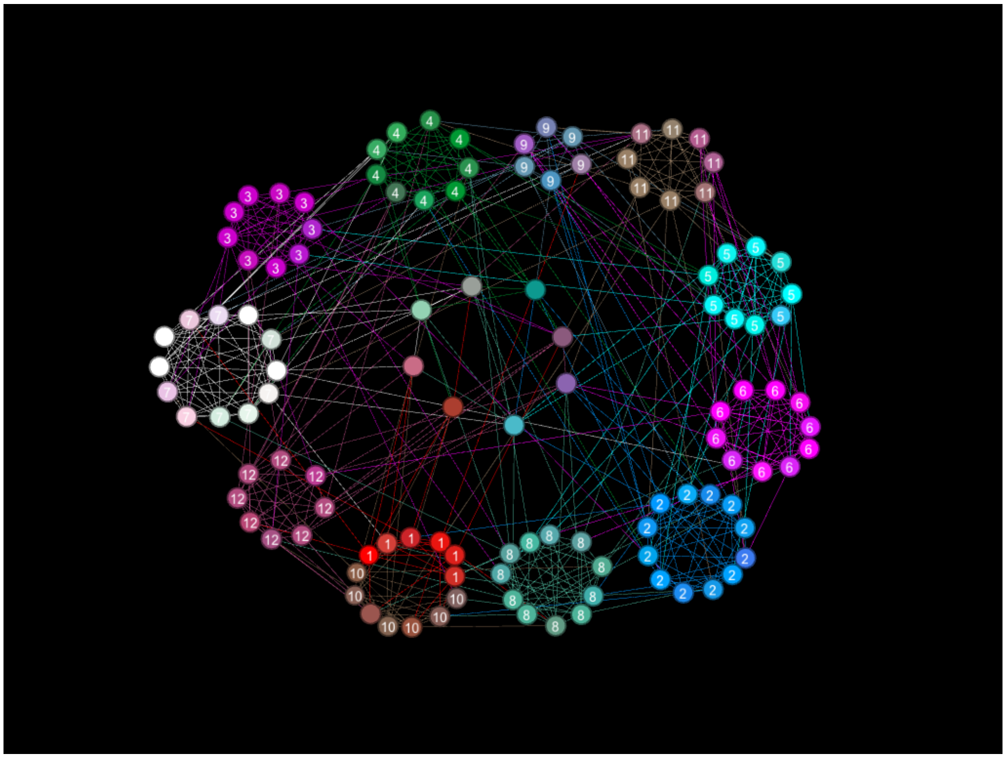

American Football League; onmi: 0.856281; thresould: 0.55

4 American Football League c = 12

```
In [93]: params={}
         params["-t"] = np.arange(0.05, 1.0, 0.05)
         all_results = lp_experiment(clustersNumber=12,
                       algorithm = "kmd",
                       distance = "acm",
                       inputFile =  "../datasets/football/footballTSEinput.gml",
                       groundTruth = "../datasets/football/truth_footballTSEinput.dat",
                       params = params,
                       vertexNumerationShift=-1,
                       benchmarkFormat=False)
```

```
Output dir name: ../Results/lp_kmd_acm_football
Output file name: ../Results/lp_kmd_acm_football/pmp_footballTSEinput_ACM_kmd_12.dat
Force flag detected. Remove *.ser and *.lp files from output dir.
rm: cannot remove '../Results/lp_kmd_acm_football/*.lp': No such file or directory
```

```
HBox(children=(IntProgress(value=0, max=19), HTML(value='')))
```

```
Matrix time: 832362 Clustering time: 660 Best ONMI: 0.871144 params: '-t 0.45'
```

```
In [ ]: plot_all_params(algorithm = "LP_CLARANS + acm", dataset = "American Foolball League",
        all_results = all_results)
```

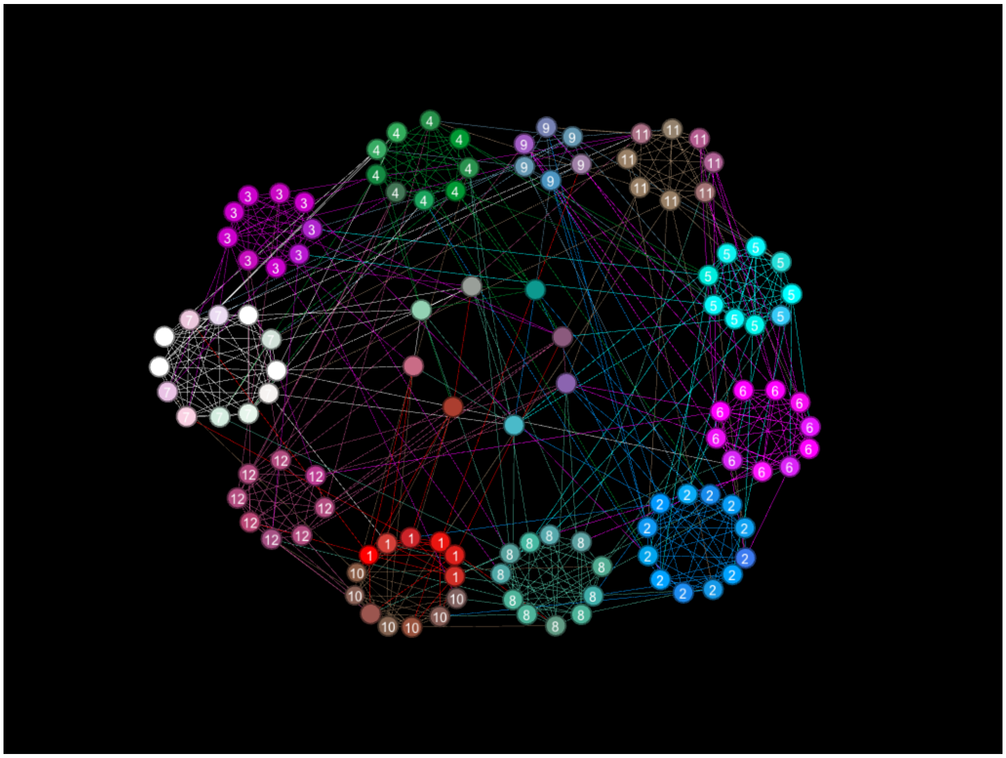

American Football League; onmi: 0.856281; thresould: 0.55

5 Adj-noun

```
In [108]: params={}
          params["-t"] = np.arange(0.05, 1.0, 0.05)
          all_results = lp_experiment(clustersNumber=2,
                        algorithm = "kmd",
                        distance = "acm",
                        inputFile =  "../datasets/adjnoun/adjnoun.dat",
                        groundTruth = "../datasets/adjnoun/truth_adjnoun.dat",
                        params = params,
                        vertexNumerationShift=0,
                        benchmarkFormat=True, force = True)
```

```
Output dir name: ../Results/lp_kmd_acm_adjnoun
Output file name: ../Results/lp_kmd_acm_adjnoun/pmp_adjnoun_ACM_kmd_2.dat
Force flag detected. Remove *.ser and *.lp files from output dir.
rm: cannot remove '../Results/lp_kmd_acm_adjnoun/*.lp': No such file or directory
```

```
HBox(children=(IntProgress(value=0, max=19), HTML(value='')))
```

```
Matrix time: 102112 Clustering time: 553 Best ONMI: 0.00490969 params: '-t 0.1'
```

```
In [153]: plot_all_params(algorithm = "LP_CLARANS + Commute Distance", dataset = "Adj noun",
          all_results = all_results)
```

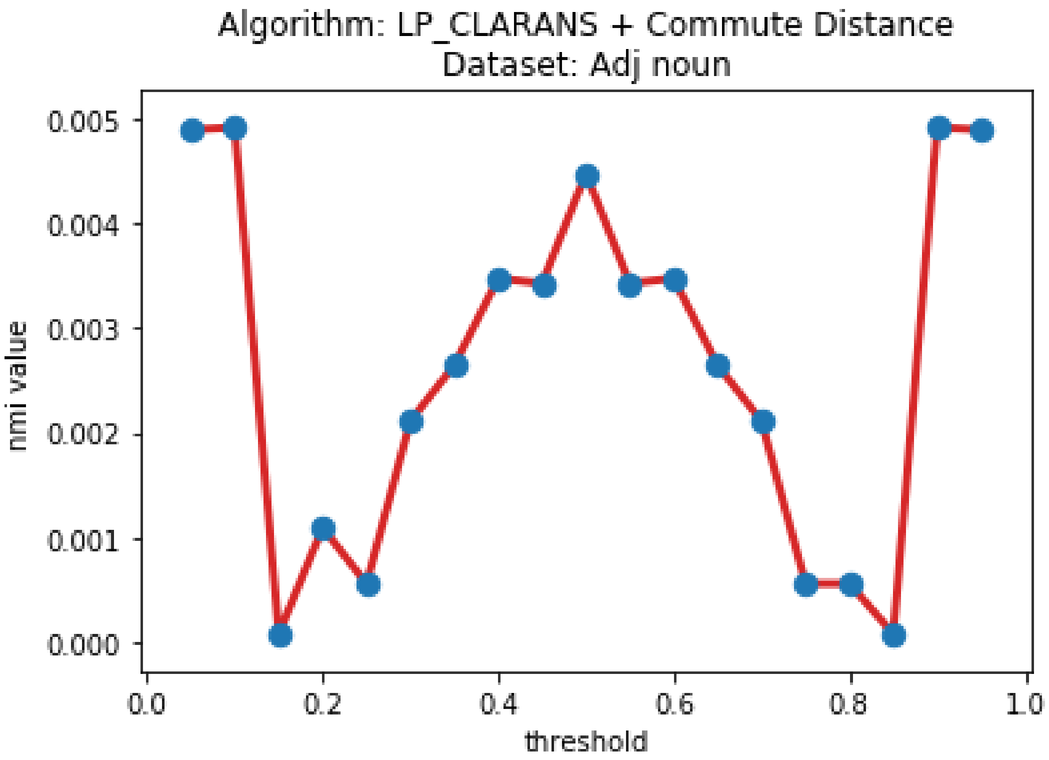

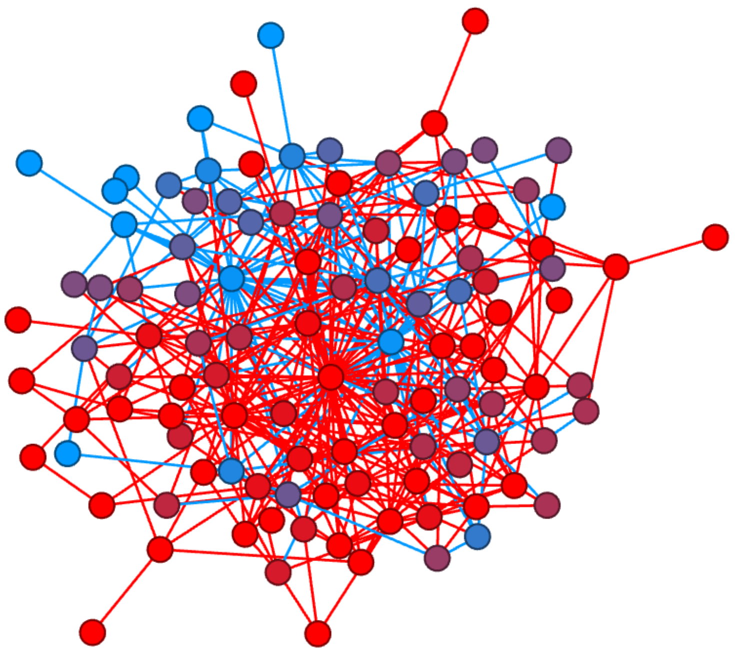

Adj-noun onmi: 0.00490969; thresould: 0.1

6 Political Books

```
In [138]: params={}
          params["-t"] = np.arange(0.05, 1.0, 0.05)
          all_results = lp_experiment(clustersNumber=2,
                        algorithm = "kmd",
                        distance = "acm",
                        inputFile = "../datasets/polbooks/polbooks.dat",
                        groundTruth = "../datasets/polbooks/truth_polbooks.dat",
                        params = params,
                        vertexNumerationShift=0,
                        benchmarkFormat=True)
```

```
Output dir name: ../Results/lp_kmd_acm_polbooks
Output file name: ../Results/lp_kmd_acm_polbooks/pmp_polbooks_ACM_kmd_2.dat
Force flag detected. Remove *.ser and *.lp files from output dir.
rm: cannot remove '../Results/lp_kmd_acm_polbooks/*.lp': No such file or directory
```

```
HBox(children=(IntProgress(value=0, max=19), HTML(value='')))
```

```
Matrix time: 136142 Clustering time: 594 Best ONMI: 0.464154 params: '-t 0.55'
```

```
In [139]: plot_all_params(algorithm = "LP PAM-heuristic + acm", dataset = "Politics Books",
          all_results = all_results)
```

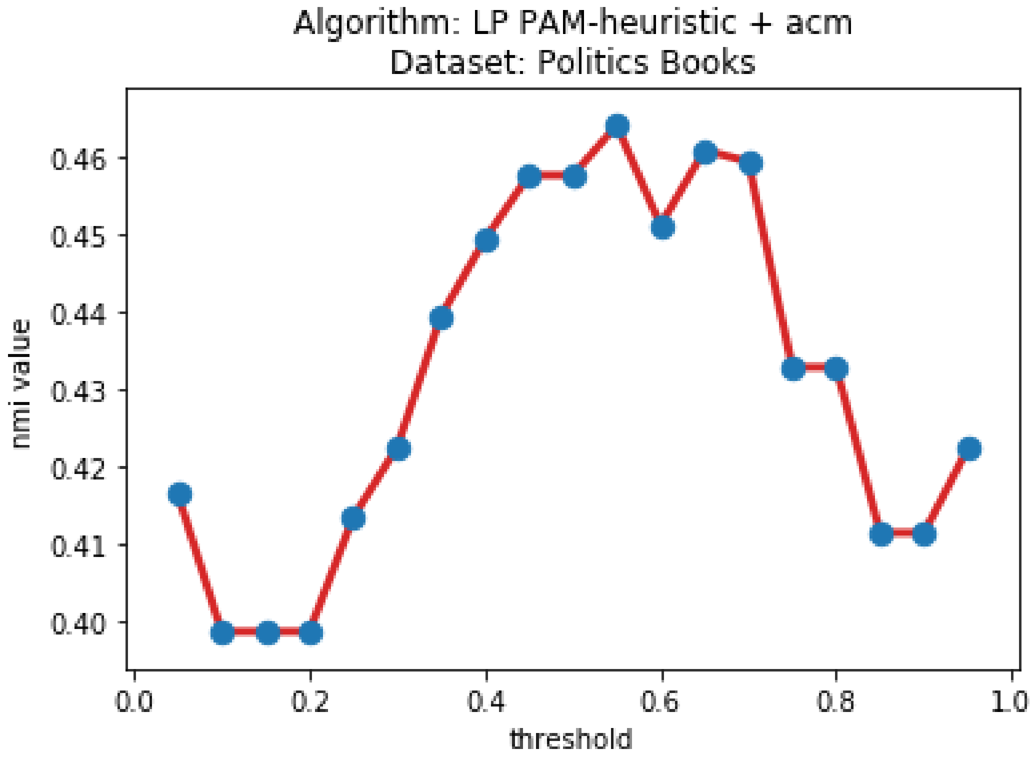

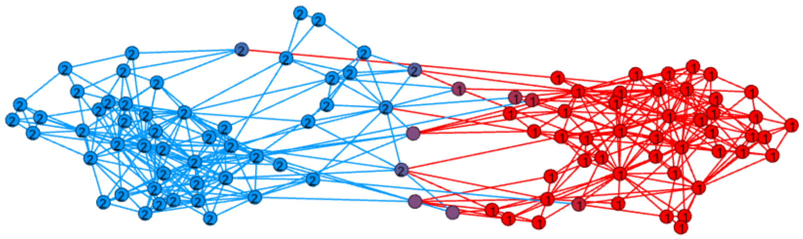

Political books

7 Syntetic Datasets

7.1 bench_30

In [122]:
```
params={}
params["-t"] = np.arange(0.05, 1.0, 0.05)
all_results = lp_experiment(clustersNumber=3,
                 algorithm = "kmd",
                 distance = "acm",
                 inputFile = "../datasets/bench_30/bench_30_network.dat",
                 groundTruth = "../datasets/bench_30/bench_30_truth.dat",
                 params = params,
                 vertexNumerationShift=0,
                 benchmarkFormat=True, force=True)
```

```
Output dir name: ../Results/lp_kmd_acm_bench_30
Output file name: ../Results/lp_kmd_acm_bench_30/pmp_bench_30_network_ACM_kmd_3.dat
Force flag detected. Remove *.ser and *.lp files from output dir.
rm: cannot remove '../Results/lp_kmd_acm_bench_30/*.lp': No such file or directory
```

```
HBox(children=(IntProgress(value=0, max=19), HTML(value='')))
```

```
Matrix time: 153 Clustering time: 179 Best ONMI: 0.931866 params: '-t
0.35000000000000003'
```

In [170]:
```
plot_all_params(algorithm = "LP PAM-heuristic + acm", dataset = "bench_30", all_results
    = all_results)
```

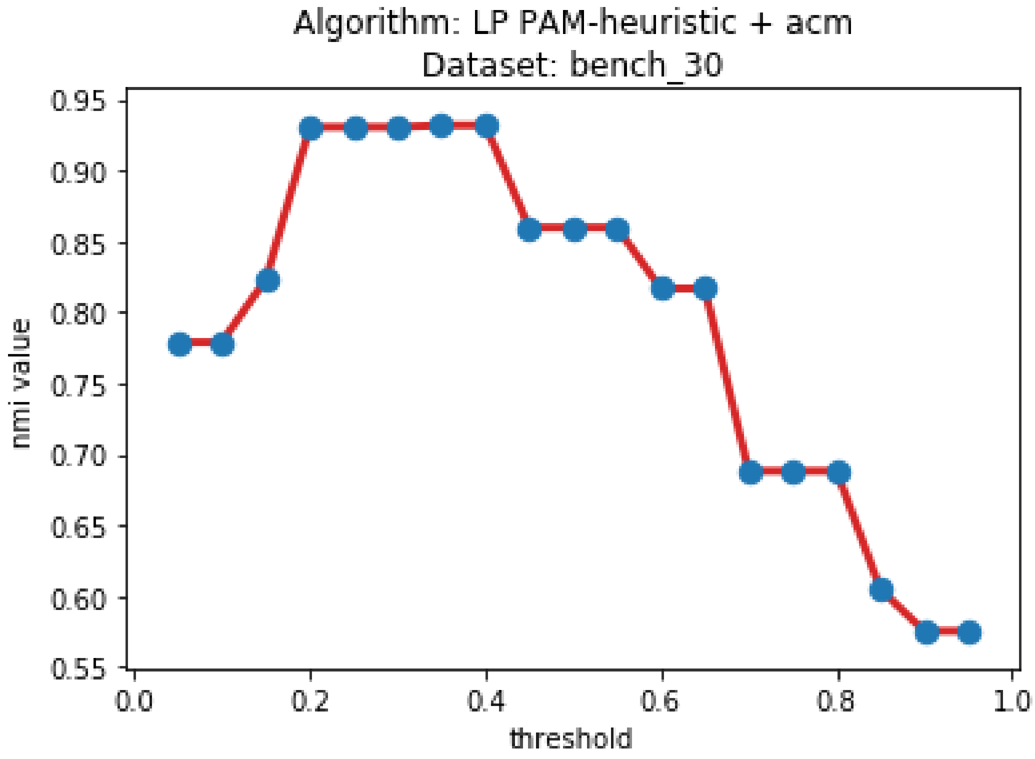

7.2 bench_40

```
In [121]: params={}
          params["-t"] = np.arange(0.05, 1.0, 0.05)
          all_results = lp_experiment(clustersNumber=3,
                        algorithm = "kmd",
                        distance = "acm",
                        inputFile = "../datasets/bench_40/bench_40_network.dat",
                        groundTruth = "../datasets/bench_40/bench_40_truth.dat",
                        params = params,
                        vertexNumerationShift=0,
                        benchmarkFormat=True)
```

```
Output dir name: ../Results/lp_kmd_acm_bench_40
Output file name: ../Results/lp_kmd_acm_bench_40/pmp_bench_40_network_ACM_kmd_3.dat
Force flag detected. Remove *.ser and *.lp files from output dir.
rm: cannot remove '../Results/lp_kmd_acm_bench_40/*.lp': No such file or directory
```

```
HBox(children=(IntProgress(value=0, max=19), HTML(value='')))
```

```
Matrix time: 418 Clustering time: 388 Best ONMI: 0.273879 params: '-t 0.05'
```

```
In [173]: plot_all_params(algorithm = "LP PAM-heuristic + acm", dataset = "bench_40", all_results
          = all_results)
```

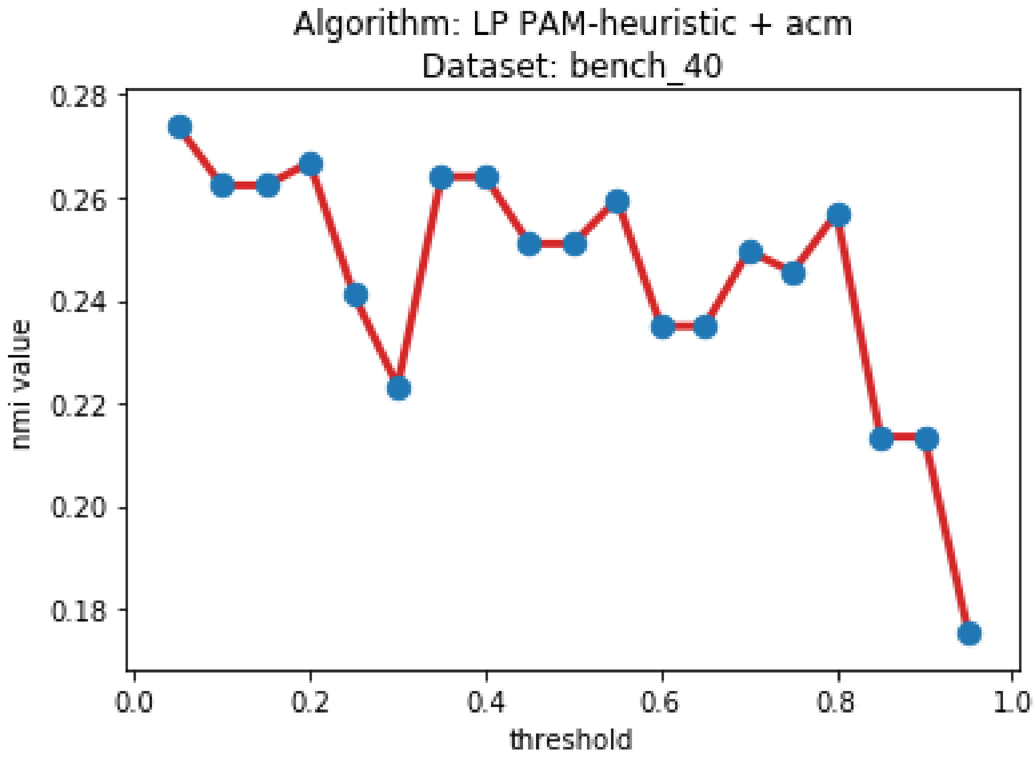

7.3 bench_50

```
In [115]: params={}
          params["-t"] = np.arange(0.05, 1.0, 0.05)
          all_results = lp_experiment(clustersNumber=4,
                        algorithm = "kmd",
                        distance = "acm",
                        inputFile = "../datasets/bench_50/bench_50_network.dat",
                        groundTruth = "../datasets/bench_50/bench_50_truth.dat",
                        params = params,
                        vertexNumerationShift=0,
                        benchmarkFormat=True)
```

```
Output dir name: ../Results/lp_kmd_acm_bench_50
Output file name: ../Results/lp_kmd_acm_bench_50/pmp_bench_50_network_ACM_kmd_4.dat
Force flag detected. Remove *.ser and *.lp files from output dir.
rm: cannot remove '../Results/lp_kmd_acm_bench_50/*.lp': No such file or directory
```

```
HBox(children=(IntProgress(value=0, max=19), HTML(value='')))
```

```
Matrix time: 922 Clustering time: 548 Best ONMI: 0.845306 params: '-t
0.35000000000000003'
```

```
In [177]: plot_all_params(algorithm = "LPAM-Heuristic-CLARANS + acm", dataset = "bench_50",
          all_results = all_results)
```

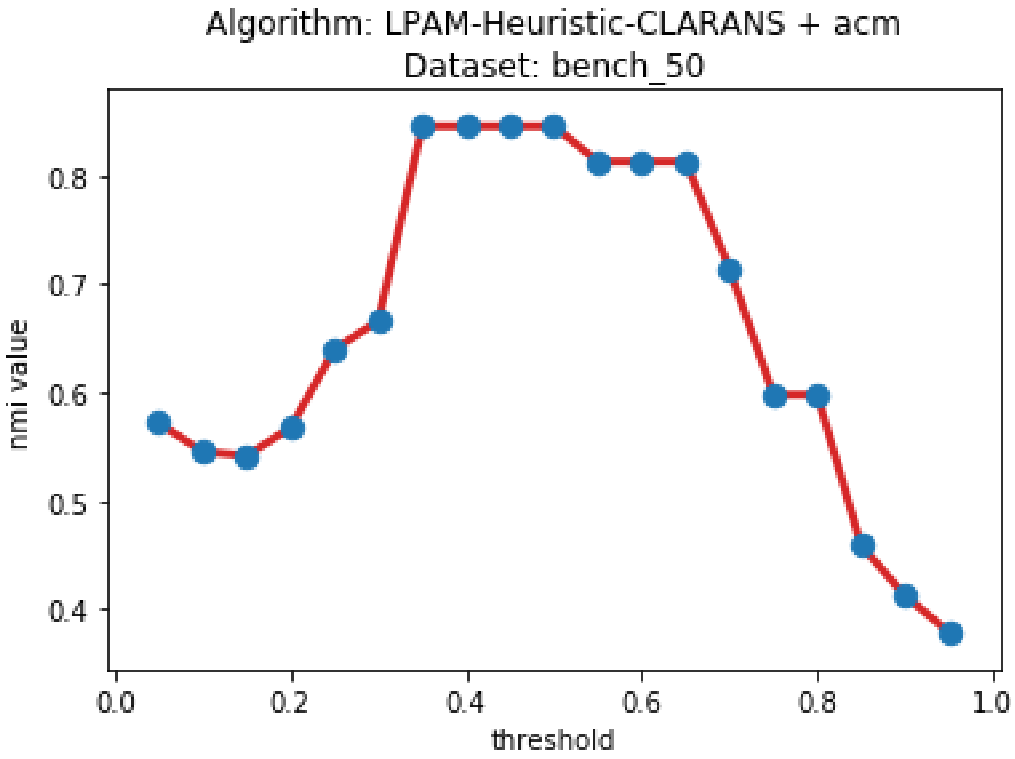

7.4 bench_60

```
In [116]: params={}
          params["-t"] = np.arange(0.05, 1.0, 0.05)
          all_results = lp_experiment(clustersNumber=6,
                        algorithm = "kmd",
                        distance = "acm",
                        inputFile = "../datasets/bench_60/bench_60_network.dat",
                        groundTruth = "../datasets/bench_60/bench_60_truth.dat",
                        params = params,
                        vertexNumerationShift=0,
                        benchmarkFormat=True)
```

```
Output dir name: ../Results/lp_kmd_acm_bench_60
Output file name: ../Results/lp_kmd_acm_bench_60/pmp_bench_60_network_ACM_kmd_6.dat
Force flag detected. Remove *.ser and *.lp files from output dir.
rm: cannot remove '../Results/lp_kmd_acm_bench_60/*.ser': No such file or directory
rm: cannot remove '../Results/lp_kmd_acm_bench_60/*.lp': No such file or directory
```

```
HBox(children=(IntProgress(value=0, max=19), HTML(value='')))
```

```
Matrix time: 676 Clustering time: 336 Best ONMI: 0.586147 params: '-t
0.35000000000000003'
```

```
In [179]: plot_all_params(algorithm = "LPAM-Heuristic-CLARANS + acm", dataset = "bench_60",
          all_results = all_results)
```

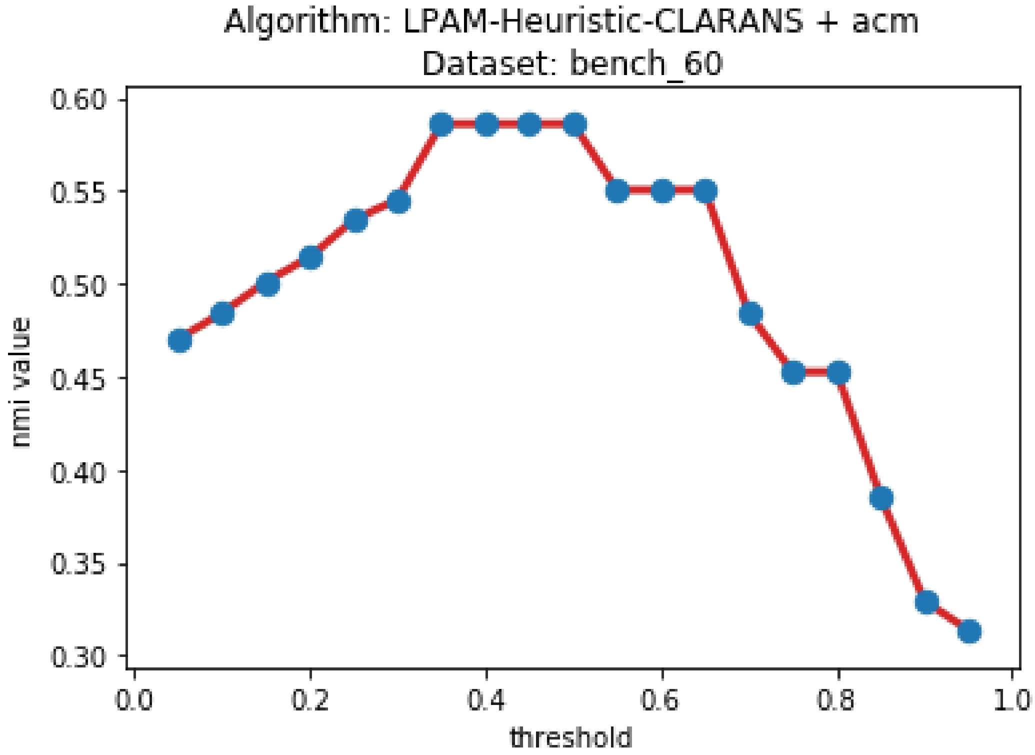

7.5 bench_60_dense

In [131]: `!cat ../datasets/bench_60_dense/bench_60_dense_truth.dat`

```
1 4 20 26 29
2 7 8 23 38 43 44 45 46 56
2 3 31 32 34
5 6 12 14 20 22 24 27 37 50 53
9 10 13 16 18 19 21 28 32 33 35 40 41 42 47 49 55 58 59 60
11 15 17 19 25 27 30 36 39 48 51 52 54 57
```

In [ ]:

In [119]:
```
params={}
params["-t"] = np.arange(0.05, 1.0, 0.05)
all_results = lp_experiment(clustersNumber=3,
              algorithm = "kmd",
              distance = "acm",
              inputFile = "../datasets/bench_60_dense/bench_60_dense_network.dat",
              groundTruth = "../datasets/bench_60_dense/bench_60_dense_truth.dat",
              params = params,
              vertexNumerationShift=0,
              benchmarkFormat=True, force=True)
```

```
Output dir name: ../Results/lp_kmd_acm_bench_60_dense
Output file name:
../Results/lp_kmd_acm_bench_60_dense/pmp_bench_60_dense_network_ACM_kmd_3.dat
Force flag detected. Remove *.ser and *.lp files from output dir.
rm: cannot remove '../Results/lp_kmd_acm_bench_60_dense/*.lp': No such file or
directory
```

```
HBox(children=(IntProgress(value=0, max=19), HTML(value='')))
```

```
Matrix time: 680 Clustering time: 125657 Best ONMI: 0.283863 params: '-t 0.55'
```

In [133]:
```
params={}
params["-t"] = np.arange(0.05, 1.0, 0.05)
all_results = lp_experiment(clustersNumber=5,
              algorithm = "kmd",
              distance = "acm",
              inputFile = "../datasets/bench_60_dense/bench_60_dense_network.dat",
              groundTruth = "../datasets/bench_60_dense/bench_60_dense_truth.dat",
              params = params,
              vertexNumerationShift=0,
              benchmarkFormat=True, force=True)
```

```
Output dir name: ../Results/lp_kmd_acm_bench_60_dense
Output file name:
../Results/lp_kmd_acm_bench_60_dense/pmp_bench_60_dense_network_ACM_kmd_5.dat
Force flag detected. Remove *.ser and *.lp files from output dir.
rm: cannot remove '../Results/lp_kmd_acm_bench_60_dense/*.lp': No such file or
directory
```

```
HBox(children=(IntProgress(value=0, max=19), HTML(value='')))
```

Matrix time: 707 Clustering time: 310535 Best ONMI: 0.416051 params: '-t 0.2'

In [132]:
```
params={}
params["-t"] = np.arange(0.05, 1.0, 0.05)
all_results = lp_experiment(clustersNumber=6,
              algorithm = "kmd",
              distance = "acm",
              inputFile = "../datasets/bench_60_dense/bench_60_dense_network.dat",
              groundTruth = "../datasets/bench_60_dense/bench_60_dense_truth.dat",
              params = params,
              vertexNumerationShift=0,
              benchmarkFormat=True, force=True)
```

Output dir name: ../Results/lp_kmd_acm_bench_60_dense
Output file name:
../Results/lp_kmd_acm_bench_60_dense/pmp_bench_60_dense_network_ACM_kmd_6.dat
Force flag detected. Remove *.ser and *.lp files from output dir.
rm: cannot remove '../Results/lp_kmd_acm_bench_60_dense/*.lp': No such file or directory

HBox(children=(IntProgress(value=0, max=19), HTML(value='')))

Matrix time: 712 Clustering time: 422 Best ONMI: 0.50446 params: '-t 0.35000000000000003'

In [135]:
```
params={}
params["-t"] = np.arange(0.05, 1.0, 0.05)
all_results = lp_experiment(clustersNumber=7,
              algorithm = "kmd",
              distance = "acm",
              inputFile = "../datasets/bench_60_dense/bench_60_dense_network.dat",
              groundTruth = "../datasets/bench_60_dense/bench_60_dense_truth.dat",
              params = params,
              vertexNumerationShift=0,
              benchmarkFormat=True, force=True)
```

Output dir name: ../Results/lp_kmd_acm_bench_60_dense
Output file name:
../Results/lp_kmd_acm_bench_60_dense/pmp_bench_60_dense_network_ACM_kmd_7.dat
Force flag detected. Remove *.ser and *.lp files from output dir.
rm: cannot remove '../Results/lp_kmd_acm_bench_60_dense/*.lp': No such file or directory

HBox(children=(IntProgress(value=0, max=19), HTML(value='')))

Matrix time: 715 Clustering time: 494870 Best ONMI: 0.439694 params: '-t 0.4'

In [120]:
```
plot_all_params(algorithm = "LPAM-Heuristic-CLARANS + acm", dataset = "bench_60_dense",
    all_results = all_results)
```

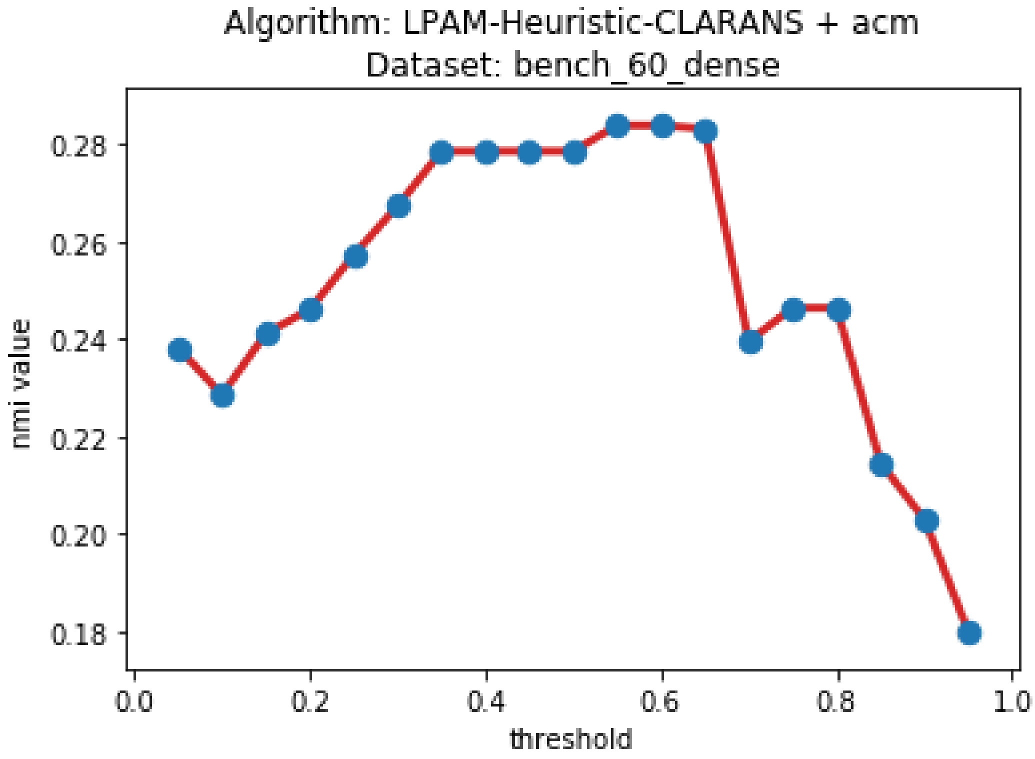

8 Latice

In [24]:
```
!java -Djava.library.path=../lib -jar ../lpam/target/Clustering-1.1-jar-with-
dependencies.jar -a kmd -o ../Results/lp_kmd_acm_lattice_8x8/ -i
../datasets/lattice_8x8/lattice_8x8.gml -k 4 -d acm -t 0.5
```

```
Suffix: lattice_8x8
# Nodes loaded: 64
# Edges loaded: 112
Checking file /home/latna/aponom/lpam-clustering/Scripts/../Results/lp_kmd_acm_lattice
_8x8/d_matrix_lattice_8x8_ACM_kmd_4.ser...not found
Starting calculation of the distance map

Distance map between edges has been calculated.

Matrix is saved in /home/latna/aponom/lpam-clustering/Scripts/../Results/lp_kmd_acm_la
ttice_8x8/d_matrix_lattice_8x8_ACM_kmd_4.serChecking file /home/latna/aponom/lpam-clus
tering/Scripts/../Results/lp_kmd_acm_lattice_8x8/cluster_edges_hmap_lattice_8x8_ACM_km
d_4.ser...not found
Starting calculation clustering map

SLF4J: Failed to load class "org.slf4j.impl.StaticLoggerBinder".
SLF4J: Defaulting to no-operation (NOP) logger implementation
SLF4J: See http://www.slf4j.org/codes.html#StaticLoggerBinder for further details.
Clastering map has been calculated.

Matrix is saved in /home/latna/aponom/lpam-clustering/Scripts/../Results/lp_kmd_acm_la
```

ttice_8x8/cluster_edges_hmap_lattice_8x8_ACM_kmd_4.serSorted clusters indices: [[0, 1, 2, 3]]
PMP clusters: [{0={4=2}, 1={4=3}, 2={4=3}, 3={4=3}, 4={1=2, 4=1}, 5={1=3}, 6={1=3}, 7={1=2}, 9={4=3}, 11={4=4}, 12={4=4}, 13={4=4}, 14={1=3, 4=1}, 15={1=4}, 16={1=4}, 17={1=3}, 19={4=3}, 20={4=4}, 21={4=4}, 22={4=4}, 23={1=3, 4=1}, 25={1=4}, 26={1=4}, 27={1=3}, 29={2=1, 4=2}, 30={2=1, 4=3}, 31={2=1, 4=3}, 32={2=1, 4=3}, 33={1=3, 4=1}, 34={1=4}, 35={1=4}, 37={1=3}, 40={2=3}, 42={2=4}, 43={2=4}, 44={2=3, 3=1}, 45={1=1, 3=3}, 47={1=1, 3=3}, 49={1=1, 3=3}, 50={1=1, 3=2}, 52={2=3}, 53={2=4}, 54={2=4}, 55={2=3, 3=1}, 56={3=4}, 57={3=4}, 58={3=4}, 59={3=3}, 63={2=3}, 65={2=4}, 67={2=4}, 68={2=3, 3=1}, 69={3=4}, 71={3=4}, 72={3=4}, 73={3=3}, 76={2=2}, 78={2=3}, 79={2=3}, 81={2=2, 3=1}, 83={3=3}, 84={3=3}, 85={3=3}, 86={3=2}}]
Node: 0 4 100.00%
Node: 1 4 100.00%
Node: 2 4 100.00%
Node: 3 4 100.00%
Node: 4 1 66.67% 4 33.33%
Node: 5 1 100.00%
Node: 6 1 100.00%
Node: 7 1 100.00%
Node: 9 4 100.00%
Node: 11 4 100.00%
Node: 12 4 100.00%
Node: 13 4 100.00%
Node: 14 1 75.00% 4 25.00%
Node: 15 1 100.00%
Node: 16 1 100.00%
Node: 17 1 100.00%
Node: 19 4 100.00%
Node: 20 4 100.00%
Node: 21 4 100.00%
Node: 22 4 100.00%
Node: 23 1 75.00% 4 25.00%
Node: 25 1 100.00%
Node: 26 1 100.00%
Node: 27 1 100.00%
Node: 29 2 33.33% 4 66.67%
Node: 30 2 25.00% 4 75.00%
Node: 31 2 25.00% 4 75.00%
Node: 32 2 25.00% 4 75.00%
Node: 33 1 75.00% 4 25.00%
Node: 34 1 100.00%
Node: 35 1 100.00%
Node: 37 1 100.00%
Node: 40 2 100.00%
Node: 42 2 100.00%
Node: 43 2 100.00%
Node: 44 2 75.00% 3 25.00%
Node: 45 1 25.00% 3 75.00%
Node: 47 1 25.00% 3 75.00%
Node: 49 1 25.00% 3 75.00%
Node: 50 1 33.33% 3 66.67%
Node: 52 2 100.00%
Node: 53 2 100.00%
Node: 54 2 100.00%
Node: 55 2 75.00% 3 25.00%
Node: 56 3 100.00%
Node: 57 3 100.00%
Node: 58 3 100.00%
Node: 59 3 100.00%

```
Node: 63 2 100.00%
Node: 65 2 100.00%
Node: 67 2 100.00%
Node: 68 2 75.00% 3 25.00%
Node: 69 3 100.00%
Node: 71 3 100.00%
Node: 72 3 100.00%
Node: 73 3 100.00%
Node: 76 2 100.00%
Node: 78 2 100.00%
Node: 79 2 100.00%
Node: 81 2 66.67% 3 33.33%
Node: 83 3 100.00%
Node: 84 3 100.00%
Node: 85 3 100.00%
Node: 86 3 100.00%
Clusters: [{1=[4, 5, 6, 7, 14, 15, 16, 17, 23, 25, 26, 27, 33, 34, 35, 37], 2=[40, 42,
43, 44, 52, 53, 54, 55, 63, 65, 67, 68, 76, 78, 79, 81], 3=[45, 47, 49, 50, 56, 57,
58, 59, 69, 71, 72, 73, 83, 84, 85, 86], 4=[0, 1, 2, 3, 9, 11, 12, 13, 19, 20, 21, 22,
29, 30, 31, 32]}]
The work has been done successfully!
```

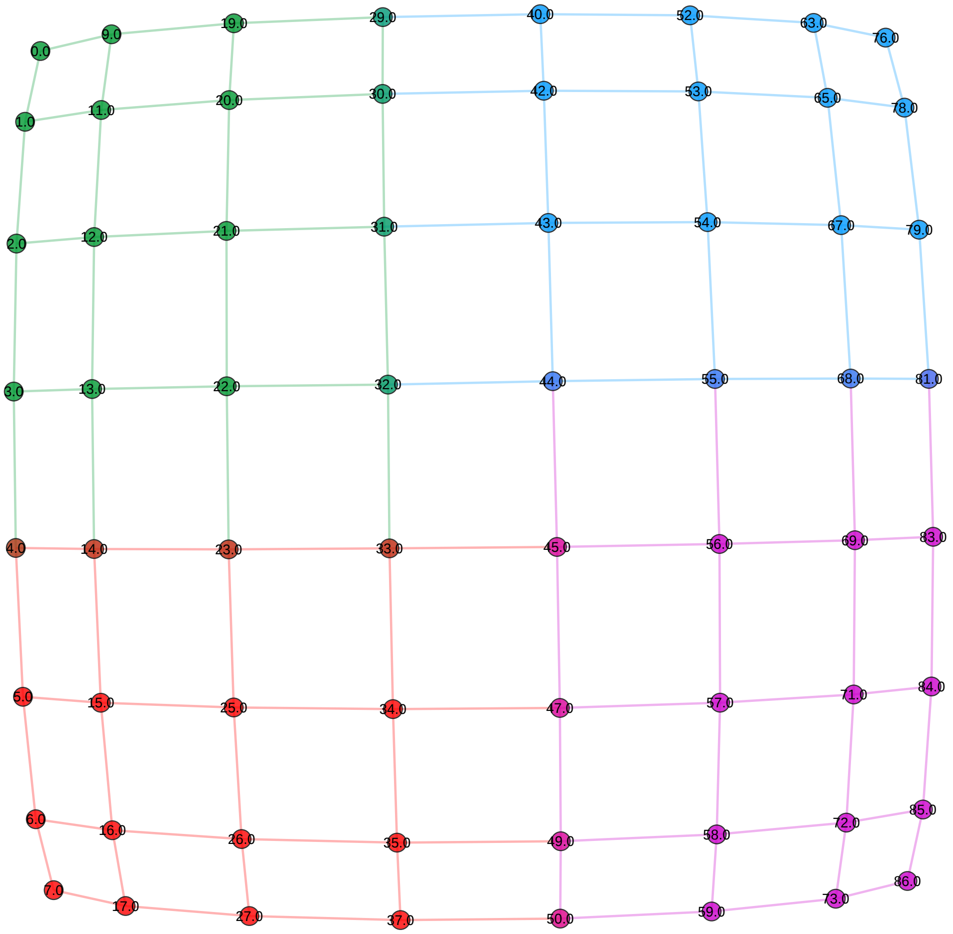

lattice_8x8

9 FARZ

```
In [28]: params={}
         params["-t"] = np.arange(0.05, 1.0, 0.05)
         all_results = lp_experiment(clustersNumber=2,
                       algorithm = "kmd",
                       distance = "acm",
```

```
                        inputFile = "../datasets/FARZ_n_60_m_5_k_2_beta_0.9/network.dat",
                        groundTruth = "../datasets/FARZ_n_60_m_5_k_2_beta_0.9/network.lgt",
                        params = params,
                        vertexNumerationShift=0,
                        benchmarkFormat=True)
```

```
Output dir name: ../Results/lp_kmd_acm_FARZ_n_60_m_5_k_2_beta_0.9
Output file name:
../Results/lp_kmd_acm_FARZ_n_60_m_5_k_2_beta_0.9/pmp_network_ACM_kmd_2.dat



Best ONMI: 0.561627 params: '-t 0.25'
```

9.1 FARZ_n_200_m_5_k_5_beta_1

```
In [31]: params={}
         params["-t"] = np.arange(0.05, 1.0, 0.05)
         all_results = lp_experiment(clustersNumber=5,
                        algorithm = "kmd",
                        distance = "acm",
                        inputFile = "../datasets/FARZ_n_200_m_5_k_5_beta_1/network.dat",
                        groundTruth = "../datasets/FARZ_n_200_m_5_k_5_beta_1/network.lgt",
                        params = params,
                        vertexNumerationShift=0,
                        benchmarkFormat=True)
```

```
Output dir name: ../Results/lp_kmd_acm_FARZ_n_200_m_5_k_5_beta_1
Output file name:
../Results/lp_kmd_acm_FARZ_n_200_m_5_k_5_beta_1/pmp_network_ACM_kmd_5.dat



Best ONMI: 1.0 params: '-t 0.2'
```

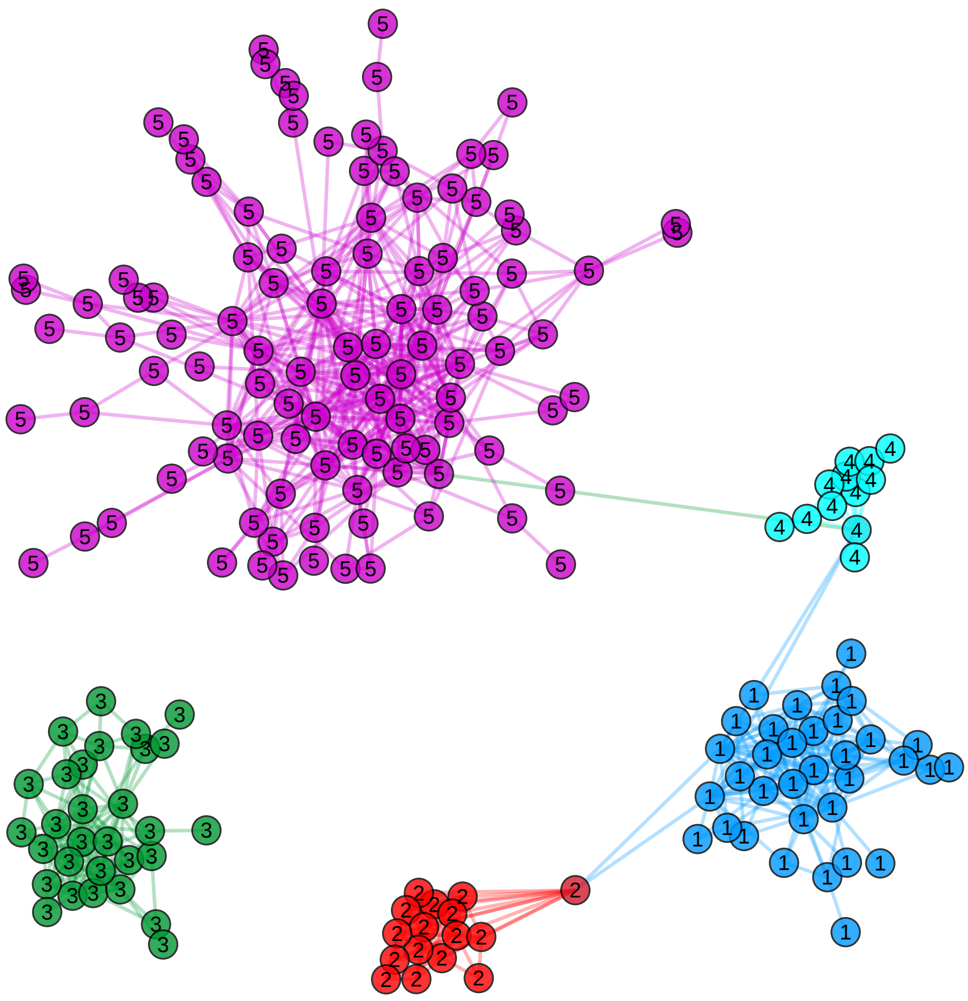

lp_kmd_acm_FARZ_n_200_m_5_k_5_beta_1

9.2 FARZ_n_200_m_5_k_5_beta_0.95

```
In [32]: params={}
         params["-t"] = np.arange(0.05, 1.0, 0.05)
         all_results = lp_experiment(clustersNumber=5,
                       algorithm = "kmd",
                       distance = "acm",
                       inputFile = "../datasets/FARZ_n_200_m_5_k_5_beta_0.95/network.dat",
                       groundTruth = "../datasets/FARZ_n_200_m_5_k_5_beta_0.95/network.lgt",
                       params = params,
                       vertexNumerationShift=0,
                       benchmarkFormat=True)
```

```
Output dir name: ../Results/lp_kmd_acm_FARZ_n_200_m_5_k_5_beta_0.95
Output file name:
../Results/lp_kmd_acm_FARZ_n_200_m_5_k_5_beta_0.95/pmp_network_ACM_kmd_5.dat



Best ONMI: 0.775677 params: '-t 0.3'
```

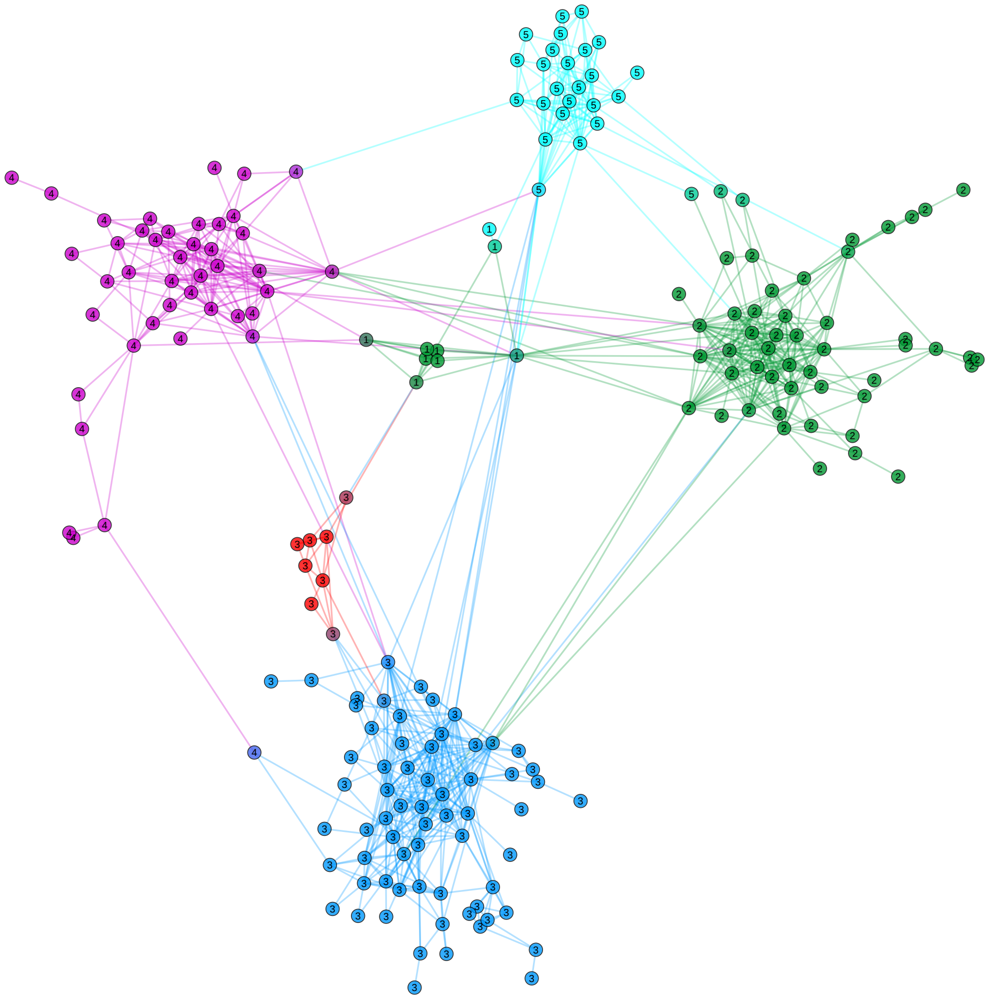

lp_kmd_acm_FARZ_n_200_m_5_k_5_beta_0.95

9.3 FARZ_n_200_m_5_k_5_beta_0.9

```
In [13]: params={}
         params["-t"] = np.arange(0.05, 1.0, 0.05)
         all_results = lp_experiment(clustersNumber=5,
                       algorithm = "kmd",
                       distance = "acm",
                       inputFile = "../datasets/FARZ_n_200_m_5_k_5_beta_0.9/network.dat",
                       groundTruth = "../datasets/FARZ_n_200_m_5_k_5_beta_0.9/network.lgt",
                       params = params,
                       vertexNumerationShift=0,
                       benchmarkFormat=True)
```

```
Output dir name: ../Results/lp_kmd_acm_FARZ_n_200_m_5_k_5_beta_0.9
Output file name:
../Results/lp_kmd_acm_FARZ_n_200_m_5_k_5_beta_0.9/pmp_network_ACM_kmd_5.dat
```

```
HBox(children=(IntProgress(value=0, max=19), HTML(value='')))
```

```
Best ONMI: 0.728205 params: '-t 0.55'
```

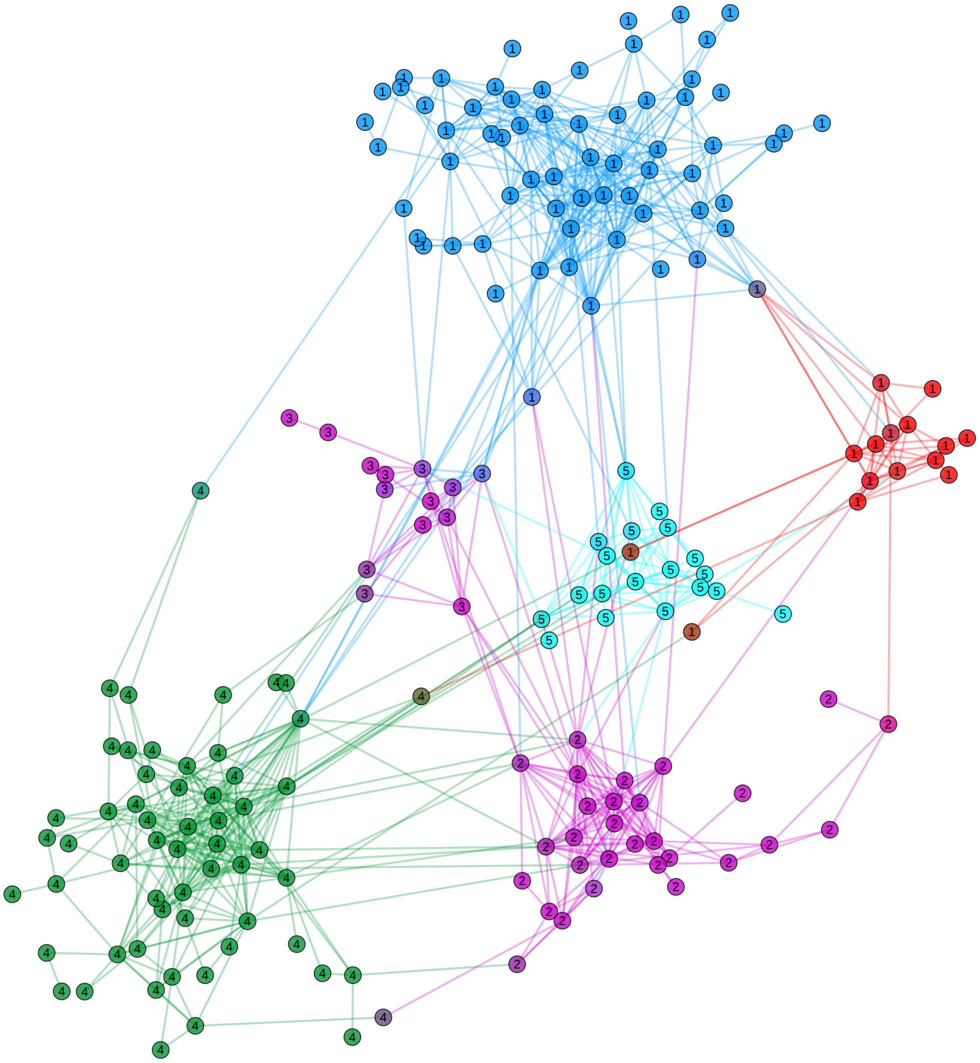

lp_kmd_acm_FARZ_n_200_m_5_k_5_beta_0.9

9.4 FARZ_n_200_m_5_k_5_beta_0.85

```
In [14]: params={}
         params["-t"] = np.arange(0.05, 1.0, 0.05)
         all_results = lp_experiment(clustersNumber=5,
                       algorithm = "kmd",
                       distance = "acm",
                       inputFile = "../datasets/FARZ_n_200_m_5_k_5_beta_0.85/network.dat",
                       groundTruth = "../datasets/FARZ_n_200_m_5_k_5_beta_0.85/network.lgt",
                       params = params,
                       vertexNumerationShift=0,
                       benchmarkFormat=True)
```

```
Output dir name: ../Results/lp_kmd_acm_FARZ_n_200_m_5_k_5_beta_0.85
Output file name:
```

```
../Results/lp_kmd_acm_FARZ_n_200_m_5_k_5_beta_0.85/pmp_network_ACM_kmd_5.dat
```

```
HBox(children=(IntProgress(value=0, max=19), HTML(value='')))
```

```
Best ONMI: 0.818325 params: '-t 0.45'
```

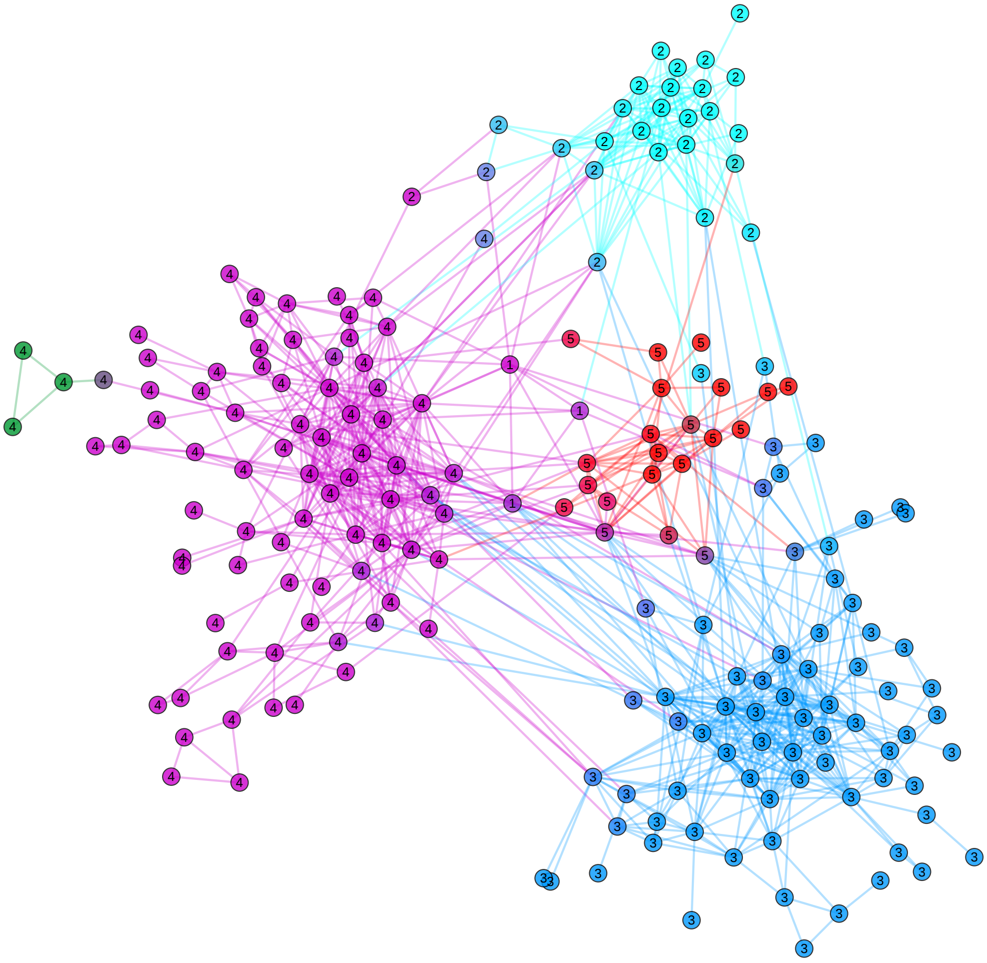

lp_kmd_acm_FARZ_n_200_m_5_k_5_beta_0.85

9.5 FARZ_n_200_m_5_k_5_beta_0.8

In [76]:
```
params={}
params["-t"] = np.arange(0.05, 1.0, 0.05)
all_results = lp_experiment(clustersNumber=5,
              algorithm = "kmd",
              distance = "acm",
              inputFile = "../datasets/FARZ_n_200_m_5_k_5_beta_0.8/network.dat",
              groundTruth = "../datasets/FARZ_n_200_m_5_k_5_beta_0.8/network.lgt",
              params = params,
              vertexNumerationShift=0,
              benchmarkFormat=True)
```

```
Output dir name: ../Results/lp_kmd_acm_FARZ_n_200_m_5_k_5_beta_0.8
Output file name:
../Results/lp_kmd_acm_FARZ_n_200_m_5_k_5_beta_0.8/pmp_network_ACM_kmd_5.dat
```

```
HBox(children=(IntProgress(value=0, max=19), HTML(value='')))
```

```
Best ONMI: 0.497982 params: '-t 0.3'
```

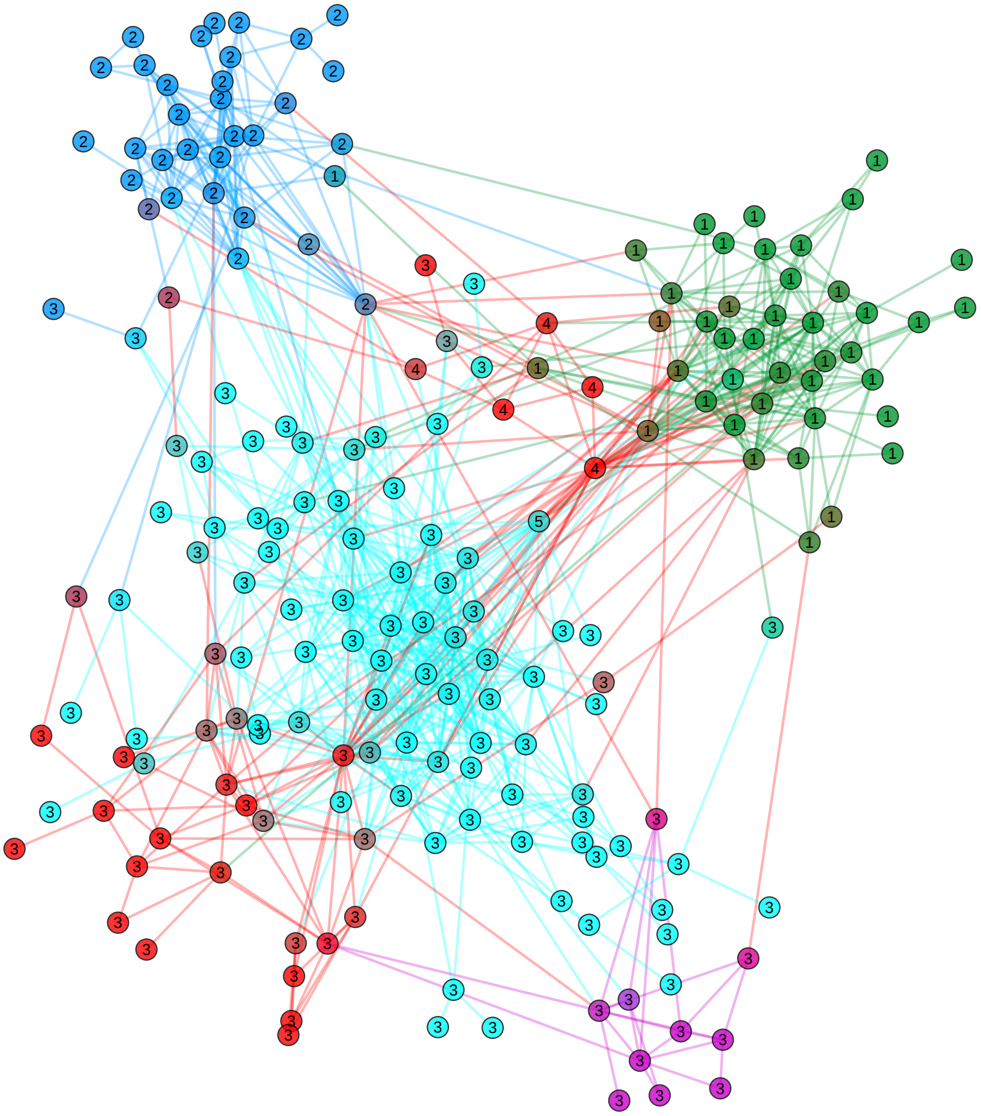

lp_kmd_acm_FARZ_n_200_m_5_k_5_beta_0.8

9.6 FARZ_n_200_m_5_k_5_beta_0.75

```
In [16]: params={}
         params["-t"] = np.arange(0.05, 1.0, 0.05)
```

```
all_results = lp_experiment(clustersNumber=5,
              algorithm = "kmd",
              distance = "acm",
              inputFile = "../datasets/FARZ_n_200_m_5_k_5_beta_0.75/network.dat",
              groundTruth = "../datasets/FARZ_n_200_m_5_k_5_beta_0.75/network.lgt",
              params = params,
              vertexNumerationShift=0,
              benchmarkFormat=True)
```

```
Output dir name: ../Results/lp_kmd_acm_FARZ_n_200_m_5_k_5_beta_0.75
Output file name:
../Results/lp_kmd_acm_FARZ_n_200_m_5_k_5_beta_0.75/pmp_network_ACM_kmd_5.dat
```

```
HBox(children=(IntProgress(value=0, max=19), HTML(value='')))
```

```
Best ONMI: 0.629307 params: '-t 0.35000000000000003'
```

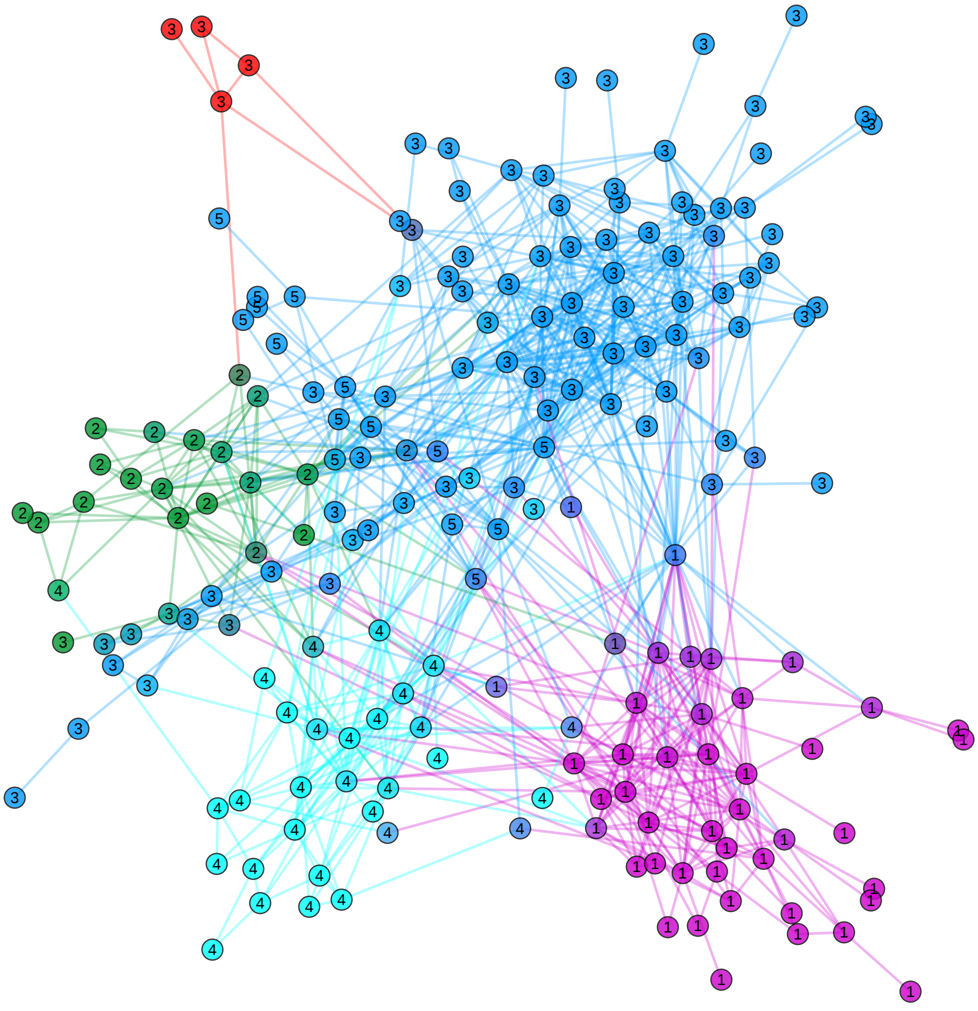

lp_kmd_acm_FARZ_n_200_m_5_k_5_beta_0.75

9.7 FARZ_n_200_m_5_k_5_beta_0.7

```
In [77]: params={}
         params["-t"] = np.arange(0.05, 1.0, 0.05)
         all_results = lp_experiment(clustersNumber=5,
                         algorithm = "kmd",
                         distance = "acm",
                         inputFile = "../datasets/FARZ_n_200_m_5_k_5_beta_0.7/network.dat",
                         groundTruth = "../datasets/FARZ_n_200_m_5_k_5_beta_0.7/network.lgt",
                         params = params,
                         vertexNumerationShift=0,
                         benchmarkFormat=True)
```

```
Output dir name: ../Results/lp_kmd_acm_FARZ_n_200_m_5_k_5_beta_0.7
Output file name:
../Results/lp_kmd_acm_FARZ_n_200_m_5_k_5_beta_0.7/pmp_network_ACM_kmd_5.dat
```

```
HBox(children=(IntProgress(value=0, max=19), HTML(value='')))
```

```
Best ONMI: 0.436827 params: '-t 0.3'
```

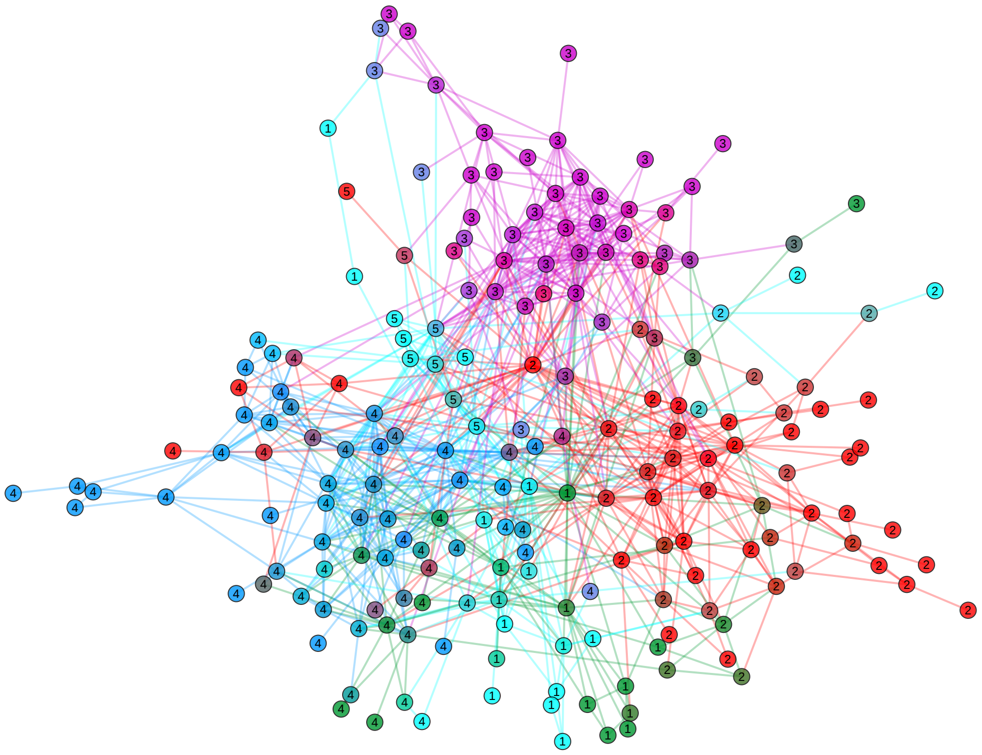

lp_kmd_acm_FARZ_n_200_m_5_k_5_beta_0.7

9.8 FARZ_n_200_m_5_k_5_beta_0.65

```
In [18]: params={}
         params["-t"] = np.arange(0.05, 1.0, 0.05)
         all_results = lp_experiment(clustersNumber=5,
```

```
                algorithm = "kmd",
                distance = "acm",
                inputFile = "../datasets/FARZ_n_200_m_5_k_5_beta_0.65/network.dat",
                groundTruth = "../datasets/FARZ_n_200_m_5_k_5_beta_0.65/network.lgt",
                params = params,
                vertexNumerationShift=0,
                benchmarkFormat=True)
```

```
Output dir name: ../Results/lp_kmd_acm_FARZ_n_200_m_5_k_5_beta_0.65
Output file name:
../Results/lp_kmd_acm_FARZ_n_200_m_5_k_5_beta_0.65/pmp_network_ACM_kmd_5.dat
```

```
HBox(children=(IntProgress(value=0, max=19), HTML(value='')))
```

```
Best ONMI: 0.52277 params: '-t 0.45'
```

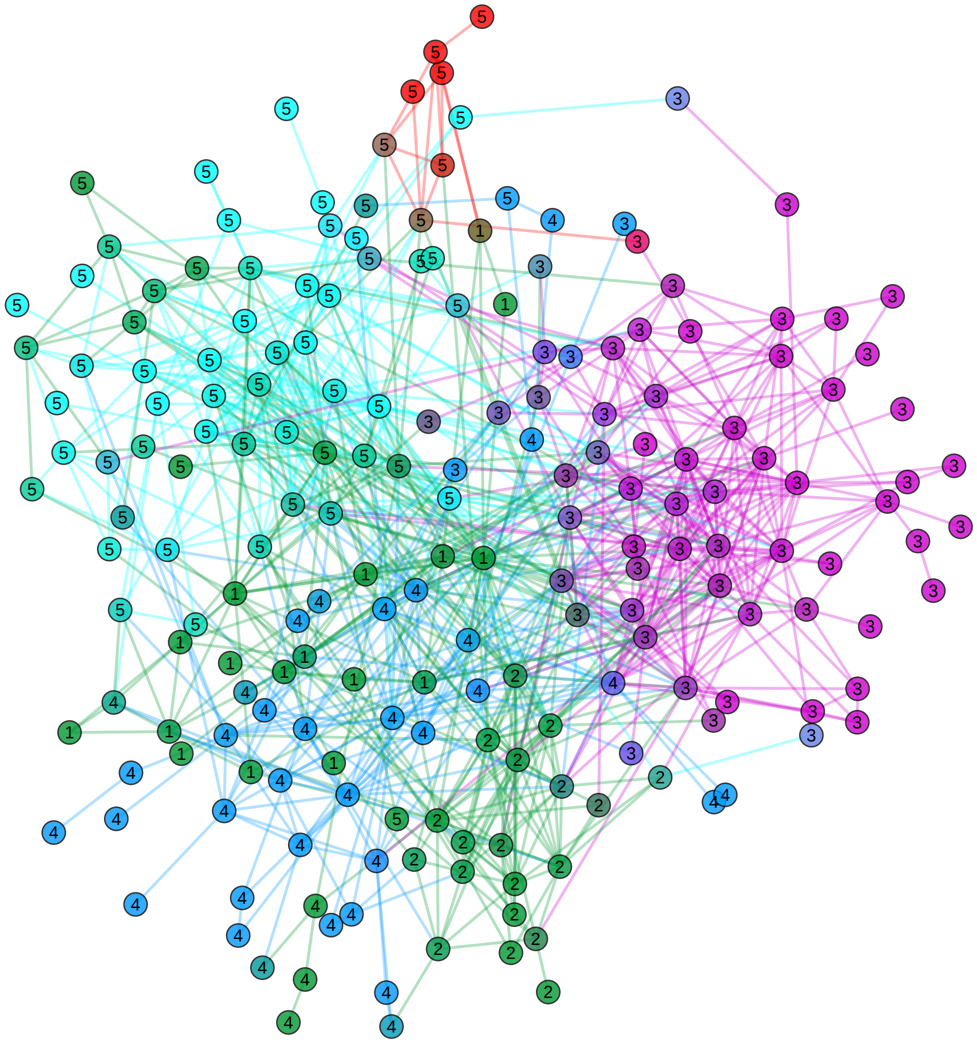

lp_kmd_acm_FARZ_n_200_m_5_k_5_beta_0.65

9.9 FARZ_n_200_m_5_k_5_beta_0.6

```
In [19]: params={}
         params["-t"] = np.arange(0.05, 1.0, 0.05)
         all_results = lp_experiment(clustersNumber=5,
                         algorithm = "kmd",
                         distance = "acm",
                         inputFile = "../datasets/FARZ_n_200_m_5_k_5_beta_0.6/network.dat",
                         groundTruth = "../datasets/FARZ_n_200_m_5_k_5_beta_0.6/network.lgt",
                         params = params,
                         vertexNumerationShift=0,
                         benchmarkFormat=True)
```

```
Output dir name: ../Results/lp_kmd_acm_FARZ_n_200_m_5_k_5_beta_0.6
Output file name:
../Results/lp_kmd_acm_FARZ_n_200_m_5_k_5_beta_0.6/pmp_network_ACM_kmd_5.dat
```

```
HBox(children=(IntProgress(value=0, max=19), HTML(value='')))
```

```
Best ONMI: 0.342269 params: '-t 0.55'
```

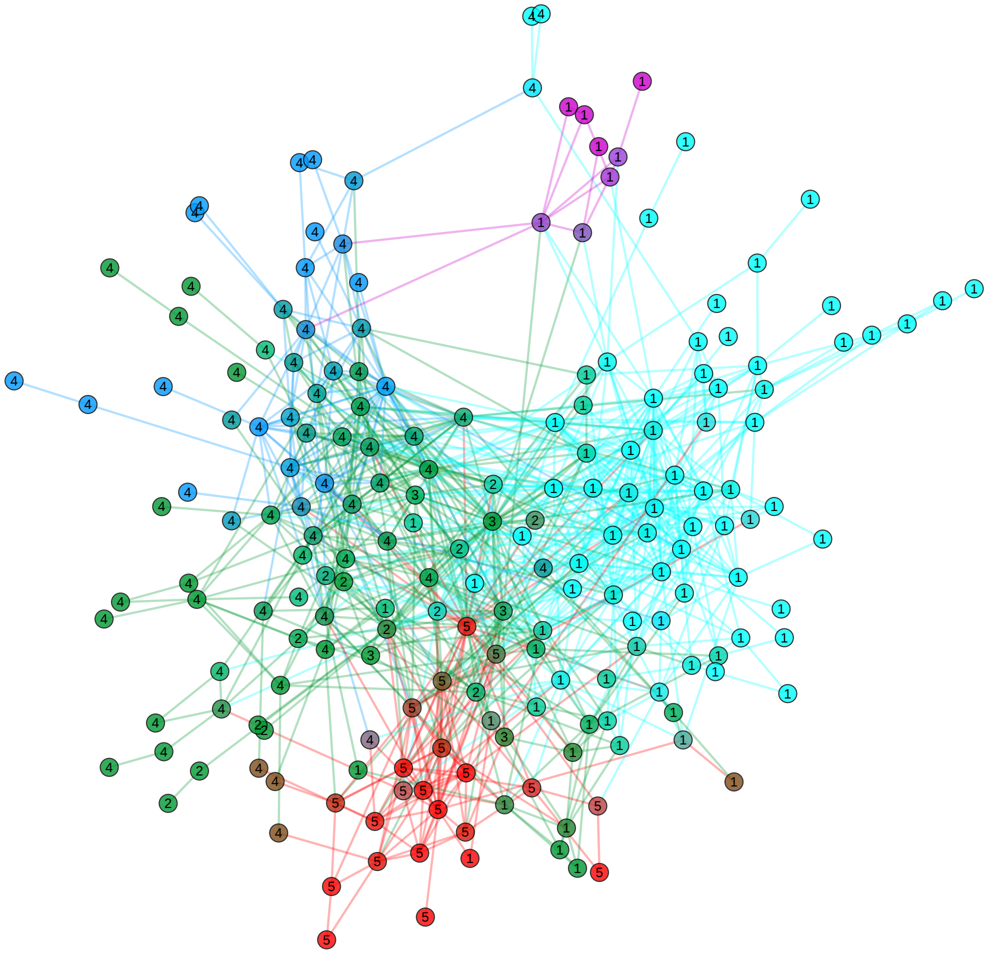

lp_kmd_acm_FARZ_n_200_m_5_k_5_beta_0.6

9.10 FARZ_n_200_m_5_k_5_beta_0.55

```
In [ ]: params={}
        params["-t"] = np.arange(0.05, 1.0, 0.05)
        all_results = lp_experiment(clustersNumber=3,
                      algorithm = "fkmd",
                      distance = "iacm",
                      inputFile = "../datasets/FARZ_n_200_m_5_k_5_beta_0.55/network.dat",
                      groundTruth = "../datasets/FARZ_n_200_m_5_k_5_beta_0.55/network.lgt",
                      params = params,
                      vertexNumerationShift=0,
                      benchmarkFormat=True,
                                  verbose=True)
```

```
Output dir name: ../Results/lp_fkmd_iacm_FARZ_n_200_m_5_k_5_beta_0.55
Output file name:
../Results/lp_fkmd_iacm_FARZ_n_200_m_5_k_5_beta_0.55/pmp_network_IACM_fkmd_3.dat
```

```
HBox(children=(IntProgress(value=0, max=19), HTML(value='')))
```

```
java -jar ../lpam/target/Clustering-1.2-jar-with-dependencies.jar -b -a fkmd -o
../Results/lp_fkmd_iacm_FARZ_n_200_m_5_k_5_beta_0.55 -i
../datasets/FARZ_n_200_m_5_k_5_beta_0.55/network.dat -k 3 -d iacm -t 0.05
```

```
In [63]: params={}
         params["-t"] = np.arange(0.05, 1.0, 0.05)
         all_results = lp_experiment(clustersNumber=4,
                       algorithm = "kmd",
                       distance = "acm",
                       inputFile = "../datasets/FARZ_n_200_m_5_k_5_beta_0.55/network.dat",
                       groundTruth = "../datasets/FARZ_n_200_m_5_k_5_beta_0.55/network.lgt",
                       params = params,
                       vertexNumerationShift=0,
                       benchmarkFormat=True)
```

```
Output dir name: ../Results/lp_kmd_acm_FARZ_n_200_m_5_k_5_beta_0.55
Output file name:
../Results/lp_kmd_acm_FARZ_n_200_m_5_k_5_beta_0.55/pmp_network_ACM_kmd_4.dat
```

```
HBox(children=(IntProgress(value=0, max=19), HTML(value='')))
```

```
Best ONMI: 0.31533 params: '-t 0.6000000000000001'
```

```
In [20]: params={}
         params["-t"] = np.arange(0.05, 1.0, 0.05)
         all_results = lp_experiment(clustersNumber=5,
                       algorithm = "kmd",
                       distance = "acm",
                       inputFile = "../datasets/FARZ_n_200_m_5_k_5_beta_0.55/network.dat",
                       groundTruth = "../datasets/FARZ_n_200_m_5_k_5_beta_0.55/network.lgt",
                       params = params,
                       vertexNumerationShift=0,
                       benchmarkFormat=True)
```

```
Output dir name: ../Results/lp_kmd_acm_FARZ_n_200_m_5_k_5_beta_0.55
Output file name:
../Results/lp_kmd_acm_FARZ_n_200_m_5_k_5_beta_0.55/pmp_network_ACM_kmd_5.dat
```

HBox(children=(IntProgress(value=0, max=19), HTML(value='')))

Best ONMI: 0.295493 params: '-t 0.5'

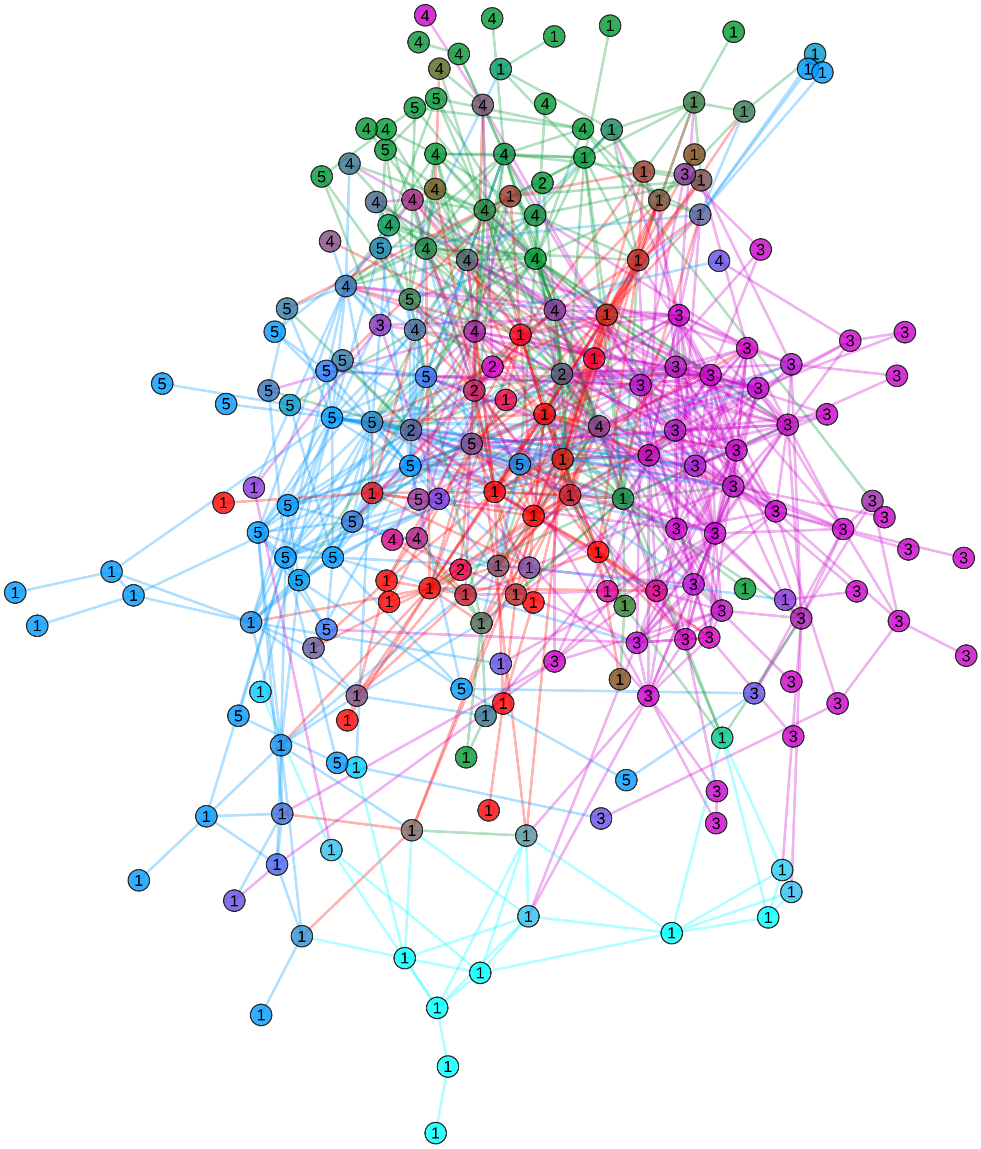

lp_kmd_acm_FARZ_n_200_m_5_k_5_beta_0.55

```
In [59]: params={}
         params["-t"] = np.arange(0.05, 1.0, 0.05)
         all_results = lp_experiment(clustersNumber=6,
                       algorithm = "kmd",
                       distance = "acm",
                       inputFile = "../datasets/FARZ_n_200_m_5_k_5_beta_0.55/network.dat",
                       groundTruth = "../datasets/FARZ_n_200_m_5_k_5_beta_0.55/network.lgt",
                       params = params,
```

```
                   vertexNumerationShift=0,
                   benchmarkFormat=True)
```

Output dir name: ../Results/lp_kmd_acm_FARZ_n_200_m_5_k_5_beta_0.55
Output file name:
../Results/lp_kmd_acm_FARZ_n_200_m_5_k_5_beta_0.55/pmp_network_ACM_kmd_6.dat

HBox(children=(IntProgress(value=0, max=19), HTML(value='')))

Best ONMI: 0.295061 params: '-t 0.7000000000000001'

```
In [60]: params={}
         params["-t"] = np.arange(0.05, 1.0, 0.05)
         all_results = lp_experiment(clustersNumber=7,
                        algorithm = "kmd",
                        distance = "acm",
                        inputFile = "../datasets/FARZ_n_200_m_5_k_5_beta_0.55/network.dat",
                        groundTruth = "../datasets/FARZ_n_200_m_5_k_5_beta_0.55/network.lgt",
                        params = params,
                        vertexNumerationShift=0,
                        benchmarkFormat=True)
```

Output dir name: ../Results/lp_kmd_acm_FARZ_n_200_m_5_k_5_beta_0.55
Output file name:
../Results/lp_kmd_acm_FARZ_n_200_m_5_k_5_beta_0.55/pmp_network_ACM_kmd_7.dat

HBox(children=(IntProgress(value=0, max=19), HTML(value='')))

Best ONMI: 0.209124 params: '-t 0.55'

```
In [61]: params={}
         params["-t"] = np.arange(0.05, 1.0, 0.05)
         all_results = lp_experiment(clustersNumber=8,
                        algorithm = "kmd",
                        distance = "acm",
                        inputFile = "../datasets/FARZ_n_200_m_5_k_5_beta_0.55/network.dat",
                        groundTruth = "../datasets/FARZ_n_200_m_5_k_5_beta_0.55/network.lgt",
                        params = params,
                        vertexNumerationShift=0,
                        benchmarkFormat=True)
```

Output dir name: ../Results/lp_kmd_acm_FARZ_n_200_m_5_k_5_beta_0.55
Output file name:
../Results/lp_kmd_acm_FARZ_n_200_m_5_k_5_beta_0.55/pmp_network_ACM_kmd_8.dat

HBox(children=(IntProgress(value=0, max=19), HTML(value='')))

Best ONMI: 0.21049 params: '-t 0.6000000000000001'

```
In [62]: params={}
         params["-t"] = np.arange(0.05, 1.0, 0.05)
         all_results = lp_experiment(clustersNumber=9,
                        algorithm = "kmd",
                        distance = "acm",
                        inputFile = "../datasets/FARZ_n_200_m_5_k_5_beta_0.55/network.dat",
                        groundTruth = "../datasets/FARZ_n_200_m_5_k_5_beta_0.55/network.lgt",
                        params = params,
                        vertexNumerationShift=0,
                        benchmarkFormat=True)
```

```
Output dir name: ../Results/lp_kmd_acm_FARZ_n_200_m_5_k_5_beta_0.55
Output file name:
../Results/lp_kmd_acm_FARZ_n_200_m_5_k_5_beta_0.55/pmp_network_ACM_kmd_9.dat
```

```
HBox(children=(IntProgress(value=0, max=19), HTML(value='')))
```

```
Best ONMI: 0.22652 params: '-t 0.55'
```

```
In [64]: params={}
         params["-t"] = np.arange(0.05, 1.0, 0.05)
         all_results = lp_experiment(clustersNumber=10,
                        algorithm = "kmd",
                        distance = "acm",
                        inputFile = "../datasets/FARZ_n_200_m_5_k_5_beta_0.55/network.dat",
                        groundTruth = "../datasets/FARZ_n_200_m_5_k_5_beta_0.55/network.lgt",
                        params = params,
                        vertexNumerationShift=0,
                        benchmarkFormat=True)
```

```
Output dir name: ../Results/lp_kmd_acm_FARZ_n_200_m_5_k_5_beta_0.55
Output file name:
../Results/lp_kmd_acm_FARZ_n_200_m_5_k_5_beta_0.55/pmp_network_ACM_kmd_10.dat
```

```
HBox(children=(IntProgress(value=0, max=19), HTML(value='')))
```

```
Best ONMI: 0.196806 params: '-t 0.55'
```

9.11 FARZ_n_200_m_5_k_5_beta_0.5

```
In [81]: params={}
         params["-t"] = np.arange(0.05, 1.0, 0.05)
         all_results = lp_experiment(clustersNumber=4,
                        algorithm = "kmd",
                        distance = "acm",
                        inputFile = "../datasets/FARZ_n_200_m_5_k_5_beta_0.5/network.dat",
                        groundTruth = "../datasets/FARZ_n_200_m_5_k_5_beta_0.5/network.lgt",
                        params = params,
                        vertexNumerationShift=0,
                        benchmarkFormat=True)
```

```
Output dir name: ../Results/lp_kmd_acm_FARZ_n_200_m_5_k_5_beta_0.5
Output file name:
../Results/lp_kmd_acm_FARZ_n_200_m_5_k_5_beta_0.5/pmp_network_ACM_kmd_4.dat
```

```
HBox(children=(IntProgress(value=0, max=19), HTML(value='')))
```

```
Best ONMI: 0.0900377 params: '-t 0.35000000000000003'
```

```
In [21]: params={}
         params["-t"] = np.arange(0.05, 1.0, 0.05)
         all_results = lp_experiment(clustersNumber=5,
                         algorithm = "kmd",
                         distance = "acm",
                         inputFile = "../datasets/FARZ_n_200_m_5_k_5_beta_0.5/network.dat",
                         groundTruth = "../datasets/FARZ_n_200_m_5_k_5_beta_0.5/network.lgt",
                         params = params,
                         vertexNumerationShift=0,
                         benchmarkFormat=True)
```

```
Output dir name: ../Results/lp_kmd_acm_FARZ_n_200_m_5_k_5_beta_0.5
Output file name:
../Results/lp_kmd_acm_FARZ_n_200_m_5_k_5_beta_0.5/pmp_network_ACM_kmd_5.dat
```

```
HBox(children=(IntProgress(value=0, max=19), HTML(value='')))
```

```
Best ONMI: 0.10506 params: '-t 0.15000000000000002'
```

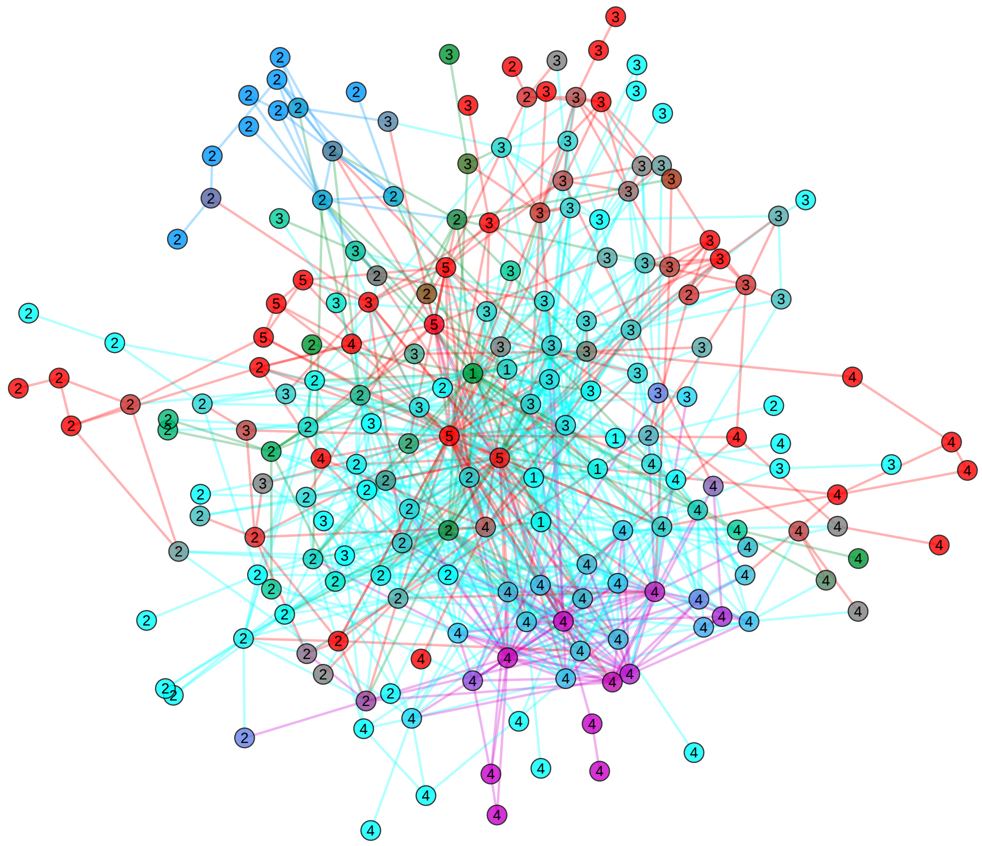

lp_kmd_acm_FARZ_n_200_m_5_k_5_beta_0.5

9.12 FARZ_n_1000_m_7_k_20_beta_0.9

```
In [ ]: params={}
        params["-t"] = np.arange(0.05, 1.0, 0.05)
        all_results = lp_experiment(clustersNumber=20,
                      algorithm = "kmd",
                      distance = "acm",
                      inputFile = "../datasets/FARZ_n_1000_m_7_k_20_beta_0.9/network.dat",
                      groundTruth = "../datasets/FARZ_n_1000_m_7_k_20_beta_0.9/network.lgt",
                      params = params,
                      vertexNumerationShift=0,
                      benchmarkFormat=True,
                      verbose=False)
```

```
Output dir name: ../Results/lp_kmd_acm_FARZ_n_1000_m_7_k_20_beta_0.9
Output file name:
../Results/lp_kmd_acm_FARZ_n_1000_m_7_k_20_beta_0.9/pmp_network_ACM_kmd_20.dat
```

```
HBox(children=(IntProgress(value=0, max=19), HTML(value='')))
```

10 CKB and CKB-t

10.1 CKB_n_200

```
In [38]: !ls ../datasets/CKB_n_200
```

```
dl_coms.txt  dl_edges_tabs.txt  dl_edges.txt
```

```
In [27]: !wc -l ../datasets/CKB_n_200/dl_coms.txt
```

```
78 ../datasets/CKB_n_200/dl_coms.txt
```

```
In [46]: params={}
         params["-t"] = np.arange(0.05, 1.0, 0.05)
         all_results = lp_experiment(clustersNumber=78,
                       algorithm = "kmd",
                       distance = "acm",
                       inputFile = "../datasets/CKB_n_200/dl_edges_tabs.txt",
                       groundTruth = "../datasets/CKB_n_200/dl_coms.txt",
                       params = params,
                       vertexNumerationShift=0,
                       benchmarkFormat=True)
```

```
Output dir name: ../Results/lp_kmd_acm_CKB_n_200
Output file name: ../Results/lp_kmd_acm_CKB_n_200/pmp_dl_edges_tabs_ACM_kmd_78.dat
```

```
HBox(children=(IntProgress(value=0, max=19), HTML(value='')))
```

```
Best ONMI: 0.0973312 params: '-t 0.15000000000000002'
```

10.2 CKB-t_n_200_alfa_0.1_gamma_0.5

```
In [50]: !wc -l ../datasets/CKB-t_n_200_alfa_0.1_gamma_0.5/dl_coms.txt
```

```
96 ../datasets/CKB-t_n_200_alfa_0.1_gamma_0.5/dl_coms.txt
```

```
In [79]: params={}
         params["-t"] = np.arange(0.05, 1.0, 0.05)
         all_results = lp_experiment(clustersNumber=120,
                       algorithm = "kmd",
                       distance = "acm",
                       inputFile = "../datasets/CKB-
         t_n_200_alfa_0.1_gamma_0.5/dl_edges_tabs.txt",
                       groundTruth = "../datasets/CKB-t_n_200_alfa_0.1_gamma_0.5/dl_coms.txt",
                       params = params,
                       vertexNumerationShift=0,
                       benchmarkFormat=True,
                       verbose=False)
```

```
Output dir name: ../Results/lp_kmd_acm_CKB-t_n_200_alfa_0.1_gamma_0.5
Output file name: ../Results/lp_kmd_acm_CKB-
t_n_200_alfa_0.1_gamma_0.5/pmp_dl_edges_tabs_ACM_kmd_120.dat
```

```
HBox(children=(IntProgress(value=0, max=19), HTML(value='')))
```

```
Best ONMI: 0.0877926 params: '-t 0.05'
```

Best ONMI: 0.056446 params: ′-t 0.05′ clustersNumber=80 Best ONMI: 0.056553 params: ′-t 0.05′ clustersNumber=96 Best ONMI: 0.0877926 params: ′-t 0.05′ clustersNumber=120 Best ONMI: 0.0819077 params: ′-t 0.05′ clustersNumber=130

10.3 CKB-t_n_1000_alfa_0.1_gamma_0.5_max_memb_20_max_com_size_200

```
In [82]: !wc -l ../datasets/CKB-
         t_n_1000_alfa_0.1_gamma_0.5_max_memb_20_max_com_size_200/dl_coms.txt
```

```
175 ../datasets/CKB-
t_n_1000_alfa_0.1_gamma_0.5_max_memb_20_max_com_size_200/dl_coms.txt
```

```
In [88]: params={}
         params["-t"] = np.arange(0.05, 1.0, 0.05)
         all_results = lp_experiment(clustersNumber=10,
                       algorithm = "fkmd",
                       distance = "iacm",
                       inputFile = "../datasets/CKB-
         t_n_1000_alfa_0.1_gamma_0.5_max_memb_20_max_com_size_200/dl_edges_tabs.txt",
                       groundTruth = "../datasets/CKB-
         t_n_1000_alfa_0.1_gamma_0.5_max_memb_20_max_com_size_200/dl_coms.txt",
                       params = params,
                       vertexNumerationShift=0,
                       benchmarkFormat=True,
                                    verbose=False)
```

```
Output dir name: ../Results/lp_fkmd_iacm_CKB-
t_n_1000_alfa_0.1_gamma_0.5_max_memb_20_max_com_size_200
Output file name: ../Results/lp_fkmd_iacm_CKB-t_n_1000_alfa_0.1_gamma_0.5_max_memb_20_
max_com_size_200/pmp_dl_edges_tabs_IACM_fkmd_10.dat
```

```
HBox(children=(IntProgress(value=0, max=19), HTML(value='')))
```

```
Best ONMI: 0.0104175 params: '-t 0.05'
```

```
In [ ]:
```

ONMI: 0.0104175 params: ′-t 0.05′ clustersNumber=10 Best ONMI: 0.0104175 params: ′-t 0.05′ clustersNumber=100 Best ONMI: 0.0104175 params: ′-t 0.05′ clustersNumber=175

```
In [ ]:
```

```
In [ ]:
```
