# Supplementary material for: Overlapping community detection in networks based on link partitioning and partitioning around medoids
Source: PLoS One. 2021 Aug 25;16(8):e0255717. doi: 10.1371/journal.pone.0255717 (PMC8386890; doi:10.1371/journal.pone.0255717)
Supplement: S3 Appendix — The computational results for the exact version of the LPAM method with commute distance. (PDF) [file pone.0255717.s003.pdf]

LPAM-Exact-Commute-Distance

July 18, 2019

Link Partitioning Around Medoids - Exact
Distance function: Commute Distance

```
In [8]: import numpy as np
        import random
        random.seed = 108
        from tqdm import tqdm_notebook as tqdm
        import seaborn as sns
        import matplotlib.pyplot as plt
        from mpl_toolkits import mplot3d
        import pandas as pd
        %matplotlib inline
```

```
In [3]: !java -jar ../lpam/out/artifacts/Clustering_jar/Clustering.jar
```

```
Missing required options: i, k
usage: PMPClustering <-i input_file> <-k number_of_clusters> [-ftbcd]
 -a,--algorithm <arg>      Specifies algorithm that will be used to find
                           disjoint edge clusters. Possible values:
                           pmp (p-median exact algorithm).
                           kmd (k-medoids heuristic)
                           kmn (k-means heuristic).
                           If this option is omitted, the P-Median
                           algorithm will be used.
 -b,--benchmark            use benchmark format (.dat file)
 -d,--distance <arg>       the type of function to measure the distance
                           between nodes.
                           Possible values:
                           sp (shortest path)
                           gd (Generalize Degree)
                           cm (Commute Distance)
                           acm (Amplified Commute Distance).
                           If this option is omitted, the amplified
                           commute distance function will be used.
 -f,--force                The previous founded solution by lp_solver will
                           not be used. lp_solver will be started
                           forcibly.
                           The flag effects only the PMP exact edge
                           clustering algorithm.
                           By default algorithm will try to get previous
                           founded solution
 -gt,--groundTruth <arg>   load info about ground truth community file
 -i,--input <arg>          path to the input file in GML format
 -k <arg>                  Number of clusters to detect (int)
 -l,--linegraph            produce line graph as output
 -o,--outputDir <arg>      the name of the output file. If option was
                           omitted, the name of the output file
```

```
                             will be composed automatically as
                             "{suffix}_{distanceName}_{clusterNumber}_out.ge
                             xf"
 -t,--threshold <arg>        Vertex i belongs to cluster C, if node i has
                             fraction of edges in cluster C greater then
                             this value
                             Default value is 0.
```

```
In [4]: def lp_experiment(clustersNumber, algorithm, distance, inputFile, groundTruth, params =
        {}, vertexNumerationShift=0, benchmarkFormat=False, verbose=False, lineGraph=False):
            lineGraphParam = ""
            if (lineGraph == True):
                lineGraphParam = " -l"
            datasetName = inputFile.split('/')[-2]
            sufix = inputFile.split('/')[-1].split('.')[0]
            outputDir = "../Results/lp_{}_{}_{}".format(algorithm, distance, datasetName)
            outputFile = outputDir +'/' +"pmp_{3}_{2}_{0}_{1}.dat".format(algorithm,
        clustersNumber, distance.upper(), sufix)
            print("Output dir name: {}".format(outputDir) )
            print("Output file name: {}".format(outputFile) )
            all_results = {}
            bestParam = "not found"
            nmi_best = 0;
            param_list = list(generate_params(params))
            tmp = None
            for param in tqdm(param_list):
                if benchmarkFormat:
                    tmp=!java -jar ../lpam/out/artifacts/Clustering_jar/Clustering.jar -b -a
        {algorithm} -o {outputDir} -i {inputFile} -k {clustersNumber} -d {distance} -gt
        {groundTruth} {param}
                    tmpFile = outputFile
                else:
                    tmp=!java -jar ../lpam/out/artifacts/Clustering_jar/Clustering.jar -a
        {algorithm} -o {outputDir} -i {inputFile} -k {clustersNumber} -d {distance} -gt
        {groundTruth} {param}
                    lines=[]
                    with open(outputFile) as f:
                        lines = f.readlines()
                    tmpFile="tmpFile.dat"
                    with open(tmpFile, 'w') as the_file:
                        for line in lines:
                            the_file.write(" ".join([str(int(a)+vertexNumerationShift) for a in
        line.split()]) + "\n")

                output=!../Overlapping-NMI/onmi {groundTruth} {tmpFile}
                nmi=float(output[0].split()[1])
                all_results[param] = nmi
                if nmi >  nmi_best:
                    bestParam = param
                    nmi_best = nmi
            #restorign solution for the best parameters
            if benchmarkFormat:
                tmp=!java -jar ../lpam/out/artifacts/Clustering_jar/Clustering.jar -b -a
        {algorithm} -o {outputDir} -i {inputFile} -gt {groundTruth} -k {clustersNumber} -d
        {distance} -gt {groundTruth} {bestParam} {lineGraphParam}
                tmpFile = outputFile
            else:
                tmp=!java -jar ../lpam/out/artifacts/Clustering_jar/Clustering.jar -a
        {algorithm} -o {outputDir} -i {inputFile} -gt {groundTruth} -k {clustersNumber} -d
        {distance} -gt {groundTruth} {bestParam} {lineGraphParam}
                lines=[]
                with open(outputFile) as f:
                    lines = f.readlines()
                tmpFile="tmpFile.dat"
                with open(tmpFile, 'w') as the_file:
                    for line in lines:
```

```
                    the_file.write(" ".join([str(int(a)+vertexNumerationShift) for a in
        line.split()]) + "\n")

            if verbose:
                print(tmp)
            print("Best ONMI: {} params: '{}'".format(nmi_best, bestParam) )
            return all_results
```

```
In [5]: def generate_params(params):
            keys = list(params.keys())
            if len(keys) == 1:
                for value in params[keys[0]]:
                    yield ( keys[0] + " " + str(value) )
            if len( keys ) > 1:
                for value in params[keys[0]]:
                    for remain_params in generate_params({k:params[k] for k in keys[1:]}):
                        yield ( keys[0] + " " + str(value) + " " + remain_params )
```

```
In [6]: def plot_all_params(algorithm, dataset, all_results ):
            xdata=[]
            ydata=[]
            df = pd.DataFrame()
            for param, nmi in all_results.items():
                splited = param.split()
                xdata.append(float(splited[1]))
                ydata.append(nmi)
                df = df.append({'x': float(splited[1]), 'y': nmi}, ignore_index=True)

            plt.plot(xdata, ydata, 'C3', zorder=1, lw=3)
            # ax = plt.axes(projection='3d')
            plt.scatter(xdata, ydata,s=70,zorder=2)
            plt.xlabel('threshold')
            plt.ylabel('nmi value');
            plt.title('Algorithm: {}\nDataset: {}'.format(algorithm, dataset));
            plt.show()
```

1 School friendship network

```
In [9]: params={}
        params["-t"] = np.arange(0.05, 1.0, 0.05)
```

```
In [11]: all_results = lp_experiment(clustersNumber=7,
                         algorithm = "pmp",
                         distance = "cm",
                         inputFile =  "../datasets/school_friendship/school-2.gml",
                         groundTruth = "../datasets/school_friendship/truth-school.dat",
                         params = params,
                         vertexNumerationShift=-1,
                         benchmarkFormat=False, lineGraph=True)
```

```
Output dir name: ../Results/lp_pmp_cm_school_friendship
Output file name: ../Results/lp_pmp_cm_school_friendship/pmp_school-2_CM_pmp_7.dat
```

```
HBox(children=(IntProgress(value=0, max=19), HTML(value='')))
```

```
Best ONMI: 0.675358 params: '-t 0.35000000000000003'
```

```
In [13]: plot_all_params(algorithm = "LPAM-Exact + Commute Distance", dataset = "School
         Friendship", all_results = all_results)
```

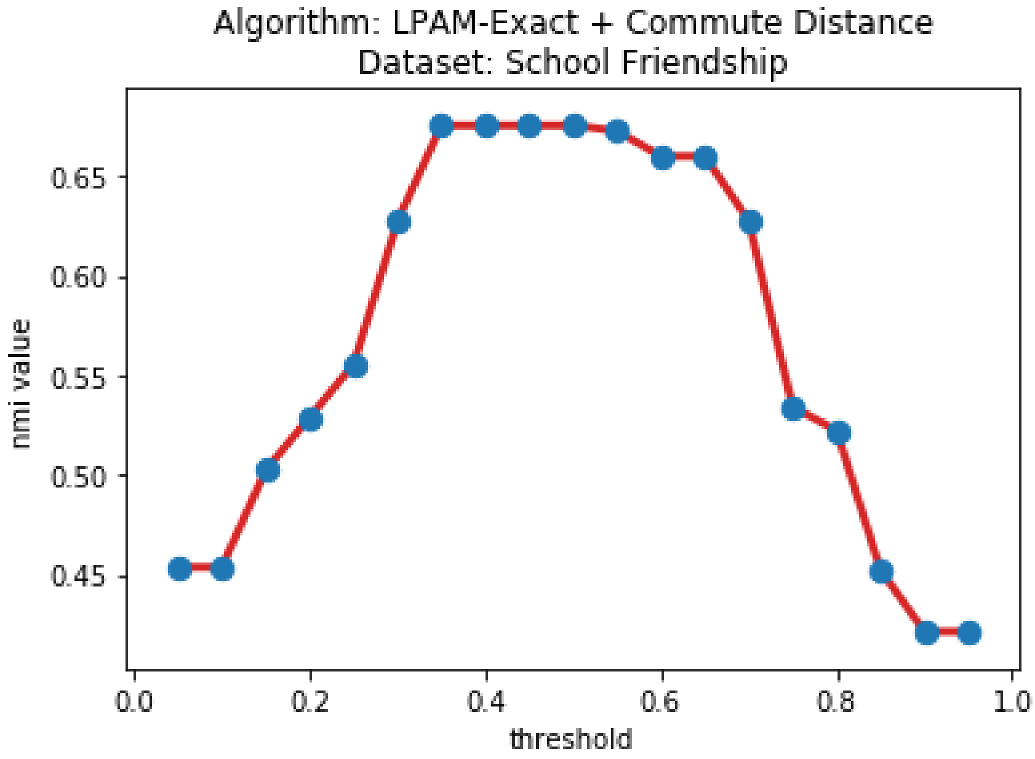

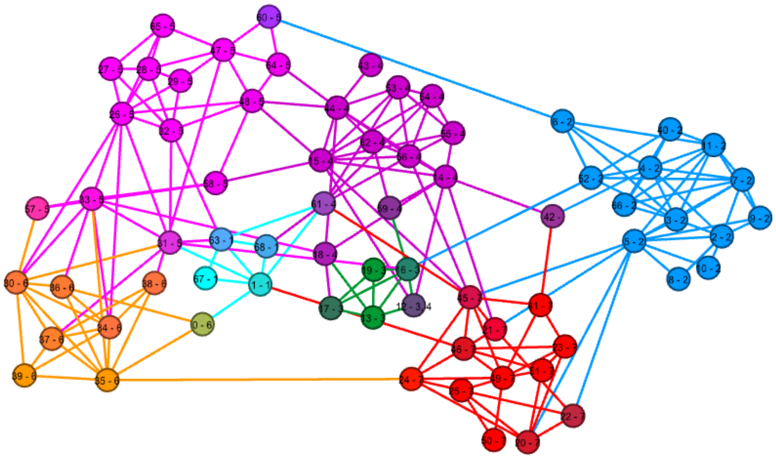

School friendship network. Number of Clusters: 7

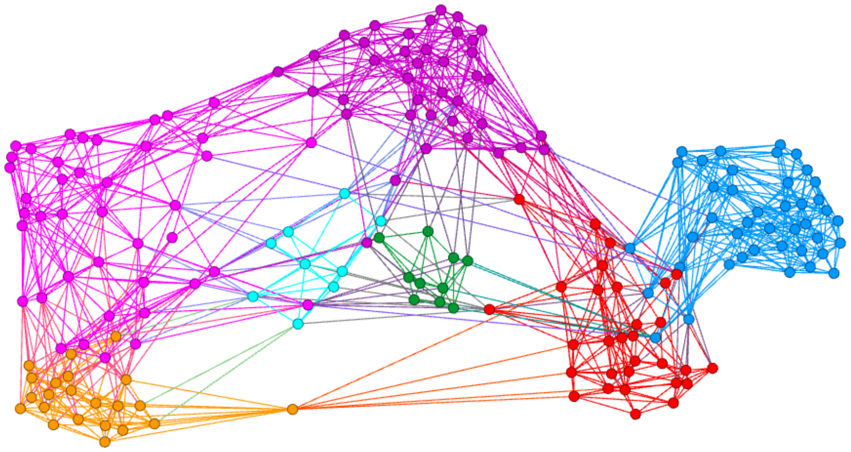

School friendship network - line graph

2 Karate Club

```
In [20]: params={}
         params["-t"] = np.arange(0.05, 1.0, 0.05)
         all_results = lp_experiment(clustersNumber=2,
                         algorithm = "pmp",
                         distance = "cm",
                         inputFile =  "../datasets/karate/karate.gml",
                         groundTruth = "../datasets/karate/truth_karate.dat",
                         params = params,
                         vertexNumerationShift=0,
                         benchmarkFormat=False, verbose=False, lineGraph=True)
```

```
Output dir name: ../Results/lp_pmp_cm_karate
Output file name: ../Results/lp_pmp_cm_karate/pmp_karate_CM_pmp_2.dat
```

```
HBox(children=(IntProgress(value=0, max=19), HTML(value='')))
```

```
Best ONMI: 0.91796 params: '-t 0.45'
```

```
In [37]: plot_all_params(algorithm = "LPAM-Exact+Commute-Distance", dataset = "Karate Club ",
         all_results = all_results)
```

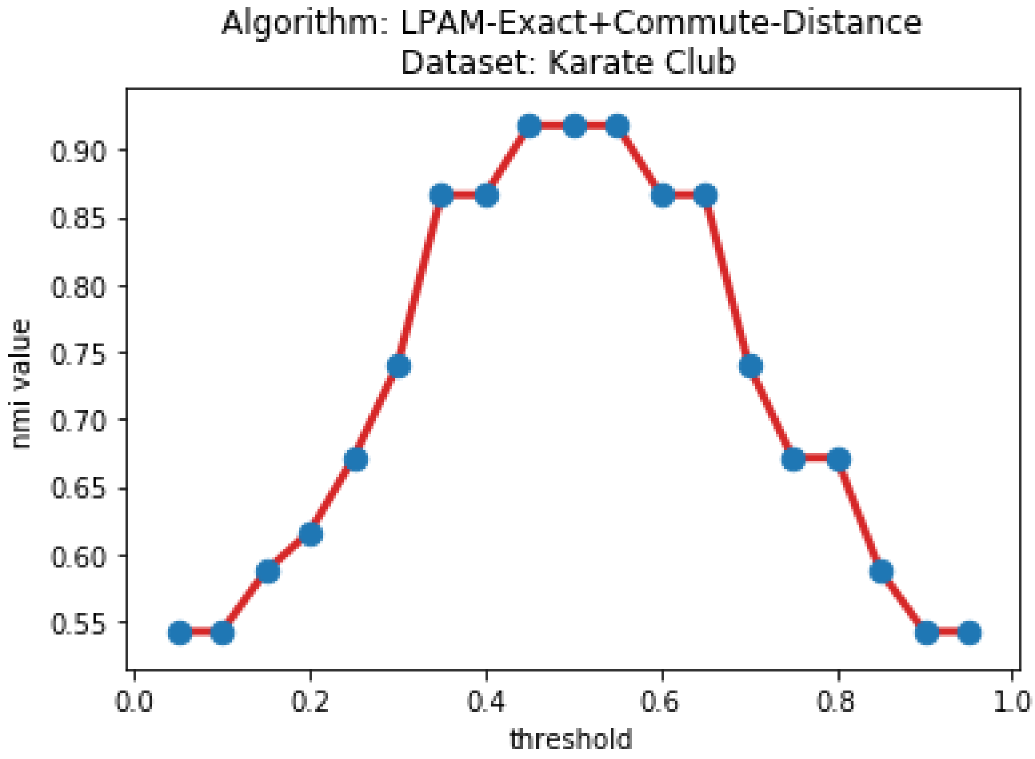
Algorithm: LPAM-Exact+Commute-Distance
Dataset: Karate Club
nmi value
0.90
0.85
0.80
0.75
0.70
0.65
0.60
0.55
0.0
0.2
0.4
0.6
0.8
1.0
threshold

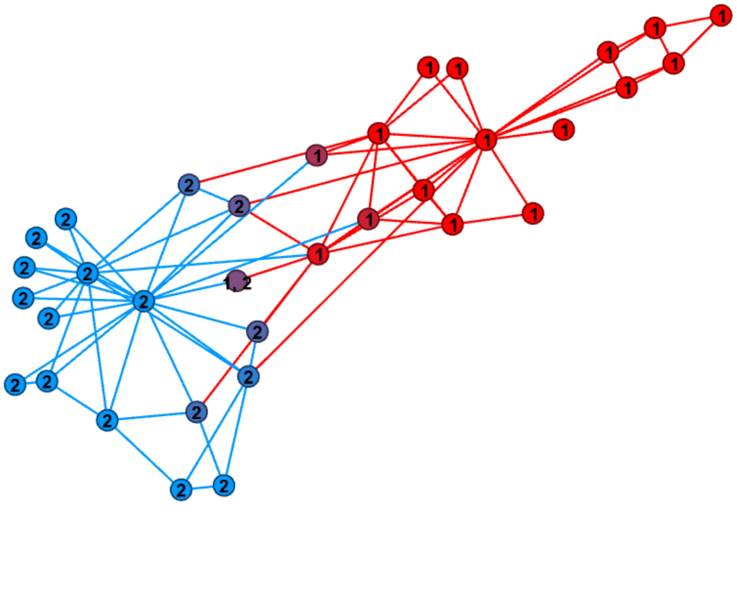

Karate club

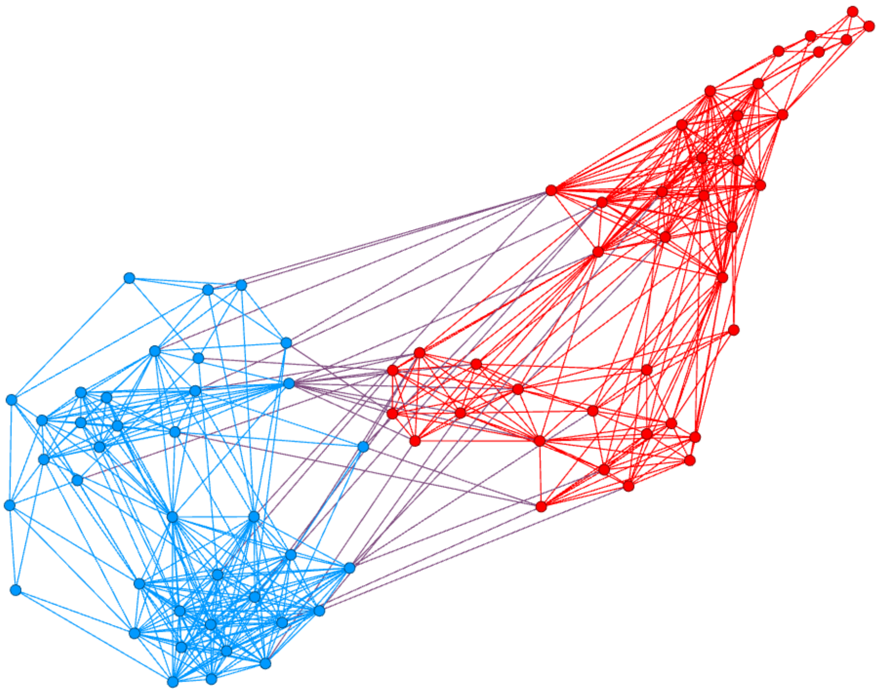

Karate club - line graph

3 American Football League c = 12

```
In [38]: params={}
         params["-t"] = np.arange(0.05, 1.0, 0.05)
         all_results = lp_experiment(clustersNumber=12,
                         algorithm = "kmd",
                         distance = "cm",
                         inputFile =  "../datasets/football/footballTSEinput.gml",
                         groundTruth = "../datasets/football/truth_footballTSEinput.dat",
                         params = params,
                         vertexNumerationShift=-1,
                         benchmarkFormat=False)
```

```
Output dir name: ../Results/lp_kmd_cm_football
Output file name: ../Results/lp_kmd_cm_football/pmp_footballTSEinput_CM_kmd_12.dat
```

```
HBox(children=(IntProgress(value=0, max=19), HTML(value='')))
```

```
Best ONMI: 0.71194 params: '-t 0.5'
```

```
In [39]: plot_all_params(algorithm = "LPAM-Exact + Commute Distance", dataset = "American
         Foolball League", all_results = all_results)
```

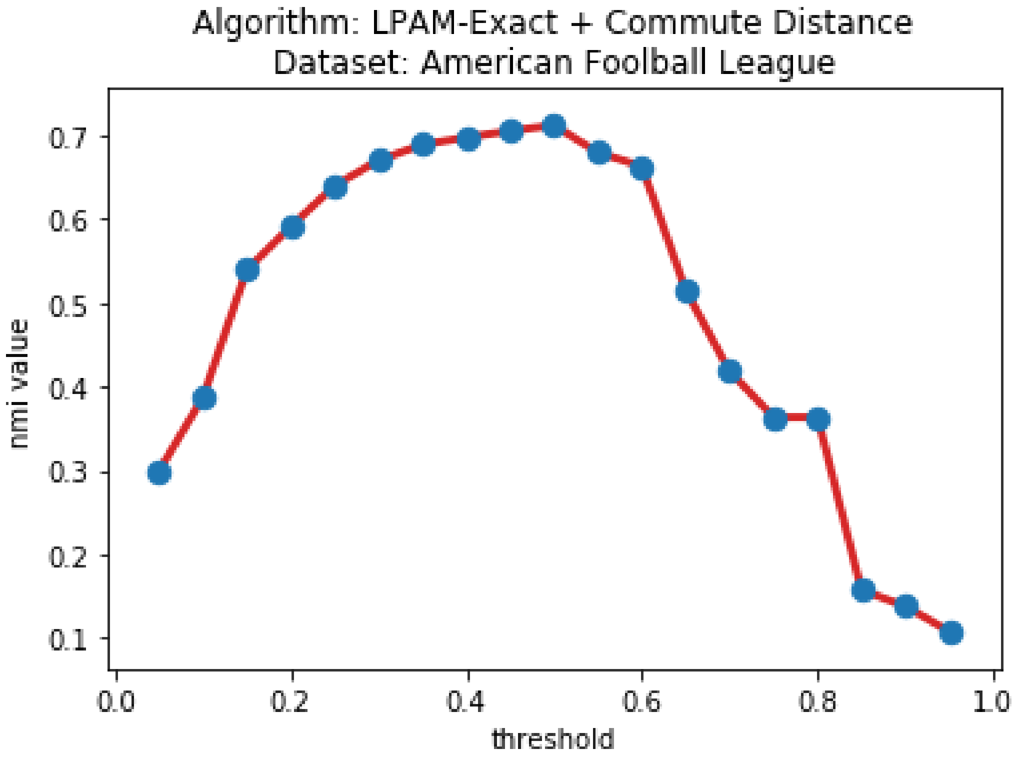

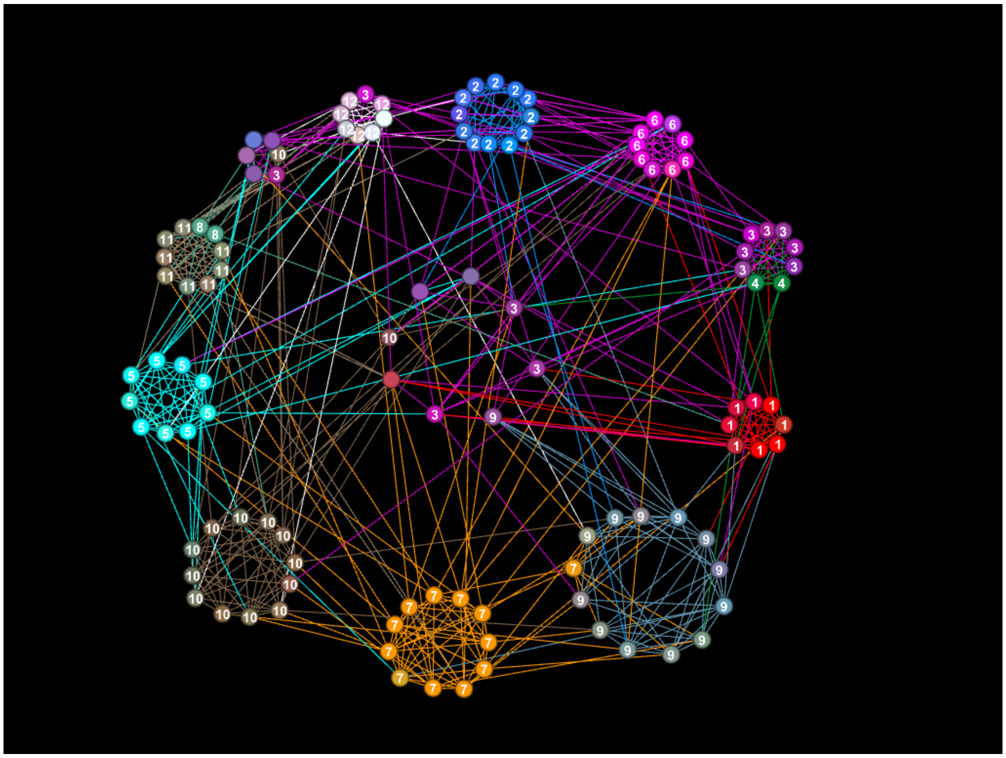

American Football League. onmi: 0.71194; thresould: 0.5

4 Adj-noun

```
In [40]: params={}
         params["-t"] = np.arange(0.05, 1.0, 0.05)
         all_results = lp_experiment(clustersNumber=2,
                       algorithm = "pmp",
                       distance = "cm",
                       inputFile =  "../datasets/adjnoun/adjnoun.dat",
                       groundTruth = "../datasets/adjnoun/truth_adjnoun.dat",
                       params = params,
                       vertexNumerationShift=0,
                       benchmarkFormat=True, verbose=True, lineGraph=True)
```

```
Output dir name: ../Results/lp_pmp_cm_adjnoun
Output file name: ../Results/lp_pmp_cm_adjnoun/pmp_adjnoun_CM_pmp_2.dat
```

```
HBox(children=(IntProgress(value=0, max=19), HTML(value='')))
```

```
Best ONMI: 0.00953164 params: '-t 0.05'
```

```
In [41]: plot_all_params(algorithm = "LPAM-Exact + acm", dataset = "Adj noun", all_results =
         all_results)
```

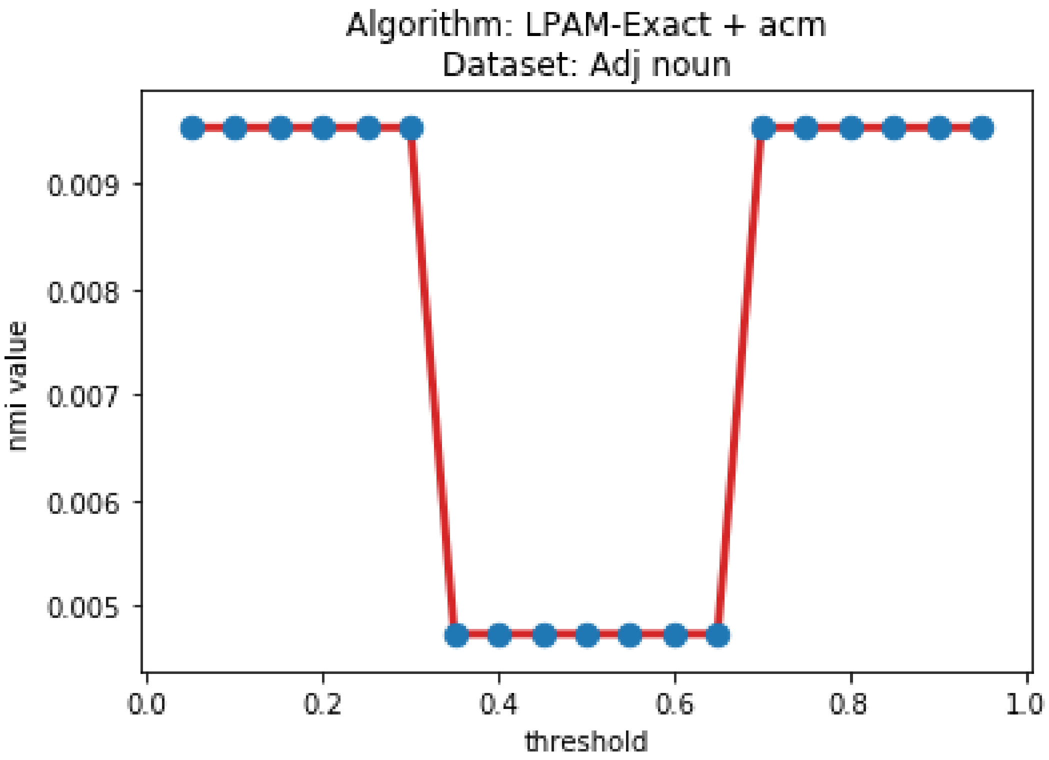

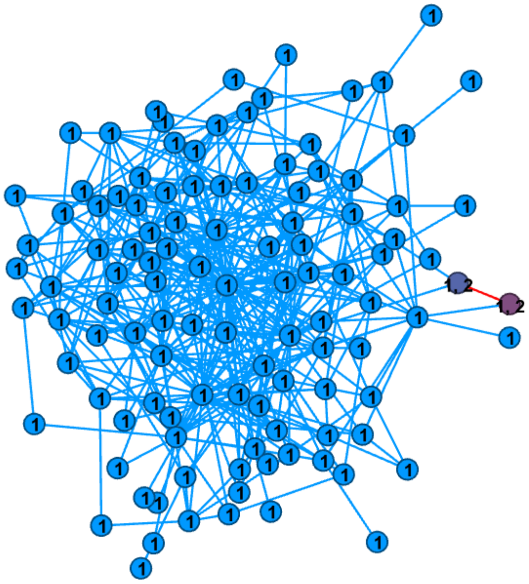

adjn-oun. onmi: 0.00490969; thresould: 0.1

5 Political Books

```
In [21]: params={}
         params["-t"] = np.arange(0.05, 1.0, 0.05)
         all_results = lp_experiment(clustersNumber=2,
                         algorithm = "pmp",
                         distance = "cm",
                         inputFile = "../datasets/polbooks/polbooks.dat",
                         groundTruth = "../datasets/polbooks/truth_polbooks.dat",
                         params = params,
                         vertexNumerationShift=0,
                         benchmarkFormat=True, verbose=False, lineGraph=True)
```

```
Output dir name: ../Results/lp_pmp_cm_polbooks
Output file name: ../Results/lp_pmp_cm_polbooks/pmp_polbooks_CM_pmp_2.dat
```

```
HBox(children=(IntProgress(value=0, max=19), HTML(value='')))
```

```
Best ONMI: 0.477871 params: '-t 0.55'
```

```
In [22]: plot_all_params(algorithm = "LPAM-exact + Commute Distance", dataset = "Politics Books",
         all_results = all_results)
```

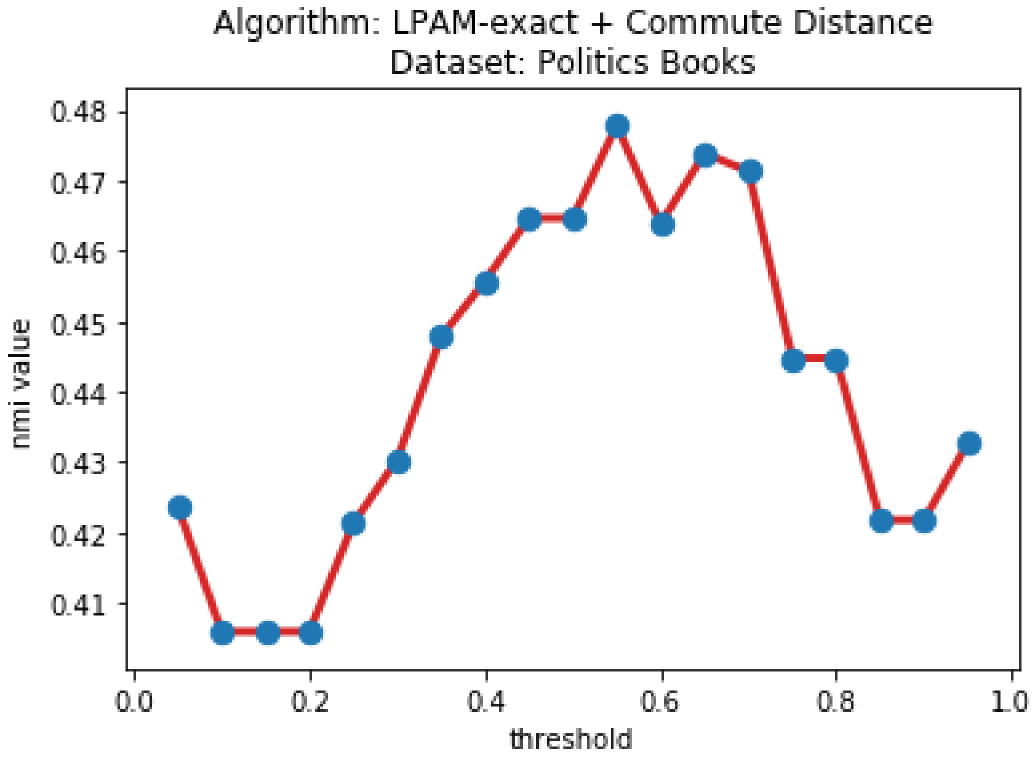

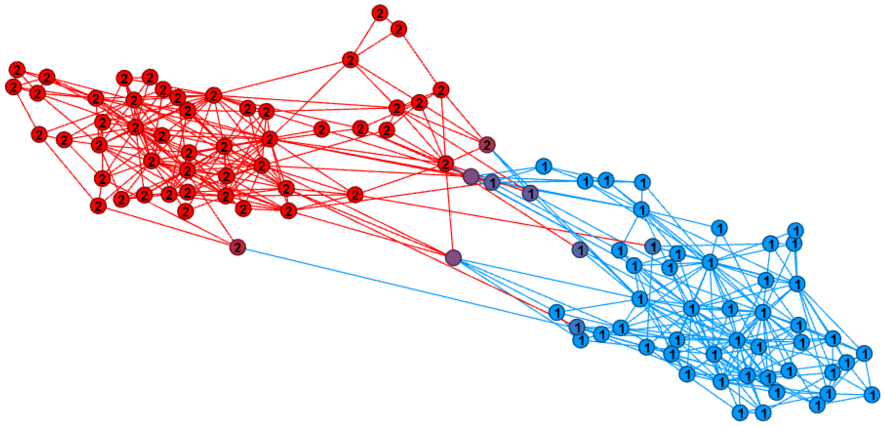

Political Books

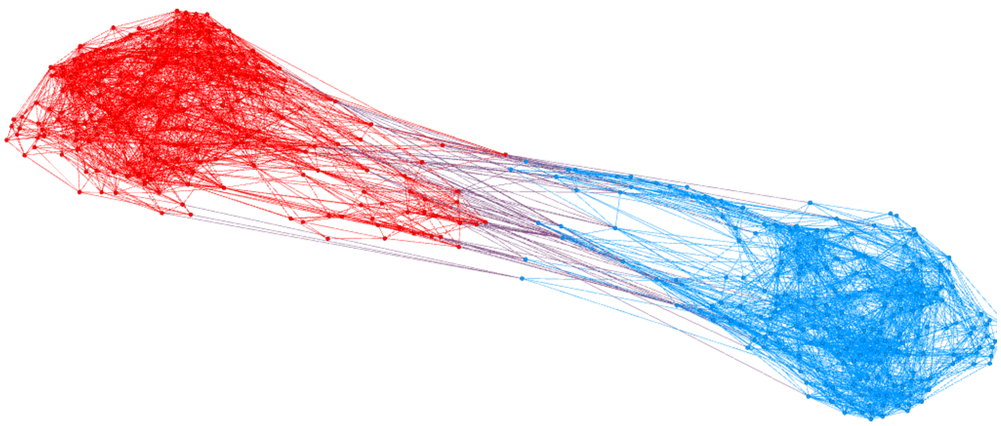

Political Books - Line Graph

6 bench_30_network

```
In [147]: params={}
          params["-t"] = np.arange(0.05, 1.0, 0.05)
          all_results = lp_experiment(clustersNumber=3,
                        algorithm = "pmp",
                        distance = "cm",
                        inputFile = "../datasets/bench_30/bench_30_network.dat",
                        groundTruth = "../datasets/bench_30/bench_30_truth.dat",
                        params = params,
                        vertexNumerationShift=0,
                        benchmarkFormat=True)
```

```
Output dir name: ../Results/lp_pmp_cm_bench_30
Output file name: ../Results/lp_pmp_cm_bench_30/pmp_bench_30_network_CM_pmp_3.dat
```

```
HBox(children=(IntProgress(value=0, max=19), HTML(value='')))
```

```
Best ONMI: 0.931866 params: '-t 0.35000000000000003'
```

```
In [148]: plot_all_params(algorithm = "LPAM-exact + Commute Distance", dataset = "bench_30",
          all_results = all_results)
```

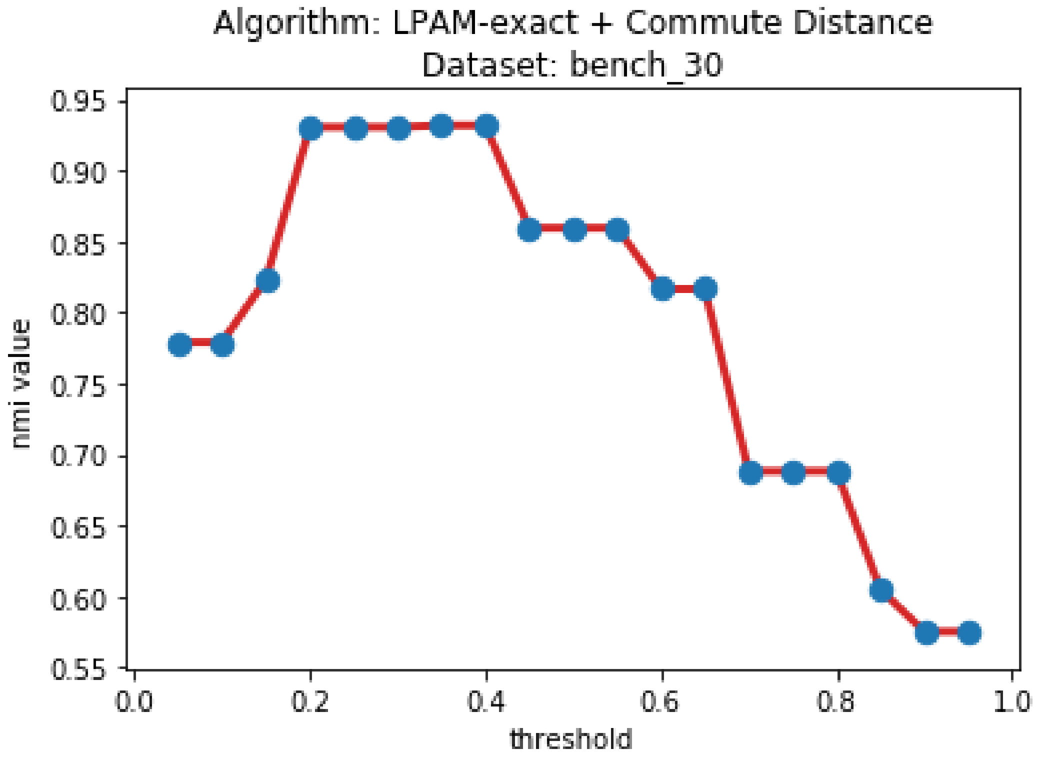
Algorithm: LPAM-exact + Commute Distance
Dataset: bench_30
nmi value
threshold
0.95
0.90
0.85
0.80
0.75
0.70
0.65
0.60
0.55
0.0
0.2
0.4
0.6
0.8
1.0

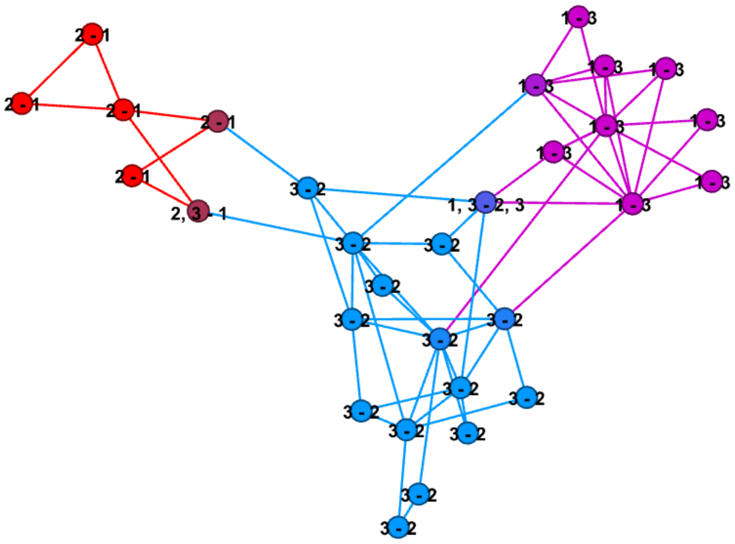

bench_30

7 bench_40_network

```
In [150]: params={}
          params["-t"] = np.arange(0.05, 1.0, 0.05)
          all_results = lp_experiment(clustersNumber=3,
                        algorithm = "pmp",
                        distance = "cm",
                        inputFile = "../datasets/bench_40/bench_40_network.dat",
                        groundTruth = "../datasets/bench_40/bench_40_truth.dat",
                        params = params,
                        vertexNumerationShift=0,
                        benchmarkFormat=True)
```

```
Output dir name: ../Results/lp_pmp_cm_bench_40
Output file name: ../Results/lp_pmp_cm_bench_40/pmp_bench_40_network_CM_pmp_3.dat
```

```
HBox(children=(IntProgress(value=0, max=19), HTML(value='')))
```

```
Best ONMI: 0.203899 params: '-t 0.7500000000000001'
```

```
In [151]: plot_all_params(algorithm = "LPAM-exact + Commute Distance", dataset = "bench_40",
          all_results = all_results)
```

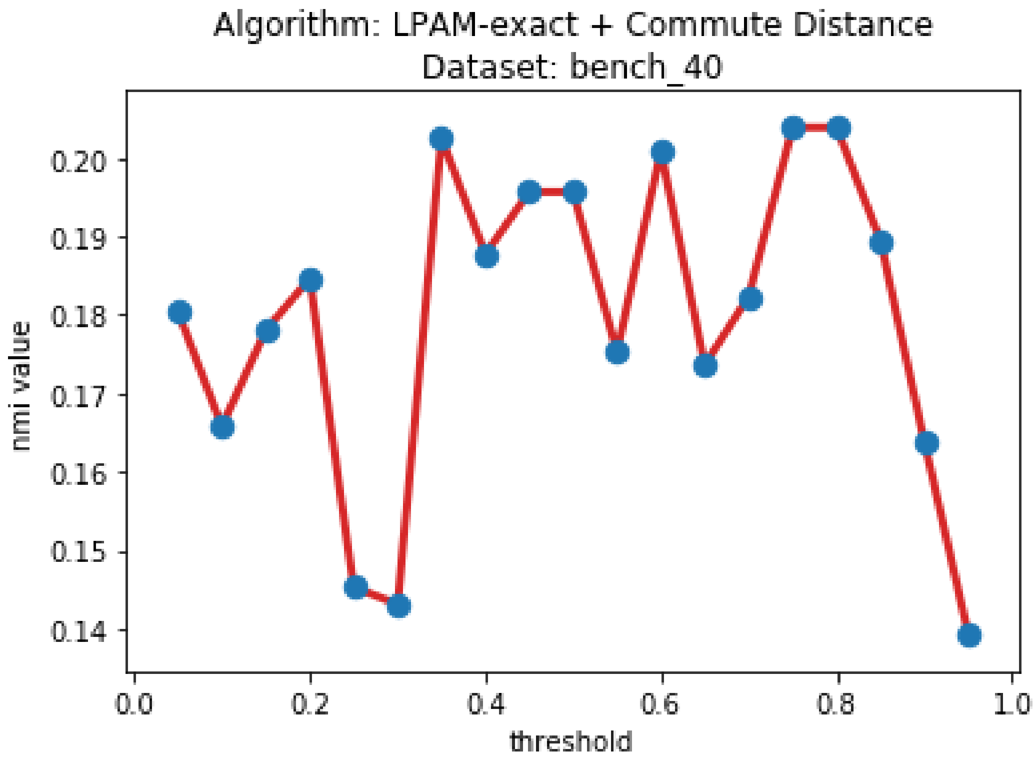

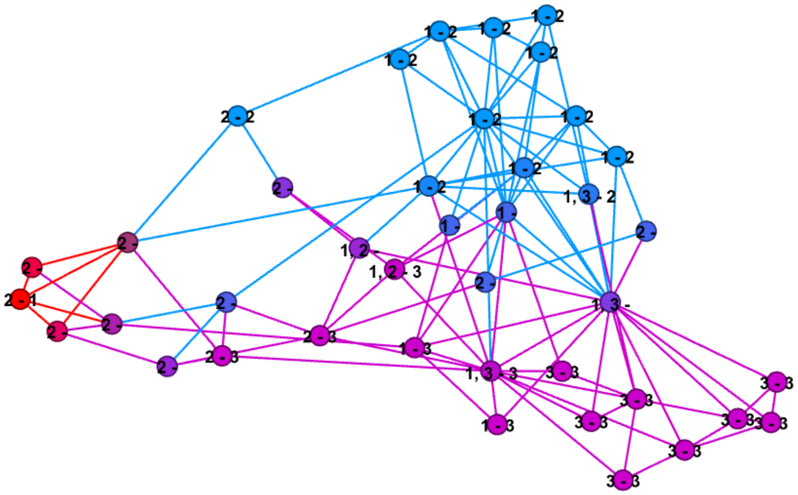

bench_40

8 bench_50

```
In [156]: params={}
          params["-t"] = np.arange(0.05, 1.0, 0.05)
          all_results = lp_experiment(clustersNumber=4,
                        algorithm = "pmp",
                        distance = "cm",
                        inputFile = "../datasets/bench_50/bench_50_network.dat",
                        groundTruth = "../datasets/bench_50/bench_50_truth.dat",
                        params = params,
                        vertexNumerationShift=0,
                        benchmarkFormat=True)
```

```
Output dir name: ../Results/lp_pmp_cm_bench_50
Output file name: ../Results/lp_pmp_cm_bench_50/pmp_bench_50_network_CM_pmp_4.dat
```

```
HBox(children=(IntProgress(value=0, max=19), HTML(value='')))
```

```
Best ONMI: 0.309034 params: '-t 0.25'
```

```
In [154]: plot_all_params(algorithm = "LPAM-exact + Commute Distance", dataset = "bench_50",
          all_results = all_results)
```

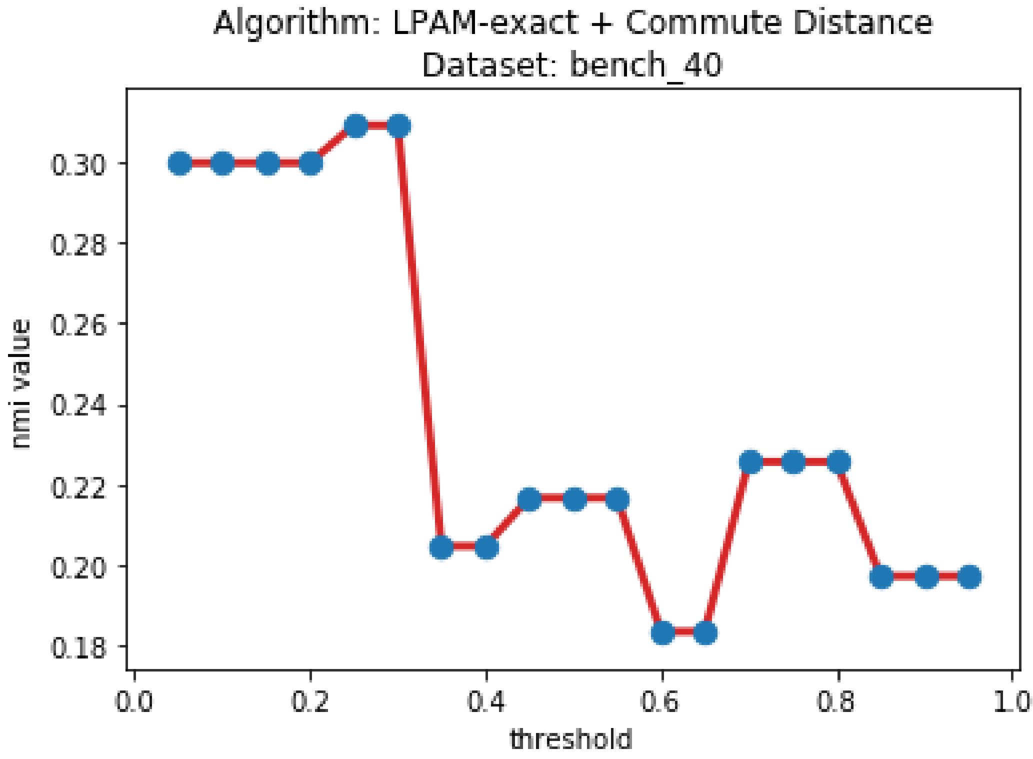
Algorithm: LPAM-exact + Commute Distance
Dataset: bench_40
nmi value
threshold
0.30
0.28
0.26
0.24
0.22
0.20
0.18
0.0
0.2
0.4
0.6
0.8
1.0

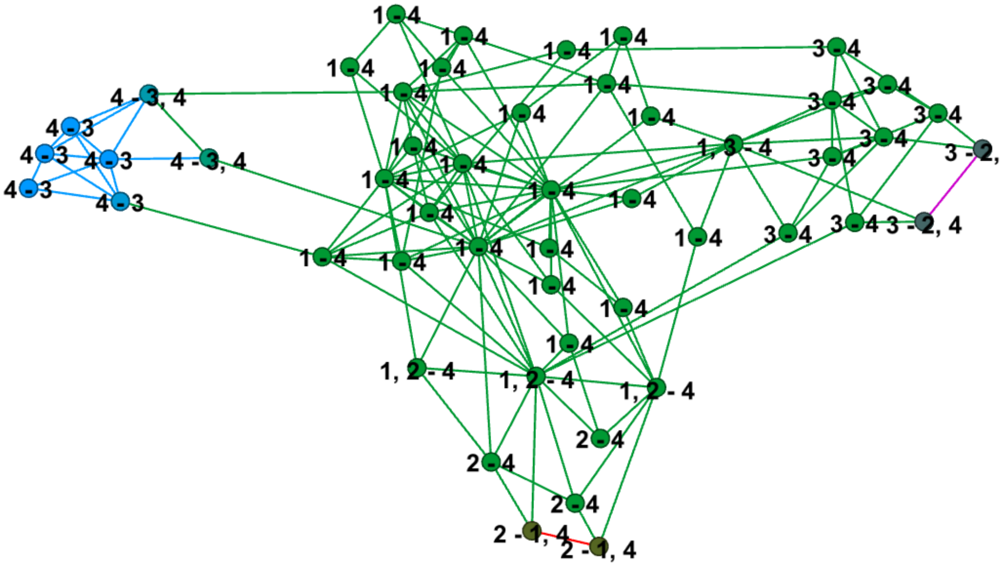

bench_50

9 bench_60

In [157]:
```
params={}
params["-t"] = np.arange(0.05, 1.0, 0.05)
all_results = lp_experiment(clustersNumber=6,
                            algorithm = "pmp",
                            distance = "cm",
                            inputFile = "../datasets/bench_60/bench_60_network.dat",
                            groundTruth = "../datasets/bench_60/bench_60_truth.dat",
                            params = params,
                            vertexNumerationShift=0,
                            benchmarkFormat=True)
```

```
Output dir name: ../Results/lp_pmp_cm_bench_60
Output file name: ../Results/lp_pmp_cm_bench_60/pmp_bench_60_network_CM_pmp_6.dat
```

```
HBox(children=(IntProgress(value=0, max=19), HTML(value='')))
```

```
Best ONMI: 0.405531 params: '-t 0.05'
```

In [158]:
```
plot_all_params(algorithm = "LPAM-exact + Commute Distance", dataset = "bench_60",
                all_results = all_results)
```

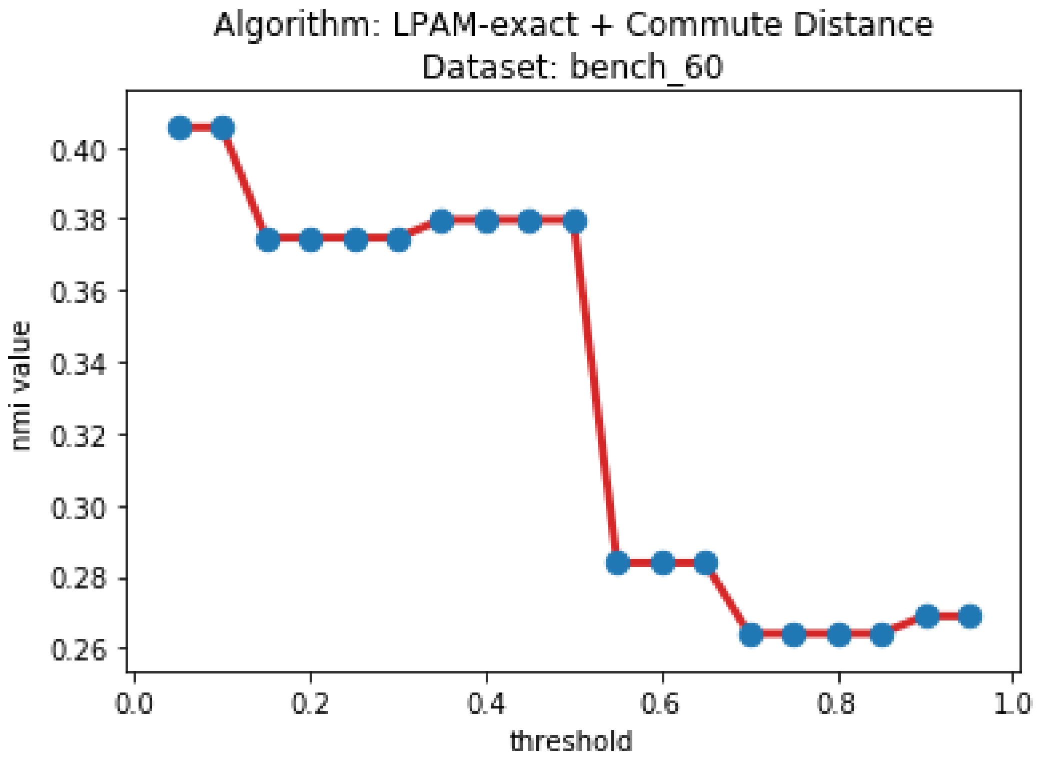

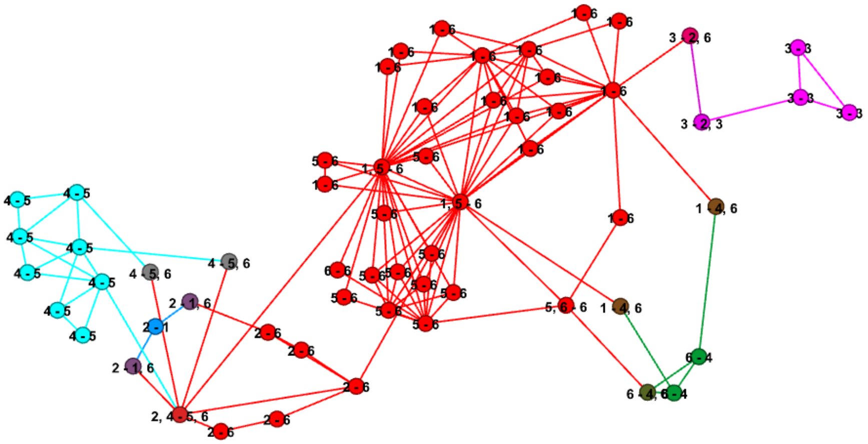

bench_60

10 bench_60_dense

```
In [160]: params={}
          params["-t"] = np.arange(0.05, 1.0, 0.05)
          all_results = lp_experiment(clustersNumber=6,
                        algorithm = "pmp",
                        distance = "cm",
                        inputFile = "../datasets/bench_60_dense/bench_60_dense_network.dat",
                        groundTruth = "../datasets/bench_60_dense/bench_60_dense_truth.dat",
                        params = params,
                        vertexNumerationShift=0,
                        benchmarkFormat=True)
```

```
Output dir name: ../Results/lp_pmp_cm_bench_60_dense
Output file name:
../Results/lp_pmp_cm_bench_60_dense/pmp_bench_60_dense_network_CM_pmp_6.dat
```

```
HBox(children=(IntProgress(value=0, max=19), HTML(value='')))
```

```
Best ONMI: 0.112146 params: '-t 0.05'
```

```
In [162]: plot_all_params(algorithm = "LPAM-exact + Commute Distance", dataset = "bench_60_dense",
          all_results = all_results)
```

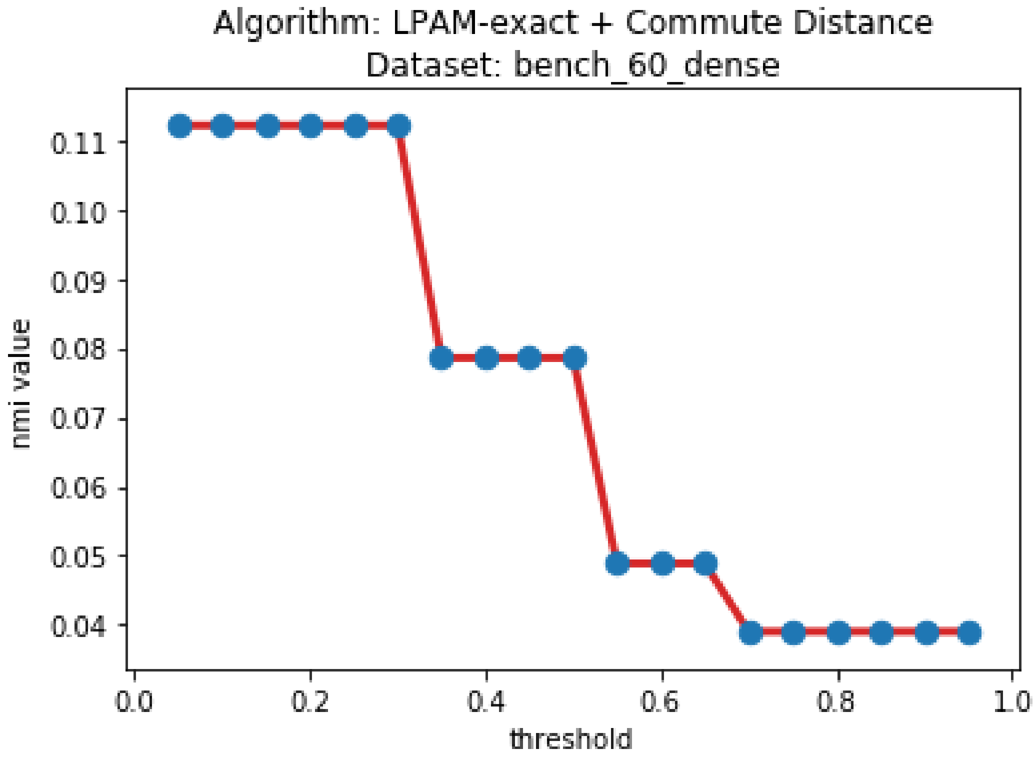

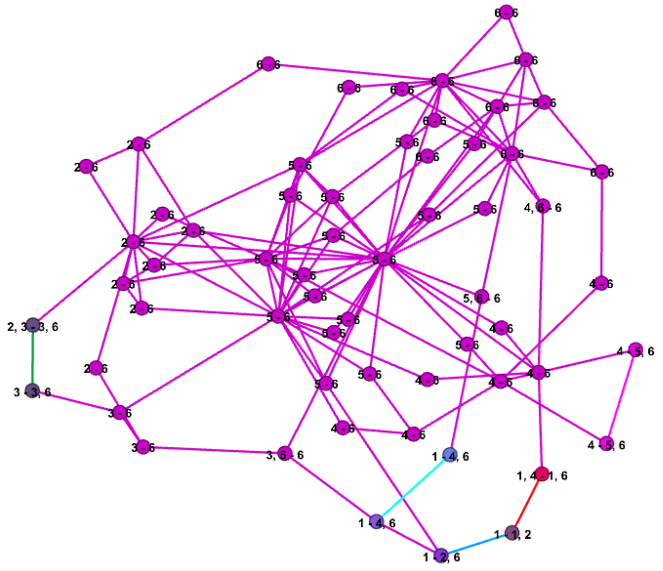

bench_60_dense

11 Format transformet Helper Tool

```
In [159]: #Transform from benchmark community format to onmi-tool community structure format
          oldF = "../datasets/bench_60_dense/bench_60_dense_community.dat"
          newF = "../datasets/bench_60_dense/bench_60_dense_truth.dat"

          v = {}
          comm = {}
          with open(oldF, "r") as ins:
              array = []
              for line in ins:
                  _id = line.split()[0]
                  v[_id] = [int(_c) for _c in line.split()[1: ]]
                  for _c in line.split()[1: ]:
                      if int(_c) in comm:
                          comm[int(_c)].append(_id)
                      else:
                          comm[int(_c)] = [_id]

          with open(newF, 'w') as the_file:
              for _c in comm:
                  the_file.write(" ".join(comm[_c] ) + '\n')
          !cat {newF}
```

```
1 4 20 26 29
2 7 8 23 38 43 44 45 46 56
2 3 31 32 34
5 6 12 14 20 22 24 27 37 50 53
9 10 13 16 18 19 21 28 32 33 35 40 41 42 47 49 55 58 59 60
11 15 17 19 25 27 30 36 39 48 51 52 54 57
```
