# Supplementary material for: Overlapping community detection in networks based on link partitioning and partitioning around medoids
Source: PLoS One. 2021 Aug 25;16(8):e0255717. doi: 10.1371/journal.pone.0255717 (PMC8386890; doi:10.1371/journal.pone.0255717)
Supplement: S4 Appendix — The computational results for the heuristic version of the LPAM method with commute distance. (PDF) [file pone.0255717.s004.pdf]

LPAM-Heuristic-Clarans-Commute-Distance

July 18, 2019

Link Partitioning by Partioning Around Medoids with Clarans Heuristic
Distance function: Commute Distance

```
In [1]: import numpy as np
        import random
        random.seed = 108
        from tqdm import tqdm_notebook as tqdm
        import seaborn as sns
        import matplotlib.pyplot as plt
        from mpl_toolkits import mplot3d
        import pandas as pd
        %matplotlib inline
```

```
In [2]: !java -jar ../lpam/target/Clustering-1.0-SNAPSHOT-jar-with-dependencies.jar
```

```
Missing required options: i, k
usage: PMPClustering <-i input_file> <-k number_of_clusters> [-ftbcd]
 -a,--algorithm <arg>      Specifies algorithm that will be used to find
                           disjoint edge clusters. Possible values:
                           pmp (p-median exact algorithm).
                           kmd (k-medoids heuristic)
                           kmn (k-means heuristic).
                           If this option is omitted, the P-Median
                           algorithm will be used.
 -b,--benchmark            use benchmark format (.dat file)
 -d,--distance <arg>       the type of function to measure the distance
                           between nodes.
                           Possible values:
                           sp (shortest path)
                           gd (Generalize Degree)
                           cm (Commute Distance)
                           acm (Amplified Commute Distance).
                           If this option is omitted, the amplified
                           commute distance function will be used.
 -f,--force                The previous founded solution by lp_solver will
                           not be used. lp_solver will be started
                           forcibly.
                           The flag effects only the PMP exact edge
                           clustering algorithm.
                           By default algorithm will try to get previous
                           founded solution
 -gt,--groundTruth <arg>   load info about ground truth community file
 -i,--input <arg>          path to the input file in GML format
 -k <arg>                  Number of clusters to detect (int)
 -l,--linegraph            produce line graph as output
 -o,--outputDir <arg>      the name of the output file. If option was
                           omitted, the name of the output file
```

```
                         will be composed automatically as
                         "{suffix}_{distanceName}_{clusterNumber}_out.ge
                         xf"
 -t,--threshold <arg>    Vertex i belongs to cluster C, if node i has
                         fraction of edges in cluster C greater then
                         this value
                         Default value is 0.
```

```
In [3]: def lp_experiment(clustersNumber, algorithm, distance, inputFile, groundTruth, params =
        {}, vertexNumerationShift=0, benchmarkFormat=False):
            path_to_jar = "../lpam/target/Clustering-1.0-SNAPSHOT-jar-with-dependencies.jar"
            datasetName = inputFile.split('/')[-2]
            sufix = inputFile.split('/')[-1].split('.')[0]
            outputDir = "../Results/lp_{}_{}_{}".format(algorithm, distance, datasetName)
            outputFile = outputDir +'/' +"pmp_{3}_{2}_{0}_{1}.dat".format(algorithm,
        clustersNumber, distance.upper(), sufix)
            print("Output dir name: {}".format(outputDir) )
            print("Output file name: {}".format(outputFile) )
            all_results = {}
            bestParam = "not found"
            nmi_best = 0;
            param_list = list(generate_params(params))
            for param in tqdm(param_list):
                if benchmarkFormat:
                    tmp=!java -jar {path_to_jar} -b -a {algorithm} -o {outputDir} -i {inputFile}
        -k {clustersNumber} -d {distance} {param}
                    tmpFile = outputFile
                else:
                    tmp=!java -jar {path_to_jar} -a {algorithm} -o {outputDir} -i {inputFile} -k
        {clustersNumber} -d {distance} {param}
                    lines=[]
                    with open(outputFile) as f:
                        lines = f.readlines()
                    tmpFile="tmpFile.dat"
                    with open(tmpFile, 'w') as the_file:
                        for line in lines:
                            the_file.write(" ".join([str(int(a)+vertexNumerationShift) for a in
        line.split()]) + "\n")

                output=!../Overlapping-NMI/onmi {groundTruth} {tmpFile}
                nmi=float(output[0].split()[1])
                all_results[param] = nmi
                if nmi >  nmi_best:
                    bestParam = param
                    nmi_best = nmi
            #restorign solution for the best parameters
            if benchmarkFormat:
                tmp=!java -jar ../lpam/out/artifacts/Clustering_jar/Clustering.jar -b -a
        {algorithm} -o {outputDir} -i {inputFile} -k {clustersNumber} -d {distance} {bestParam}
                tmpFile = outputFile
            else:
                tmp=!java -jar ../lpam/out/artifacts/Clustering_jar/Clustering.jar -a
        {algorithm} -o {outputDir} -i {inputFile} -k {clustersNumber} -d {distance} {bestParam}
                lines=[]
                with open(outputFile) as f:
                    lines = f.readlines()
                tmpFile="tmpFile.dat"
                with open(tmpFile, 'w') as the_file:
                    for line in lines:
                        the_file.write(" ".join([str(int(a)+vertexNumerationShift) for a in
        line.split()]) + "\n")

            print("Best ONMI: {} params: '{}'".format(nmi_best, bestParam) )
            return all_results
```

```
In [52]: def generate_params(params):
```

```
    keys = list(params.keys())
    if len(keys) == 1:
        for value in params[keys[0]]:
            yield ( keys[0] + " " + str(value) )
    if len( keys ) > 1:
        for value in params[keys[0]]:
            for remain_params in generate_params({k:params[k] for k in keys[1:]}):
                yield ( keys[0] + " " + str(value) + " " + remain_params )
```

In [53]:
```
def plot_all_params(algorithm, dataset, all_results ):
    xdata=[]
    ydata=[]
    df = pd.DataFrame()
    for param, nmi in all_results.items():
        splited = param.split()
        xdata.append(float(splited[1]))
        ydata.append(nmi)
        df = df.append({'x': float(splited[1]), 'y': nmi}, ignore_index=True)

    plt.plot(xdata, ydata, 'C3', zorder=1, lw=3)
    # ax = plt.axes(projection='3d')
    plt.scatter(xdata, ydata,s=70,zorder=2)
    plt.xlabel('threshold')
    plt.ylabel('nmi value');
    plt.title('Algorithm: {}\nDataset: {}'.format(algorithm, dataset));
    plt.show()
```

1 School friendship network

In [54]:
```
params={}
params["-t"] = np.arange(0.05, 1.0, 0.05)
```

In [55]:
```
all_results = lp_experiment(clustersNumber=7,
               algorithm = "kmd",
               distance = "cm",
               inputFile =  "../datasets/school_friendship/school-2.gml",
               groundTruth = "../datasets/school_friendship/truth-school.dat",
               params = params,
               vertexNumerationShift=-1,
               benchmarkFormat=False)
```

```
Output dir name: ../Results/lp_kmd_cm_school_friendship
Output file name: ../Results/lp_kmd_cm_school_friendship/pmp_school-2_CM_kmd_7.dat
```

```
HBox(children=(IntProgress(value=0, max=19), HTML(value='')))
```

```
Best ONMI: 0.577798 params: '-t 0.7000000000000001'
```

In [56]:
```
plot_all_params(algorithm = "LPMA_Heuristic-CLARANS + commute Distance", dataset =
"School Friendship", all_results = all_results)
```

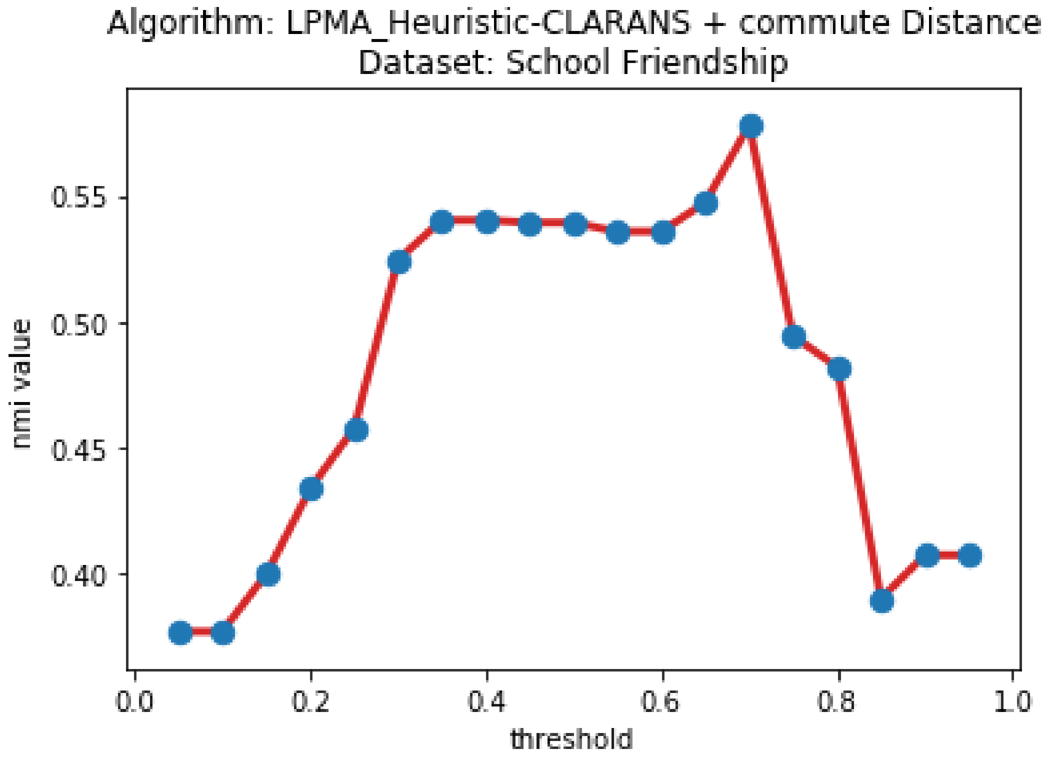
Algorithm: LPMA_Heuristic-CLARANS + commute Distance
Dataset: School Friendship
nmi value
0.55
0.50
0.45
0.40
0.0
0.2
0.4
0.6
0.8
1.0
threshold

1.1 Clustering results for School Frienship Netwrok with the best parameters

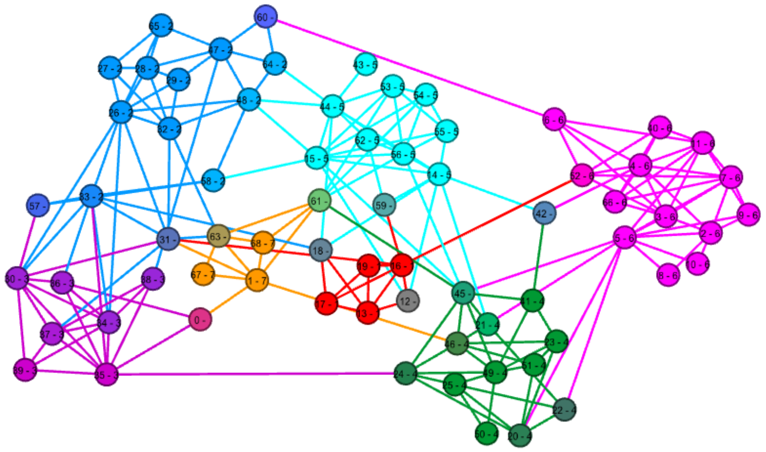

School friendship network

2 Karate Club

```
In [57]: params={}
         params["-t"] = np.arange(0.05, 1.0, 0.05)
         all_results = lp_experiment(clustersNumber=2,
                       algorithm = "kmd",
                       distance = "cm",
                       inputFile =  "../datasets/karate/karate.gml",
                       groundTruth = "../datasets/karate/truth_karate.dat",
                       params = params,
                       vertexNumerationShift=0,
                       benchmarkFormat=False)
```

```
Output dir name: ../Results/lp_kmd_cm_karate
Output file name: ../Results/lp_kmd_cm_karate/pmp_karate_CM_kmd_2.dat
```

```
HBox(children=(IntProgress(value=0, max=19), HTML(value='')))
```

```
Best ONMI: 0.91796 params: '-t 0.45'
```

```
In [58]: plot_all_params(algorithm = "LPAM-Heuristic + Commute Distance", dataset = "Karate Club
         ", all_results = all_results)
```

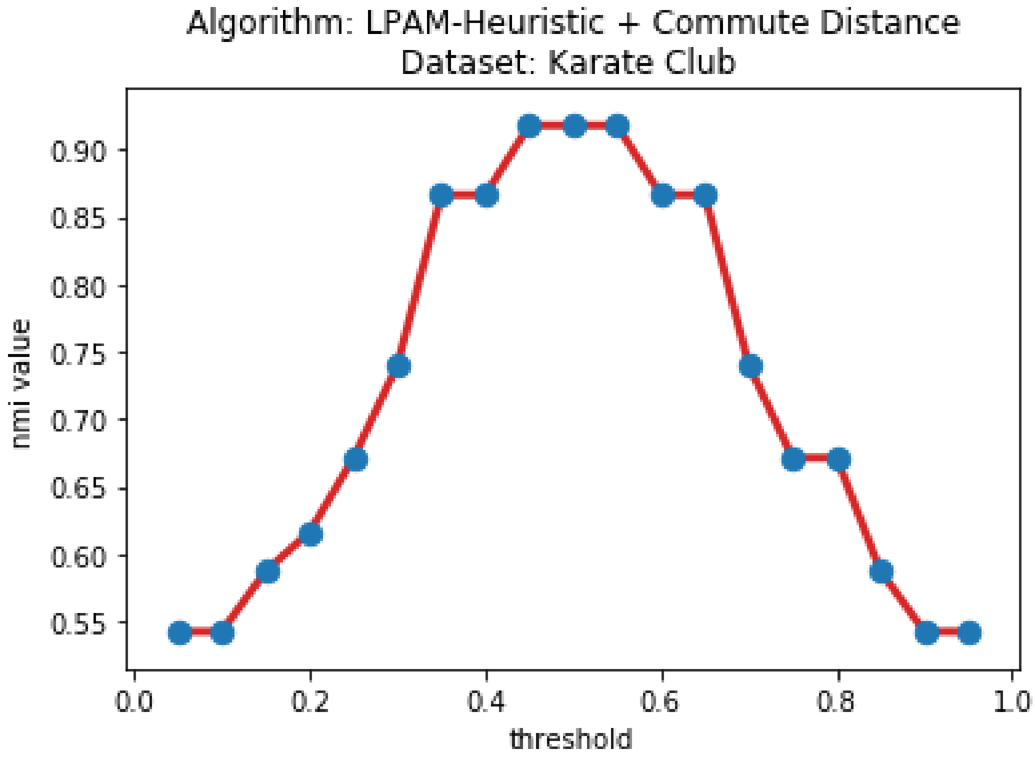
Algorithm: LPAM-Heuristic + Commute Distance
Dataset: Karate Club
nmi value
0.90
0.85
0.80
0.75
0.70
0.65
0.60
0.55
0.0
0.2
0.4
0.6
0.8
1.0
threshold

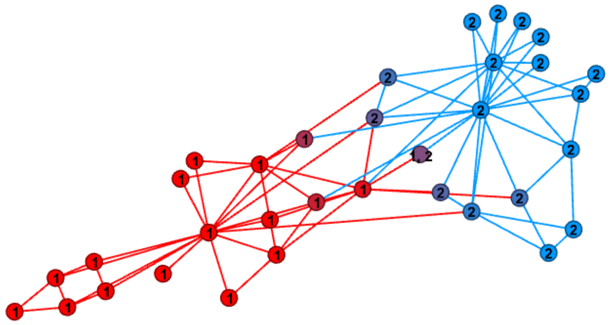

Karate Club

3 American Football League c = 12

```
In [59]: params={}
         params["-t"] = np.arange(0.05, 1.0, 0.05)
         all_results = lp_experiment(clustersNumber=12,
                       algorithm = "kmd",
                       distance = "cm",
                       inputFile =  "../datasets/football/footballTSEinput.gml",
                       groundTruth = "../datasets/football/truth_footballTSEinput.dat",
                       params = params,
                       vertexNumerationShift=-1,
                       benchmarkFormat=False)
```

```
Output dir name: ../Results/lp_kmd_cm_football
Output file name: ../Results/lp_kmd_cm_football/pmp_footballTSEinput_CM_kmd_12.dat
```

```
HBox(children=(IntProgress(value=0, max=19), HTML(value='')))
```

```
Best ONMI: 0.71194 params: '-t 0.5'
```

```
In [60]: plot_all_params(algorithm = "LPAM-Heuristic-Clarance + Commute Distance", dataset =
         "American Foolball League", all_results = all_results)
```

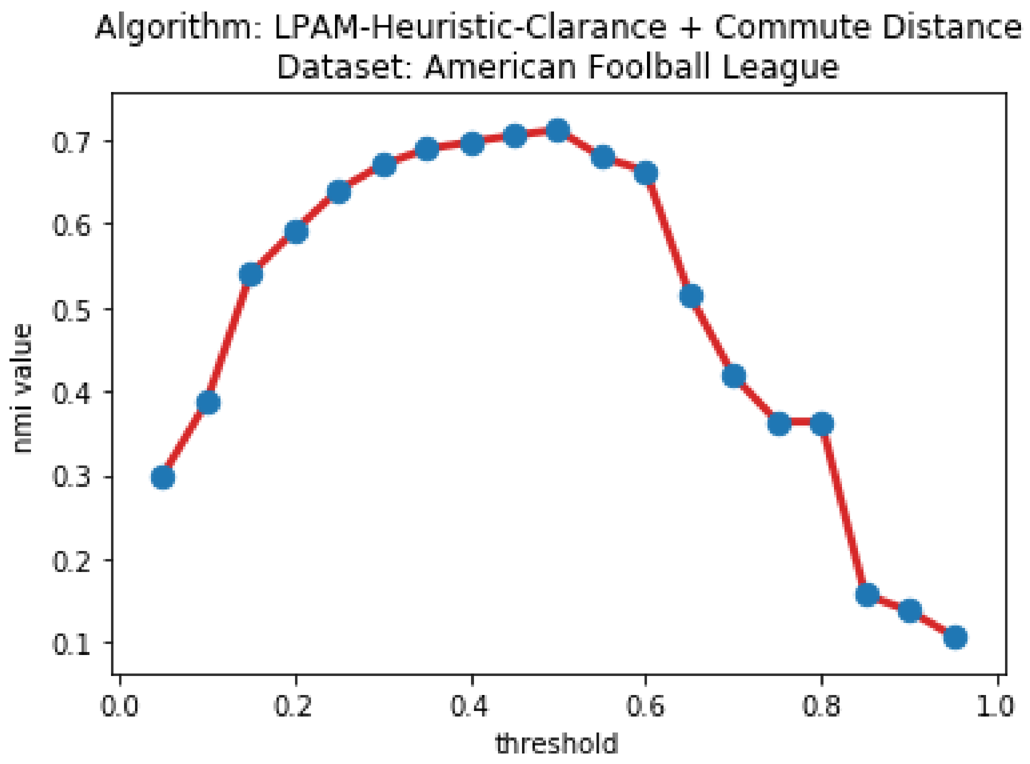

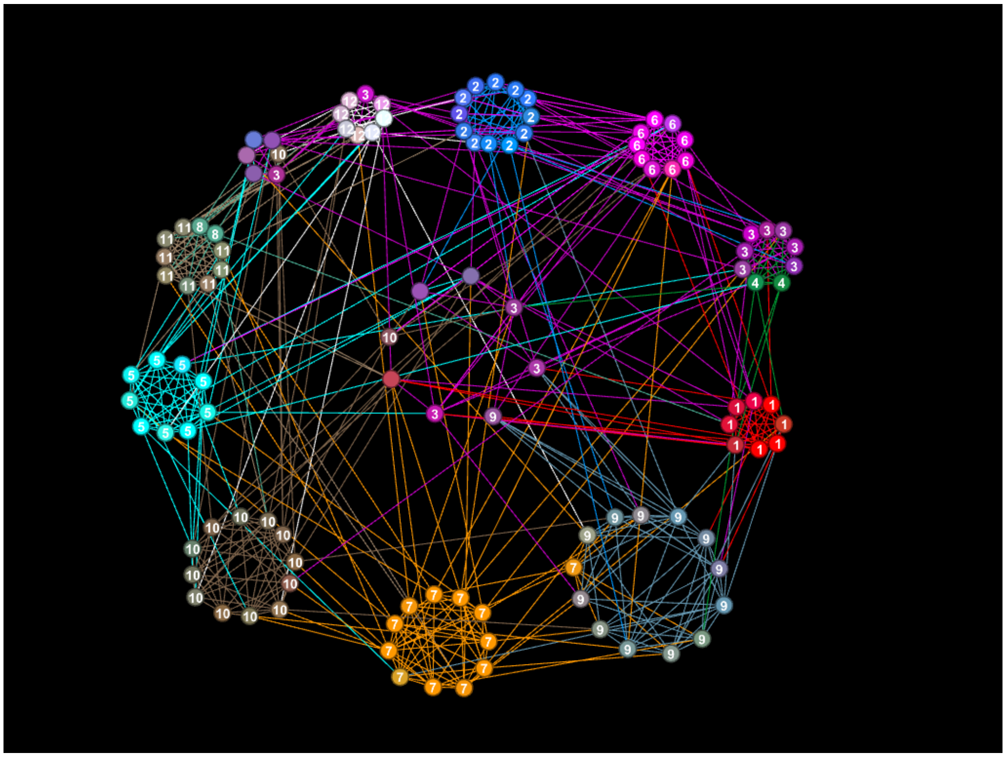

Americal Football League. onmi: 0.71194; thresould: 0.5

4 Adj-noun

```
In [61]: params={}
         params["-t"] = np.arange(0.05, 1.0, 0.05)
         all_results = lp_experiment(clustersNumber=2,
                         algorithm = "kmd",
                         distance = "cm",
                         inputFile =  "../datasets/adjnoun/adjnoun.dat",
                         groundTruth = "../datasets/adjnoun/truth_adjnoun.dat",
                         params = params,
                         vertexNumerationShift=0,
                         benchmarkFormat=True)
```

```
Output dir name: ../Results/lp_kmd_cm_adjnoun
Output file name: ../Results/lp_kmd_cm_adjnoun/pmp_adjnoun_CM_kmd_2.dat
```

```
HBox(children=(IntProgress(value=0, max=19), HTML(value='')))
```

```
Best ONMI: 0.00953164 params: '-t 0.05'
```

```
In [62]: plot_all_params(algorithm = "LPAM-Heuristic-Clarance + Commute Distance", dataset = "Adj
         noun", all_results = all_results)
```

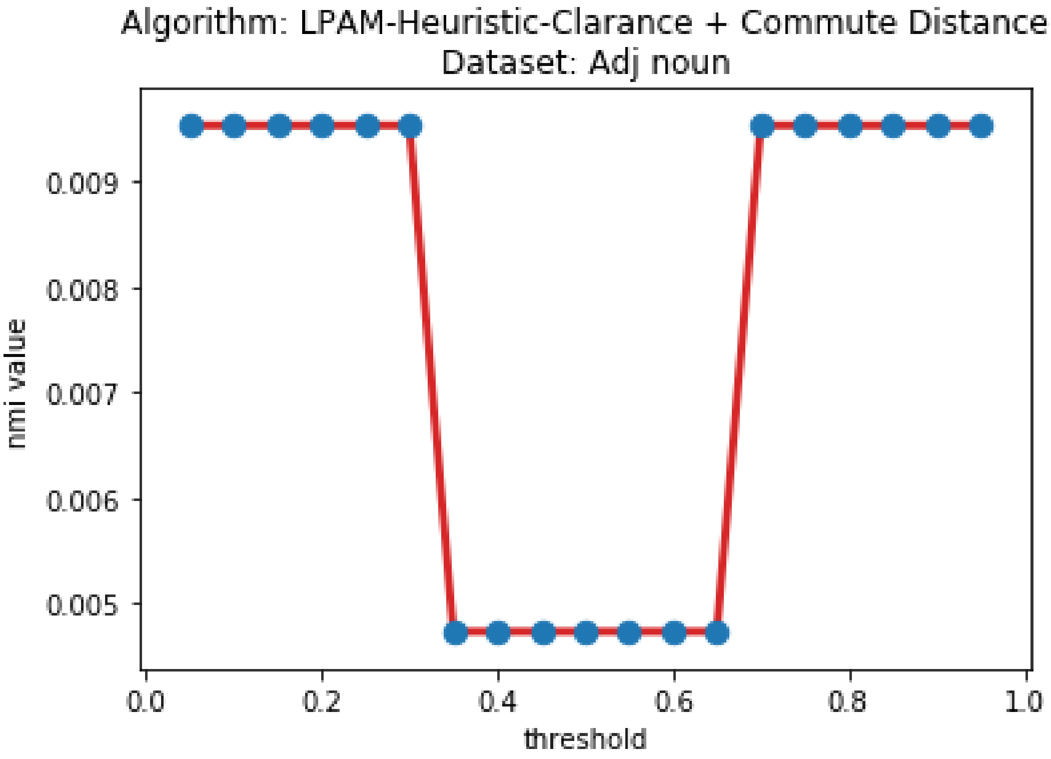

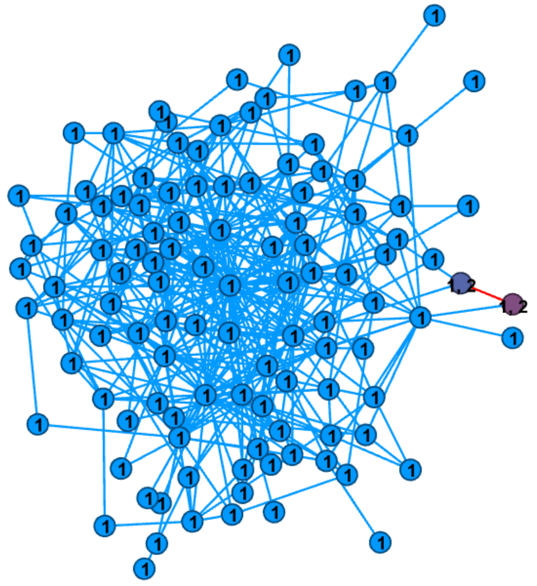

Adj-noun onmi: 0.00953164; thresould: 0.05

5 Political Books

```
In [63]: params={}
         params["-t"] = np.arange(0.05, 1.0, 0.05)
         all_results = lp_experiment(clustersNumber=2,
                        algorithm = "kmd",
                        distance = "cm",
                        inputFile = "../datasets/polbooks/polbooks.dat",
                        groundTruth = "../datasets/polbooks/truth_polbooks.dat",
                        params = params,
                        vertexNumerationShift=0,
                        benchmarkFormat=True)
```

```
Output dir name: ../Results/lp_kmd_cm_polbooks
Output file name: ../Results/lp_kmd_cm_polbooks/pmp_polbooks_CM_kmd_2.dat
```

```
HBox(children=(IntProgress(value=0, max=19), HTML(value='')))
```

```
Best ONMI: 0.440109 params: '-t 0.7000000000000001'
```

```
In [148]: plot_all_params(algorithm = "LPAM-Heuristic-Clarance + Commute Distance", dataset =
          "Politics Books", all_results = all_results)
```

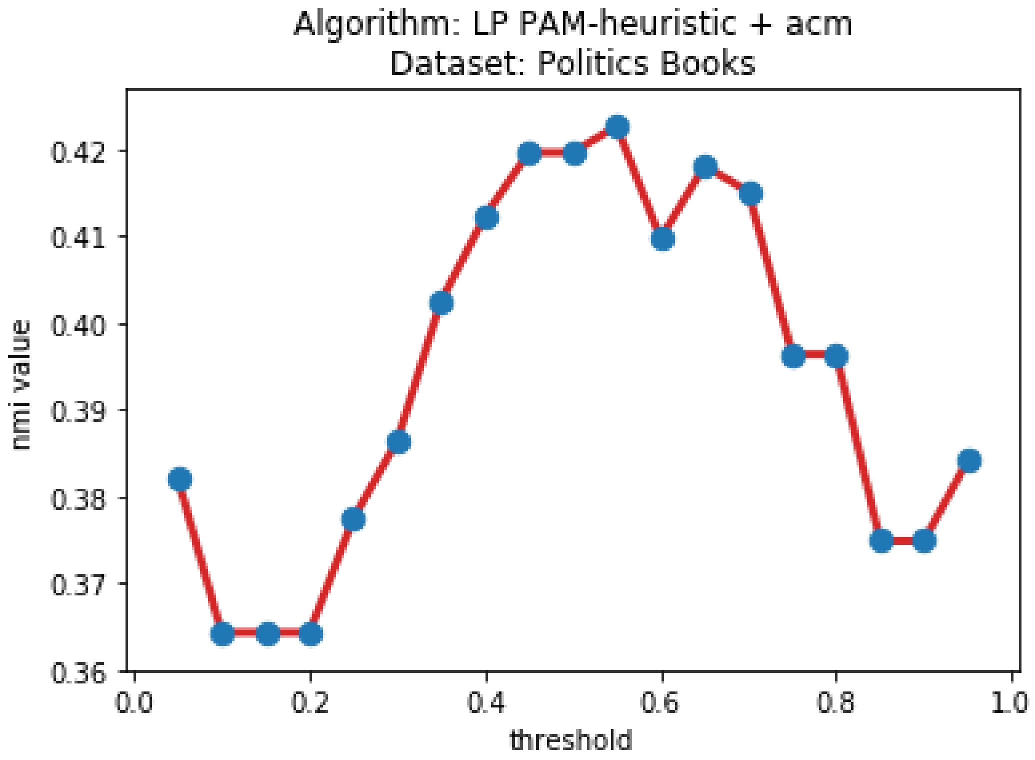

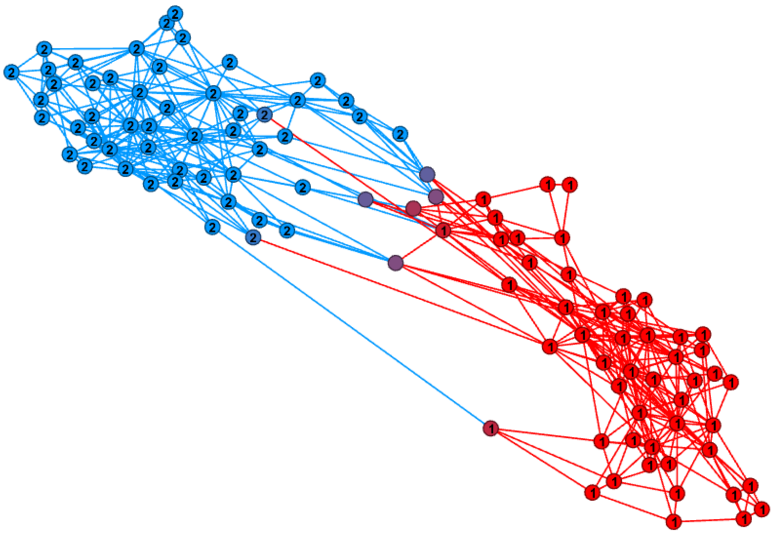

Political Books

6 Syntetic Datasets

6.1 bench_30_network

```
In [64]: params={}
         params["-t"] = np.arange(0.05, 1.0, 0.05)
         all_results = lp_experiment(clustersNumber=3,
                         algorithm = "kmd",
                         distance = "cm",
                         inputFile = "../datasets/bench_30/bench_30_network.dat",
                         groundTruth = "../datasets/bench_30/bench_30_truth.dat",
                         params = params,
                         vertexNumerationShift=0,
                         benchmarkFormat=True)
```

```
Output dir name: ../Results/lp_kmd_cm_bench_30
Output file name: ../Results/lp_kmd_cm_bench_30/pmp_bench_30_network_CM_kmd_3.dat
```

```
HBox(children=(IntProgress(value=0, max=19), HTML(value='')))
```

```
Best ONMI: 0.931866 params: '-t 0.35000000000000003'
```

```
In [65]: plot_all_params(algorithm = "LPAM-Heuristic-Clarance + Commute Distance", dataset =
         "bench_30", all_results = all_results)
```

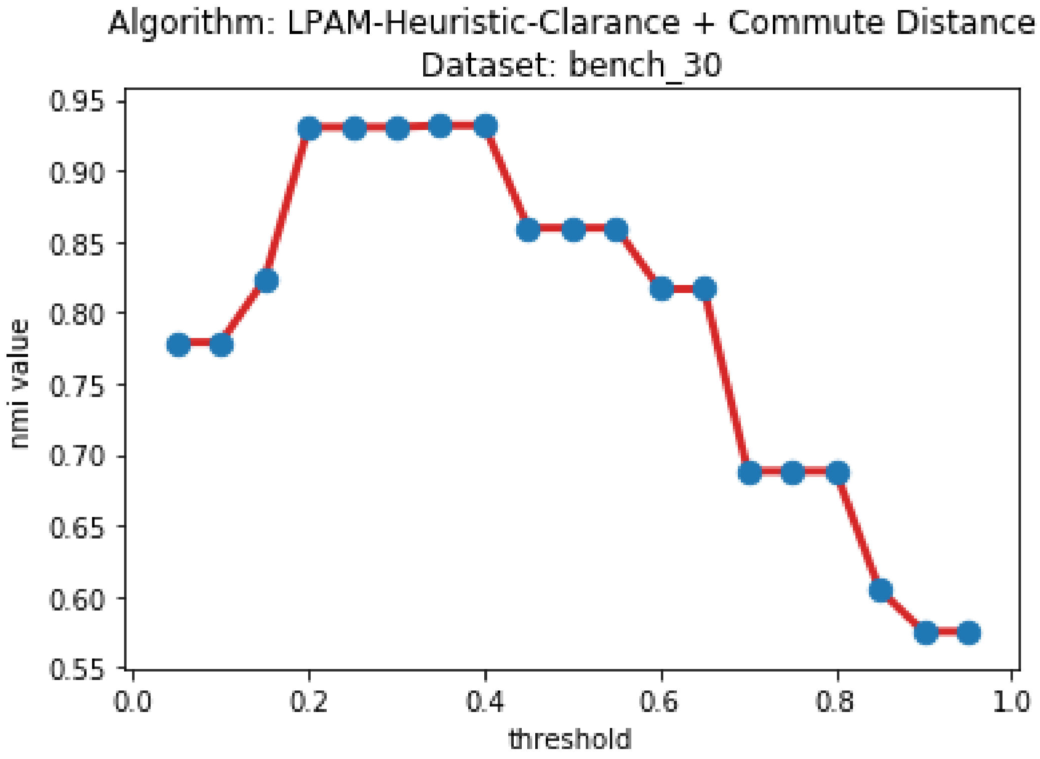

6.2 bench_40_network

```
In [66]: params={}
         params["-t"] = np.arange(0.05, 1.0, 0.05)
         all_results = lp_experiment(clustersNumber=3,
                       algorithm = "kmd",
                       distance = "cm",
                       inputFile = "../datasets/bench_40/bench_40_network.dat",
                       groundTruth = "../datasets/bench_40/bench_40_truth.dat",
                       params = params,
                       vertexNumerationShift=0,
                       benchmarkFormat=True)
```

```
Output dir name: ../Results/lp_kmd_cm_bench_40
Output file name: ../Results/lp_kmd_cm_bench_40/pmp_bench_40_network_CM_kmd_3.dat
```

```
HBox(children=(IntProgress(value=0, max=19), HTML(value='')))
```

```
Best ONMI: 0.203899 params: '-t 0.7500000000000001'
```

```
In [67]: plot_all_params(algorithm = "LPAM-Heuristic-Clarance + Commute Distance", dataset =
         "bench_40", all_results = all_results)
```

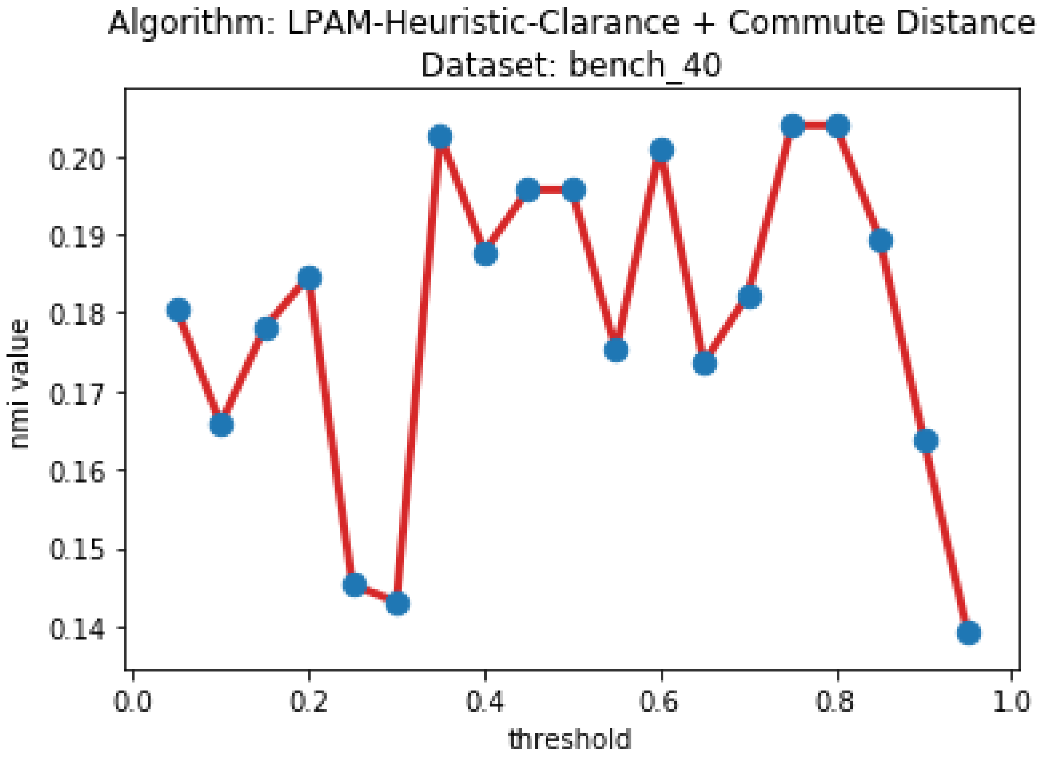

6.3 bench_50_network

```
In [68]: params={}
         params["-t"] = np.arange(0.05, 1.0, 0.05)
         all_results = lp_experiment(clustersNumber=3,
                       algorithm = "kmd",
                       distance = "cm",
                       inputFile = "../datasets/bench_50/bench_50_network.dat",
                       groundTruth = "../datasets/bench_50/bench_50_truth.dat",
                       params = params,
                       vertexNumerationShift=0,
                       benchmarkFormat=True)
```

```
Output dir name: ../Results/lp_kmd_cm_bench_50
Output file name: ../Results/lp_kmd_cm_bench_50/pmp_bench_50_network_CM_kmd_3.dat
```

```
HBox(children=(IntProgress(value=0, max=19), HTML(value='')))
```

```
Best ONMI: 0.275624 params: '-t 0.25'
```

```
In [69]: plot_all_params(algorithm = "LPAM-Heuristic-Clarance + Commute Distance", dataset =
         "bench_50", all_results = all_results)
```

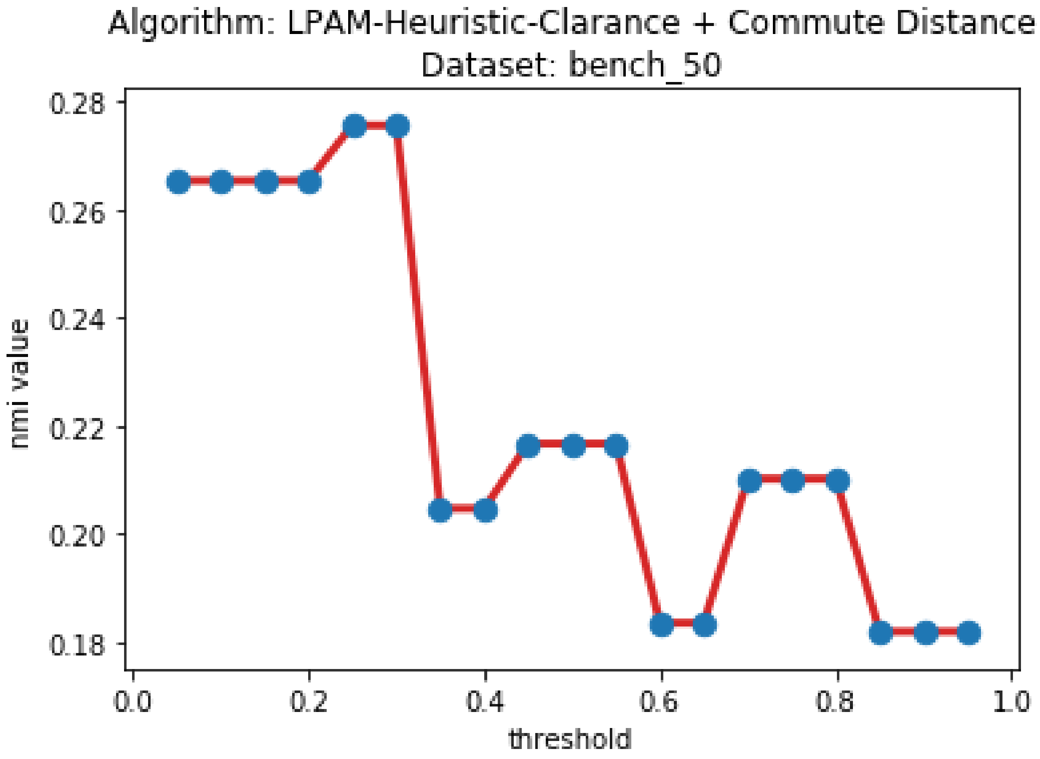

6.4 bench_60_network

```
In [70]: params={}
         params["-t"] = np.arange(0.05, 1.0, 0.05)
         all_results = lp_experiment(clustersNumber=3,
                         algorithm = "kmd",
                         distance = "cm",
                         inputFile = "../datasets/bench_60/bench_60_network.dat",
                         groundTruth = "../datasets/bench_60/bench_60_truth.dat",
                         params = params,
                         vertexNumerationShift=0,
                         benchmarkFormat=True)
```

```
Output dir name: ../Results/lp_kmd_cm_bench_60
Output file name: ../Results/lp_kmd_cm_bench_60/pmp_bench_60_network_CM_kmd_3.dat
```

```
HBox(children=(IntProgress(value=0, max=19), HTML(value='')))
```

```
Best ONMI: 0.307907 params: '-t 0.05'
```

```
In [71]: plot_all_params(algorithm = "LPAM-Heuristic-Clarance + Commute Distance", dataset =
         "bench_60", all_results = all_results)
```

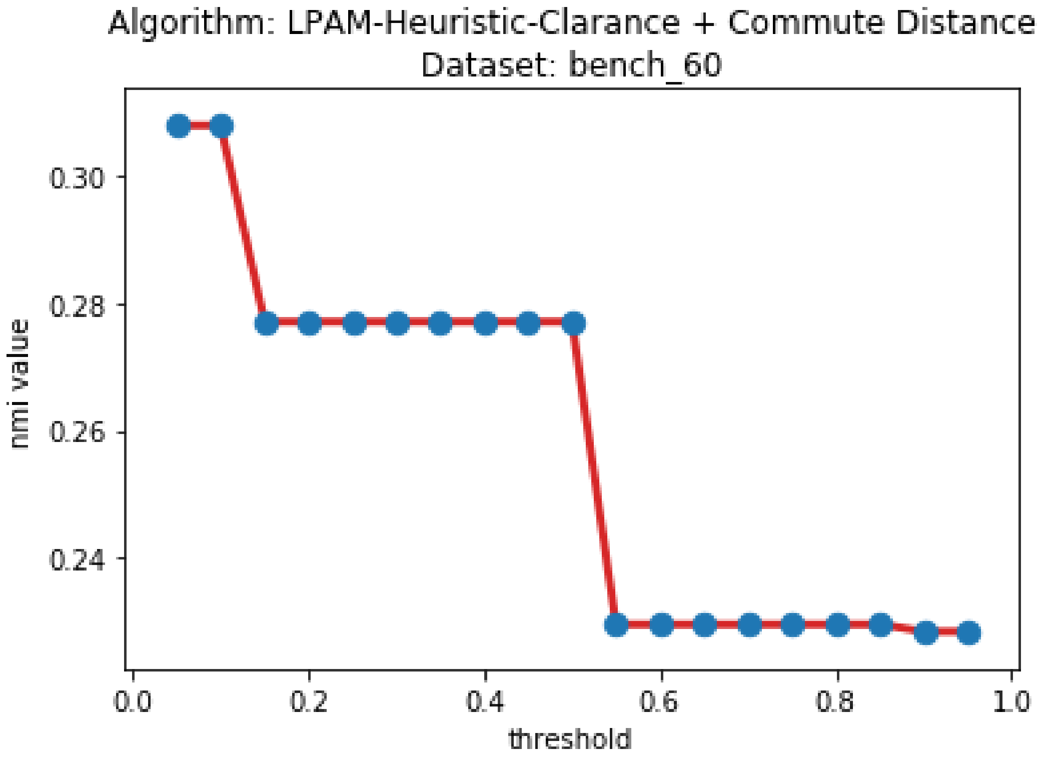

6.5 bench_60_dense_network

In [72]:
```
params={}
params["-t"] = np.arange(0.05, 1.0, 0.05)
all_results = lp_experiment(clustersNumber=3,
                algorithm = "kmd",
                distance = "cm",
                inputFile = "../datasets/bench_60_dense/bench_60_dense_network.dat",
                groundTruth = "../datasets/bench_60_dense/bench_60_dense_truth.dat",
                params = params,
                vertexNumerationShift=0,
                benchmarkFormat=True)
```

```
Output dir name: ../Results/lp_kmd_cm_bench_60_dense
Output file name:
../Results/lp_kmd_cm_bench_60_dense/pmp_bench_60_dense_network_CM_kmd_3.dat
```

```
HBox(children=(IntProgress(value=0, max=19), HTML(value='')))
```

```
Best ONMI: 0.0671985 params: '-t 0.05'
```

In [73]:
```
plot_all_params(algorithm = "LPAM-Heuristic-Clarance + Commute Distance", dataset =
"bench_60_dense", all_results = all_results)
```

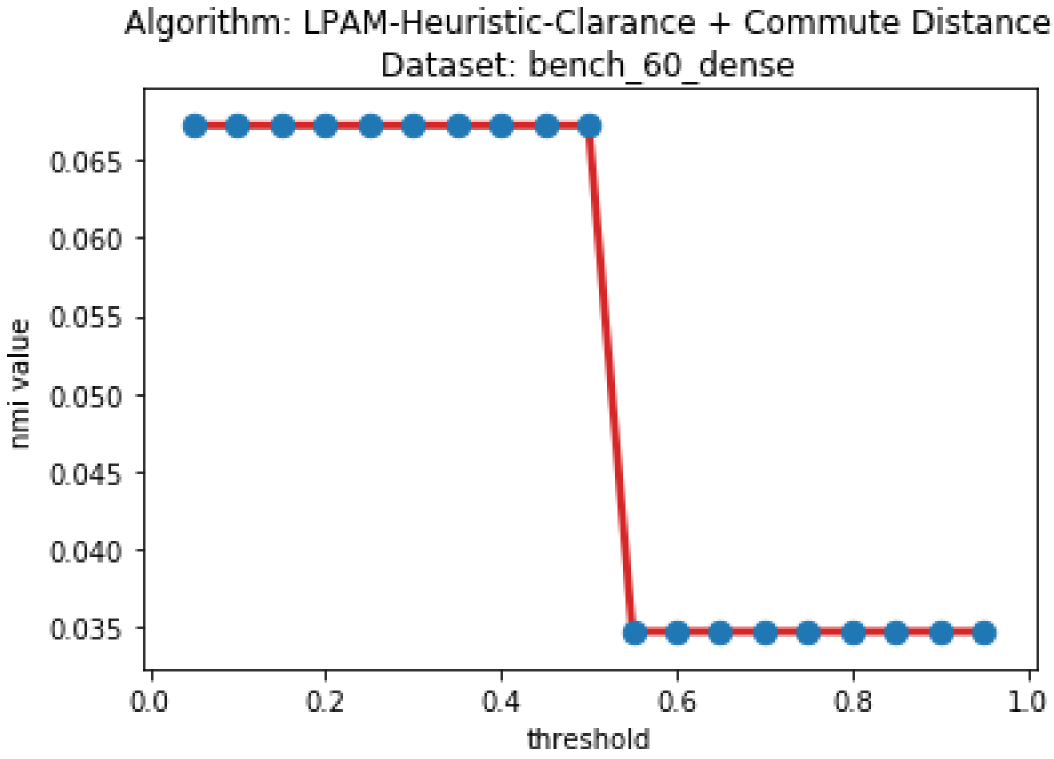
Algorithm: LPAM-Heuristic-Clarance + Commute Distance
Dataset: bench_60_dense
0.065
0.060
0.055
0.050
0.045
0.040
0.035
nmi value
0.0
0.2
0.4
0.6
0.8
1.0
threshold

```
In [ ]:
```
