# Supplementary material for: Overlapping community detection in networks based on link partitioning and partitioning around medoids
Source: PLoS One. 2021 Aug 25;16(8):e0255717. doi: 10.1371/journal.pone.0255717 (PMC8386890; doi:10.1371/journal.pone.0255717)
Supplement: S5 Appendix — The computational results for the Greedy Clique Expansion method. (PDF) [file pone.0255717.s005.pdf]

GCE

July 18, 2019

1 Greedy Clique Expansion Community Finder (GCE)

```
In [155]: import numpy as np
          import random
          random.seed = 108
          from tqdm import tqdm_notebook as tqdm
          import seaborn as sns
          import matplotlib.pyplot as plt
          from mpl_toolkits import mplot3d
          import pandas as pd
          import seaborn as sns; sns.set()
          %matplotlib inline
```

```
In [14]: !pwd
```

```
/home/latna/aponom/lpam-clustering/Scripts
```

```
In [19]: !ls ../related_methods/GCECommunityFinder/build/
```

```
aaron_utils.d                         GCECommunityFinder
aaron_utils.o                         graph_loading.d
cliques.d                             graph_loading.o
cliques.o                             graph_representation.d
Community_Finder.d                    graph_representation.o
Community_Finder.o                    makefile
find_communities.d                    Seed.d
find_communities.o                    Seed.o
gce_clustering_school_friendship.dat
```

```
In [16]: !(cd ../related_methods/GCECommunityFinder/build/ && make)
```

```
g++ -O3 -g3 -Wall -c -fmessage-length=0 -MMD -MP -MF"Seed.d" -MT"Seed.d" -o"Seed.o"
"../Seed.cpp"
g++ -O3 -g3 -Wall -c -fmessage-length=0 -MMD -MP -MF"Community_Finder.d"
-MT"Community_Finder.d" -o"Community_Finder.o" "../Community_Finder.cpp"
In file included from ../Community_Finder.h:14:0,
                 from ../Community_Finder.cpp:9:
../graph_loading.hpp:12:55: warning: dynamic exception
specifications are deprecated in C++11 [-Wdeprecated]
 const char *readEdge(const char *cur, int &l, int &r) throw
(readEdgeInvalidLineInDataException);
                                                       ^~~~~
g++ -O3 -g3 -Wall -c -fmessage-length=0 -MMD -MP -MF"aaron_utils.d" -MT"aaron_utils.d"
-o"aaron_utils.o" "../aaron_utils.cpp"
g++ -O3 -g3 -Wall -c -fmessage-length=0 -MMD -MP -MF"cliques.d" -MT"cliques.d"
```

```
-o"cliques.o" "../cliques.cpp"
g++ -O3 -g3 -Wall -c -fmessage-length=0 -MMD -MP -MF"find_communities.d"
-MT"find_communities.d" -o"find_communities.o" "../find_communities.cpp"
In file included from ../find_communities.cpp:5:0:
../graph_loading.hpp:12:55: warning: dynamic exception
specifications are deprecated in C++11 [-Wdeprecated]
 const char *readEdge(const char *cur, int &l, int &r) throw
(readEdgeInvalidLineInDataException);
                                                       ^~~~~
g++ -O3 -g3 -Wall -c -fmessage-length=0 -MMD -MP -MF"graph_loading.d"
-MT"graph_loading.d" -o"graph_loading.o" "../graph_loading.cpp"
In file included from ../graph_loading.cpp:11:0:
../graph_loading.hpp:12:55: warning: dynamic exception
specifications are deprecated in C++11 [-Wdeprecated]
 const char *readEdge(const char *cur, int &l, int &r) throw
(readEdgeInvalidLineInDataException);
                                                       ^~~~~
../graph_loading.cpp:34:55: warning: dynamic exception
specifications are deprecated in C++11 [-Wdeprecated]
 const char *readEdge(const char *cur, int &l, int &r) throw
(readEdgeInvalidLineInDataException);
                                                       ^~~~~
../graph_loading.cpp: In function ‘voidgraph_loading::findDistinctVertices(graph_loading::RangeOfEdges<int>,SimpleI
../graph_loading.cpp:148:23: warning: typedef
‘ID’ locally defined but not used [-Wunused-local-typedefs]
  typedef unsigned int ID;
                       ^~
../graph_loading.cpp: At global scope:
../graph_loading.cpp:334:55: warning: dynamic exception
specifications are deprecated in C++11 [-Wdeprecated]
 const char *readEdge(const char *cur, int &l, int &r) throw
(readEdgeInvalidLineInDataException) {
                                                       ^~~~~
g++ -O3 -g3 -Wall -c -fmessage-length=0 -MMD -MP -MF"graph_representation.d"
-MT"graph_representation.d" -o"graph_representation.o" "../graph_representation.cpp"
g++  -o "GCECommunityFinder"  ./Community_Finder.o ./Seed.o ./aaron_utils.o
./cliques.o ./find_communities.o ./graph_loading.o ./graph_representation.o
```

Use with either full (if specify all 5) or default (specify just graph file) parameters: Full parameters are: The name of the file to load

1) The minimum size of cliques to use as seeds. Recommend 4 as default, unless particularly small communities are required (in which case use 3). 2 The minimum value for one seed to overlap with another seed before it is considered sufficiently overlapping to be discarded (eta). 1 is complete overlap. However smaller values may be used to prune seeds more aggressively. A value of 0.6 is recommended. 3) The alpha value to use in the fitness function greedily expanding the seeds. 1.0 is recommended default. Values between .8 and 1.5 may be useful. As the density of edges increases, alpha may need to be increased to stop communities expanding to engulf the whole graph. If this occurs, a warning message advising that a higher value of alpha be used, will be printed. 4) The proportion of nodes (phi) within a core clique that must have already been covered by other cliques, for the clique to be 'sufficiently covered' in the Clique Coveage Heuristic

Usage: ./GCECommunityFinder graphfilename minimumCliqueSizeK overlapToDiscardEta fitnessExponentAlpha CCHthresholdPhi

The default values of: minimumCliqueSizeK 4, overlapToDiscardEta 0.6, fitnessExponentAlpha 1.0, CCHthresholdPhi .75

```
In [130]: def generate_params(params):
              keys = list(params.keys())
              if len(keys) == 1:
                  for value in params[keys[0]]:
                      yield ( keys[0] + " " + str(value) )
              if len( keys ) > 1:
                  for value in params[keys[0]]:
                      for remain_params in generate_params({k:params[k] for k in keys[1:]}):
                          yield ( keys[0] + " " + str(value) + " " + remain_params )
```

```
In [131]: def GCE_experiment(inputFile, groundTruth, params = {}, vertexNumerationShift=0,
          verbose=False):
              datasetName = inputFile.split('/')[-2]
              workingDir = "../Results/GCE_{}".format(datasetName)
              outputFile = workingDir + "/GCE_output.txt"
              print("workingDir: {}".format(workingDir) )
              print("outputFile: {}".format(outputFile) )

              !mkdir {workingDir}

              all_results = {}
              bestParam = "not found"
              nmi_best = 0;
              for param in tqdm(list(generate_params(params))):
                  if verbose:
                      !echo "../related_methods/GCECommunityFinder/build/GCECommunityFinder
          {inputFile} 4 0.9 {param} .75"
                  out = !../related_methods/GCECommunityFinder/build/GCECommunityFinder
          {inputFile} 4 0.9 {param} .75
                  if verbose:
                      print(out)
                  with open(outputFile, 'w') as the_file:
                      for line in out[out.index('Finished')+1:]:
                          the_file.write(" ".join([str(int(a)+vertexNumerationShift) for a in
          line.split()]) + "\n")

                  output=!../Overlapping-NMI/onmi {groundTruth} {outputFile}
                  if verbose:
                      print(output)
                  nmi=float(output[0].split()[1])
                  all_results[param] = nmi
                  if nmi >  nmi_best:
                      bestParam = param
                      nmi_best = nmi
              print("Best ONMI: {} params: '{}'".format(nmi_best, bestParam) )
              print("Avg ONMI: {} ".format(np.mean(list(all_results.values()))))
              return all_results
```

```
In [160]: import matplotlib.ticker as mtick
          def plot_graph_for_all_results(all_results, datasetName):
              xdata=[]
              ydata=[]
              df = pd.DataFrame()
              for param, nmi in all_results.items():
                  splited = param.split()
                  xdata.append(param)
                  ydata.append(nmi)
                  df = df.append({'fitnessExponentAlpha': float(param), "onmi value": nmi},
          ignore_index=True)

          #     plt.plot(xdata, ydata, 'C3', zorder=1, lw=3)
          #     plt.scatter(xdata, ydata,s=10,zorder=2)
              sns.scatterplot(x="fitnessExponentAlpha", y="onmi value", data=df)

              plt.xlabel('fitnessExponentAlpha')
              plt.xticks(rotation=90)
          #     plt.gca().xaxis.set_major_formatter(mtick.FormatStrFormatter('%0.0f'))
          #     plt.ylabel('onmi value');
```

```
plt.title('Algorithm: "GCE"\nDataset: {}'.format(datasetName));
plt.show()
```

2 School Friendship Network

```
In [45]: params={}
         params[""] = np.arange(0.8, 1.5, 0.025)

         inputFile = "../datasets/school_friendship/school-2.dat"
         groundTruth = "../datasets/school_friendship/truth-school.dat"
         all_results = GCE_experiment(inputFile, groundTruth, params, vertexNumerationShift=-1)
```

```
workingDir: ../Results/GCE_school_friendship
outputFile: ../Results/GCE_school_friendship/GCE_output.txt
mkdir: cannot create directory '../Results/GCE_school_friendship': File exists
```

```
HBox(children=(IntProgress(value=0, max=18), HTML(value='')))
```

```
Best ONMI: 0.868026 params: ' 0.9000000000000001'
```

```
In [46]: plot_graph_for_all_results(all_results, "school_friendship")
```

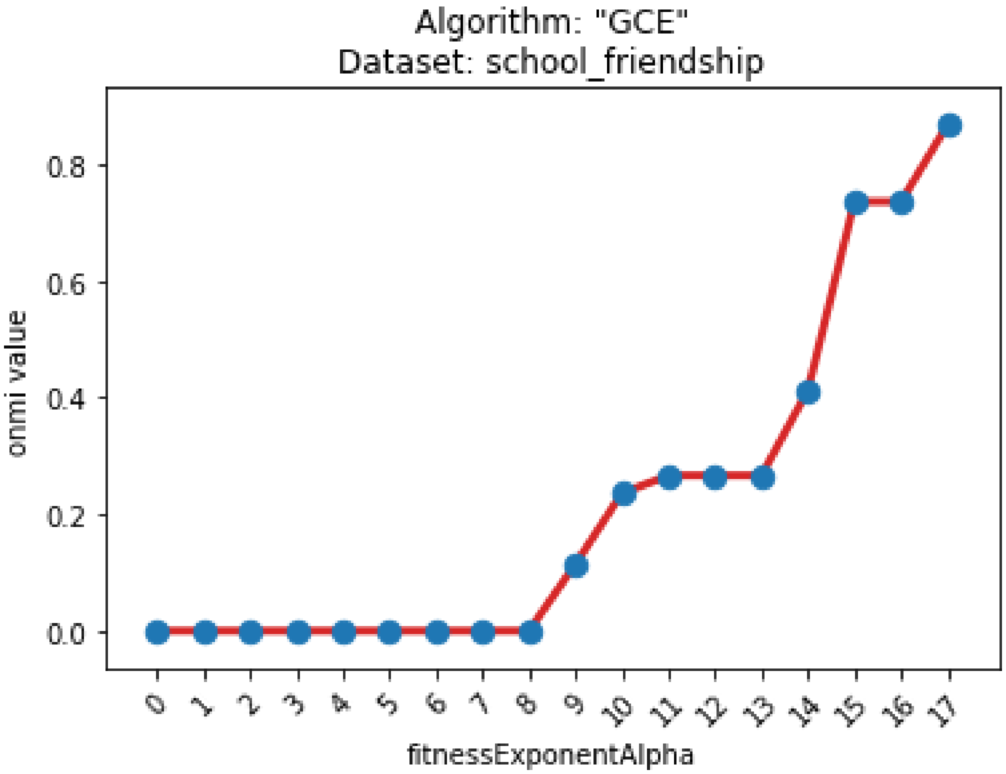

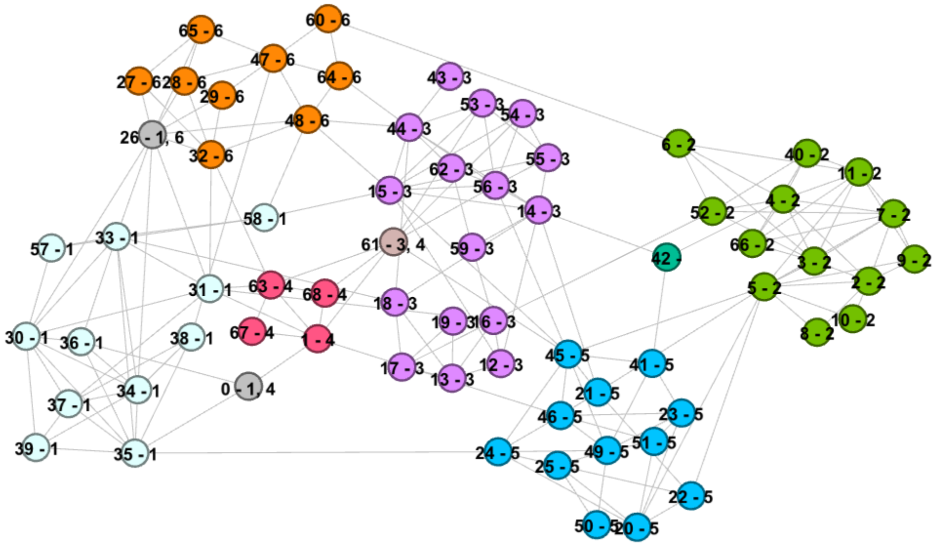

School Friendship Network. Algorithm - GCE

3 Karate Club

```
In [47]: inputFile = "../datasets/karate/karate.dat"
         groundTruth = "../datasets/karate/truth_karate.dat"
         all_results = GCE_experiment(inputFile, groundTruth, params, vertexNumerationShift=0)
```

```
workingDir: ../Results/GCE_karate
outputFile: ../Results/GCE_karate/GCE_output.txt
mkdir: cannot create directory ‘../Results/GCE_karate’: File exists
```

```
HBox(children=(IntProgress(value=0, max=18), HTML(value='')))
```

```
Best ONMI: 0.685455 params: ' 0.8'
```

```
In [48]: plot_graph_for_all_results(all_results, "Karate Club")
```

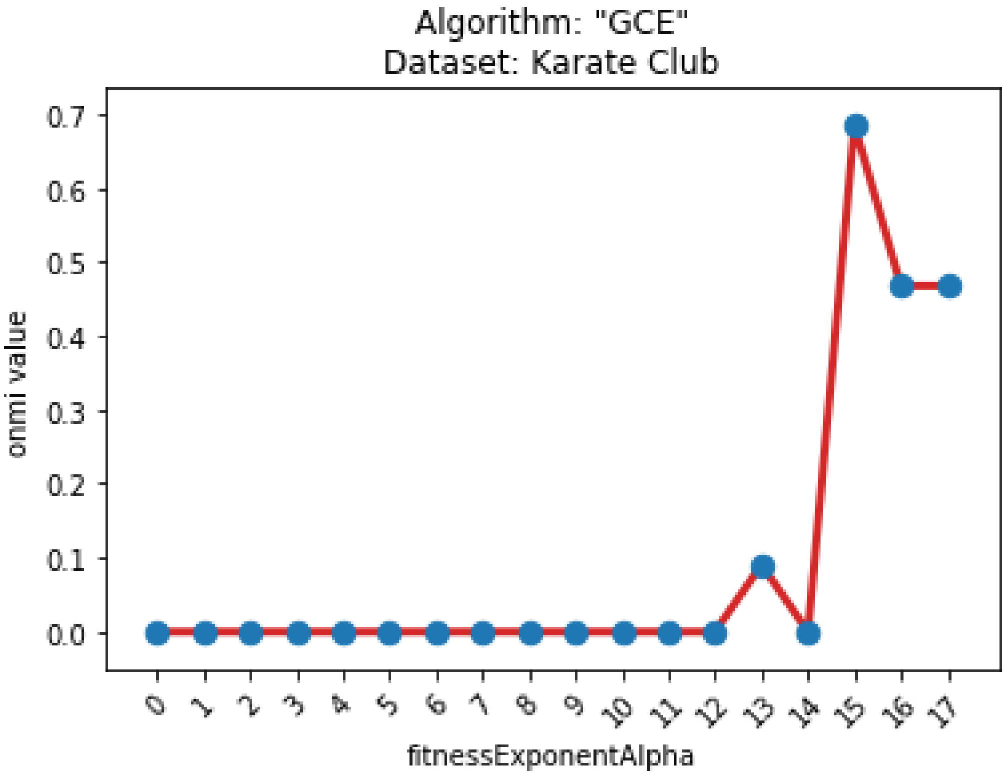

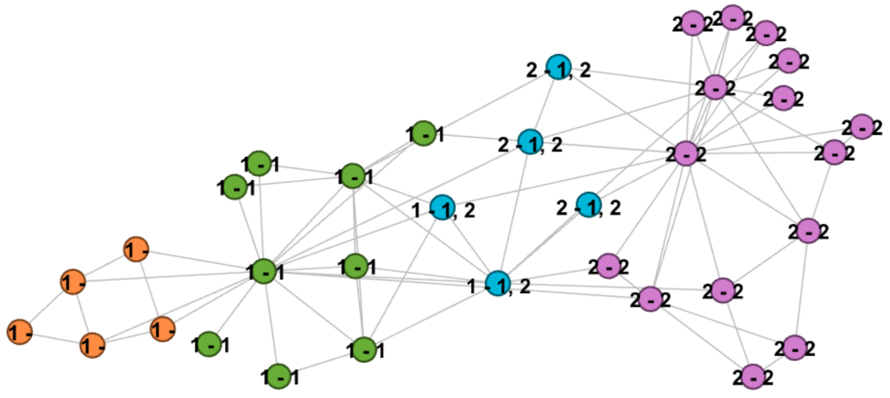

Karate Club. Algorithm - GCE

4 Adjnoun

```
In [49]: inputFile = "../datasets/adjnoun/adjnoun.dat"
         groundTruth = "../datasets/adjnoun/truth_adjnoun.dat"
         all_results = GCE_experiment(inputFile, groundTruth, params, vertexNumerationShift=-1)
```

```
workingDir: ../Results/GCE_adjnoun
outputFile: ../Results/GCE_adjnoun/GCE_output.txt
mkdir: cannot create directory ‘../Results/GCE_adjnoun’: File exists
```

```
HBox(children=(IntProgress(value=0, max=18), HTML(value='')))
```

```
Best ONMI: 0.00463105 params: ' 0.05'
```

```
In [50]: plot_graph_for_all_results(all_results, "Adj Noun")
```

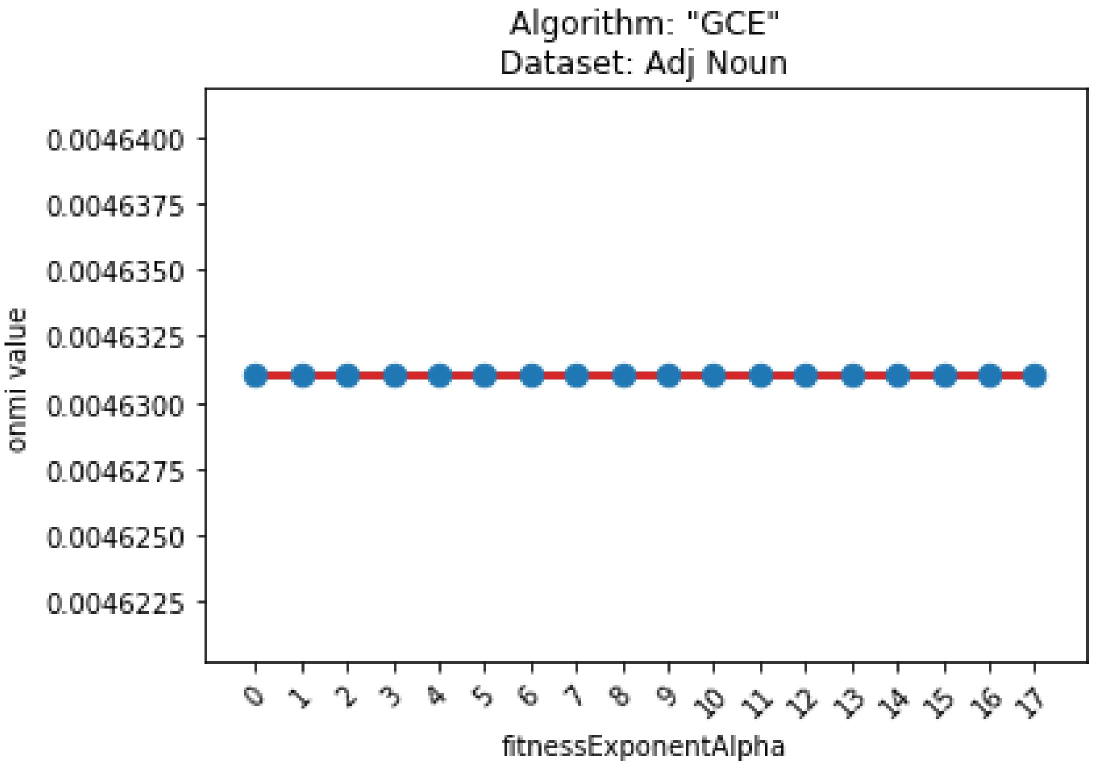

5 American Football League

```
In [52]: inputFile = "../datasets/football/footballTSEinput_original.dat"
         groundTruth = "../datasets/football/truth_footballTSEinput.dat"
         all_results = GCE_experiment(inputFile, groundTruth, params, vertexNumerationShift=-1)
```

```
workingDir: ../Results/GCE_football
outputFile: ../Results/GCE_football/GCE_output.txt
mkdir: cannot create directory '../Results/GCE_football': File exists
```

```
HBox(children=(IntProgress(value=0, max=18), HTML(value='')))
```

```
Best ONMI: 0.880363 params: ' 0.8500000000000001'
```

```
In [53]: plot_graph_for_all_results(all_results, "Football Club")
```

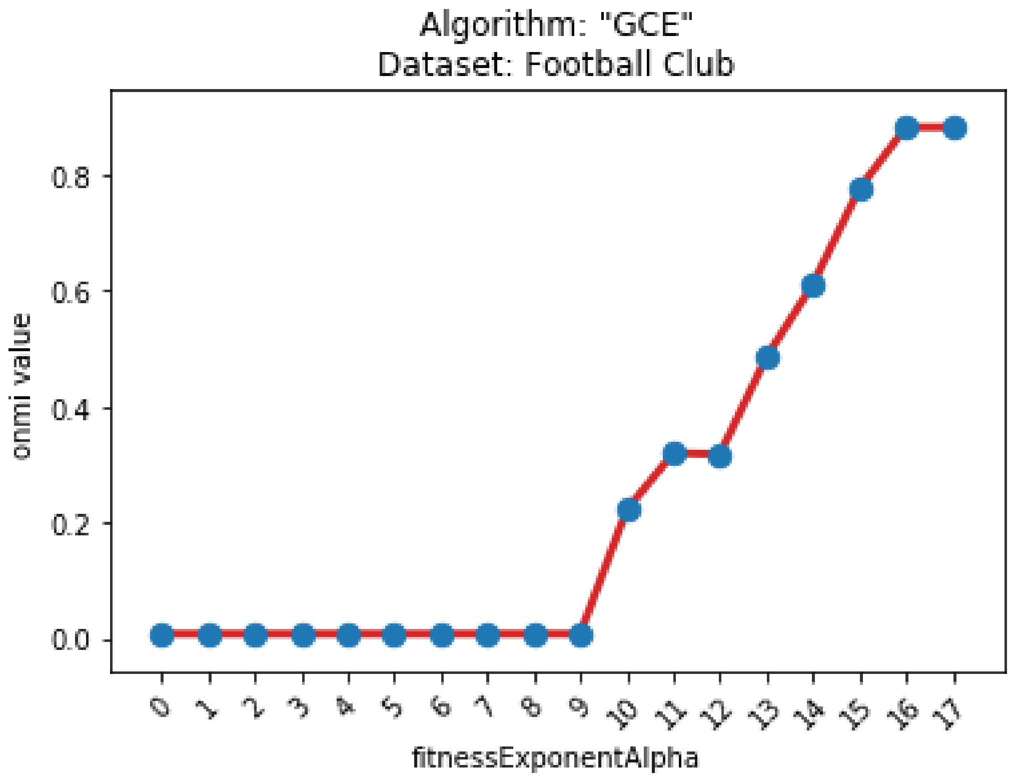

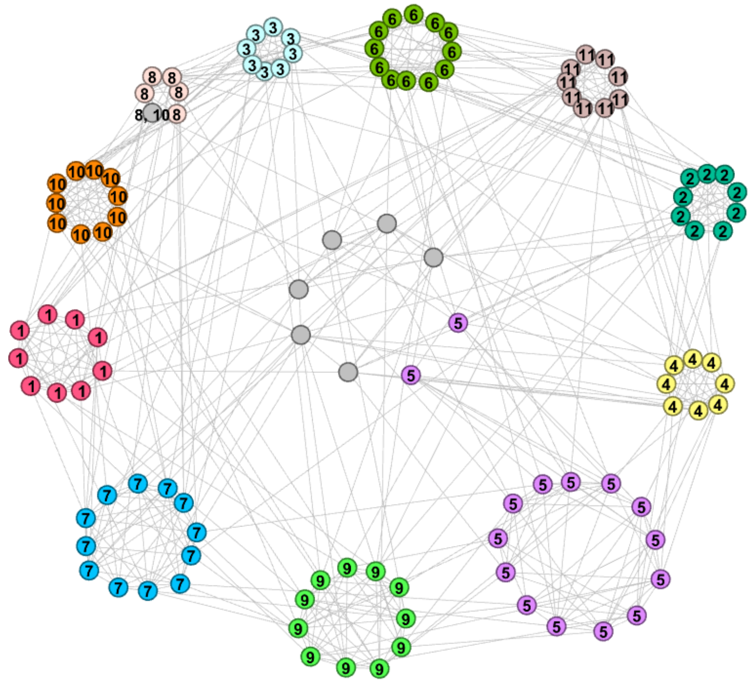

American Football League. Algorithm - GCE

6 Political Books

```
In [54]: inputFile = "../datasets/polbooks/polbooks.dat"
         groundTruth = "../datasets/polbooks/truth_polbooks.dat"
         all_results = GCE_experiment(inputFile, groundTruth, params, vertexNumerationShift=0)
```

```
workingDir: ../Results/GCE_polbooks
outputFile: ../Results/GCE_polbooks/GCE_output.txt
mkdir: cannot create directory '../Results/GCE_polbooks': File exists
```

```
HBox(children=(IntProgress(value=0, max=18), HTML(value='')))
```

```
Best ONMI: 0.434772 params: ' 0.8500000000000001'
```

```
In [55]: plot_graph_for_all_results(all_results, "Political Books")
```

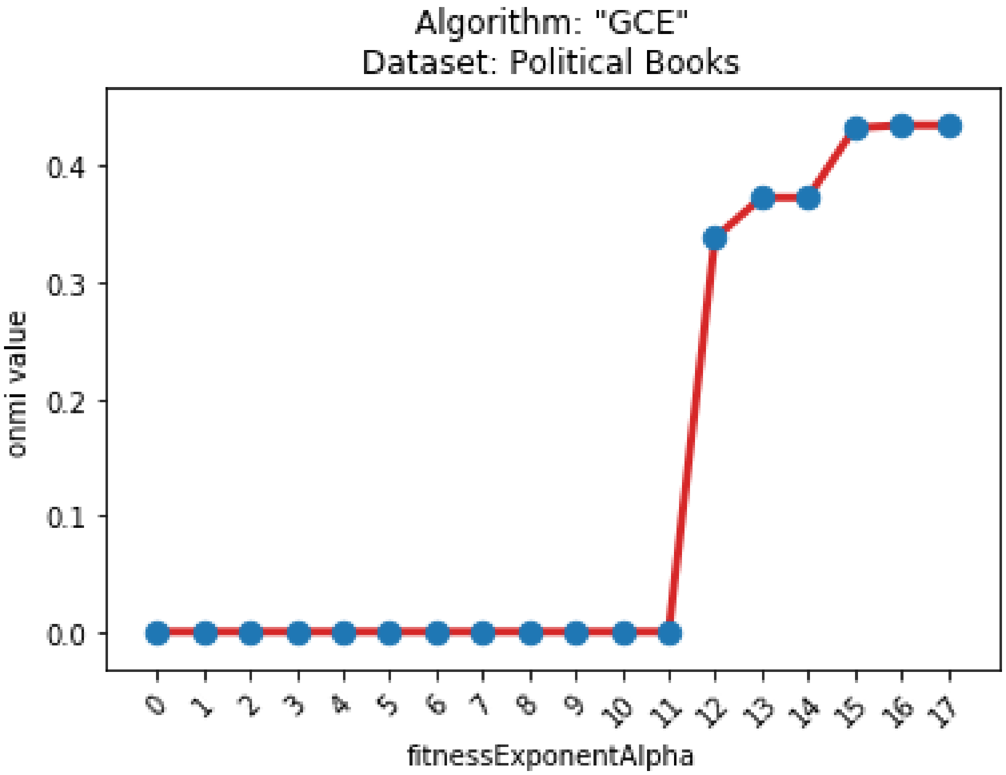

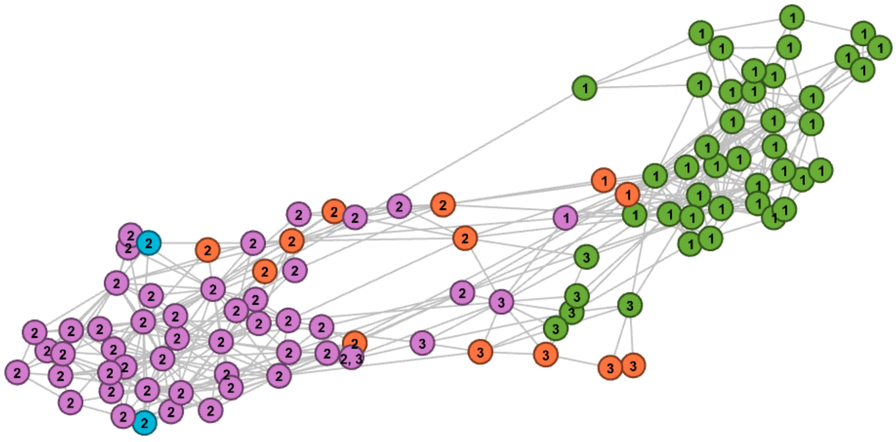

Political Books. Algorithm - GCE

7 Syntetic Datasets

```
In [35]: inputFile = "../datasets/bench_30/bench_30_network.dat"
         groundTruth = "../datasets/bench_30/bench_30_truth.dat"
         all_results = GCE_experiment(inputFile, groundTruth, params, vertexNumerationShift=0,
         verbose=False)
```

```
workingDir: ../Results/GCE_bench_30
outputFile: ../Results/GCE_bench_30/GCE_output.txt
mkdir: cannot create directory '../Results/GCE_bench_30': File exists
```

```
HBox(children=(IntProgress(value=0, max=18), HTML(value='')))
```

```
../related_methods/GCECommunityFinder/build/GCECommunityFinder
../datasets/bench_30/bench_30_network.dat 4 0.9  0.05 .75
../related_methods/GCECommunityFinder/build/GCECommunityFinder
../datasets/bench_30/bench_30_network.dat 4 0.9  0.1 .75
../related_methods/GCECommunityFinder/build/GCECommunityFinder
../datasets/bench_30/bench_30_network.dat 4 0.9  0.15000000000000002 .75
../related_methods/GCECommunityFinder/build/GCECommunityFinder
../datasets/bench_30/bench_30_network.dat 4 0.9  0.2 .75
../related_methods/GCECommunityFinder/build/GCECommunityFinder
../datasets/bench_30/bench_30_network.dat 4 0.9  0.25 .75
../related_methods/GCECommunityFinder/build/GCECommunityFinder
../datasets/bench_30/bench_30_network.dat 4 0.9  0.3 .75
../related_methods/GCECommunityFinder/build/GCECommunityFinder
../datasets/bench_30/bench_30_network.dat 4 0.9  0.35000000000000003 .75
../related_methods/GCECommunityFinder/build/GCECommunityFinder
../datasets/bench_30/bench_30_network.dat 4 0.9  0.4 .75
../related_methods/GCECommunityFinder/build/GCECommunityFinder
../datasets/bench_30/bench_30_network.dat 4 0.9  0.45 .75
../related_methods/GCECommunityFinder/build/GCECommunityFinder
../datasets/bench_30/bench_30_network.dat 4 0.9  0.5 .75
../related_methods/GCECommunityFinder/build/GCECommunityFinder
../datasets/bench_30/bench_30_network.dat 4 0.9  0.55 .75
../related_methods/GCECommunityFinder/build/GCECommunityFinder
../datasets/bench_30/bench_30_network.dat 4 0.9  0.6000000000000001 .75
../related_methods/GCECommunityFinder/build/GCECommunityFinder
../datasets/bench_30/bench_30_network.dat 4 0.9  0.6500000000000001 .75
../related_methods/GCECommunityFinder/build/GCECommunityFinder
../datasets/bench_30/bench_30_network.dat 4 0.9  0.7000000000000001 .75
../related_methods/GCECommunityFinder/build/GCECommunityFinder
../datasets/bench_30/bench_30_network.dat 4 0.9  0.7500000000000001 .75
../related_methods/GCECommunityFinder/build/GCECommunityFinder
../datasets/bench_30/bench_30_network.dat 4 0.9  0.8 .75
../related_methods/GCECommunityFinder/build/GCECommunityFinder
../datasets/bench_30/bench_30_network.dat 4 0.9  0.8500000000000001 .75
../related_methods/GCECommunityFinder/build/GCECommunityFinder
../datasets/bench_30/bench_30_network.dat 4 0.9  0.9000000000000001 .75
```

```
Best ONMI: 0.348233 params: ' 0.8'
```

7.1 bench_30

```
In [56]: inputFile = "../datasets/bench_30/bench_30_network.dat"
         groundTruth = "../datasets/bench_30/bench_30_truth.dat"
```

```
all_results = GCE_experiment(inputFile, groundTruth, params, vertexNumerationShift=0,
verbose=False)
```

```
workingDir: ../Results/GCE_bench_30
outputFile: ../Results/GCE_bench_30/GCE_output.txt
mkdir: cannot create directory '../Results/GCE_bench_30': File exists
```

```
HBox(children=(IntProgress(value=0, max=18), HTML(value='')))
```

```
Best ONMI: 0.348233 params: ' 0.8'
```

```
In [57]: plot_graph_for_all_results(all_results, "bench_30_network")
```

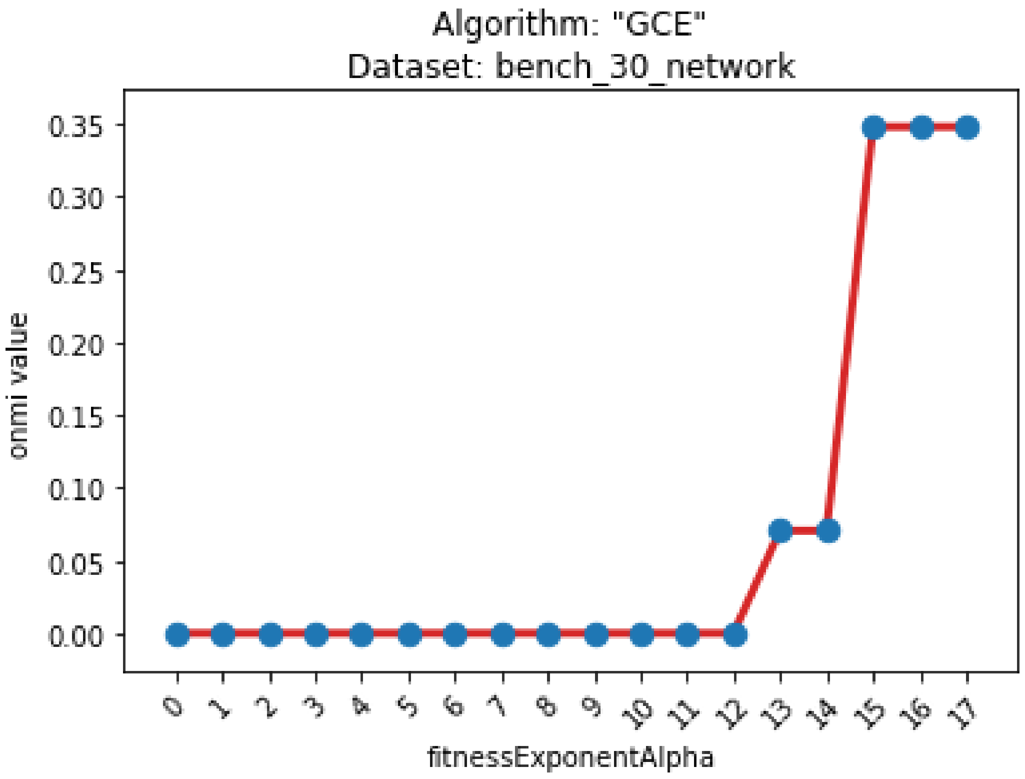

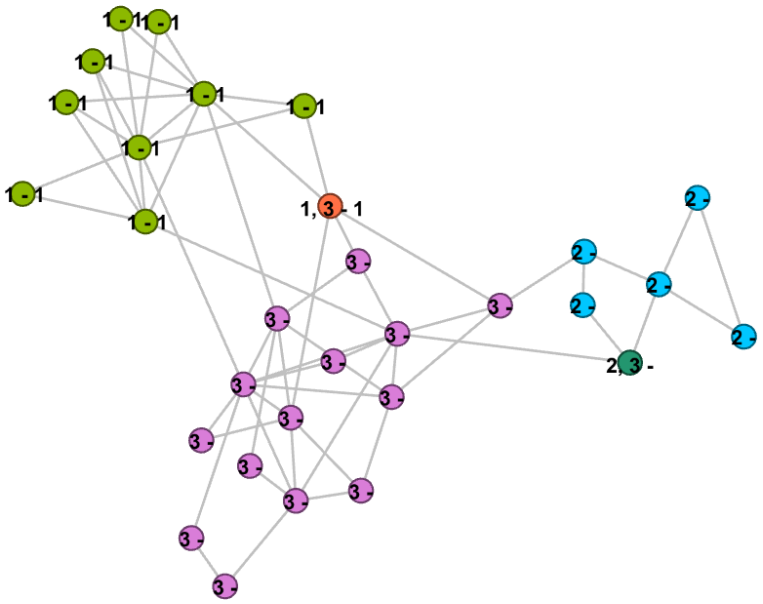

Bench_30. Algorithm - GCE

bench_40

```
In [58]: inputFile = "../datasets/bench_40/bench_40_network.dat"
         groundTruth = "../datasets/bench_40/bench_40_truth.dat"
         all_results = GCE_experiment(inputFile, groundTruth, params, vertexNumerationShift=0,
         verbose=False)
```

```
workingDir: ../Results/GCE_bench_40
outputFile: ../Results/GCE_bench_40/GCE_output.txt
mkdir: cannot create directory ‘../Results/GCE_bench_40’: File exists
```

```
HBox(children=(IntProgress(value=0, max=18), HTML(value='')))
```

```
Best ONMI: 0.0969355 params: ' 0.8500000000000001'
```

```
In [59]: plot_graph_for_all_results(all_results, "bench_40_network")
```

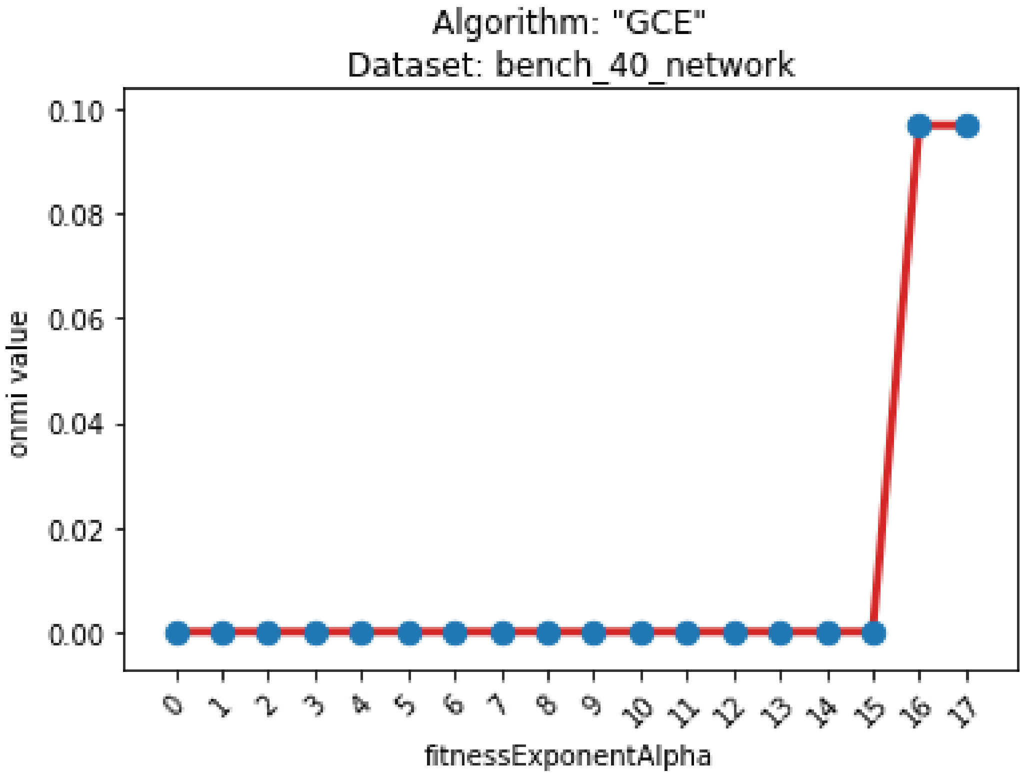

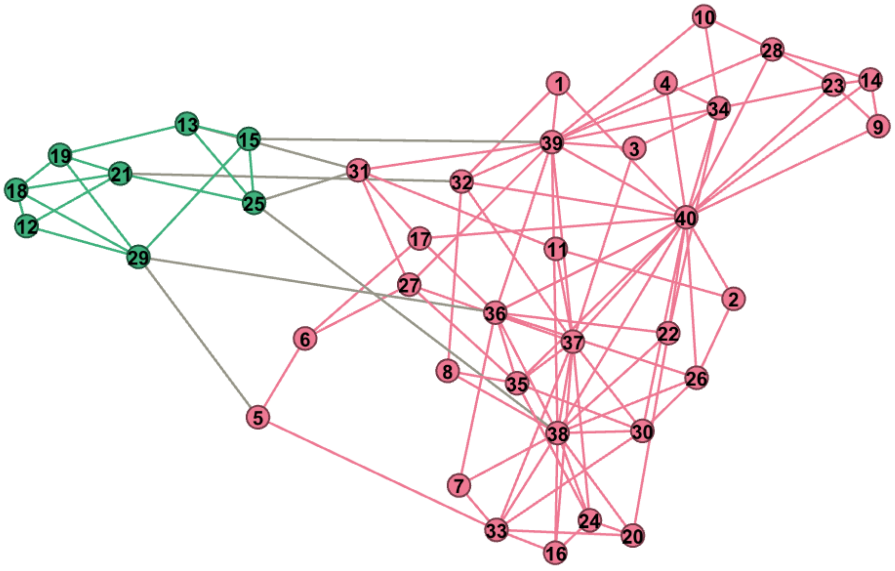

Bench_40. Algorithm - GCE

7.2 bench_50

```
In [60]: inputFile = "../datasets/bench_50/bench_50_network.dat"
         groundTruth = "../datasets/bench_50/bench_50_truth.dat"
         all_results = GCE_experiment(inputFile, groundTruth, params, vertexNumerationShift=0,
         verbose=False)
```

```
workingDir: ../Results/GCE_bench_50
outputFile: ../Results/GCE_bench_50/GCE_output.txt
mkdir: cannot create directory '../Results/GCE_bench_50': File exists
```

```
HBox(children=(IntProgress(value=0, max=18), HTML(value='')))
```

```
Best ONMI: 0.392884 params: ' 0.9000000000000001'
```

```
In [61]: plot_graph_for_all_results(all_results, "bench_50_network")
```

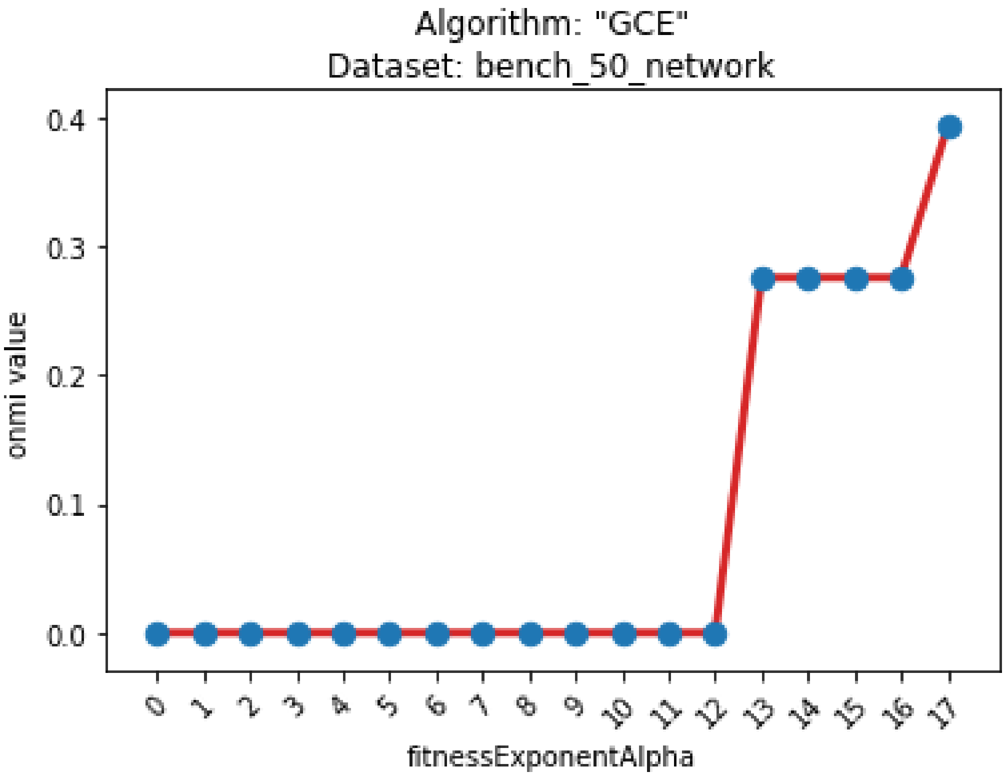

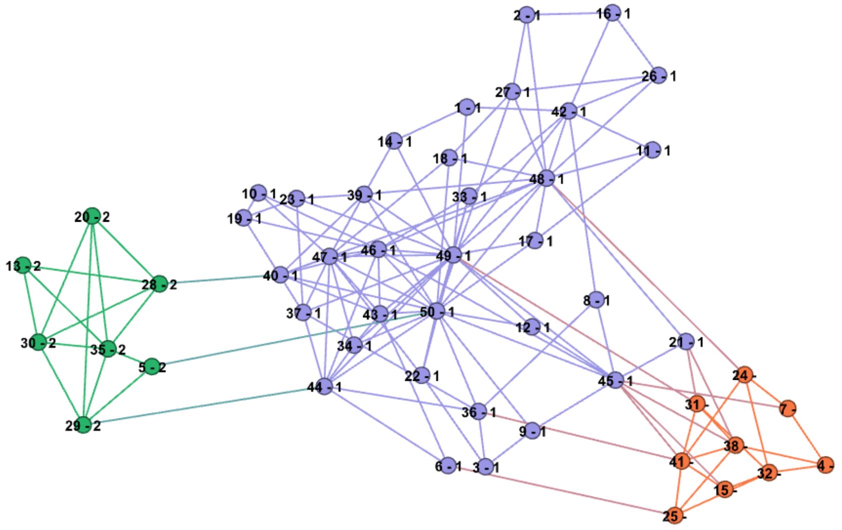

gce_bench_50

7.3 bench_60

```
In [62]: inputFile = "../datasets/bench_60/bench_60_network.dat"
         groundTruth = "../datasets/bench_60/bench_60_truth.dat"
         all_results = GCE_experiment(inputFile, groundTruth, params, vertexNumerationShift=0,
         verbose=False)
```

```
workingDir: ../Results/GCE_bench_60
outputFile: ../Results/GCE_bench_60/GCE_output.txt
mkdir: cannot create directory ‘../Results/GCE_bench_60’: File exists
```

```
HBox(children=(IntProgress(value=0, max=18), HTML(value='')))
```

```
Best ONMI: 0.257251 params: ' 0.7000000000000001'
```

```
In [63]: plot_graph_for_all_results(all_results, "bench_60_network")
```

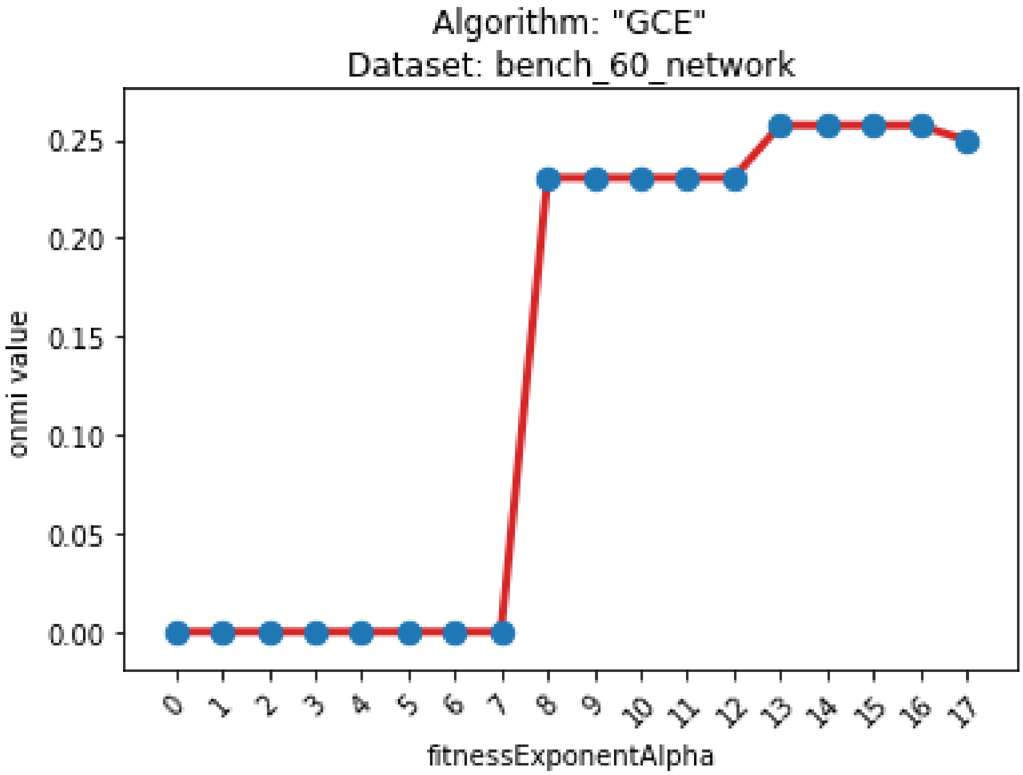

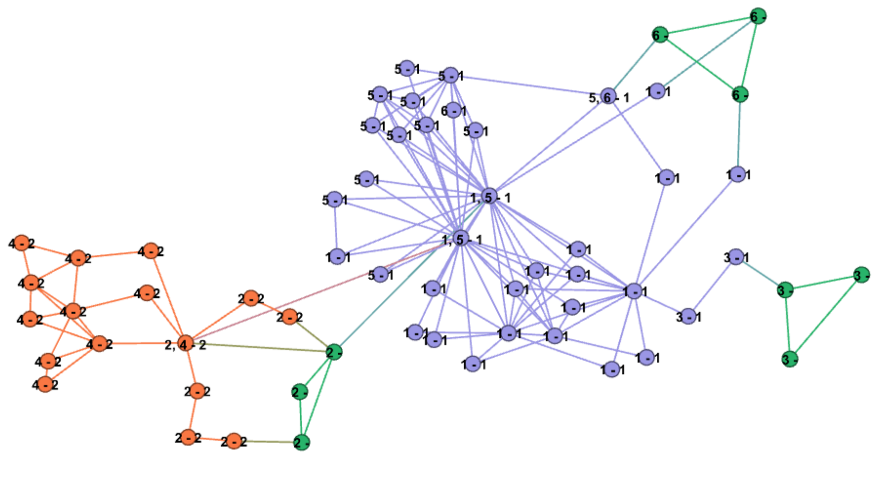

Bench_60. Algorithm - GCE

7.4 bench_60_dense

```
In [64]: inputFile = "../datasets/bench_60_dense/bench_60_dense_network.dat"
         groundTruth = "../datasets/bench_60_dense/bench_60_dense_truth.dat"
         all_results = GCE_experiment(inputFile, groundTruth, params, vertexNumerationShift=0,
         verbose=False)
```

```
workingDir: ../Results/GCE_bench_60_dense
outputFile: ../Results/GCE_bench_60_dense/GCE_output.txt
mkdir: cannot create directory '../Results/GCE_bench_60_dense': File exists
```

```
HBox(children=(IntProgress(value=0, max=18), HTML(value='')))
```

```
Best ONMI: 0.156888 params: ' 0.9000000000000001'
```

```
In [65]: plot_graph_for_all_results(all_results, "bench_60_dense_network")
```

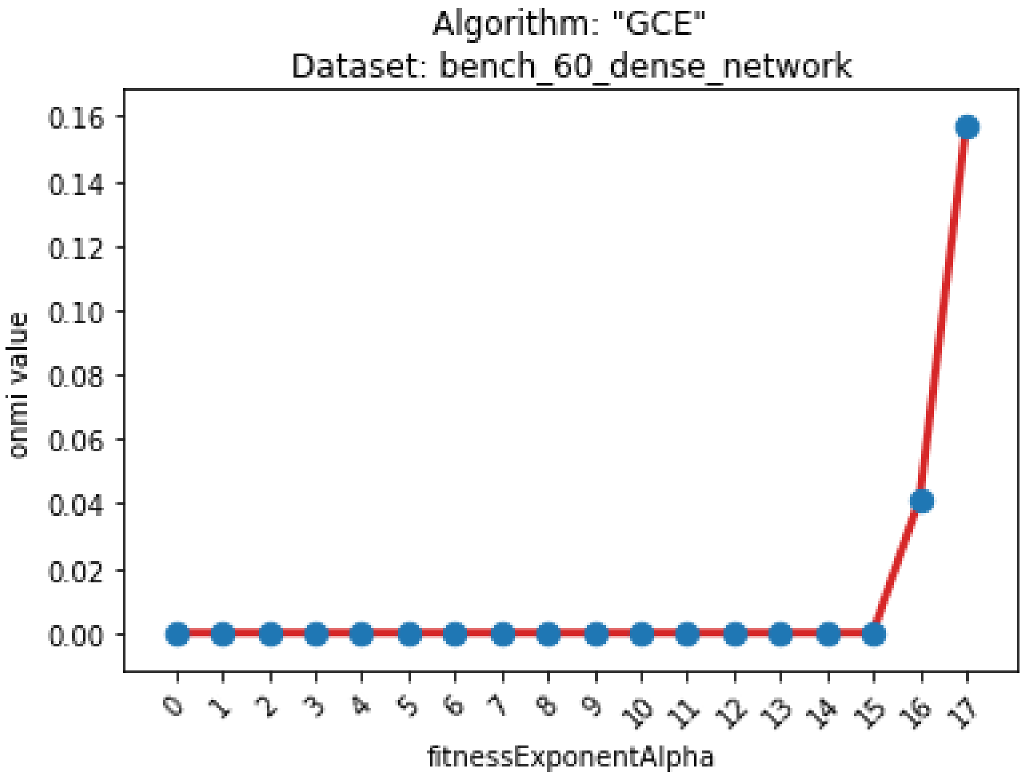

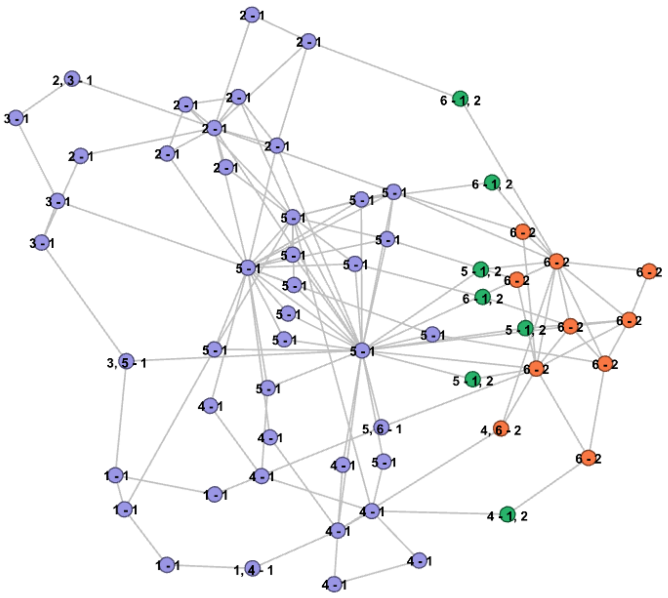

Bench_60_dense. Algorithm - GCE

```
In [21]: !../related_methods/GCECommunityFinder/build/GCECommunityFinder
         "../datasets/lattice_8x8/lattice_8x8.dat" 4 0.9 0.9 .05
```

```
Greedy Clique Expansion Community Finder
Running with parameters: k: 4 eta: 0.9 alpha: 0.9 Phi: 0.05
Loading file: ../datasets/lattice_8x8/lattice_8x8.dat
bg.ecount():112
bg.vcount():64
bg.edge_targets.size():224
bg.degrees.size():64
bg.offsets.size():65
Loaded : 0 cliques
Cliques sorted. About to run spokenFor pruning...number of seeds before: 0
Sweeping the seeds marked for deletion
Done
SpokenFor complete. Number of seeds after: 0
0.00s: Total number of seeds remaining: 0
Edges in loaded graph:  112
Nodes in loaded graph:  64
0.00s: 0.00s: --------------------------------
Number of seeds: 0
0.00s: Number seeds discarded before expansion: 0 seeds
Number seeds discarded after expansion: 0 seeds
Number seeds kept: 0 seeds
Sweeping the seeds marked for deletion
Done
Finished
```

```
In [ ]: !java -jar ../CommunityVisualizer/target/CommunityVisualizer-1.0-SNAPSHOT.jar
        {inputFile} {}
```

8 FARZ

8.1 FARZ_n_200_m_5_k_5_beta_1

In [164]:
```
params={}
params[""] = np.arange(0.8, 1.5, 0.025)
inputFile = "../datasets/FARZ_n_200_m_5_k_5_beta_1/network.dat"
groundTruth = "../datasets/FARZ_n_200_m_5_k_5_beta_1/network.lgt"
all_results = GCE_experiment(inputFile, groundTruth, params, vertexNumerationShift=0,
verbose=False)
```

```
workingDir: ../Results/GCE_FARZ_n_200_m_5_k_5_beta_1
outputFile: ../Results/GCE_FARZ_n_200_m_5_k_5_beta_1/GCE_output.txt
mkdir: cannot create directory ‘../Results/GCE_FARZ_n_200_m_5_k_5_beta_1’: File exists
```

```
HBox(children=(IntProgress(value=0, max=28), HTML(value='')))
```

```
Best ONMI: 0.951262 params: ' 0.8'
Avg ONMI: 0.7384528214285713
```

In [165]:
```
plot_graph_for_all_results(all_results, "FARZ_n_200_m_5_k_5_beta_1")
```

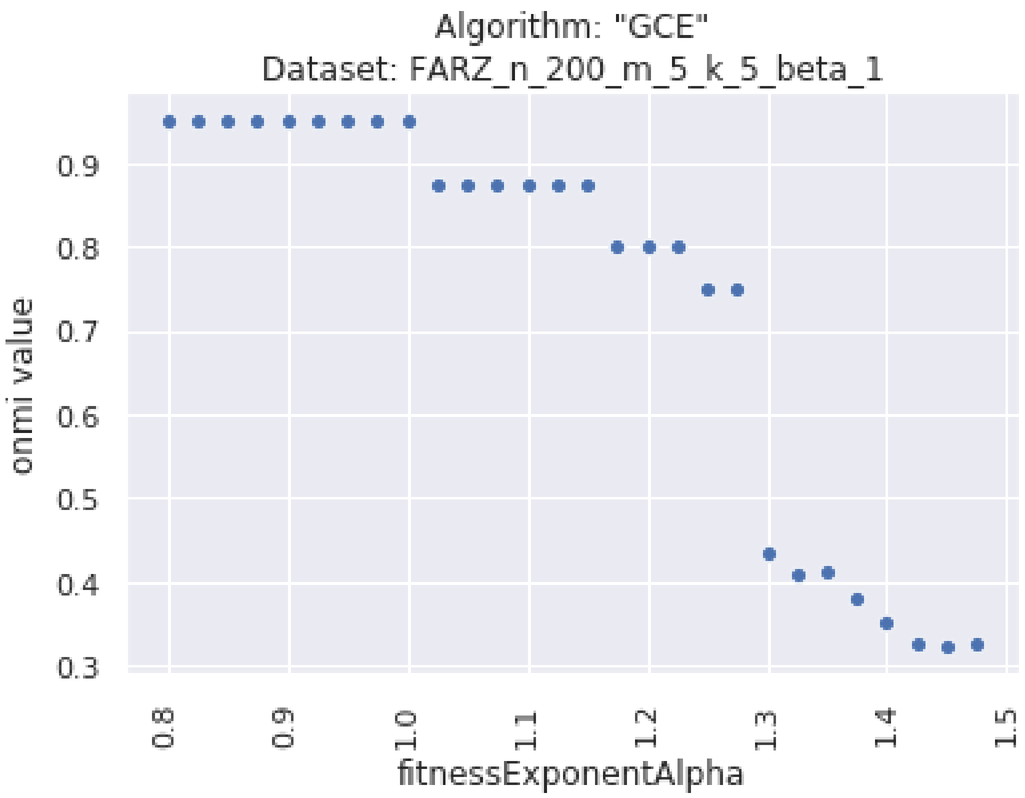

8.2 FARZ_n_200_m_5_k_5_beta_0.95

```
In [166]: params={}
          params[""] = np.arange(0.8, 1.5, 0.025)
          inputFile = "../datasets/FARZ_n_200_m_5_k_5_beta_0.95/network.dat"
          groundTruth = "../datasets/FARZ_n_200_m_5_k_5_beta_0.95/network.lgt"
          all_results = GCE_experiment(inputFile, groundTruth, params, vertexNumerationShift=0,
          verbose=False)
```

```
workingDir: ../Results/GCE_FARZ_n_200_m_5_k_5_beta_0.95
outputFile: ../Results/GCE_FARZ_n_200_m_5_k_5_beta_0.95/GCE_output.txt
mkdir: cannot create directory '../Results/GCE_FARZ_n_200_m_5_k_5_beta_0.95': File
exists
```

```
HBox(children=(IntProgress(value=0, max=28), HTML(value='')))
```

```
Best ONMI: 0.887785 params: ' 0.8'
Avg ONMI: 0.5970368928571429
```

```
In [167]: plot_graph_for_all_results(all_results, "FARZ_n_200_m_5_k_5_beta_0.95")
```

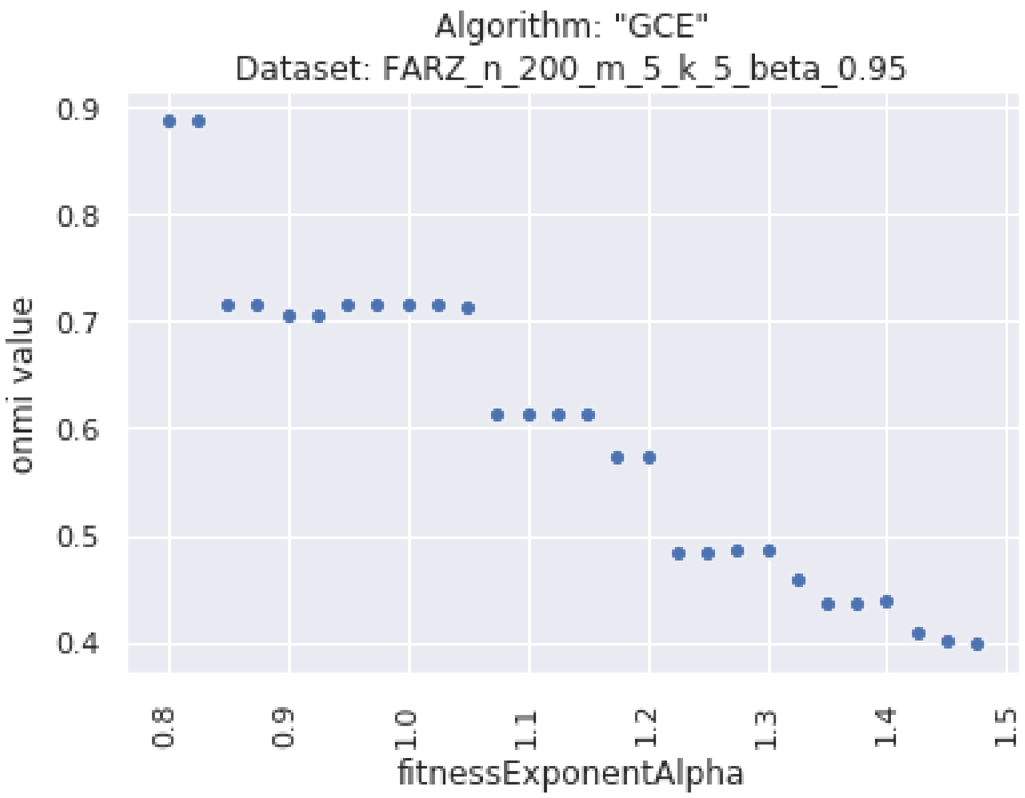

8.3 FARZ_n_200_m_5_k_5_beta_0.9

```
In [168]: params={}
          params[""] = np.arange(0.8, 1.5, 0.025)
          inputFile = "../datasets/FARZ_n_200_m_5_k_5_beta_0.9/network.dat"
          groundTruth = "../datasets/FARZ_n_200_m_5_k_5_beta_0.9/network.lgt"
          all_results = GCE_experiment(inputFile, groundTruth, params, vertexNumerationShift=0,
          verbose=False)
```

```
workingDir: ../Results/GCE_FARZ_n_200_m_5_k_5_beta_0.9
outputFile: ../Results/GCE_FARZ_n_200_m_5_k_5_beta_0.9/GCE_output.txt
mkdir: cannot create directory '../Results/GCE_FARZ_n_200_m_5_k_5_beta_0.9': File
exists
```

```
HBox(children=(IntProgress(value=0, max=28), HTML(value='')))
```

```
Best ONMI: 0.817856 params: ' 0.9000000000000001'
Avg ONMI: 0.6797227499999999
```

```
In [169]: plot_graph_for_all_results(all_results, "FARZ_n_200_m_5_k_5_beta_0.9")
```

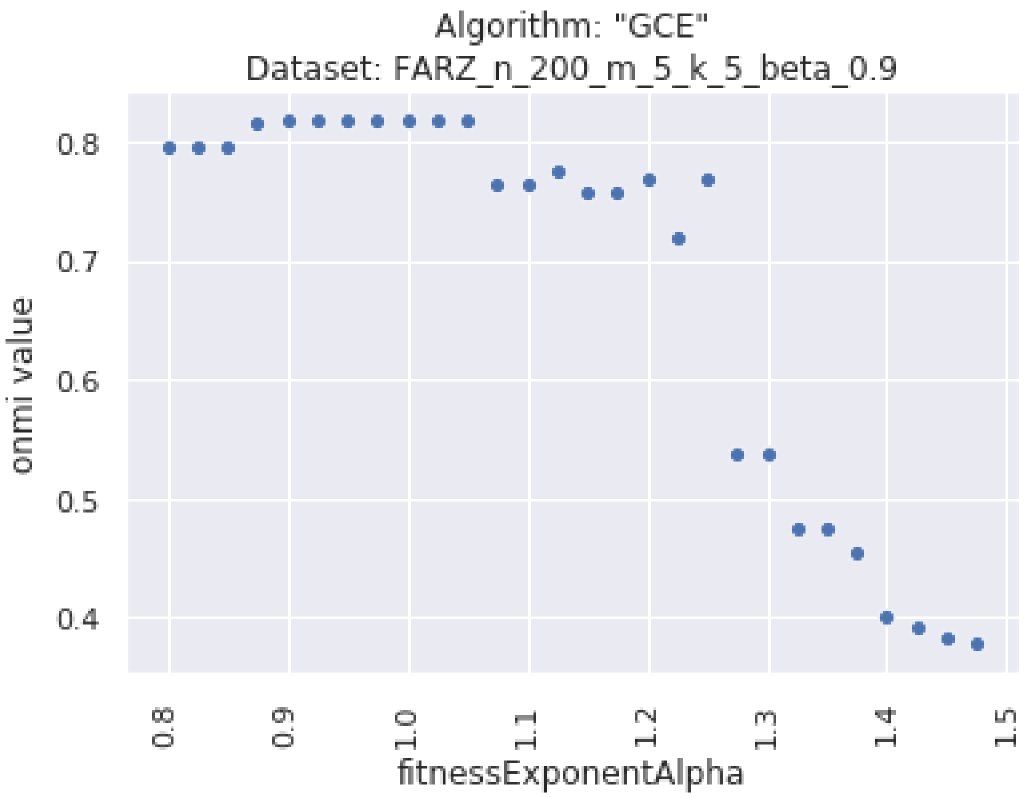

8.4 FARZ_n_200_m_5_k_5_beta_0.85

```
In [170]: params={}
          params[""] =  np.arange(0.8, 1.5, 0.025)
          inputFile = "../datasets/FARZ_n_200_m_5_k_5_beta_0.85/network.dat"
          groundTruth = "../datasets/FARZ_n_200_m_5_k_5_beta_0.85/network.lgt"
          all_results = GCE_experiment(inputFile, groundTruth, params, vertexNumerationShift=0,
          verbose=False)
```

```
workingDir: ../Results/GCE_FARZ_n_200_m_5_k_5_beta_0.85
outputFile: ../Results/GCE_FARZ_n_200_m_5_k_5_beta_0.85/GCE_output.txt
mkdir: cannot create directory ‘../Results/GCE_FARZ_n_200_m_5_k_5_beta_0.85’: File
exists
```

```
HBox(children=(IntProgress(value=0, max=28), HTML(value='')))
```

```
Best ONMI: 0.847496 params: ' 0.9000000000000001'
Avg ONMI: 0.5696628214285714
```

```
In [171]: plot_graph_for_all_results(all_results, "FARZ_n_200_m_5_k_5_beta_0.85")
```

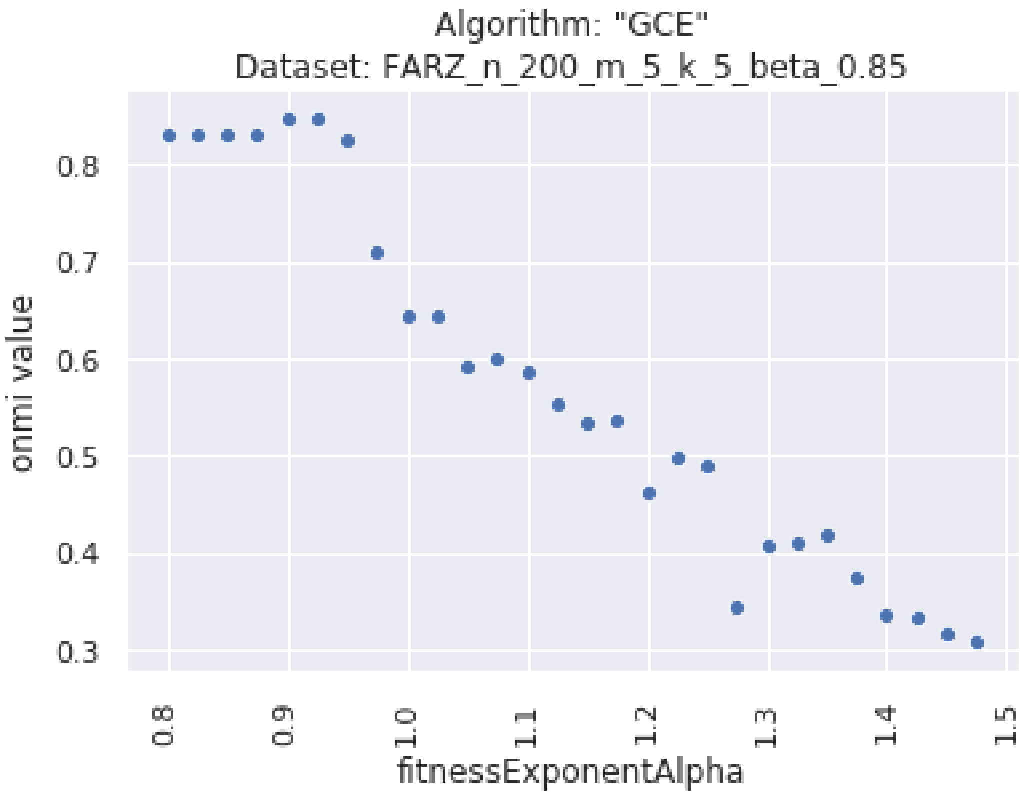

8.5 FARZ_n_200_m_5_k_5_beta_0.8

In [172]:
```
params={}
params[""] = np.arange(0.8, 1.5, 0.025)
inputFile = "../datasets/FARZ_n_200_m_5_k_5_beta_0.8/network.dat"
groundTruth = "../datasets/FARZ_n_200_m_5_k_5_beta_0.8/network.lgt"
all_results = GCE_experiment(inputFile, groundTruth, params, vertexNumerationShift=0,
verbose=False)
```

```
workingDir: ../Results/GCE_FARZ_n_200_m_5_k_5_beta_0.8
outputFile: ../Results/GCE_FARZ_n_200_m_5_k_5_beta_0.8/GCE_output.txt
mkdir: cannot create directory ‘../Results/GCE_FARZ_n_200_m_5_k_5_beta_0.8’: File
exists
```

```
HBox(children=(IntProgress(value=0, max=28), HTML(value='')))
```

```
Best ONMI: 0.722108 params: ' 0.8750000000000001'
Avg ONMI: 0.4424587142857143
```

In [173]:
```
plot_graph_for_all_results(all_results, "FARZ_n_200_m_5_k_5_beta_0.8")
```

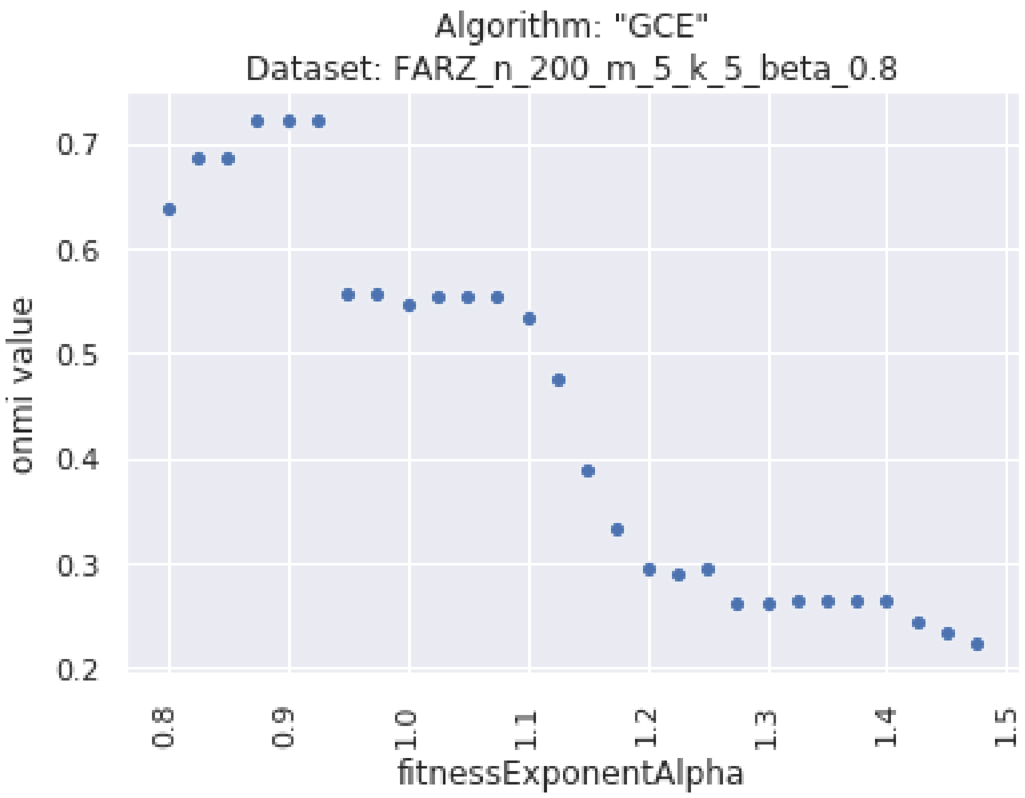

8.6 FARZ_n_200_m_5_k_5_beta_0.75

```
In [174]: params={}
          params[""] = np.arange(0.8, 1.5, 0.025)
          inputFile = "../datasets/FARZ_n_200_m_5_k_5_beta_0.75/network.dat"
          groundTruth = "../datasets/FARZ_n_200_m_5_k_5_beta_0.75/network.lgt"
          all_results = GCE_experiment(inputFile, groundTruth, params, vertexNumerationShift=0,
          verbose=False)
```

```
workingDir: ../Results/GCE_FARZ_n_200_m_5_k_5_beta_0.75
outputFile: ../Results/GCE_FARZ_n_200_m_5_k_5_beta_0.75/GCE_output.txt
mkdir: cannot create directory '../Results/GCE_FARZ_n_200_m_5_k_5_beta_0.75': File
exists
```

```
HBox(children=(IntProgress(value=0, max=28), HTML(value='')))
```

```
Best ONMI: 0.692143 params: ' 0.9250000000000002'
Avg ONMI: 0.5824832142857144
```

```
In [175]: plot_graph_for_all_results(all_results, "FARZ_n_200_m_5_k_5_beta_0.75")
```

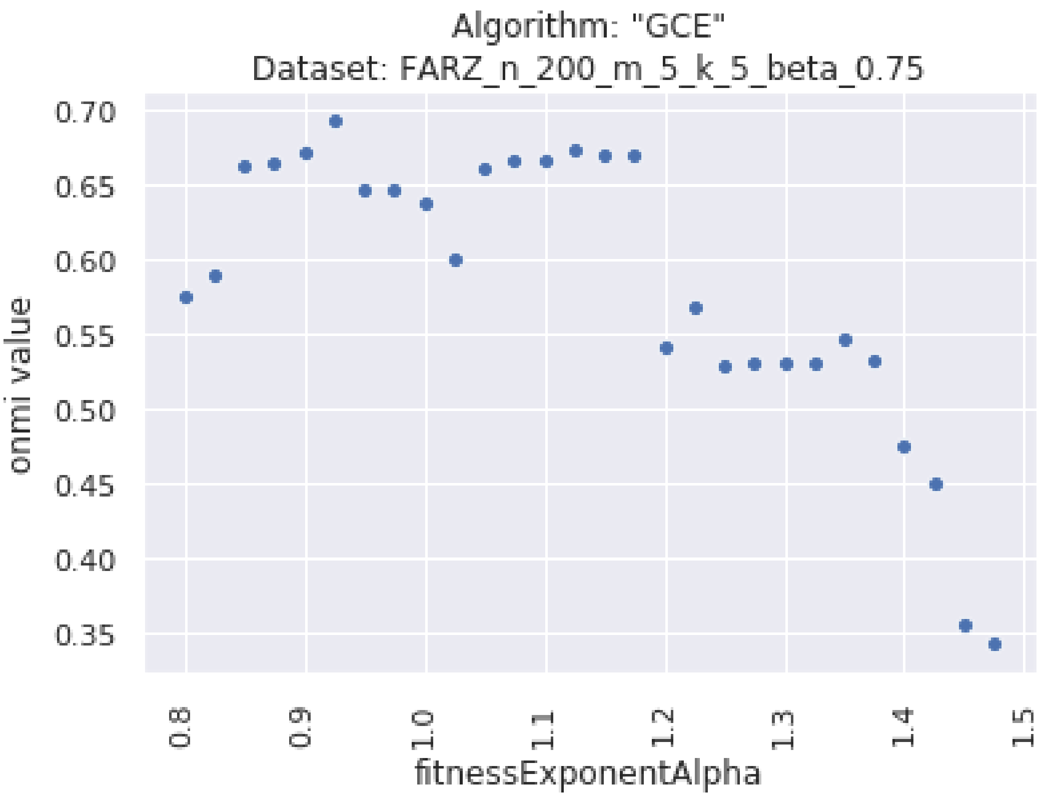

8.7 FARZ_n_200_m_5_k_5_beta_0.7

```
In [176]: params={}
          params[""] = np.arange(0.8, 1.5, 0.025)
          inputFile = "../datasets/FARZ_n_200_m_5_k_5_beta_0.7/network.dat"
          groundTruth = "../datasets/FARZ_n_200_m_5_k_5_beta_0.7/network.lgt"
          all_results = GCE_experiment(inputFile, groundTruth, params, vertexNumerationShift=0,
          verbose=False)
```

```
workingDir: ../Results/GCE_FARZ_n_200_m_5_k_5_beta_0.7
outputFile: ../Results/GCE_FARZ_n_200_m_5_k_5_beta_0.7/GCE_output.txt
mkdir: cannot create directory '../Results/GCE_FARZ_n_200_m_5_k_5_beta_0.7': File
exists
```

```
HBox(children=(IntProgress(value=0, max=28), HTML(value='')))
```

```
Best ONMI: 0.669042 params: ' 0.8250000000000001'
Avg ONMI: 0.4510196071428571
```

```
In [105]: plot_graph_for_all_results(all_results, "FARZ_n_200_m_5_k_5_beta_0.7")
```

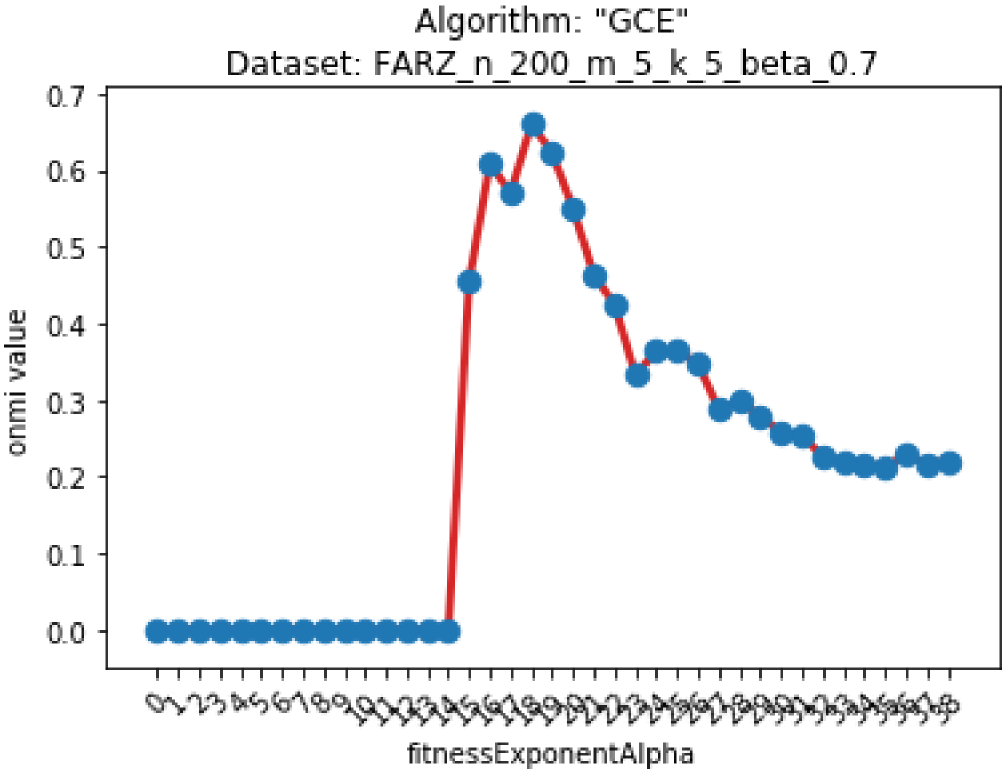

8.8 FARZ_n_200_m_5_k_5_beta_0.65

```
In [177]: params={}
          params[""] = np.arange(0.8, 1.5, 0.025)
          inputFile = "../datasets/FARZ_n_200_m_5_k_5_beta_0.65/network.dat"
          groundTruth = "../datasets/FARZ_n_200_m_5_k_5_beta_0.65/network.lgt"
          all_results = GCE_experiment(inputFile, groundTruth, params, vertexNumerationShift=0,
          verbose=False)
```

```
workingDir: ../Results/GCE_FARZ_n_200_m_5_k_5_beta_0.65
outputFile: ../Results/GCE_FARZ_n_200_m_5_k_5_beta_0.65/GCE_output.txt
mkdir: cannot create directory '../Results/GCE_FARZ_n_200_m_5_k_5_beta_0.65': File
exists
```

```
HBox(children=(IntProgress(value=0, max=28), HTML(value='')))
```

```
Best ONMI: 0.477405 params: ' 1.0250000000000004'
Avg ONMI: 0.37502714285714284
```

```
In [178]: plot_graph_for_all_results(all_results, "FARZ_n_200_m_5_k_5_beta_0.65")
```

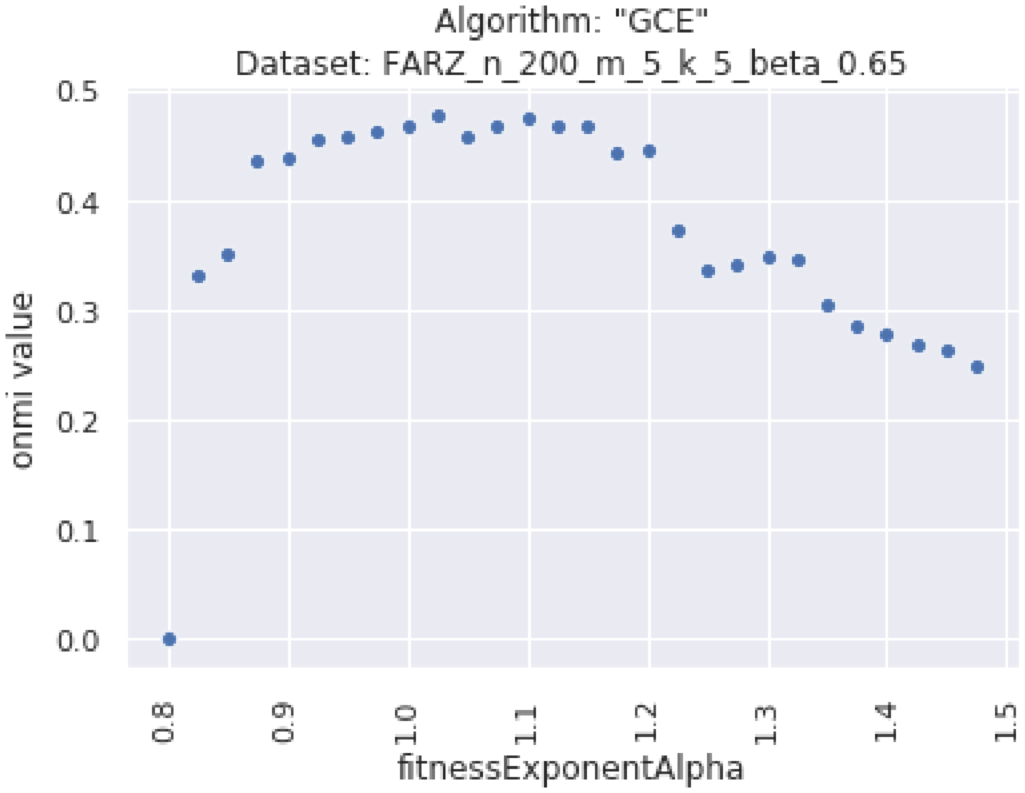

8.9 FARZ_n_200_m_5_k_5_beta_0.6

In [179]:
```
params={}
params[""] = np.arange(0.8, 1.5, 0.025)
inputFile = "../datasets/FARZ_n_200_m_5_k_5_beta_0.6/network.dat"
groundTruth = "../datasets/FARZ_n_200_m_5_k_5_beta_0.6/network.lgt"
all_results = GCE_experiment(inputFile, groundTruth, params, vertexNumerationShift=0,
verbose=False)
```

```
workingDir: ../Results/GCE_FARZ_n_200_m_5_k_5_beta_0.6
outputFile: ../Results/GCE_FARZ_n_200_m_5_k_5_beta_0.6/GCE_output.txt
mkdir: cannot create directory '../Results/GCE_FARZ_n_200_m_5_k_5_beta_0.6': File
exists
```

```
HBox(children=(IntProgress(value=0, max=28), HTML(value='')))
```

```
Best ONMI: 0.33426 params: ' 1.0500000000000003'
Avg ONMI: 0.1883288571428571
```

In [180]:
```
plot_graph_for_all_results(all_results, "FARZ_n_200_m_5_k_5_beta_0.6")
```

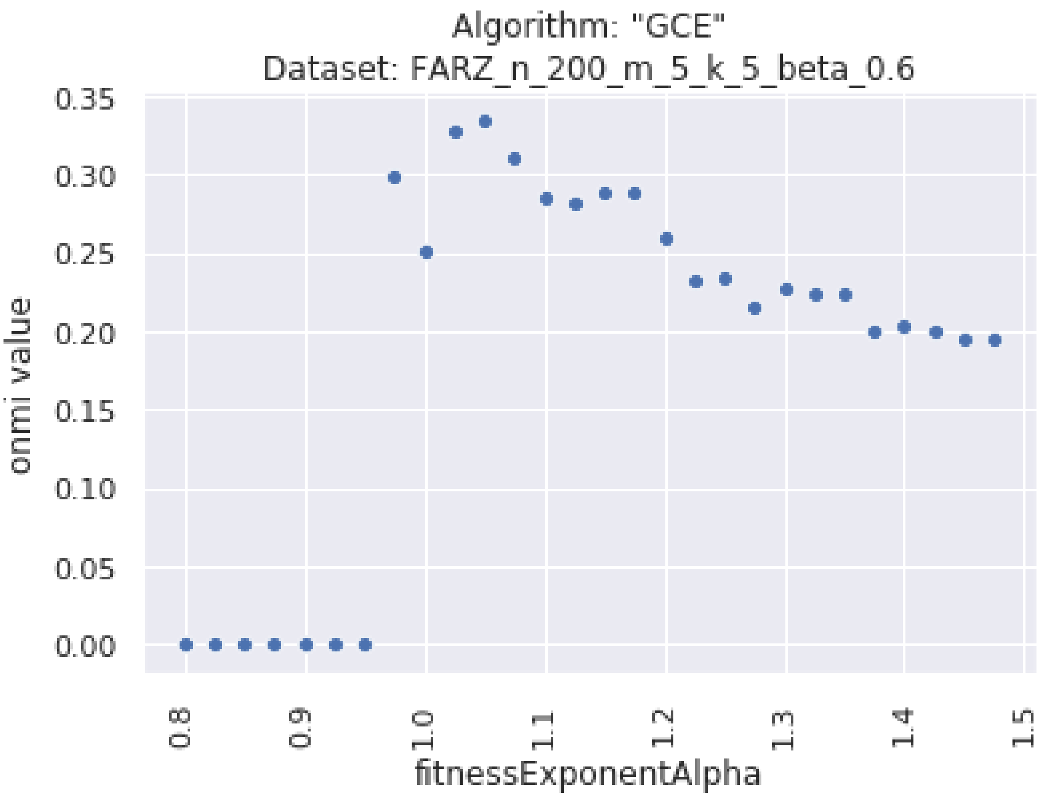

In [101]:
```
plot_graph_for_all_results(all_results, "FARZ_n_200_m_5_k_5_beta_0.6")
```

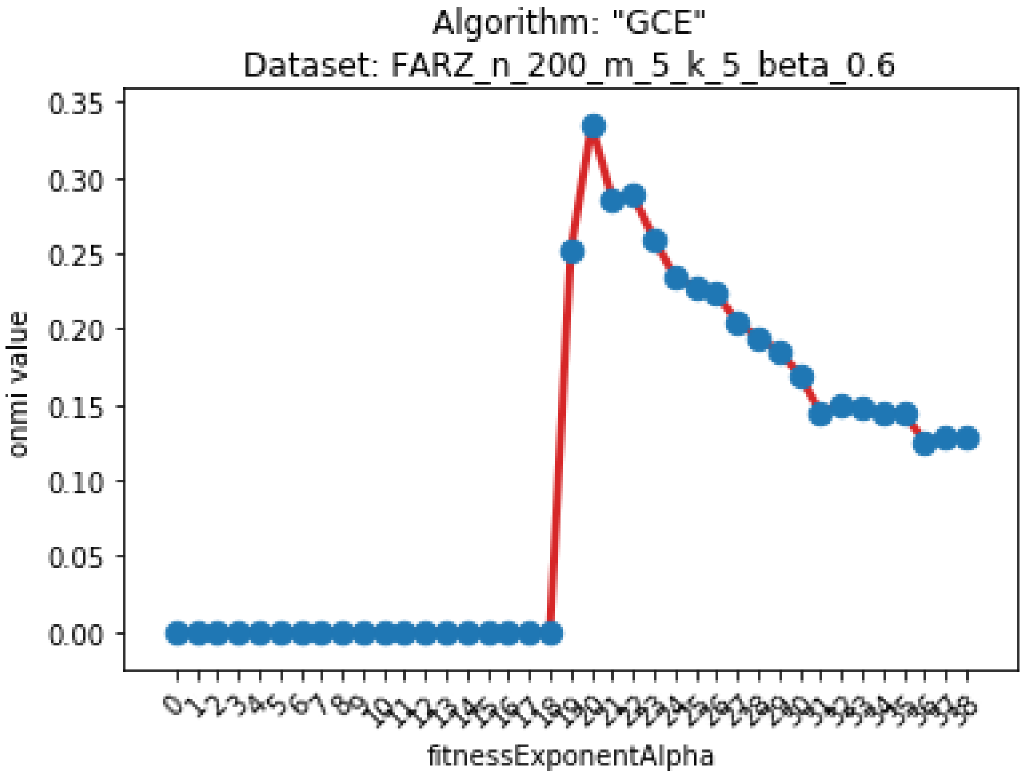

8.10 FARZ_n_200_m_5_k_5_beta_0.55

In [181]:
```
params={}
params[""] = np.arange(0.8, 1.5, 0.025)
inputFile = "../datasets/FARZ_n_200_m_5_k_5_beta_0.55/network.dat"
groundTruth = "../datasets/FARZ_n_200_m_5_k_5_beta_0.55/network.lgt"
all_results = GCE_experiment(inputFile, groundTruth, params, vertexNumerationShift=0,
verbose=False)
```

```
workingDir: ../Results/GCE_FARZ_n_200_m_5_k_5_beta_0.55
outputFile: ../Results/GCE_FARZ_n_200_m_5_k_5_beta_0.55/GCE_output.txt
mkdir: cannot create directory '../Results/GCE_FARZ_n_200_m_5_k_5_beta_0.55': File
exists
```

```
HBox(children=(IntProgress(value=0, max=28), HTML(value='')))
```

```
Best ONMI: 0.287616 params: ' 1.1250000000000004'
Avg ONMI: 0.19438387142857141
```

In [182]:
```
plot_graph_for_all_results(all_results, "FARZ_n_200_m_5_k_5_beta_0.55")
```

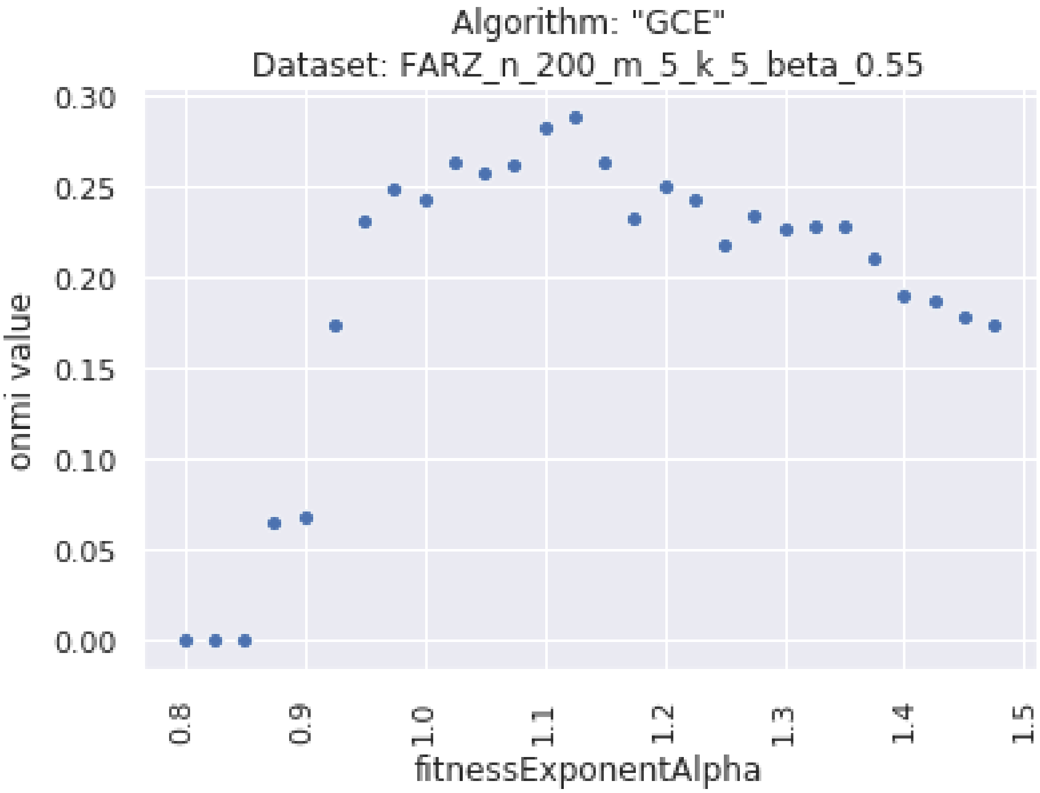


```
In [99]: plot_graph_for_all_results(all_results, "FARZ_n_200_m_5_k_5_beta_0.55")
```

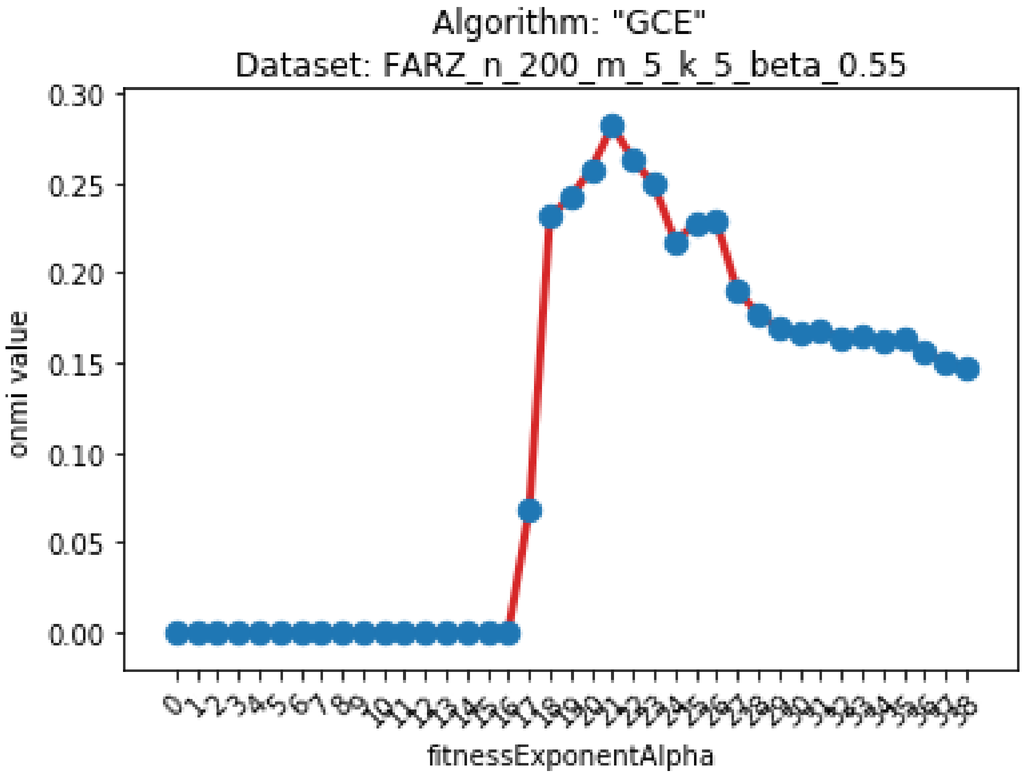

8.11 FARZ_n_200_m_5_k_5_beta_0.5

In [183]:
```
params={}
params[""] = np.arange(0.8, 1.5, 0.025)
inputFile = "../datasets/FARZ_n_200_m_5_k_5_beta_0.5/network.dat"
groundTruth = "../datasets/FARZ_n_200_m_5_k_5_beta_0.5/network.lgt"
all_results = GCE_experiment(inputFile, groundTruth, params, vertexNumerationShift=0,
verbose=False)
```

```
workingDir: ../Results/GCE_FARZ_n_200_m_5_k_5_beta_0.5
outputFile: ../Results/GCE_FARZ_n_200_m_5_k_5_beta_0.5/GCE_output.txt
mkdir: cannot create directory '../Results/GCE_FARZ_n_200_m_5_k_5_beta_0.5': File
exists
```

```
HBox(children=(IntProgress(value=0, max=28), HTML(value='')))
```

```
Best ONMI: 0.3115 params: ' 1.0750000000000002'
Avg ONMI: 0.15310775000000001
```

In [184]:
```
plot_graph_for_all_results(all_results, "FARZ_n_200_m_5_k_5_beta_0.5")
```

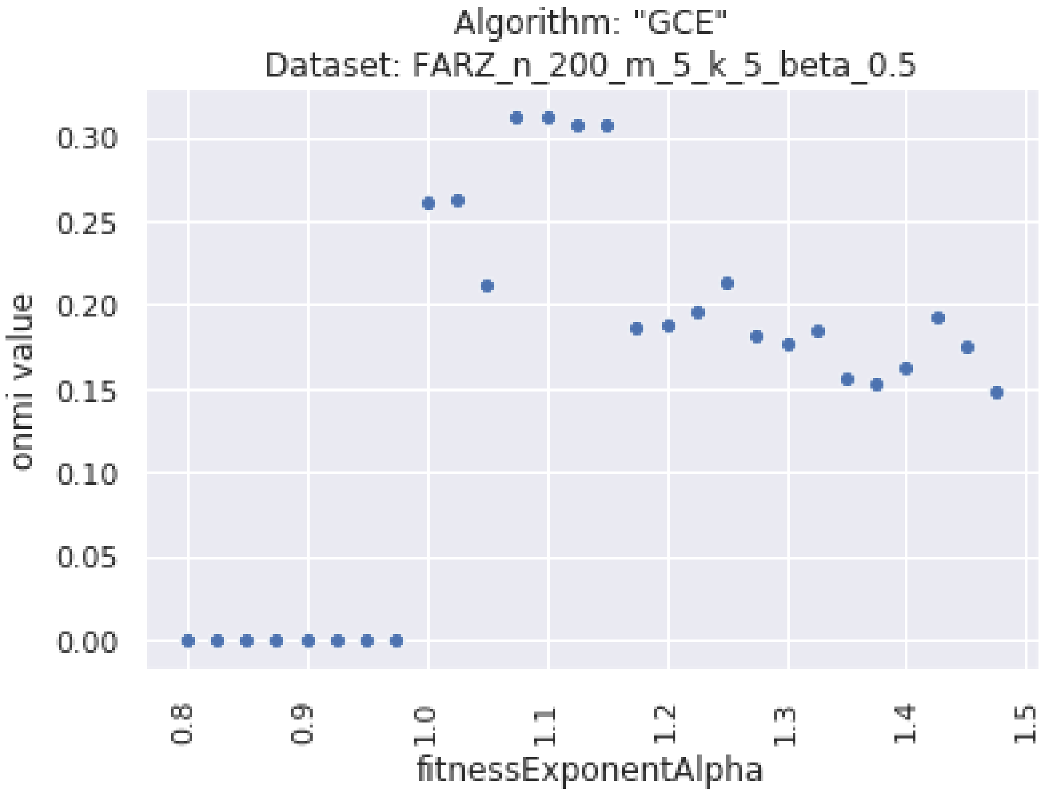

FARZ_n_1000_m_7_k_20_beta_0.9

In [127]:
```
params={}
params[""] = np.arange(0.05, 2.0, 0.05)
inputFile = "../datasets/FARZ_n_1000_m_7_k_20_beta_0.9/network.dat"
groundTruth = "../datasets/FARZ_n_1000_m_7_k_20_beta_0.9/network.lgt"
all_results = GCE_experiment(inputFile, groundTruth, params, vertexNumerationShift=0,
verbose=False)
```

```
workingDir: ../Results/GCE_FARZ_n_1000_m_7_k_20_beta_0.9
outputFile: ../Results/GCE_FARZ_n_1000_m_7_k_20_beta_0.9/GCE_output.txt
mkdir: cannot create directory '../Results/GCE_FARZ_n_1000_m_7_k_20_beta_0.9': File
exists
```

```
HBox(children=(IntProgress(value=0, max=39), HTML(value='')))
```

```
Best ONMI: 0.94957 params: ' 0.6000000000000001'
```

In [128]:
```
plot_graph_for_all_results(all_results, "FARZ_n_1000_m_7_k_20_beta_0.9")
```

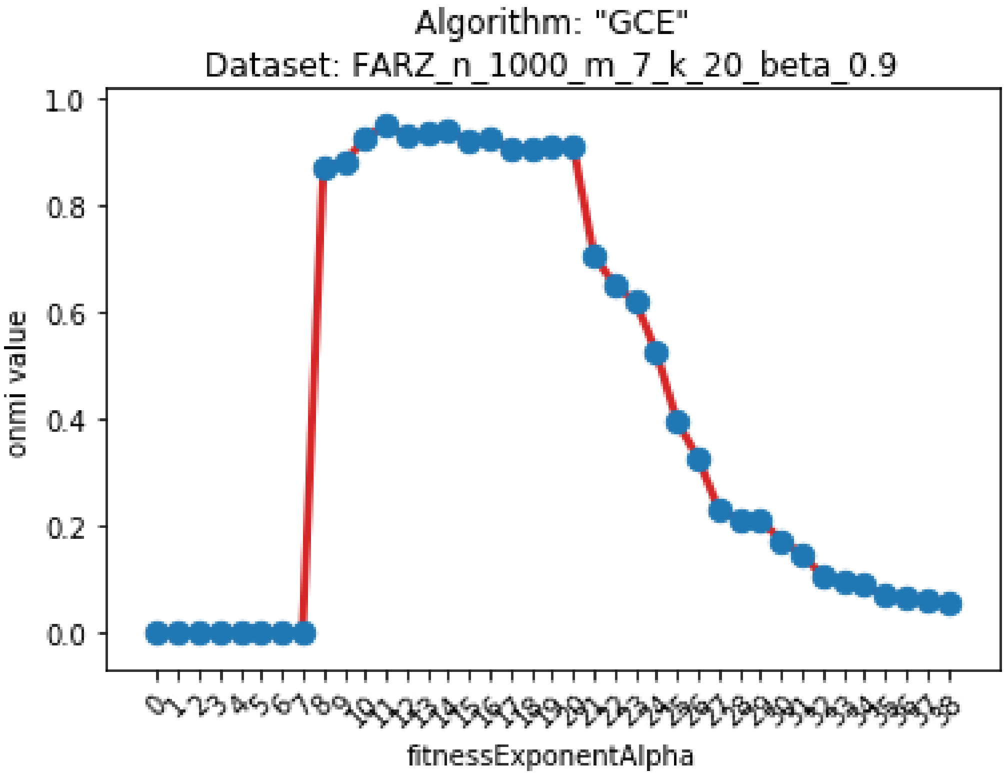

9 CKB and CKB-t

9.1 CKB-t_n_200_alfa_0.1_gamma_0.5

```
In [118]: params={}
          params[""] = np.arange(0.05, 2.0, 0.05)
          inputFile = "../datasets/CKB-t_n_200_alfa_0.1_gamma_0.5/dl_edges_tabs.txt"
          groundTruth = "../datasets/CKB-t_n_200_alfa_0.1_gamma_0.5/dl_coms.txt"
          all_results = GCE_experiment(inputFile, groundTruth, params, vertexNumerationShift=0,
          verbose=False)
```

```
workingDir: ../Results/GCE_CKB-t_n_200_alfa_0.1_gamma_0.5
outputFile: ../Results/GCE_CKB-t_n_200_alfa_0.1_gamma_0.5/GCE_output.txt
```

```
HBox(children=(IntProgress(value=0, max=39), HTML(value='')))
```

```
Best ONMI: 0.0250841 params: ' 0.05'
```

```
In [ ]:
```

9.2 FARZ_n_200_m_5_k_5_beta_0.8

In [70]:
```
params={}
params[""] = np.arange(0.05, 0.95, 0.05)
inputFile = "../datasets/FARZ_n_200_m_5_k_5_beta_0.8/network.dat"
groundTruth = "../datasets/FARZ_n_200_m_5_k_5_beta_0.8/network.lgt"
all_results = GCE_experiment(inputFile, groundTruth, params, vertexNumerationShift=0,
verbose=False)
```

```
workingDir: ../Results/GCE_FARZ_n_200_m_5_k_5_beta_0.8
outputFile: ../Results/GCE_FARZ_n_200_m_5_k_5_beta_0.8/GCE_output.txt
```

```
HBox(children=(IntProgress(value=0, max=18), HTML(value='')))
```

```
Best ONMI: 0.722108 params: ' 0.9000000000000001'
```

In [119]:
```
plot_graph_for_all_results(all_results, "FARZ_n_200_m_5_k_5_beta_0.8")
```

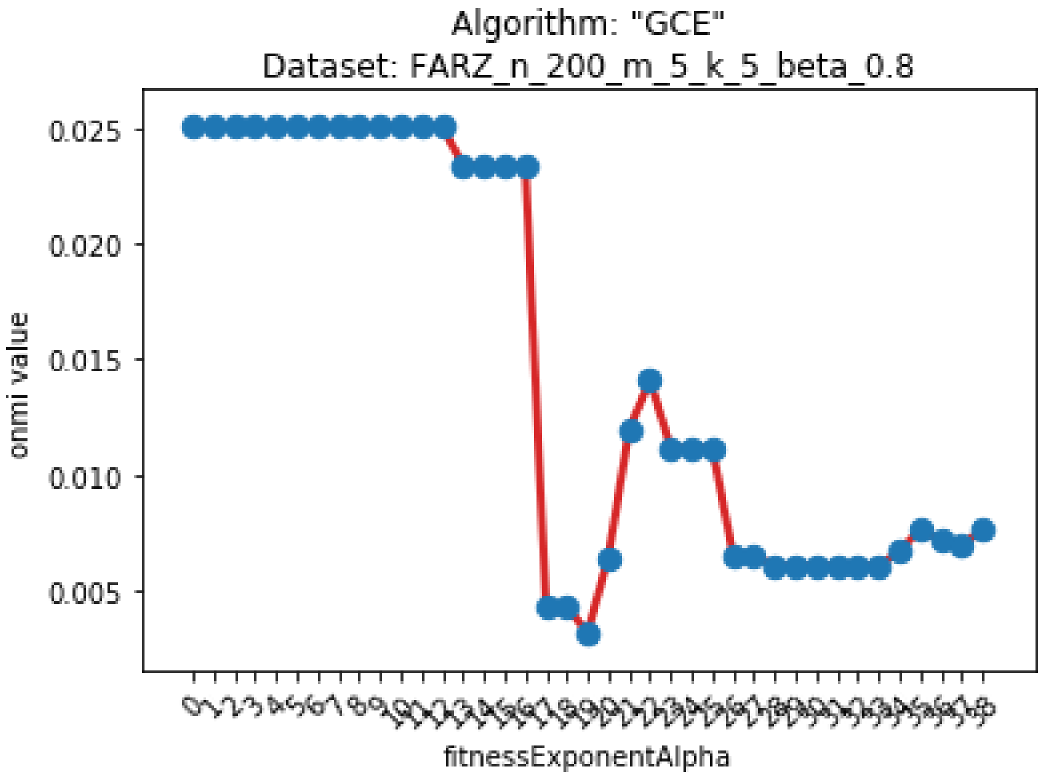

9.3 CKB-t_n_200_alfa_0.1_gamma_0.5

In [120]:
```
params={}
params[""] = np.arange(0.05, 0.95, 0.05)
inputFile = "../datasets/CKB-t_n_200_alfa_0.1_gamma_0.5/dl_edges_tabs.txt"
groundTruth = "../datasets/CKB-t_n_200_alfa_0.1_gamma_0.5/dl_coms.txt"
all_results = GCE_experiment(inputFile, groundTruth, params, vertexNumerationShift=0,
verbose=False)
```

```
workingDir: ../Results/GCE_CKB-t_n_200_alfa_0.1_gamma_0.5
outputFile: ../Results/GCE_CKB-t_n_200_alfa_0.1_gamma_0.5/GCE_output.txt
mkdir: cannot create directory '../Results/GCE_CKB-t_n_200_alfa_0.1_gamma_0.5': File
exists
```

```
HBox(children=(IntProgress(value=0, max=18), HTML(value='')))
```

```
Best ONMI: 0.0250841 params: ' 0.05'
```

In [121]:
```
plot_graph_for_all_results(all_results, "CKB-t_n_200_alfa_0.1_gamma_0.5")
```

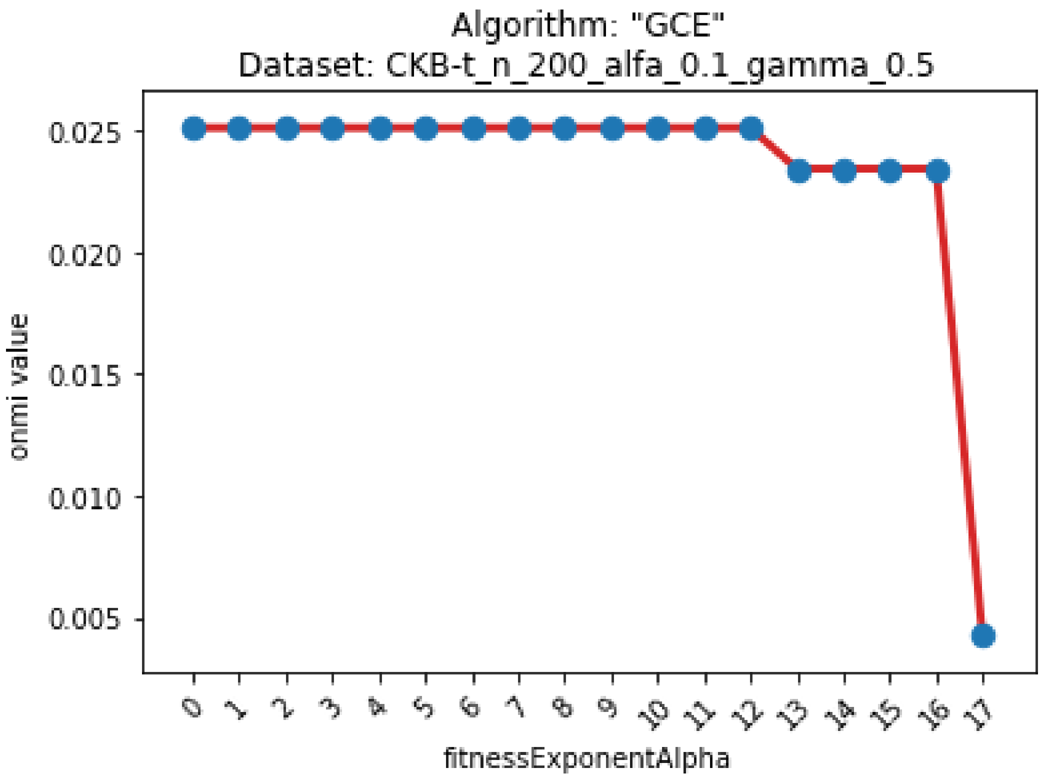

9.4 CKB-t_n_1000_alfa_0.1_gamma_0.5_max_memb_20_max_com_size_200

In [188]:
```
params={}
params[""] = np.arange(0.05, 0.95, 0.05)
inputFile = "../datasets/CKB-
t_n_1000_alfa_0.1_gamma_0.5_max_memb_20_max_com_size_200/dl_edges_tabs.txt"
groundTruth = "../datasets/CKB-
t_n_1000_alfa_0.1_gamma_0.5_max_memb_20_max_com_size_200/dl_coms.txt"
all_results = GCE_experiment(inputFile, groundTruth, params, vertexNumerationShift=0,
verbose=False)
```

```
workingDir: ../Results/GCE_CKB-
t_n_1000_alfa_0.1_gamma_0.5_max_memb_20_max_com_size_200
outputFile: ../Results/GCE_CKB-
t_n_1000_alfa_0.1_gamma_0.5_max_memb_20_max_com_size_200/GCE_output.txt
mkdir: cannot create directory '../Results/GCE_CKB-
t_n_1000_alfa_0.1_gamma_0.5_max_memb_20_max_com_size_200': File exists
```

```
HBox(children=(IntProgress(value=0, max=18), HTML(value='')))
```

```
Best ONMI: 0.0109867 params: ' 0.05'
Avg ONMI: 0.009765955555555556
```

```
In [189]: plot_graph_for_all_results(all_results, "CKB-
          t_n_1000_alfa_0.1_gamma_0.5_max_memb_20_max_com_size_200")
```

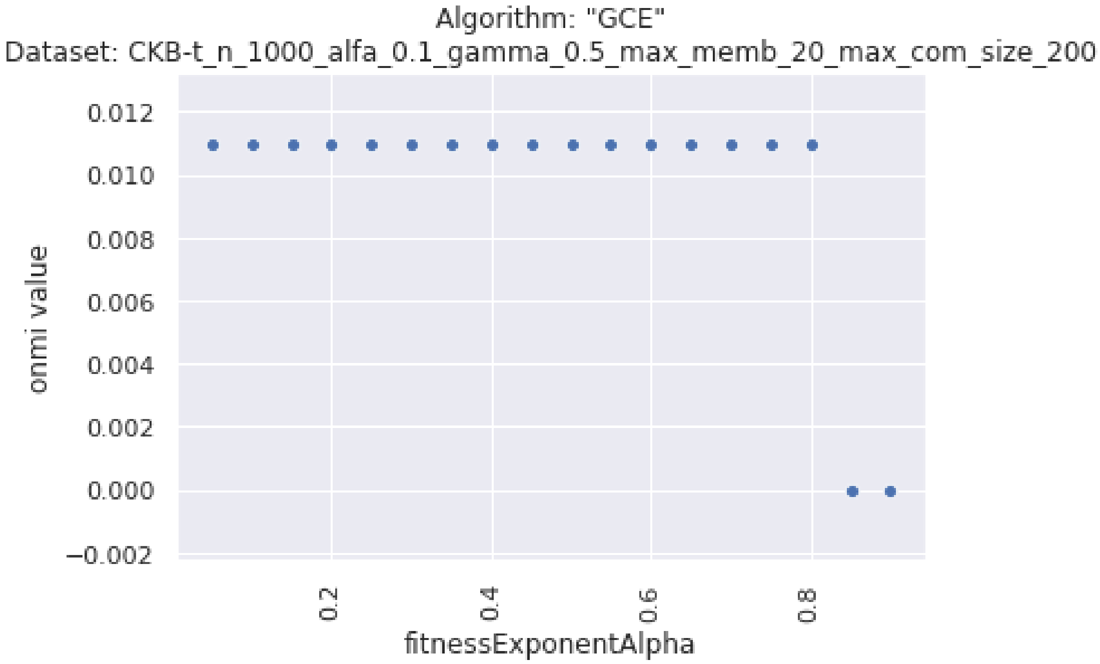


```
In [ ]:
```
