# Supplementary material for: Overlapping community detection in networks based on link partitioning and partitioning around medoids
Source: PLoS One. 2021 Aug 25;16(8):e0255717. doi: 10.1371/journal.pone.0255717 (PMC8386890; doi:10.1371/journal.pone.0255717)
Supplement: S6 Appendix — The computational results for the OSLOM method. (PDF) [file pone.0255717.s006.pdf]

OSLOM

July 18, 2019

1 OSLOM

```
In [2]: import numpy as np
        import random
        random.seed = 108
        from tqdm import tqdm_notebook as tqdm
        import seaborn as sns
        import matplotlib.pyplot as plt
        from mpl_toolkits import mplot3d
        import pandas as pd
        %matplotlib inline
```

```
In [3]: def generate_params(params):
            keys = list(params.keys())
            if len(keys) == 1:
                for value in params[keys[0]]:
                    yield ( keys[0] + " " + str(value) )
            if len( keys ) > 1:
                for value in params[keys[0]]:
                    for remain_params in generate_params({k:params[k] for k in keys[1:]}):
                        yield ( keys[0] + " " + str(value) + " " + remain_params )
```

```
In [4]: def oslom_experiment(inputFileFull, groundTruth, params = {}, vertexNumerationShift=0,
        verbose=False):
            datasetName = inputFileFull.split('/')[-2]
            outputDir = "../Results/OSLOM2_{}".format(datasetName)
            inputFile =  outputDir + '/' + inputFileFull.split('/')[-1]
            outputFile = outputDir + '/' + inputFileFull.split('/')[-1] + "_oslo_files/tp"
        #     outputFile = outputDir + '/' "tp"

            !mkdir {outputDir}
            !cp {inputFileFull} {outputDir}

            print("Output dir name: {}".format(outputDir) )
            print("old inputFileName : {}".format(inputFileFull) )
            print("new inputFileName : {}".format(inputFile) )
            print("Output file name: {}".format(outputFile) )

            !mkdir {outputDir}

            all_results = {}
            bestParam = "not found"
            onmi_best = 0;
            for param in tqdm(list(generate_params(params))):
                tmp=!(cd {outputDir} && ../../related_methods/OSLOM2/oslom_undir -f
        ../{inputFile} -uw {param})
        #         tmp=!(cd {outputDir} && ../related_methods/OSLOM2/oslom_undir -f
        ../{inputFile} -uw {param})
                if verbose:
                    print(tmp)
                !sed -i '/^#/d' {outputFile}
                lines=[]
```

```
            with open(outputFile) as f:
                lines = f.readlines()
            with open(outputFile, 'w') as the_file:
                for line in lines:
                    the_file.write(" ".join([str(int(a)+vertexNumerationShift) for a in
line.split()]) + "\n")
            output = !../Overlapping-NMI/onmi {groundTruth} {outputFile}
            onmi=float(output[0].split()[1])
            all_results[param] = onmi
            if onmi >  onmi_best:
                bestParam = param
                onmi_best = onmi


        mean_results = {}
        for line, value in all_results.items():
            key = " ".join(line.split()[:4])
            if key in mean_results:
                mean_results[key].append(value)
            else:
                mean_results[key] = [value]

        onmi_best_avg = np.max([np.mean(values) for key, values in mean_results.items() ])
        tmp=!(cd {outputDir} && ../../related_methods/OSLOM2/oslom_undir -f ../{inputFile}
-uw {bestParam})
        if verbose:
            print(tmp)
        !sed -i '/^#/d' {outputFile}
        lines=[]
        with open(outputFile) as f:
            lines = f.readlines()
        with open(outputFile, 'w') as the_file:
            for line in lines:
                the_file.write(" ".join([str(int(a)+vertexNumerationShift) for a in
line.split()]) + "\n")

        print("Best of Max ONMI: {} params: '{}'".format(onmi_best, bestParam) )
        print("Best of Avarage ONMI: {} params: '{}'".format(onmi_best_avg, bestParam) )
        return all_results
```

```
In [5]: def plot_graph_for_all_results(all_results, datasetName, random_choice='max'):
        xdata=[]
        ydata=[]
        zdata=[]
        df = pd.DataFrame()
        for param, onmi in all_results.items():
            splited = param.split()
            xdata.append(float(splited[1]))
            ydata.append(float(splited[3]))
            zdata.append(onmi)
            df = df.append({'x': float(splited[1]), 'y': float(splited[3]) , 'seed':
int(splited[5]), 'z': onmi}, ignore_index=True)

        df_ddd = df.groupby(['x','y'], as_index=False)['z'].max()
        if (random_choice == 'mean'):
            df_ddd = df.groupby(['x','y'], as_index=False)['z'].mean()

        #coolwarm  viridis
        fig = plt.figure(figsize=(24, 20), dpi= 80)
        ax = plt.axes(projection='3d')
        ax.plot_trisurf(df_ddd.x.values, df_ddd.y.values, df_ddd.z.values, cmap='plasma',
edgecolor='none')
        ax.view_init(30, 55)
        ax.set_xlabel('t parameter')
        ax.set_ylabel('cp paramter')
        ax.set_zlabel('onmi vlue');
        ax.set_title('OSLOM\n Onmi {} values for 10-random seeds\nDataset:
{}'.format(random_choice.upper(), datasetName));
```

```
In [45]: params={}
         params["-t"] = np.arange(0.05, 0.95, 0.1)
         params["-cp"] = np.arange(0.05, 0.95, 0.1)
         params["-seed"] = random.sample(range(1, 100), 10)
```

2 School Friendship Network

```
In [192]: inputFile = "../datasets/school_friendship/school-2.dat"
          groundTruth = "../datasets/school_friendship/truth-school.dat"

          all_results = oslom_experiment(inputFile, groundTruth, params, vertexNumerationShift=-1)
```

```
mkdir: cannot create directory ‘../Results/OSLOM2_school_friendship’: File exists
Output dir name: ../Results/OSLOM2_school_friendship
old inputFileName : ../datasets/school_friendship/school-2.dat
new inputFileName : ../Results/OSLOM2_school_friendship/school-2.dat
Output file name: ../Results/OSLOM2_school_friendship/school-2.dat_oslo_files/tp
mkdir: cannot create directory ‘../Results/OSLOM2_school_friendship’: File exists
```

```
HBox(children=(IntProgress(value=0, max=810), HTML(value='')))
```

```
Best of Max ONMI: 0.678634 params: '-t 0.35000000000000003 -cp 0.45000000000000007
-seed 63'
Best of Avarage ONMI: 0.5651837999999999 params: '-t 0.35000000000000003 -cp
0.45000000000000007 -seed 63'
```

```
In [ ]: plot_graph_for_all_results(all_results, "school_friendship", random_choice="mean")
```

```
In [97]: plot_graph_for_all_results(all_results, "school_friendship", random_choice='mean')
```

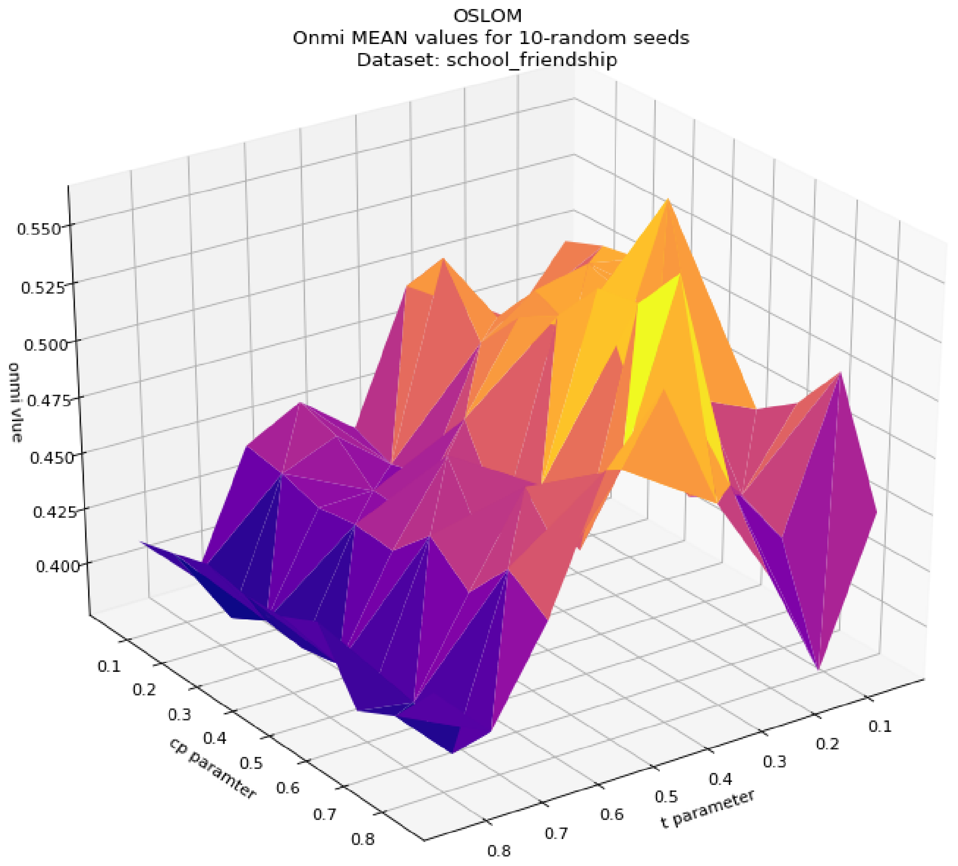
OSLOM
Onmi MEAN values for 10-random seeds
Dataset: school_friendship
onmi vlue
cp paramter
t parameter
0.550
0.525
0.500
0.475
0.450
0.425
0.400
0.1
0.2
0.3
0.4
0.5
0.6
0.7
0.8

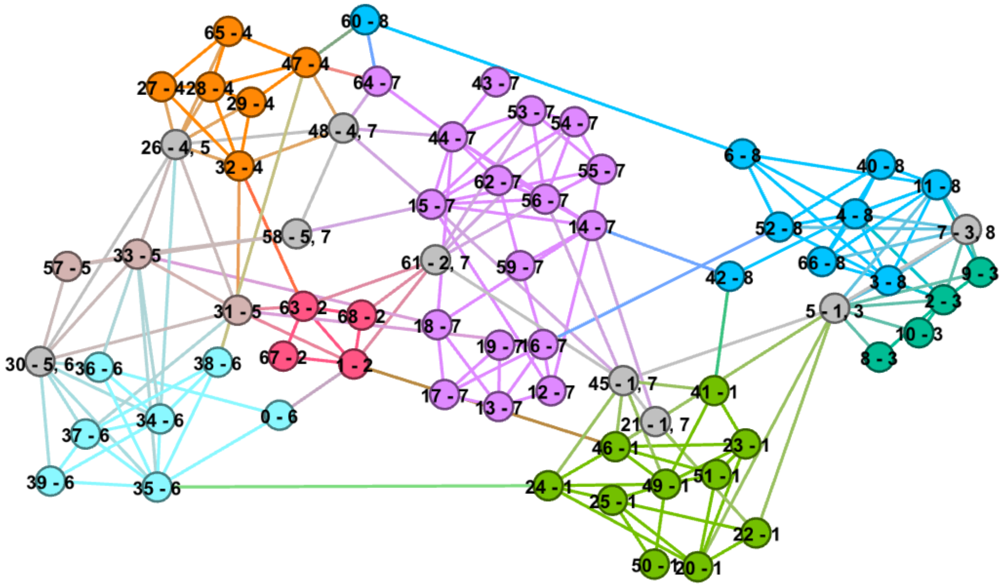

School Friendship Network. Algorithm - OSLOM

3 Karate Club

```
In [193]: inputFile = "../datasets/karate/karate.dat"
          groundTruth = "../datasets/karate/truth_karate.dat"
          all_results = oslom_experiment(inputFile, groundTruth, params, vertexNumerationShift=0)
```

```
mkdir: cannot create directory ‘../Results/OSLOM2_karate’: File exists
Output dir name: ../Results/OSLOM2_karate
old inputFileName : ../datasets/karate/karate.dat
new inputFileName : ../Results/OSLOM2_karate/karate.dat
Output file name: ../Results/OSLOM2_karate/karate.dat_oslo_files/tp
mkdir: cannot create directory ‘../Results/OSLOM2_karate’: File exists
```

```
HBox(children=(IntProgress(value=0, max=810), HTML(value='')))
```

```
Best of Max ONMI: 1.0 params: '-t 0.35000000000000003 -cp 0.05 -seed 76'
Best of Avarage ONMI: 0.9586149 params: '-t 0.35000000000000003 -cp 0.05 -seed 76'
```

```
In [194]: plot_graph_for_all_results(all_results, "Karate club", random_choice='mean')
```

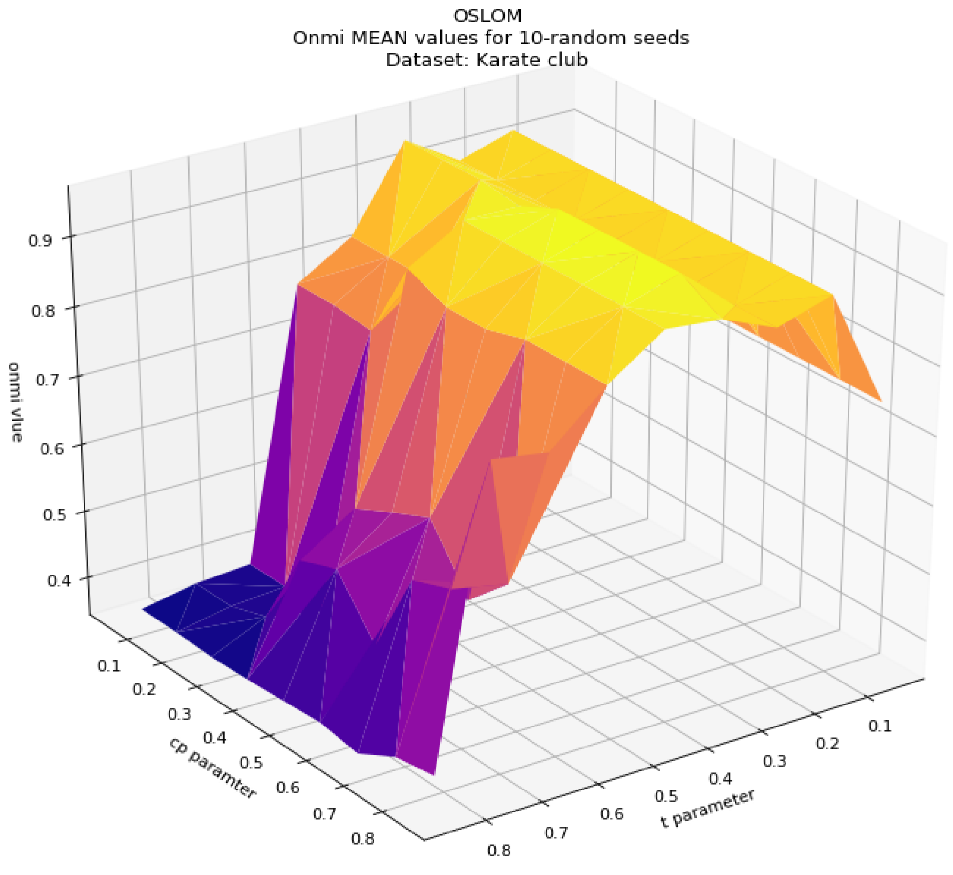
OSLOM
Onmi MEAN values for 10-random seeds
Dataset: Karate club
onmi vlue
0.9
0.8
0.7
0.6
0.5
0.4
cp paramter
0.1
0.2
0.3
0.4
0.5
0.6
0.7
0.8
t parameter
0.1
0.2
0.3
0.4
0.5
0.6
0.7
0.8

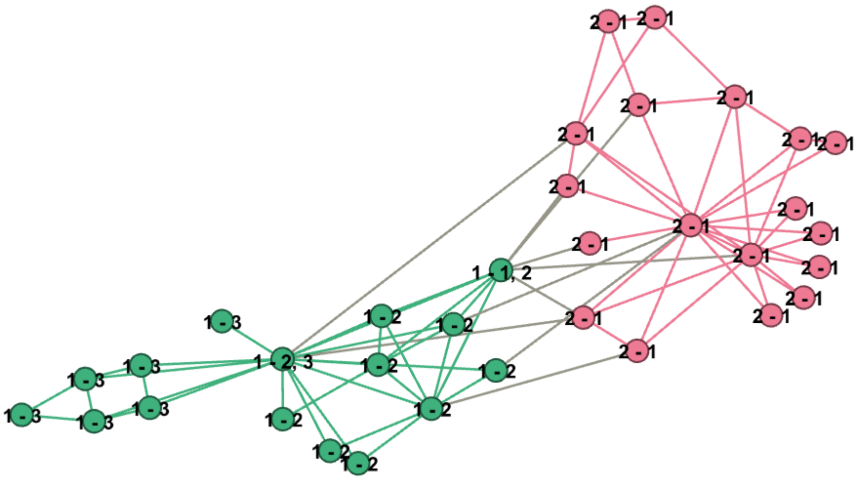

Karate Club. Algorithm - OSLOM

4 Adj-noun

```
In [195]: inputFile = "../datasets/adjnoun/adjnoun.dat"
          groundTruth = "../datasets/adjnoun/truth_adjnoun.dat"
          all_results = oslom_experiment(inputFile, groundTruth, params, vertexNumerationShift=0)
```

```
mkdir: cannot create directory '../Results/OSLOM2_adjnoun': File exists
Output dir name: ../Results/OSLOM2_adjnoun
old inputFileName : ../datasets/adjnoun/adjnoun.dat
new inputFileName : ../Results/OSLOM2_adjnoun/adjnoun.dat
Output file name: ../Results/OSLOM2_adjnoun/adjnoun.dat_oslo_files/tp
mkdir: cannot create directory '../Results/OSLOM2_adjnoun': File exists
```

```
HBox(children=(IntProgress(value=0, max=810), HTML(value='')))
```

```
Best of Max ONMI: 0.0620827 params: '-t 0.05 -cp 0.05 -seed 33'
Best of Avarage ONMI: 0.062082700000000005 params: '-t 0.05 -cp 0.05 -seed 33'
```

```
In [196]: plot_graph_for_all_results(all_results, "Adj Noun", random_choice='mean')
```

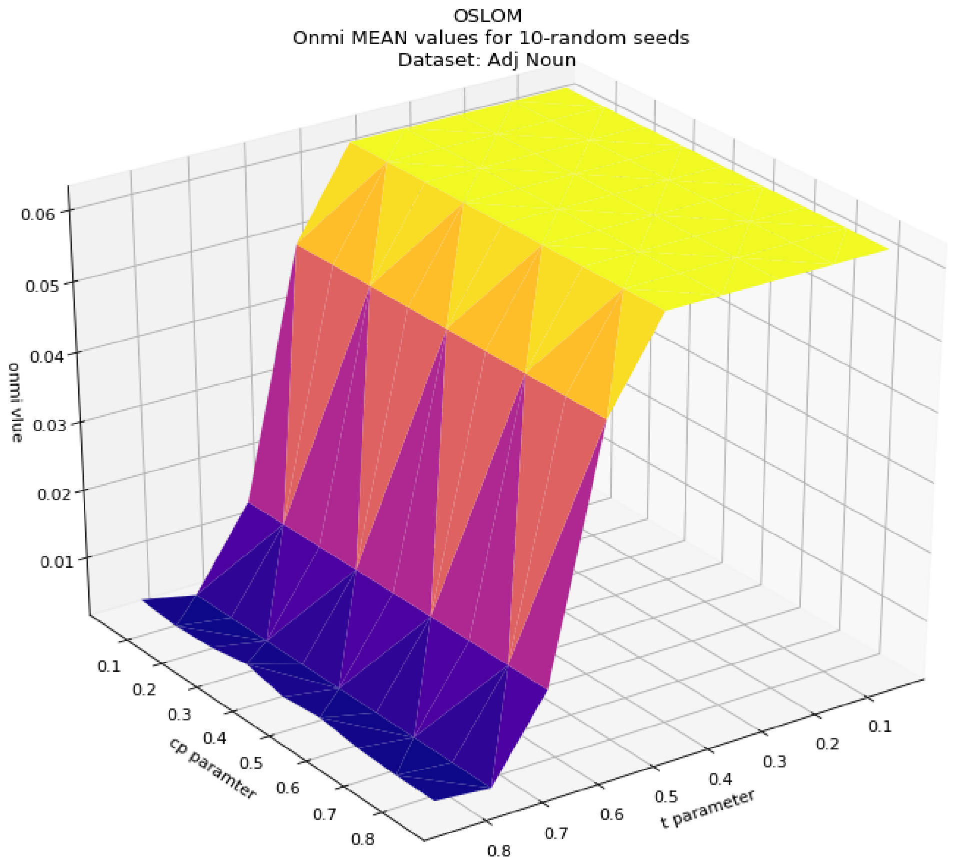

5 American Football League

```
In [197]: inputFile = "../datasets/football/footballTSEinput_original.dat"
          groundTruth = "../datasets/football/truth_footballTSEinput.dat"
          all_results = oslom_experiment(inputFile, groundTruth, params, vertexNumerationShift=-1)
```

```
mkdir: cannot create directory '../Results/OSLOM2_football': File exists
Output dir name: ../Results/OSLOM2_football
old inputFileName : ../datasets/football/footballTSEinput_original.dat
new inputFileName : ../Results/OSLOM2_football/footballTSEinput_original.dat
Output file name:
../Results/OSLOM2_football/footballTSEinput_original.dat_oslo_files/tp
mkdir: cannot create directory '../Results/OSLOM2_football': File exists
```

```
HBox(children=(IntProgress(value=0, max=810), HTML(value='')))
```

```
Best of Max ONMI: 0.920835 params: '-t 0.15000000000000002 -cp 0.25000000000000006
-seed 76'
Best of Avarage ONMI: 0.9077013 params: '-t 0.15000000000000002 -cp
```

```
0.25000000000000006 -seed 76'
```

```
In [198]: plot_graph_for_all_results(all_results, "American Fooball League", random_choice='mean')
```

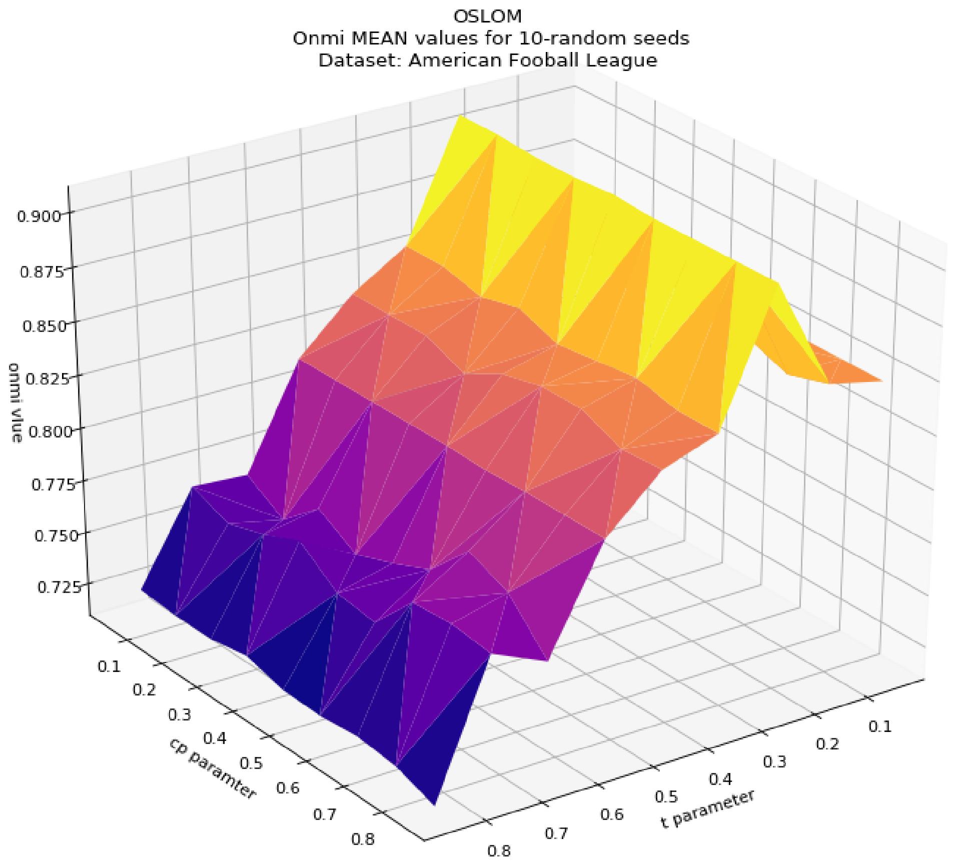

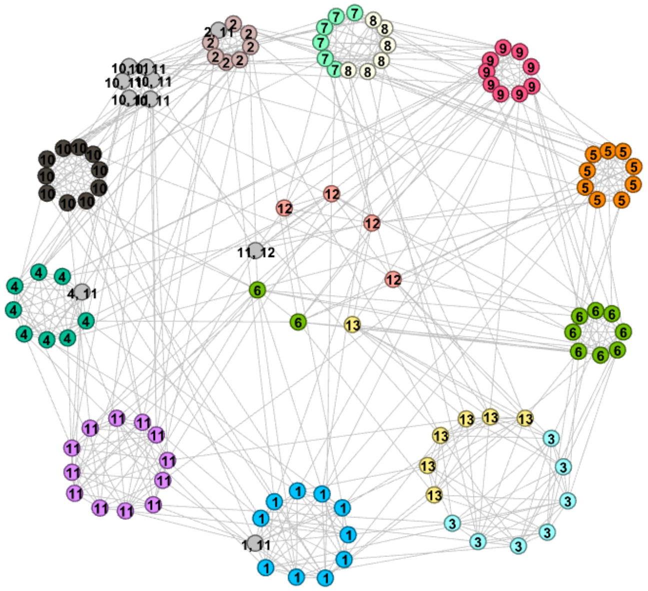

American Football League. Algorithm - OSLOM

6 Political Books

```
In [199]: inputFile = "../datasets/polbooks/polbooks.dat"
          groundTruth = "../datasets/polbooks/truth_polbooks.dat"
          all_results = oslom_experiment(inputFile, groundTruth, params, vertexNumerationShift=0)
```

```
mkdir: cannot create directory '../Results/OSLOM2_polbooks': File exists
Output dir name: ../Results/OSLOM2_polbooks
old inputFileName : ../datasets/polbooks/polbooks.dat
new inputFileName : ../Results/OSLOM2_polbooks/polbooks.dat
Output file name: ../Results/OSLOM2_polbooks/polbooks.dat_oslo_files/tp
mkdir: cannot create directory '../Results/OSLOM2_polbooks': File exists
```

```
HBox(children=(IntProgress(value=0, max=810), HTML(value='')))
```

```
Best of Max ONMI: 0.474083 params: '-t 0.05 -cp 0.6500000000000001 -seed 73'
Best of Avarage ONMI: 0.4382833 params: '-t 0.05 -cp 0.6500000000000001 -seed 73'
```

```
In [200]: plot_graph_for_all_results(all_results, "Political Books", random_choice='mean')
```

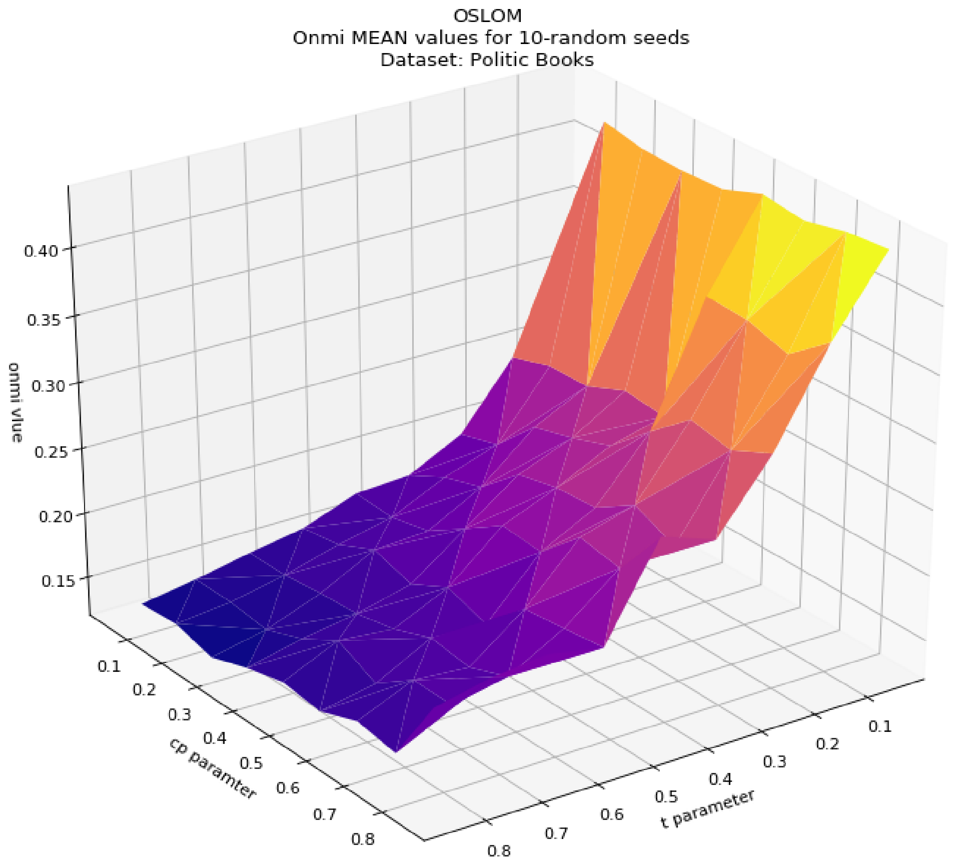
OSLOM
Onmi MEAN values for 10-random seeds
Dataset: Politic Books
onmi vlue
0.40
0.35
0.30
0.25
0.20
0.15
cp paramter
0.1
0.2
0.3
0.4
0.5
0.6
0.7
0.8
t parameter
0.1
0.2
0.3
0.4
0.5
0.6
0.7
0.8

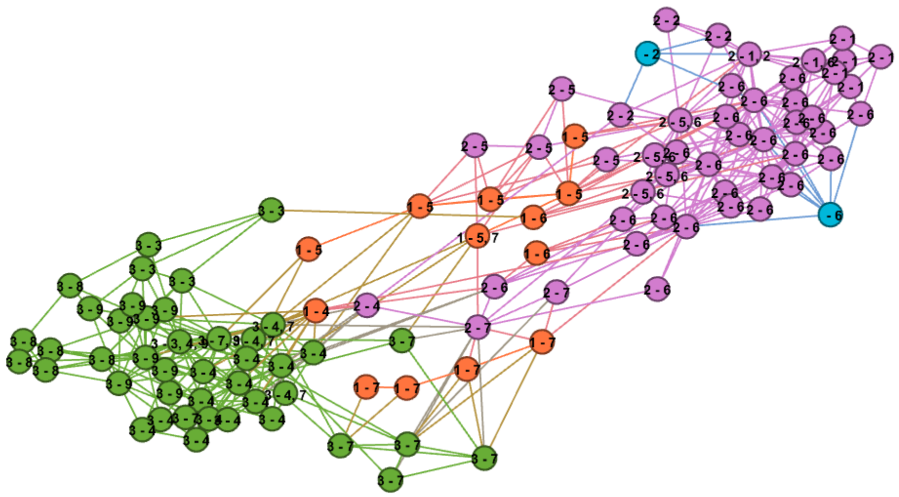

Political Books. Algorithm - OSLOM

7 Syntetic Datasets

7.1 bench_30

```
In [163]: inputFile = "../datasets/bench_30/bench_30_network.dat"
          groundTruth = "../datasets/bench_30/bench_30_truth.dat"
          all_results = oslom_experiment(inputFile, groundTruth, params, vertexNumerationShift=0)
```

```
mkdir: cannot create directory ‘../Results/OSLOM2_bench_30’: File exists
Output dir name: ../Results/OSLOM2_bench_30
old inputFileName : ../datasets/bench_30/bench_30_network.dat
new inputFileName : ../Results/OSLOM2_bench_30/bench_30_network.dat
Output file name: ../Results/OSLOM2_bench_30/bench_30_network.dat_oslo_files/tp
mkdir: cannot create directory ‘../Results/OSLOM2_bench_30’: File exists
```

```
HBox(children=(IntProgress(value=0, max=810), HTML(value='')))
```

```
Best of Max ONMI: 0.931866 params: '-t 0.15000000000000002 -cp 0.05 -seed 90'
Best of Avarage ONMI: 0.9246478 params: '-t 0.15000000000000002 -cp 0.05 -seed 90'
```

```
In [164]: plot_graph_for_all_results(all_results, "bench_30", random_choice='mean')
```

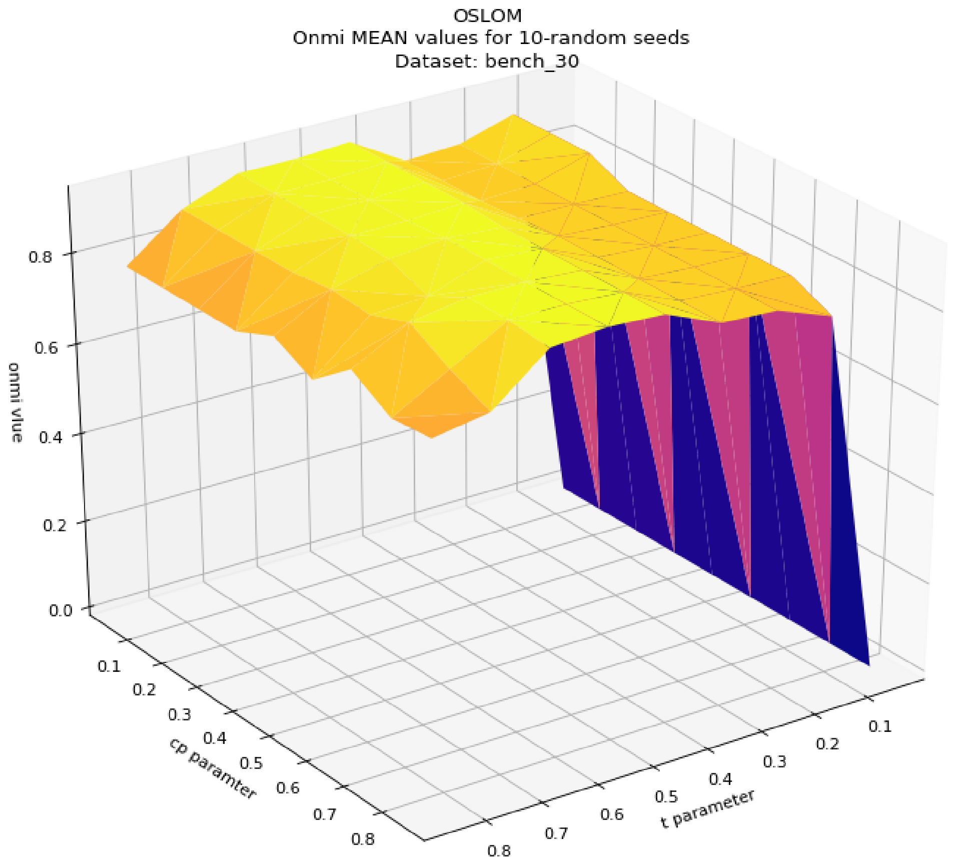
OSLOM
Onmi MEAN values for 10-random seeds
Dataset: bench_30
onmi vlue
0.0
0.2
0.4
0.6
0.8
cp paramter
0.1
0.2
0.3
0.4
0.5
0.6
0.7
0.8
t parameter
0.1
0.2
0.3
0.4
0.5
0.6
0.7
0.8

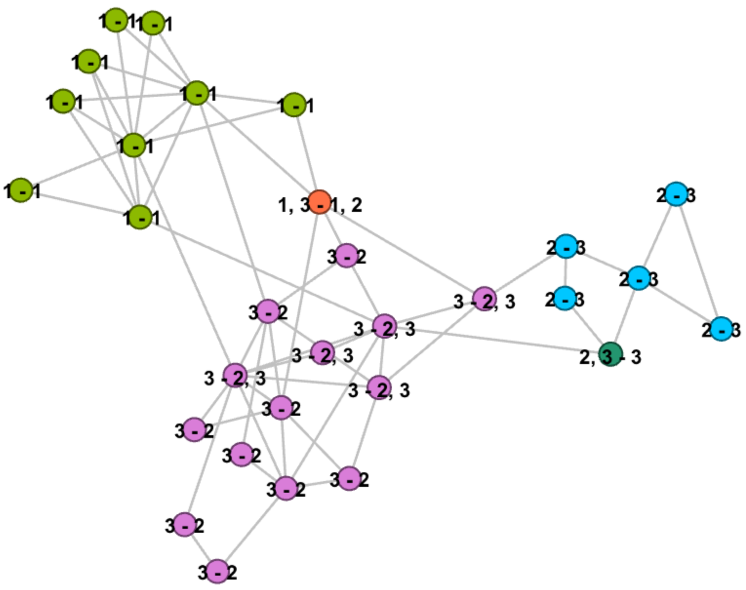

Bench_30. Algorithm - OSLOM

7.2 bench_40

```
In [184]: inputFile = "../datasets/bench_40/bench_40_network.dat"
          groundTruth = "../datasets/bench_40/bench_40_truth.dat"
          all_results = oslom_experiment(inputFile, groundTruth, params, vertexNumerationShift=0)
```

```
mkdir: cannot create directory ‘../Results/OSLOM2_bench_40’: File exists
Output dir name: ../Results/OSLOM2_bench_40
old inputFileName : ../datasets/bench_40/bench_40_network.dat
new inputFileName : ../Results/OSLOM2_bench_40/bench_40_network.dat
Output file name: ../Results/OSLOM2_bench_40/bench_40_network.dat_oslo_files/tp
mkdir: cannot create directory ‘../Results/OSLOM2_bench_40’: File exists
```

```
HBox(children=(IntProgress(value=0, max=810), HTML(value='')))
```

```
Best of Max ONMI: 0.635964 params: '-t 0.45000000000000007 -cp 0.05 -seed 66'
Best of Avarage ONMI: 0.6039149 params: '-t 0.45000000000000007 -cp 0.05 -seed 66'
```

```
In [185]: plot_graph_for_all_results(all_results, "bench_40", random_choice='mean')
```

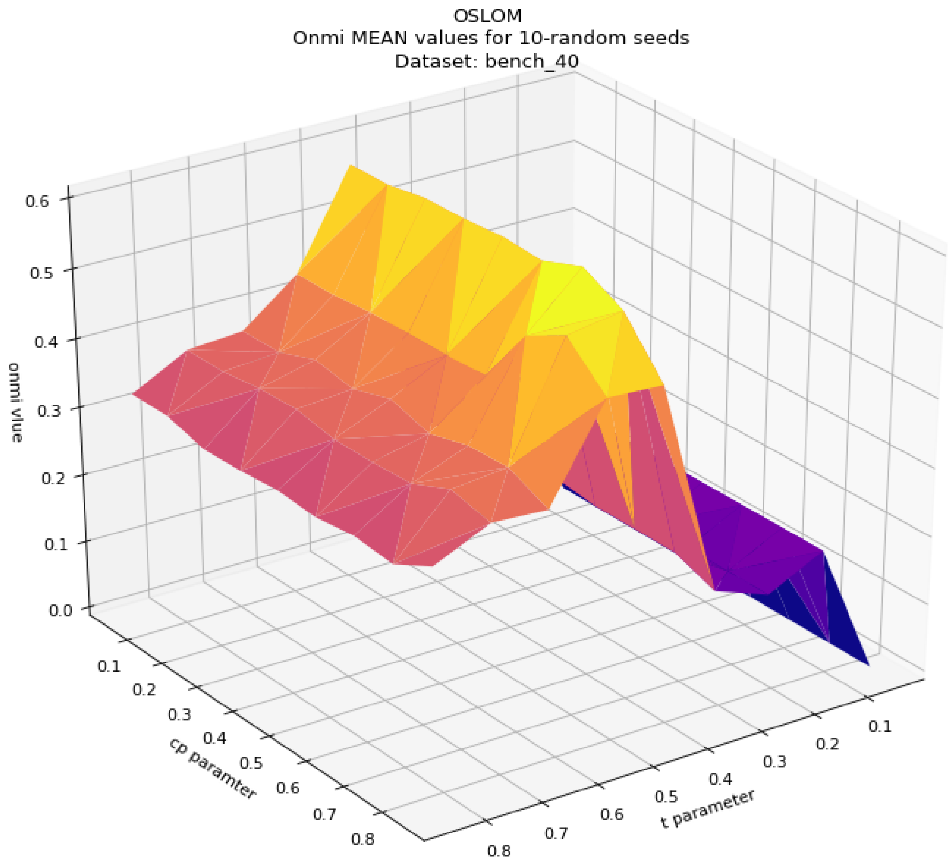
OSLOM
Onmi MEAN values for 10-random seeds
Dataset: bench_40
onmi vlue
cp paramter
t parameter
0.0
0.1
0.2
0.3
0.4
0.5
0.6
0.7
0.8

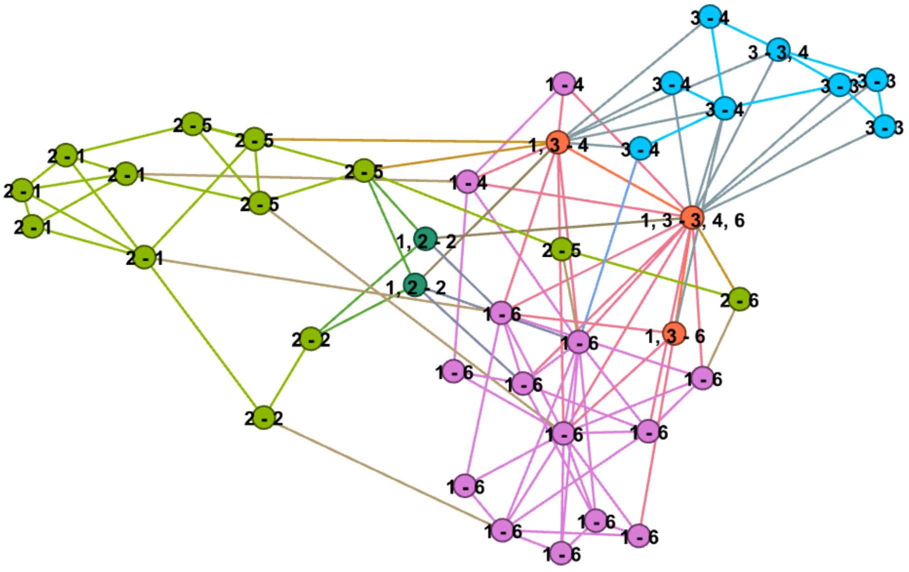

Bench_40. Algorithm - OSLOM

7.3 bench_50

```
In [186]: inputFile = "../datasets/bench_50/bench_50_network.dat"
          groundTruth = "../datasets/bench_50/bench_50_truth.dat"
          all_results = oslom_experiment(inputFile, groundTruth, params, vertexNumerationShift=0)
```

```
mkdir: cannot create directory ‘../Results/OSLOM2_bench_50’: File exists
Output dir name: ../Results/OSLOM2_bench_50
old inputFileName : ../datasets/bench_50/bench_50_network.dat
new inputFileName : ../Results/OSLOM2_bench_50/bench_50_network.dat
Output file name: ../Results/OSLOM2_bench_50/bench_50_network.dat_oslo_files/tp
mkdir: cannot create directory ‘../Results/OSLOM2_bench_50’: File exists
```

```
HBox(children=(IntProgress(value=0, max=810), HTML(value='')))
```

```
Best of Max ONMI: 0.886444 params: '-t 0.5500000000000002 -cp 0.35000000000000003
-seed 26'
Best of Avarage ONMI: 0.7614544999999999 params: '-t 0.5500000000000002 -cp
0.35000000000000003 -seed 26'
```

```
In [187]: plot_graph_for_all_results(all_results, "bench_50", random_choice='mean')
```

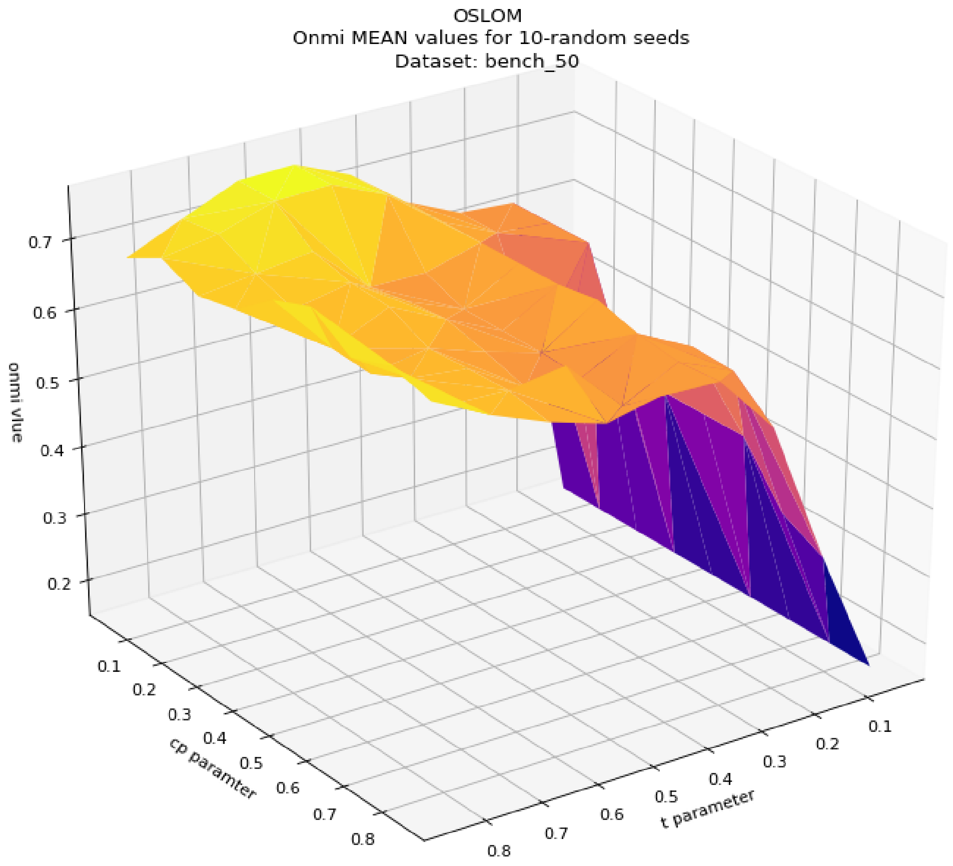
OSLOM
Onmi MEAN values for 10-random seeds
Dataset: bench_50
onmi vlue
0.7
0.6
0.5
0.4
0.3
0.2
cp paramter
0.1
0.2
0.3
0.4
0.5
0.6
0.7
0.8
t parameter
0.1
0.2
0.3
0.4
0.5
0.6
0.7
0.8

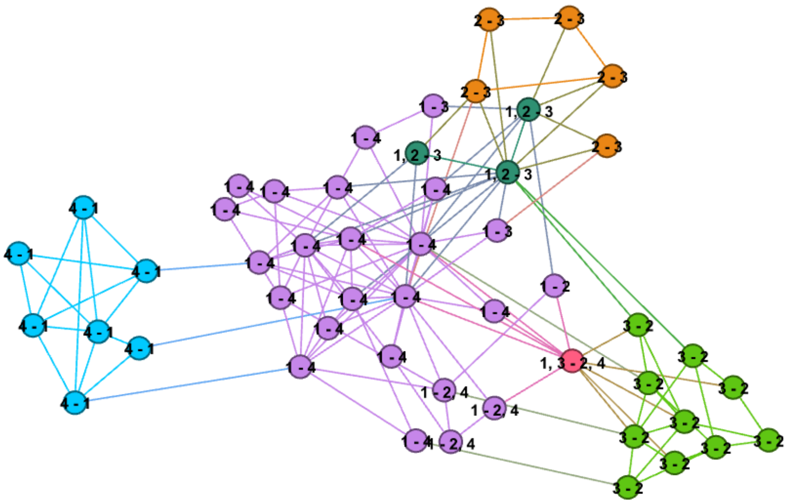

Bench_50. Algorithm - OSLOM

7.4 bench_60

```
In [188]: inputFile = "../datasets/bench_60/bench_60_network.dat"
          groundTruth = "../datasets/bench_60/bench_60_truth.dat"
          all_results = oslom_experiment(inputFile, groundTruth, params, vertexNumerationShift=0)
```

```
mkdir: cannot create directory ‘../Results/OSLOM2_bench_60’: File exists
Output dir name: ../Results/OSLOM2_bench_60
old inputFileName : ../datasets/bench_60/bench_60_network.dat
new inputFileName : ../Results/OSLOM2_bench_60/bench_60_network.dat
Output file name: ../Results/OSLOM2_bench_60/bench_60_network.dat_oslo_files/tp
mkdir: cannot create directory ‘../Results/OSLOM2_bench_60’: File exists
```

```
HBox(children=(IntProgress(value=0, max=810), HTML(value='')))
```

```
Best of Max ONMI: 0.793144 params: '-t 0.35000000000000003 -cp 0.05 -seed 26'
Best of Avarage ONMI: 0.7709094999999999 params: '-t 0.35000000000000003 -cp 0.05
-seed 26'
```

```
In [189]: plot_graph_for_all_results(all_results, "bench_60", random_choice='mean')
```

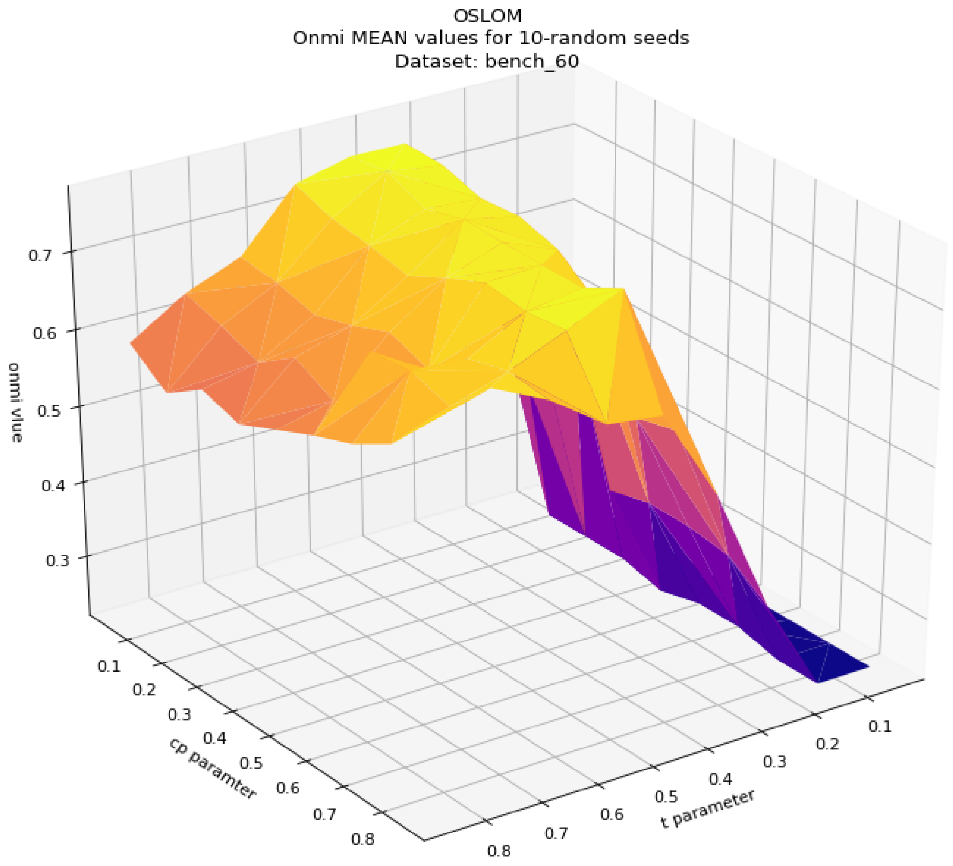
OSLOM
Onmi MEAN values for 10-random seeds
Dataset: bench_60
onmi vlue
0.7
0.6
0.5
0.4
0.3
cp paramter
0.1
0.2
0.3
0.4
0.5
0.6
0.7
0.8
t parameter
0.1
0.2
0.3
0.4
0.5
0.6
0.7
0.8

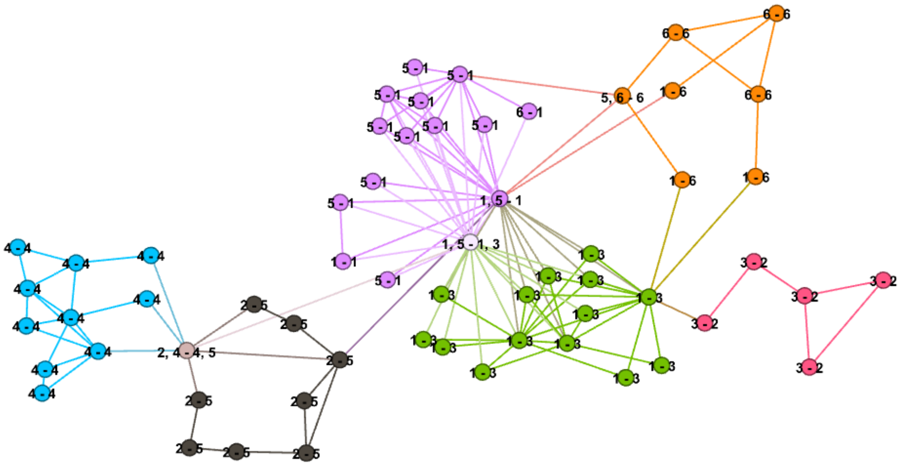

Bench_60. Algorithm - OSLOM

7.5 bench_60_dense

```
In [190]: inputFile = "../datasets/bench_60_dense/bench_60_dense_network.dat"
          groundTruth = "../datasets/bench_60_dense/bench_60_dense_truth.dat"
          all_results = oslom_experiment(inputFile, groundTruth, params, vertexNumerationShift=0)
```

```
mkdir: cannot create directory '../Results/OSLOM2_bench_60_dense': File exists
Output dir name: ../Results/OSLOM2_bench_60_dense
old inputFileName : ../datasets/bench_60_dense/bench_60_dense_network.dat
new inputFileName : ../Results/OSLOM2_bench_60_dense/bench_60_dense_network.dat
Output file name:
../Results/OSLOM2_bench_60_dense/bench_60_dense_network.dat_oslo_files/tp
mkdir: cannot create directory '../Results/OSLOM2_bench_60_dense': File exists
```

```
HBox(children=(IntProgress(value=0, max=810), HTML(value='')))
```

```
Best of Max ONMI: 0.573103 params: '-t 0.8500000000000002 -cp 0.05 -seed 10'
Best of Avarage ONMI: 0.5128860000000001 params: '-t 0.8500000000000002 -cp 0.05 -seed
10'
```

```
In [191]: plot_graph_for_all_results(all_results, "bench_60_dense", random_choice='mean')
```

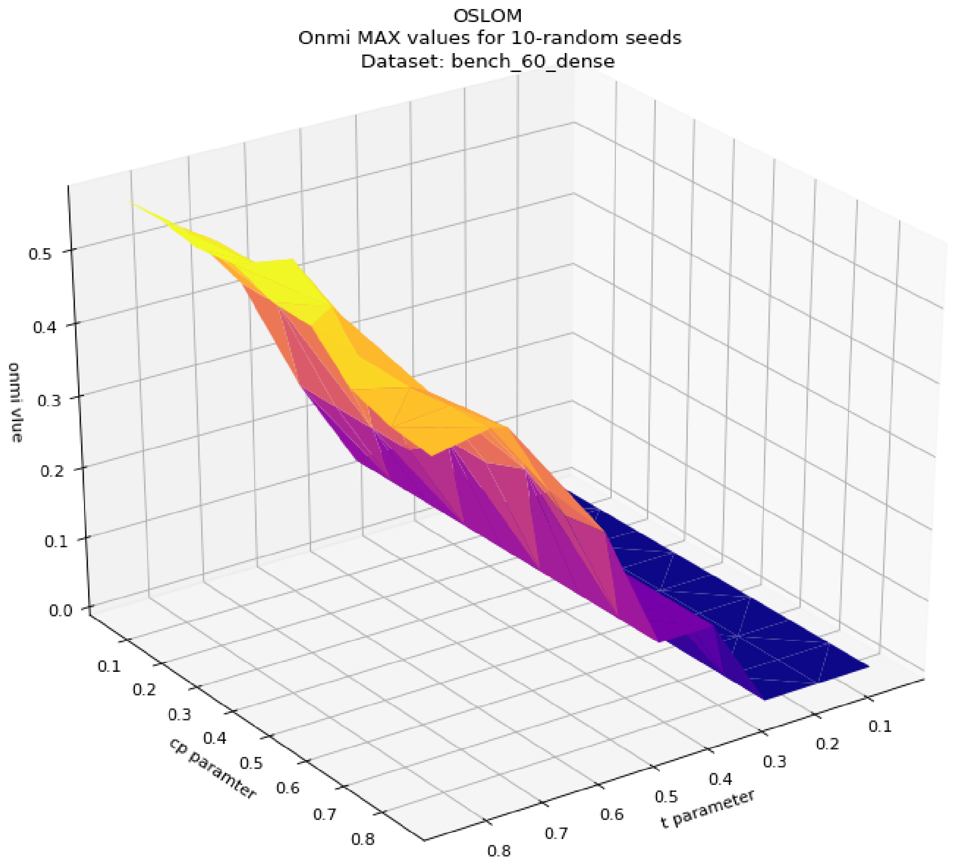
OSLOM
Onmi MAX values for 10-random seeds
Dataset: bench_60_dense
onmi vlue
0.0
0.1
0.2
0.3
0.4
0.5
cp paramter
0.1
0.2
0.3
0.4
0.5
0.6
0.7
0.8
t parameter
0.1
0.2
0.3
0.4
0.5
0.6
0.7
0.8

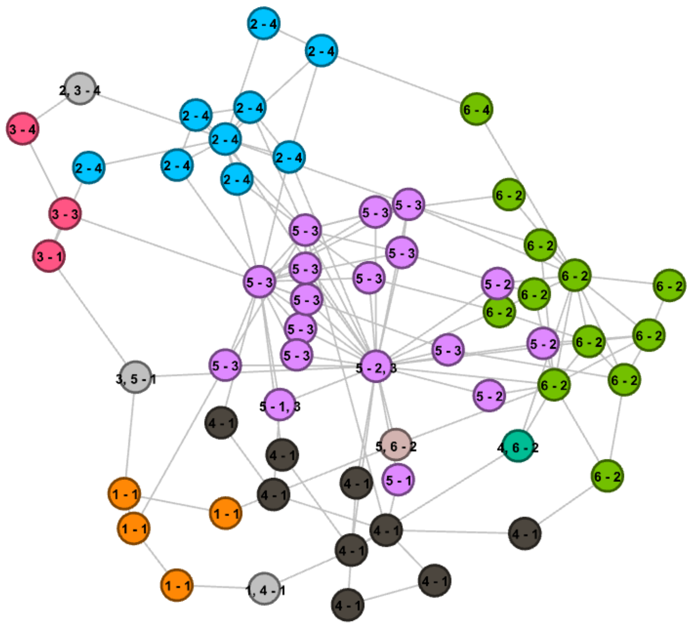

Bench_60_dense. Algorithm - OSLOM

7.6 Lattice 8x8

```
In [81]: inputFile = "../datasets/lattice_8x8/lattice_8x8.dat"
         groundTruth = "../datasets/lattice_8x8/truth_4_b.dat"
         all_results = oslom_experiment(inputFile, groundTruth, params, vertexNumerationShift=0)
```

```
mkdir: cannot create directory ‘../Results/OSLOM2_lattice_8x8’: File exists
Output dir name: ../Results/OSLOM2_lattice_8x8
old inputFileName : ../datasets/lattice_8x8/lattice_8x8.dat
new inputFileName : ../Results/OSLOM2_lattice_8x8/lattice_8x8.dat
Output file name: ../Results/OSLOM2_lattice_8x8/lattice_8x8.dat_oslo_files/tp
mkdir: cannot create directory ‘../Results/OSLOM2_lattice_8x8’: File exists
```

```
HBox(children=(IntProgress(value=0, max=810), HTML(value='')))
```

```
Best of Max ONMI: 1.0 params: '-t 0.45000000000000007 -cp 0.45000000000000007 -seed
45'
Best of Avarage ONMI: 0.7424702999999999 params: '-t 0.45000000000000007 -cp
0.45000000000000007 -seed 45'
```

```
In [82]: %matplotlib inline
         %config InlineBackend.print_figure_kwargs={'bbox_inches':None}
         plt.rcParams['figure.figsize'] = [1, 1]
```

```
In [83]: plot_graph_for_all_results(all_results, "lattice_8x8 - 4 clusters",
         random_choice='mean')
```

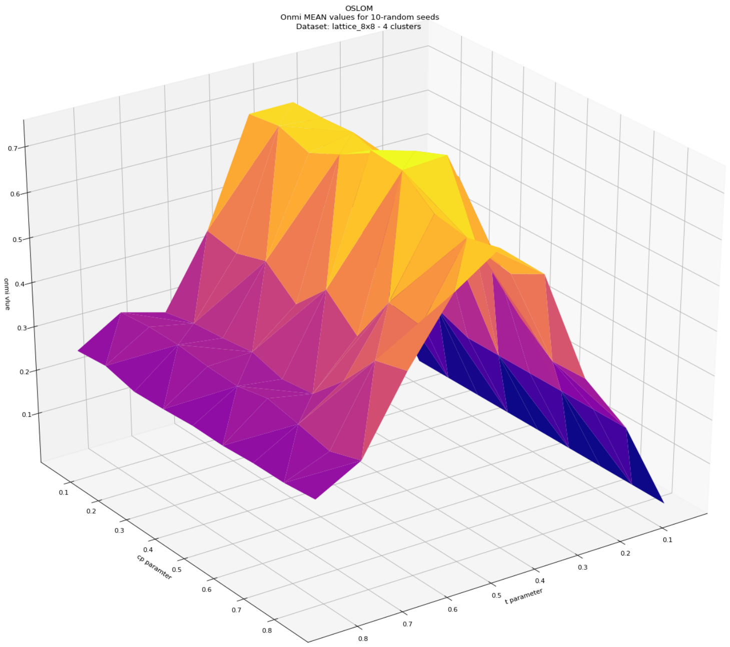


```
In [98]: !java -jar ../CommunityVisualizer/target/CommunityVisualizer-0.8-jar-with-
         dependencies.jar {inputFile} {outputFile} {outputDir}/oslom_result_c=4.gexf
```

```
Reading ground truth file ../Results/OSLOM2_lattice_8x8/lattice_8x8.dat_oslo_files/tp
...
```

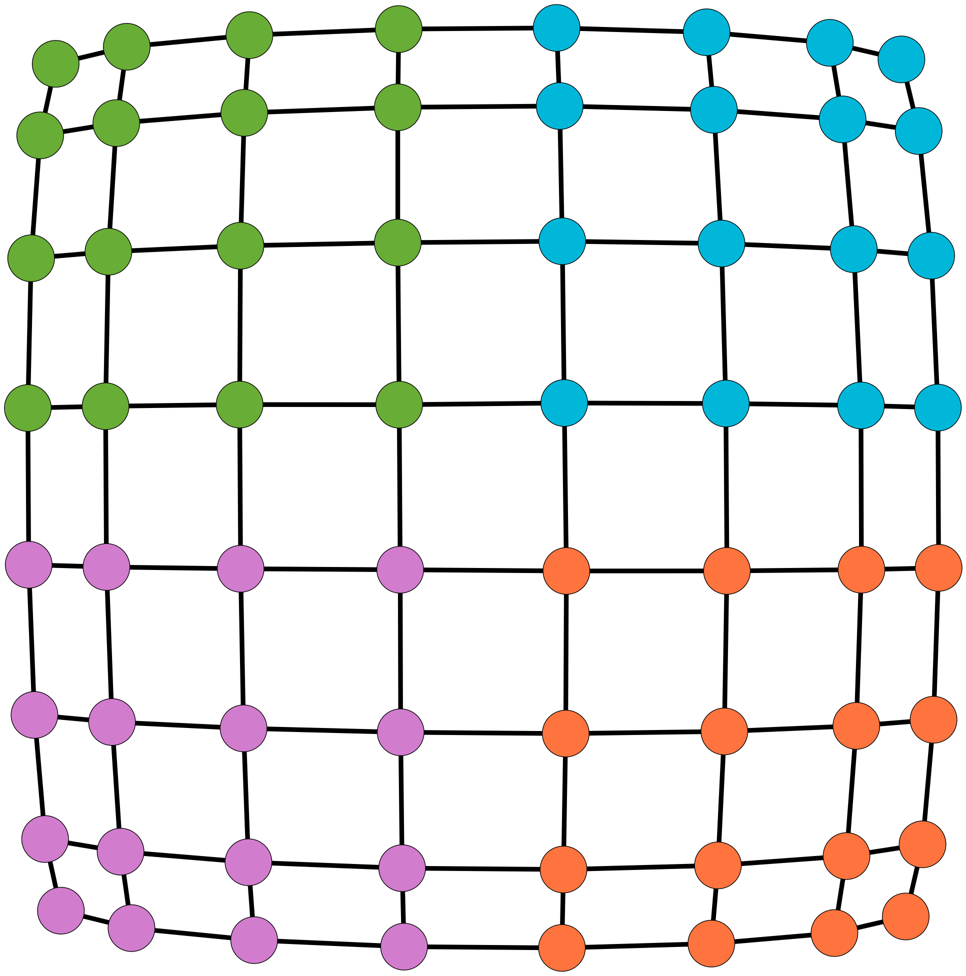

Lattice_8x8 - OSLOM

8 FARZ

8.1 FARZ_n_200_m_5_k_5_beta_1

```
In [29]: inputFile = "../datasets/FARZ_n_200_m_5_k_5_beta_1/network.dat"
         groundTruth = "../datasets/FARZ_n_200_m_5_k_5_beta_1/network.lgt"
         all_results = oslom_experiment(inputFile, groundTruth, params, vertexNumerationShift=0,
         verbose=False)
```

```
mkdir: cannot create directory '../Results/OSLOM2_FARZ_n_200_m_5_k_5_beta_1': File
exists
Output dir name: ../Results/OSLOM2_FARZ_n_200_m_5_k_5_beta_1
old inputFileName : ../datasets/FARZ_n_200_m_5_k_5_beta_1/network.dat
new inputFileName : ../Results/OSLOM2_FARZ_n_200_m_5_k_5_beta_1/network.dat
Output file name:
../Results/OSLOM2_FARZ_n_200_m_5_k_5_beta_1/network.dat_oslo_files/tp
mkdir: cannot create directory '../Results/OSLOM2_FARZ_n_200_m_5_k_5_beta_1': File
exists
```

```
HBox(children=(IntProgress(value=0, max=810), HTML(value='')))
```

```
Best of Max ONMI: 1.0 params: '-t 0.05 -cp 0.25000000000000006 -seed 60'
Best of Avarage ONMI: 1.0 params: '-t 0.05 -cp 0.25000000000000006 -seed 60'
```

```
In [30]: plot_graph_for_all_results(all_results, "FARZ_n_200_m_5_k_5_beta_1",
         random_choice='mean')
```

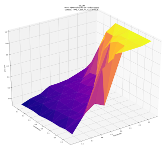

8.2 FARZ_n_200_m_5_k_5_beta_0.95

```
In [10]: inputFile = "../datasets/FARZ_n_200_m_5_k_5_beta_0.95/network.dat"
         groundTruth = "../datasets/FARZ_n_200_m_5_k_5_beta_0.95/network.lgt"
         all_results = oslom_experiment(inputFile, groundTruth, params, vertexNumerationShift=0,
         verbose=False)
```

```
mkdir: cannot create directory '../Results/OSLOM2_FARZ_n_200_m_5_k_5_beta_0.95': File
exists
Output dir name: ../Results/OSLOM2_FARZ_n_200_m_5_k_5_beta_0.95
old inputFileName : ../datasets/FARZ_n_200_m_5_k_5_beta_0.95/network.dat
new inputFileName : ../Results/OSLOM2_FARZ_n_200_m_5_k_5_beta_0.95/network.dat
Output file name:
../Results/OSLOM2_FARZ_n_200_m_5_k_5_beta_0.95/network.dat_oslo_files/tp
mkdir: cannot create directory '../Results/OSLOM2_FARZ_n_200_m_5_k_5_beta_0.95': File
exists
```

```
HBox(children=(IntProgress(value=0, max=810), HTML(value='')))
```

```
Best of Max ONMI: 0.963935 params: '-t 0.05 -cp 0.45000000000000007 -seed 90'
Best of Avarage ONMI: 0.8747809999999999 params: '-t 0.05 -cp 0.45000000000000007
-seed 90'
```

```
In [11]: plot_graph_for_all_results(all_results, "FARZ_n_200_m_5_k_5_beta_0.95",
         random_choice='mean')
```

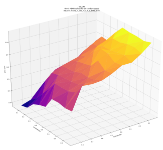

8.3 FARZ_n_200_m_5_k_5_beta_0.9

```
In [12]: inputFile = "../datasets/FARZ_n_200_m_5_k_5_beta_0.9/network.dat"
         groundTruth = "../datasets/FARZ_n_200_m_5_k_5_beta_0.9/network.lgt"
         all_results = oslom_experiment(inputFile, groundTruth, params, vertexNumerationShift=0,
         verbose=False)
```

```
Output dir name: ../Results/OSLOM2_FARZ_n_200_m_5_k_5_beta_0.9
old inputFileName : ../datasets/FARZ_n_200_m_5_k_5_beta_0.9/network.dat
new inputFileName : ../Results/OSLOM2_FARZ_n_200_m_5_k_5_beta_0.9/network.dat
Output file name:
../Results/OSLOM2_FARZ_n_200_m_5_k_5_beta_0.9/network.dat_oslo_files/tp
mkdir: cannot create directory '../Results/OSLOM2_FARZ_n_200_m_5_k_5_beta_0.9': File
exists
```

```
HBox(children=(IntProgress(value=0, max=810), HTML(value='')))
```

```
Best of Max ONMI: 0.961739 params: '-t 0.05 -cp 0.8500000000000002 -seed 60'
Best of Avarage ONMI: 0.8343755999999999 params: '-t 0.05 -cp 0.8500000000000002 -seed
60'
```

```
In [13]: plot_graph_for_all_results(all_results, "FARZ_n_200_m_5_k_5_beta_0.9",
         random_choice='mean')
```

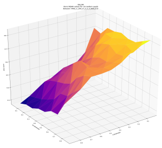

8.4 FARZ_n_200_m_5_k_5_beta_0.85

```
In [14]: inputFile = "../datasets/FARZ_n_200_m_5_k_5_beta_0.85/network.dat"
         groundTruth = "../datasets/FARZ_n_200_m_5_k_5_beta_0.85/network.lgt"
         all_results = oslom_experiment(inputFile, groundTruth, params, vertexNumerationShift=0,
         verbose=False)
```

```
Output dir name: ../Results/OSLOM2_FARZ_n_200_m_5_k_5_beta_0.85
old inputFileName : ../datasets/FARZ_n_200_m_5_k_5_beta_0.85/network.dat
new inputFileName : ../Results/OSLOM2_FARZ_n_200_m_5_k_5_beta_0.85/network.dat
Output file name:
../Results/OSLOM2_FARZ_n_200_m_5_k_5_beta_0.85/network.dat_oslo_files/tp
mkdir: cannot create directory '../Results/OSLOM2_FARZ_n_200_m_5_k_5_beta_0.85': File
exists
```

```
HBox(children=(IntProgress(value=0, max=810), HTML(value='')))
```

```
Best of Max ONMI: 0.832297 params: '-t 0.45000000000000007 -cp 0.8500000000000002
-seed 5'
Best of Avarage ONMI: 0.7033592000000001 params: '-t 0.45000000000000007 -cp
0.8500000000000002 -seed 5'
```

```
In [15]: plot_graph_for_all_results(all_results, "FARZ_n_200_m_5_k_5_beta_0.85",
         random_choice='mean')
```

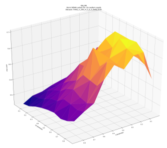

8.5 FARZ_n_200_m_5_k_5_beta_0.8

```
In [16]: inputFile = "../datasets/FARZ_n_200_m_5_k_5_beta_0.8/network.dat"
         groundTruth = "../datasets/FARZ_n_200_m_5_k_5_beta_0.8/network.lgt"
         all_results = oslom_experiment(inputFile, groundTruth, params, vertexNumerationShift=0,
         verbose=False)
```

```
Output dir name: ../Results/OSLOM2_FARZ_n_200_m_5_k_5_beta_0.8
old inputFileName : ../datasets/FARZ_n_200_m_5_k_5_beta_0.8/network.dat
new inputFileName : ../Results/OSLOM2_FARZ_n_200_m_5_k_5_beta_0.8/network.dat
Output file name:
../Results/OSLOM2_FARZ_n_200_m_5_k_5_beta_0.8/network.dat_oslo_files/tp
mkdir: cannot create directory '../Results/OSLOM2_FARZ_n_200_m_5_k_5_beta_0.8': File
exists
```

```
HBox(children=(IntProgress(value=0, max=810), HTML(value='')))
```

```
Best of Max ONMI: 0.660029 params: '-t 0.05 -cp 0.8500000000000002 -seed 60'
Best of Avarage ONMI: 0.5353789000000001 params: '-t 0.05 -cp 0.8500000000000002 -seed
60'
```

```
In [ ]: plot_graph_for_all_results(all_results, "FARZ_n_200_m_5_k_5_beta_0.95",
        random_choice='mean')
```

8.6 FARZ_n_200_m_5_k_5_beta_0.75

```
In [17]: inputFile = "../datasets/FARZ_n_200_m_5_k_5_beta_0.75/network.dat"
         groundTruth = "../datasets/FARZ_n_200_m_5_k_5_beta_0.75/network.lgt"
         all_results = oslom_experiment(inputFile, groundTruth, params, vertexNumerationShift=0,
         verbose=False)
```

```
Output dir name: ../Results/OSLOM2_FARZ_n_200_m_5_k_5_beta_0.75
old inputFileName : ../datasets/FARZ_n_200_m_5_k_5_beta_0.75/network.dat
new inputFileName : ../Results/OSLOM2_FARZ_n_200_m_5_k_5_beta_0.75/network.dat
Output file name:
```

```
../Results/OSLOM2_FARZ_n_200_m_5_k_5_beta_0.75/network.dat_oslo_files/tp
mkdir: cannot create directory ‘../Results/OSLOM2_FARZ_n_200_m_5_k_5_beta_0.75’: File
exists
```

```
HBox(children=(IntProgress(value=0, max=810), HTML(value='')))
```

```
Best of Max ONMI: 0.780434 params: '-t 0.45000000000000007 -cp 0.8500000000000002
-seed 5'
Best of Avarage ONMI: 0.5896019000000001 params: '-t 0.45000000000000007 -cp
0.8500000000000002 -seed 5'
```

```
In [18]: plot_graph_for_all_results(all_results, "FARZ_n_200_m_5_k_5_beta_0.75",
         random_choice='mean')
```

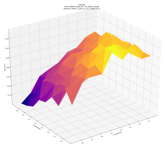

8.7 FARZ_n_200_m_5_k_5_beta_0.7

```
In [19]: inputFile = "../datasets/FARZ_n_200_m_5_k_5_beta_0.7/network.dat"
         groundTruth = "../datasets/FARZ_n_200_m_5_k_5_beta_0.7/network.lgt"
         all_results = oslom_experiment(inputFile, groundTruth, params, vertexNumerationShift=0,
         verbose=False)
```

```
Output dir name: ../Results/OSLOM2_FARZ_n_200_m_5_k_5_beta_0.7
old inputFileName : ../datasets/FARZ_n_200_m_5_k_5_beta_0.7/network.dat
new inputFileName : ../Results/OSLOM2_FARZ_n_200_m_5_k_5_beta_0.7/network.dat
Output file name:
../Results/OSLOM2_FARZ_n_200_m_5_k_5_beta_0.7/network.dat_oslo_files/tp
mkdir: cannot create directory ‘../Results/OSLOM2_FARZ_n_200_m_5_k_5_beta_0.7’: File
exists
```

```
HBox(children=(IntProgress(value=0, max=810), HTML(value='')))
```

```
Best of Max ONMI: 0.750098 params: '-t 0.45000000000000007 -cp 0.8500000000000002
-seed 60'
Best of Avarage ONMI: 0.4923299 params: '-t 0.45000000000000007 -cp 0.8500000000000002
-seed 60'
```

```
In [20]: plot_graph_for_all_results(all_results, "FARZ_n_200_m_5_k_5_beta_0.7",
         random_choice='mean')
```

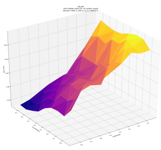

8.8 FARZ_n_200_m_5_k_5_beta_0.65

```
In [21]: inputFile = "../datasets/FARZ_n_200_m_5_k_5_beta_0.65/network.dat"
         groundTruth = "../datasets/FARZ_n_200_m_5_k_5_beta_0.65/network.lgt"
         all_results = oslom_experiment(inputFile, groundTruth, params, vertexNumerationShift=0,
         verbose=False)
```

```
Output dir name: ../Results/OSLOM2_FARZ_n_200_m_5_k_5_beta_0.65
old inputFileName : ../datasets/FARZ_n_200_m_5_k_5_beta_0.65/network.dat
new inputFileName : ../Results/OSLOM2_FARZ_n_200_m_5_k_5_beta_0.65/network.dat
Output file name:
../Results/OSLOM2_FARZ_n_200_m_5_k_5_beta_0.65/network.dat_oslo_files/tp
mkdir: cannot create directory '../Results/OSLOM2_FARZ_n_200_m_5_k_5_beta_0.65': File
exists
```

```
HBox(children=(IntProgress(value=0, max=810), HTML(value='')))
```

```
Best of Max ONMI: 0.643975 params: '-t 0.05 -cp 0.35000000000000003 -seed 71'
Best of Avarage ONMI: 0.5435125999999999 params: '-t 0.05 -cp 0.35000000000000003
-seed 71'
```

```
In [22]: plot_graph_for_all_results(all_results, "FARZ_n_200_m_5_k_5_beta_0.65",
         random_choice='mean')
```

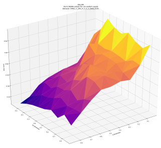

8.9 FARZ_n_200_m_5_k_5_beta_0.6

```
In [23]: inputFile = "../datasets/FARZ_n_200_m_5_k_5_beta_0.6/network.dat"
         groundTruth = "../datasets/FARZ_n_200_m_5_k_5_beta_0.6/network.lgt"
         all_results = oslom_experiment(inputFile, groundTruth, params, vertexNumerationShift=0,
         verbose=False)
```

```
Output dir name: ../Results/OSLOM2_FARZ_n_200_m_5_k_5_beta_0.6
old inputFileName : ../datasets/FARZ_n_200_m_5_k_5_beta_0.6/network.dat
new inputFileName : ../Results/OSLOM2_FARZ_n_200_m_5_k_5_beta_0.6/network.dat
Output file name:
```

```
../Results/OSLOM2_FARZ_n_200_m_5_k_5_beta_0.6/network.dat_oslo_files/tp
mkdir: cannot create directory ‘../Results/OSLOM2_FARZ_n_200_m_5_k_5_beta_0.6’: File
exists
```

```
HBox(children=(IntProgress(value=0, max=810), HTML(value='')))
```

```
Best of Max ONMI: 0.378265 params: '-t 0.25000000000000006 -cp 0.7500000000000002
-seed 32'
Best of Avarage ONMI: 0.3167039 params: '-t 0.25000000000000006 -cp 0.7500000000000002
-seed 32'
```

```
In [24]: plot_graph_for_all_results(all_results, "FARZ_n_200_m_5_k_5_beta_0.6",
         random_choice='mean')
```

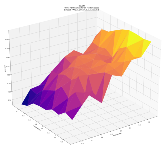

8.10 FARZ_n_200_m_5_k_5_beta_0.55

```
In [35]: inputFile = "../datasets/FARZ_n_200_m_5_k_5_beta_0.55/network.dat"
         groundTruth = "../datasets/FARZ_n_200_m_5_k_5_beta_0.55/network.lgt"
         all_results = oslom_experiment(inputFile, groundTruth, params, vertexNumerationShift=0,
         verbose=False)
```

```
mkdir: cannot create directory ‘../Results/OSLOM2_FARZ_n_200_m_5_k_5_beta_0.55’: File
exists
Output dir name: ../Results/OSLOM2_FARZ_n_200_m_5_k_5_beta_0.55
old inputFileName : ../datasets/FARZ_n_200_m_5_k_5_beta_0.55/network.dat
new inputFileName : ../Results/OSLOM2_FARZ_n_200_m_5_k_5_beta_0.55/network.dat
Output file name:
../Results/OSLOM2_FARZ_n_200_m_5_k_5_beta_0.55/network.dat_oslo_files/tp
mkdir: cannot create directory ‘../Results/OSLOM2_FARZ_n_200_m_5_k_5_beta_0.55’: File
exists
```

```
HBox(children=(IntProgress(value=0, max=810), HTML(value='')))
```

```
Best of Max ONMI: 0.482735 params: '-t 0.05 -cp 0.6500000000000001 -seed 39'
Best of Avarage ONMI: 0.3986272 params: '-t 0.05 -cp 0.6500000000000001 -seed 39'
```

Best of Max ONMI: 0.482735 params: ′-t 0.05 -cp 0.6500000000000001 -seed 39′ Best of Avarage ONMI: 0.3986272 params: ′-t 0.05 -cp 0.6500000000000001 -seed 39′

```
In [36]: plot_graph_for_all_results(all_results, "FARZ_n_200_m_5_k_5_beta_0.55",
         random_choice='mean')
```

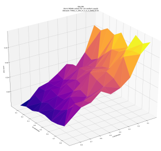

8.11 FARZ_n_200_m_5_k_5_beta_0.5

```
In [27]: inputFile = "../datasets/FARZ_n_200_m_5_k_5_beta_0.5/network.dat"
         groundTruth = "../datasets/FARZ_n_200_m_5_k_5_beta_0.5/network.lgt"
         all_results = oslom_experiment(inputFile, groundTruth, params, vertexNumerationShift=0,
         verbose=False)
```

```
Output dir name: ../Results/OSLOM2_FARZ_n_200_m_5_k_5_beta_0.5
old inputFileName : ../datasets/FARZ_n_200_m_5_k_5_beta_0.5/network.dat
new inputFileName : ../Results/OSLOM2_FARZ_n_200_m_5_k_5_beta_0.5/network.dat
Output file name:
../Results/OSLOM2_FARZ_n_200_m_5_k_5_beta_0.5/network.dat_oslo_files/tp
mkdir: cannot create directory '../Results/OSLOM2_FARZ_n_200_m_5_k_5_beta_0.5': File
exists
```

```
HBox(children=(IntProgress(value=0, max=810), HTML(value='')))
```

```
Best of Max ONMI: 0.345957 params: '-t 0.05 -cp 0.6500000000000001 -seed 71'
Best of Avarage ONMI: 0.2954157 params: '-t 0.05 -cp 0.6500000000000001 -seed 71'
```

```
In [28]: plot_graph_for_all_results(all_results, "FARZ_n_200_m_5_k_5_beta_0.5",
         random_choice='mean')
```

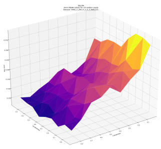

8.12 FARZ_n_1000_m_7_k_20_beta_0.9

```
In [41]: inputFile = "../datasets/FARZ_n_1000_m_7_k_20_beta_0.9/network.dat"
         groundTruth = "../datasets/FARZ_n_1000_m_7_k_20_beta_0.9/network.lgt"
         all_results = oslom_experiment(inputFile, groundTruth, params, vertexNumerationShift=0,
         verbose=False)
```

```
Output dir name: ../Results/OSLOM2_FARZ_n_1000_m_7_k_20_beta_0.9
old inputFileName : ../datasets/FARZ_n_1000_m_7_k_20_beta_0.9/network.dat
new inputFileName : ../Results/OSLOM2_FARZ_n_1000_m_7_k_20_beta_0.9/network.dat
Output file name:
../Results/OSLOM2_FARZ_n_1000_m_7_k_20_beta_0.9/network.dat_oslo_files/tp
mkdir: cannot create directory '../Results/OSLOM2_FARZ_n_1000_m_7_k_20_beta_0.9': File
exists
```

```
HBox(children=(IntProgress(value=0, max=810), HTML(value='')))
```

```
Best of Max ONMI: 0.947293 params: '-t 0.05 -cp 0.7500000000000002 -seed 5'
Best of Avarage ONMI: 0.8877864000000001 params: '-t 0.05 -cp 0.7500000000000002 -seed
5'
```

```
In [42]: plot_graph_for_all_results(all_results, "FARZ_n_1000_m_7_k_20_beta_0.9",
         random_choice='mean')
```

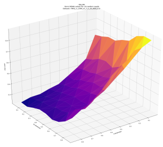

9 CKB and CKB-t

9.1 CKB_n_200

```
In [32]: inputFile = "../datasets/CKB_n_200/dl_edges_tabs.txt"
         groundTruth = "../datasets/CKB_n_200/dl_coms.txt"
         all_results = oslom_experiment(inputFile, groundTruth, params, vertexNumerationShift=0,
         verbose=False)
```

```
Output dir name: ../Results/OSLOM2_CKB_n_200
old inputFileName : ../datasets/CKB_n_200/dl_edges_tabs.txt
new inputFileName : ../Results/OSLOM2_CKB_n_200/dl_edges_tabs.txt
Output file name: ../Results/OSLOM2_CKB_n_200/dl_edges_tabs.txt_oslo_files/tp
mkdir: cannot create directory ‘../Results/OSLOM2_CKB_n_200’: File exists
```

```
HBox(children=(IntProgress(value=0, max=810), HTML(value='')))
```

```
Best of Max ONMI: 0.19299 params: '-t 0.8500000000000002 -cp 0.15000000000000002 -seed
89'
Best of Avarage ONMI: 0.1728658 params: '-t 0.8500000000000002 -cp 0.15000000000000002
-seed 89'
```

```
In [33]: plot_graph_for_all_results(all_results, "CKB_n_200", random_choice='mean')
```

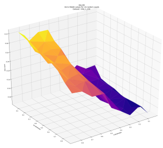

9.2 CKB-t_n_200_alfa_0.1_gamma_0.5

```
In [37]: inputFile = "../datasets/CKB-t_n_200_alfa_0.1_gamma_0.5/dl_edges_tabs.txt"
         groundTruth = "../datasets/CKB-t_n_200_alfa_0.1_gamma_0.5/dl_coms.txt"
         all_results = oslom_experiment(inputFile, groundTruth, params, vertexNumerationShift=0,
         verbose=False)
```

```
Output dir name: ../Results/OSLOM2_CKB-t_n_200_alfa_0.1_gamma_0.5
old inputFileName : ../datasets/CKB-t_n_200_alfa_0.1_gamma_0.5/dl_edges_tabs.txt
new inputFileName : ../Results/OSLOM2_CKB-t_n_200_alfa_0.1_gamma_0.5/dl_edges_tabs.txt
Output file name: ../Results/OSLOM2_CKB-
t_n_200_alfa_0.1_gamma_0.5/dl_edges_tabs.txt_oslo_files/tp
mkdir: cannot create directory '../Results/OSLOM2_CKB-t_n_200_alfa_0.1_gamma_0.5':
File exists
```

```
HBox(children=(IntProgress(value=0, max=810), HTML(value='')))
```

```
Best of Max ONMI: 0.0626467 params: '-t 0.7500000000000002 -cp 0.35000000000000003
-seed 90'
Best of Avarage ONMI: 0.04850417 params: '-t 0.7500000000000002 -cp
0.35000000000000003 -seed 90'
```

```
In [38]: plot_graph_for_all_results(all_results, "CKB-t_n_200_alfa_0.1_gamma_0.5",
         random_choice='mean')
```

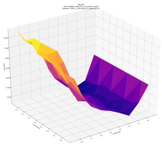

9.3 CKB-t_n_1000_alfa_0.1_gamma_0.5_max_memb_20_max_com_size_200

```
In [ ]: inputFile = "../datasets/CKB-
        t_n_1000_alfa_0.1_gamma_0.5_max_memb_20_max_com_size_200/dl_edges_tabs.txt"
        groundTruth = "../datasets/CKB-
        t_n_1000_alfa_0.1_gamma_0.5_max_memb_20_max_com_size_200/dl_coms.txt"
        all_results = oslom_experiment(inputFile, groundTruth, params, vertexNumerationShift=0,
        verbose=False)
```

```
mkdir: cannot create directory '../Results/OSLOM2_CKB-
t_n_1000_alfa_0.1_gamma_0.5_max_memb_20_max_com_size_200': File exists
Output dir name: ../Results/OSLOM2_CKB-
t_n_1000_alfa_0.1_gamma_0.5_max_memb_20_max_com_size_200
old inputFileName : ../datasets/CKB-
t_n_1000_alfa_0.1_gamma_0.5_max_memb_20_max_com_size_200/dl_edges_tabs.txt
new inputFileName : ../Results/OSLOM2_CKB-
t_n_1000_alfa_0.1_gamma_0.5_max_memb_20_max_com_size_200/dl_edges_tabs.txt
Output file name: ../Results/OSLOM2_CKB-t_n_1000_alfa_0.1_gamma_0.5_max_memb_20_max_co
m_size_200/dl_edges_tabs.txt_oslo_files/tp
mkdir: cannot create directory '../Results/OSLOM2_CKB-
t_n_1000_alfa_0.1_gamma_0.5_max_memb_20_max_com_size_200': File exists
```

```
HBox(children=(IntProgress(value=0, max=810), HTML(value='')))
```

```
In [ ]: plot_graph_for_all_results(all_results, "CKB-
        t_n_1000_alfa_0.1_gamma_0.5_max_memb_20_max_com_size_200", random_choice='mean')

In [ ]:
```

10 Lets try to build confidence intervals

```
In [49]: params={}
         # '-t 0.05 -cp 0.2
         # params["-t"] = np.arange(0.05, 0.95, 0.1)
         # params["-cp"] = np.arange(0.05, 0.95, 0.1)
         params["-t"] = [0.05]
         params["-cp"] = [0.2]
         params["-seed"] = range(1, 100)
         # params["-seed"] = random.sample(range(1, 100), 100)

In [ ]: inputFile = "../datasets/FARZ_n_200_m_5_k_5_beta_1/network.dat"
        groundTruth = "../datasets/FARZ_n_200_m_5_k_5_beta_1/network.lgt"
        all_results = oslom_experiment(inputFile, groundTruth, params, vertexNumerationShift=0,
        verbose=False)
```

```
mkdir: cannot create directory '../Results/OSLOM2_FARZ_n_200_m_5_k_5_beta_1': File
exists
Output dir name: ../Results/OSLOM2_FARZ_n_200_m_5_k_5_beta_1
old inputFileName : ../datasets/FARZ_n_200_m_5_k_5_beta_1/network.dat
new inputFileName : ../Results/OSLOM2_FARZ_n_200_m_5_k_5_beta_1/network.dat
Output file name:
../Results/OSLOM2_FARZ_n_200_m_5_k_5_beta_1/network.dat_oslo_files/tp
mkdir: cannot create directory '../Results/OSLOM2_FARZ_n_200_m_5_k_5_beta_1': File
exists
```

```
HBox(children=(IntProgress(value=0, max=99), HTML(value='')))
```

```
In [55]: import seaborn as sns
         from scipy import stats

In [72]: sns.distplot(list(all_results.values()), rug=True, bins=20)

Out[72]: <matplotlib.axes._subplots.AxesSubplot at 0x7f1b13cae128>
```

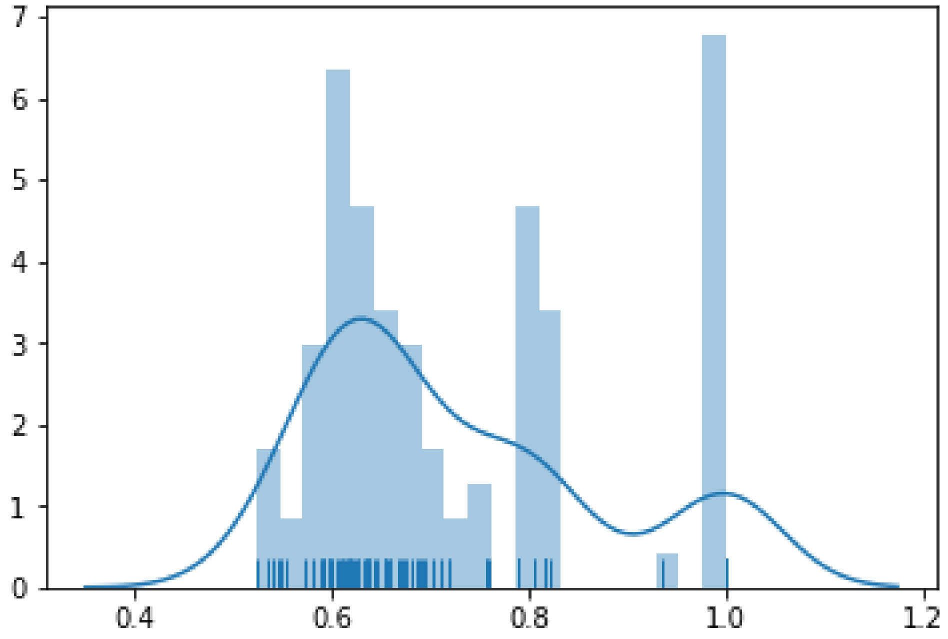


```
In [76]: import bootstrapped.bootstrap as bs
         import bootstrapped.stats_functions as bs_stats

In [73]: sns.utils.ci(sns.algorithms.bootstrap(list(all_results.values())))

Out[73]: array([0.70008461, 0.75711889])

In [ ]:
```
