# Supplementary material for: Overlapping community detection in networks based on link partitioning and partitioning around medoids
Source: PLoS One. 2021 Aug 25;16(8):e0255717. doi: 10.1371/journal.pone.0255717 (PMC8386890; doi:10.1371/journal.pone.0255717)
Supplement: S7 Appendix — The computational results for the COPRA method. (PDF) [file pone.0255717.s007.pdf]

Copra

July 18, 2019

1 COPRA

In [25]:
```
import numpy as np
import random
random.seed = 108
from tqdm import tqdm_notebook as tqdm
import seaborn as sns
import matplotlib.pyplot as plt
from mpl_toolkits import mplot3d
import pandas as pd
%matplotlib inline
```

In [26]:
```
params={}
params["-v"] = list(range(1,10))
```

In [108]:
```
params
```

Out[108]: {'-v': [1, 2, 3, 4, 5, 6, 7, 8, 9]}

In [ ]:
```
java -cp ../../related_methods/OSLOM2/copra.jar --help
```

In [28]:
```
def generate_params(params):
    keys = list(params.keys())
    if len(keys) == 1:
        for value in params[keys[0]]:
            yield ( keys[0] + " " + str(value) )
    if len( keys ) > 1:
        for value in params[keys[0]]:
            for remain_params in generate_params({k:params[k] for k in keys[1:]}):
                yield ( keys[0] + " " + str(value) + " " + remain_params )
```

In [154]:
```
def copra_experiment(inputFile, groundTruth, params = {}, vertexNumerationShift=0,
verbose=False):
    datasetName = inputFile.split('/')[-2]
    outputDir = "../Results/COPRA_{}".format(datasetName)
    outputFile = outputDir + "/" + "clusters-" + inputFile.split('/')[-1]
    print("Output dir name: {}".format(outputDir) )
    print("Output file name: {}".format(outputFile) )

    !rm -rf {outputDir}
    !mkdir {outputDir}

    all_results = {}
    bestParam = "not found"
    nmi_best = 0;
    for param in tqdm(list(generate_params(params))):
        tmp=!(cd {outputDir} && java -cp ../../related_methods/OSLOM2/copra.jar COPRA
../{inputFile} {param} -repeat 100 -mo -nosplit)
#     !sed -i '' '/^#/d' {outputFile}
        with open(outputFile) as f:
```

```
                lines = f.readlines()
            with open(outputFile, 'w') as the_file:
                for line in lines:
                    the_file.write(" ".join([str(int(a) + vertexNumerationShift) for a in
line.split()]) + "\n")
            output=!../Overlapping-NMI/onmi {groundTruth} {outputFile}
            if verbose:
                print(output)
            nmi=float(output[0].split()[1])
            all_results[param] = nmi
            if nmi >  nmi_best:
                !cp {outputFile} {outputFile}_best
                bestParam = param
                nmi_best = nmi

    print("Best ONMI: {} params: '{}'".format(nmi_best, bestParam) )
    print("Avg ONMI: {} ".format(np.mean(list(all_results.values()))))
    !java -jar ../CommunityVisualizer/target/CommunityVisualizer-0.8-jar-with-
dependencies.jar {inputFile} {outputFile}_best  {'.'.join(outputFile.split('.')[:-1] +
['gexf'])} {groundTruth}
    return all_results
```

In [30]:
```
def plot_graph_for_all_results(all_results, datasetName):
    xdata=[]
    ydata=[]
    df = pd.DataFrame()
    for param, nmi in all_results.items():
        splited = param.split()
        xdata.append(float(splited[1]))
        ydata.append(nmi)
        df = df.append({'x': float(splited[1]), 'y': nmi}, ignore_index=True)

    plt.plot(xdata, ydata, 'C3', zorder=1, lw=3)
    # ax = plt.axes(projection='3d')
    plt.scatter(xdata, ydata,s=70,zorder=2)
    plt.xlabel('v parameter')
    plt.ylabel('nmi value');
    plt.title('onmi values for dataset: {}\nAlgorithm: "COPRA" no option
"extrasimplify"'.format(datasetName));
    plt.show()
```

2 School Friendship

In [163]:
```
inputFile = "../datasets/school_friendship/school-2.dat"
groundTruth = "../datasets/school_friendship/truth-school.dat"
all_results = copra_experiment(inputFile, groundTruth, params, vertexNumerationShift=-1)
```

```
Output dir name: ../Results/COPRA_school_friendship
Output file name: ../Results/COPRA_school_friendship/clusters-school-2.dat
```

```
HBox(children=(IntProgress(value=0, max=9), HTML(value='')))
```

```
Best ONMI: 0.64575 params: '-v 1'
```

In [164]:
```
plot_graph_for_all_results(all_results, "school_friendship")
```

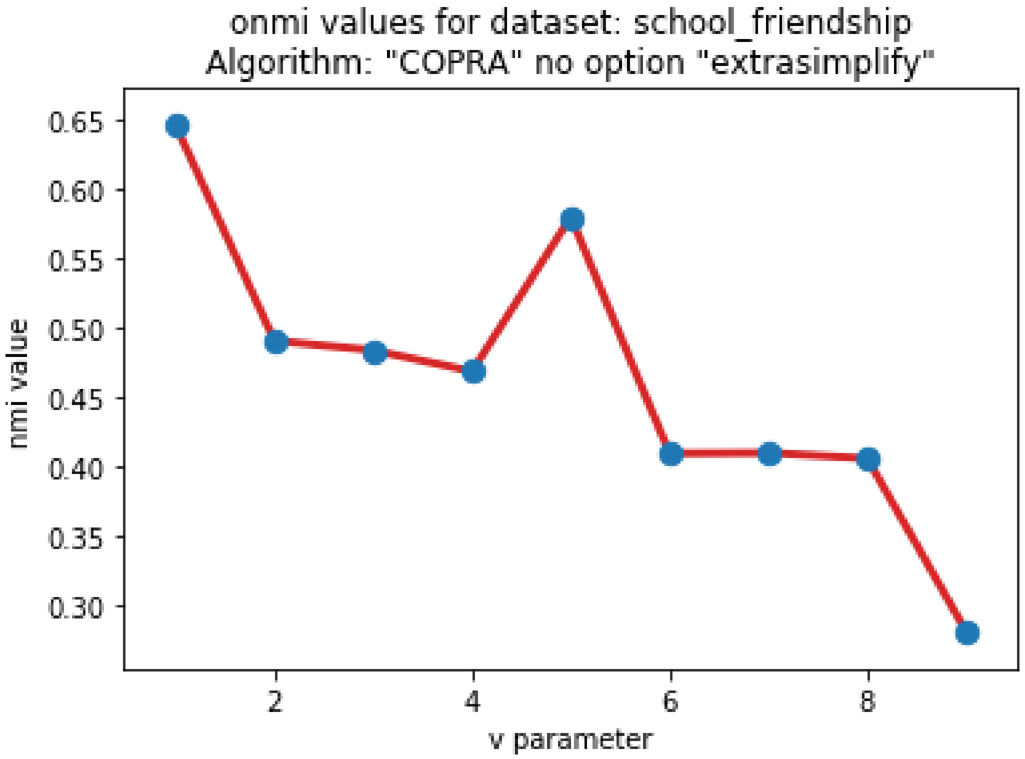

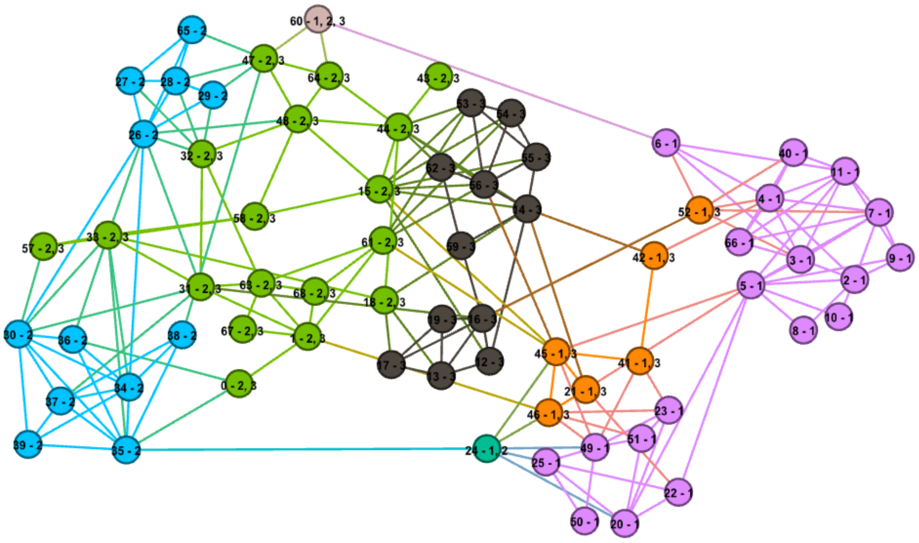

School Friendship. Algorithm - Copra

3 Karate Club

```
In [173]: inputFile = "../datasets/karate/karate.dat"
          groundTruth = "../datasets/karate/truth_karate.dat"
          all_results = copra_experiment(inputFile, groundTruth, params, vertexNumerationShift=0)
```

```
Output dir name: ../Results/COPRA_karate
Output file name: ../Results/COPRA_karate/clusters-karate.dat
mkdir: cannot create directory '../Results/COPRA_karate': File exists
```

```
HBox(children=(IntProgress(value=0, max=9), HTML(value='')))
```

```
Best ONMI: 0.590609 params: '-v 5'
```

```
In [166]: plot_graph_for_all_results(all_results, "Karate club")
```

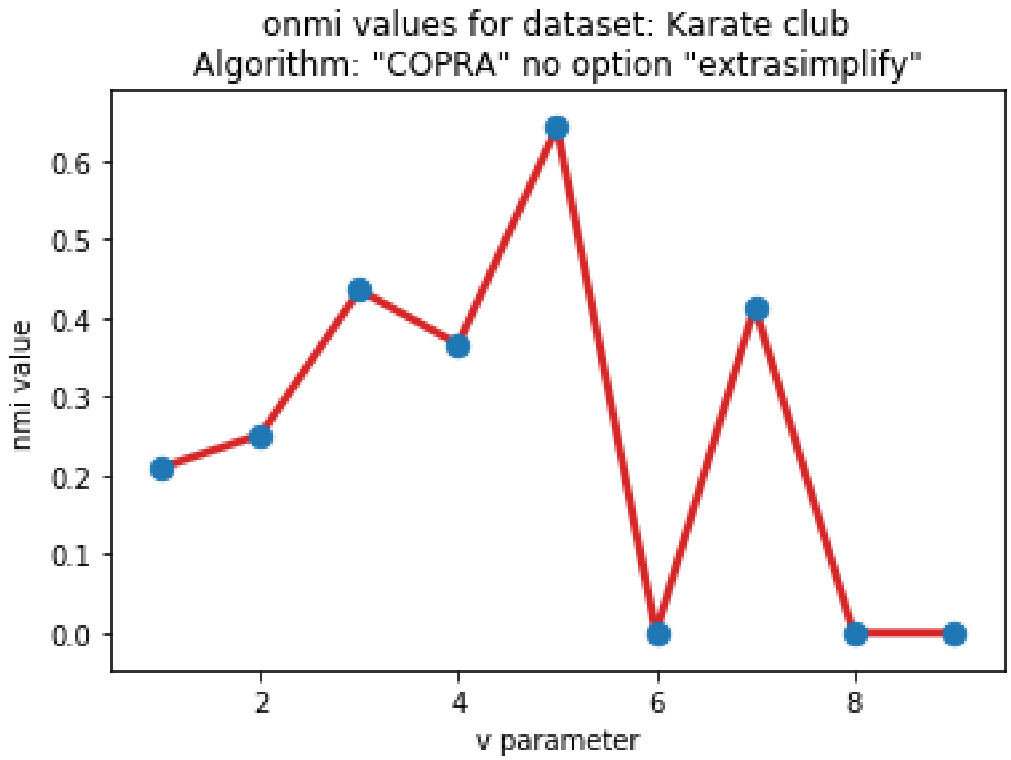

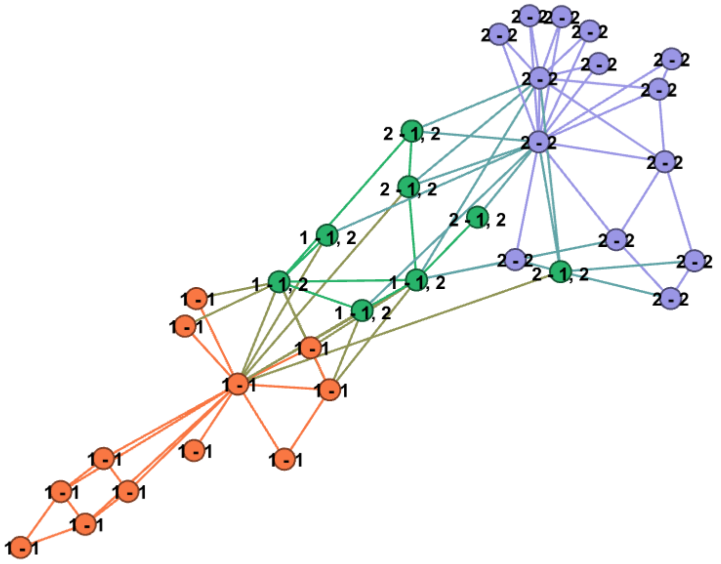

Karate Club. Algorithm - COPRA

4 Adj noun

```
In [167]: inputFile = "../datasets/adjnoun/adjnoun.dat"
          groundTruth = "../datasets/adjnoun/truth_adjnoun.dat"
          all_results = copra_experiment(inputFile, groundTruth, params, vertexNumerationShift=-1)
```

```
Output dir name: ../Results/COPRA_adjnoun
Output file name: ../Results/COPRA_adjnoun/clusters-adjnoun.dat
mkdir: cannot create directory ‘../Results/COPRA_adjnoun’: File exists
```

```
HBox(children=(IntProgress(value=0, max=9), HTML(value='')))
```

```
Best ONMI: 0.00463105 params: '-v 1'
```

```
In [168]: plot_graph_for_all_results(all_results, "Adj Noun")
```

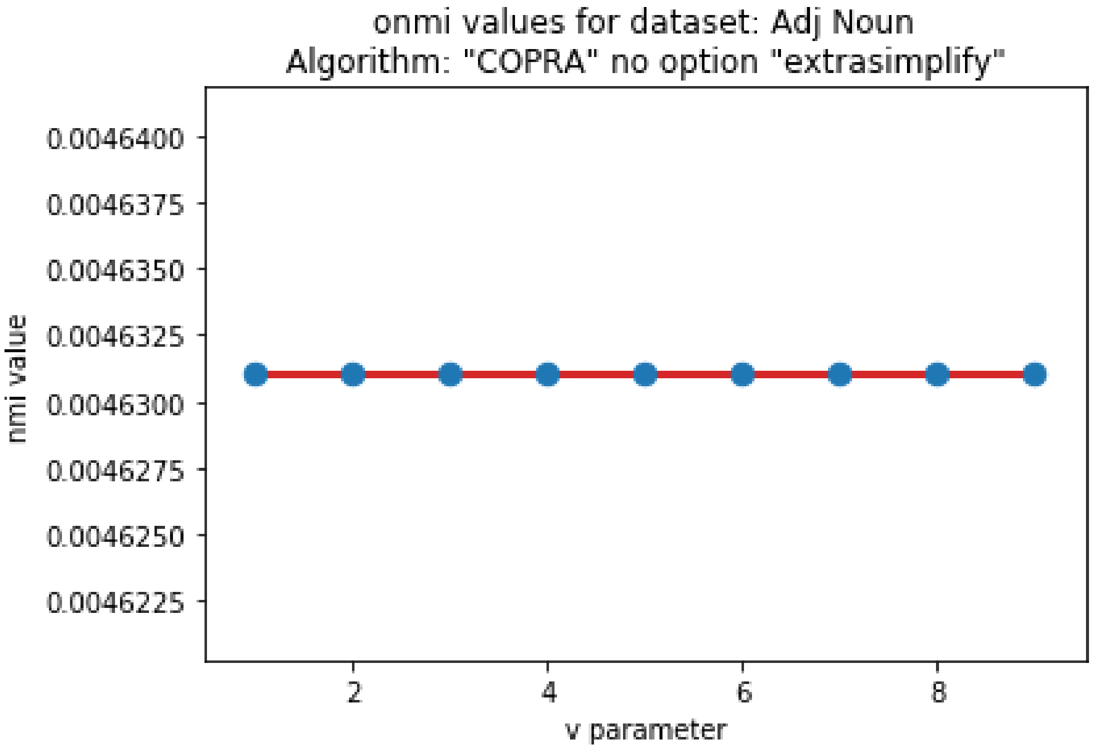

5 American Football League

```
In [169]: inputFile = "../datasets/football/footballTSEinput_original.dat"
          groundTruth = "../datasets/football/truth_footballTSEinput.dat"
          all_results = copra_experiment(inputFile, groundTruth, params, vertexNumerationShift=-1)
```

```
Output dir name: ../Results/COPRA_football
Output file name: ../Results/COPRA_football/clusters-footballTSEinput_original.dat
mkdir: cannot create directory ‘../Results/COPRA_football’: File exists
```

```
HBox(children=(IntProgress(value=0, max=9), HTML(value='')))
```

```
Best ONMI: 0.853344 params: '-v 2'
```

```
In [170]: plot_graph_for_all_results(all_results, "# American Football League #")
```

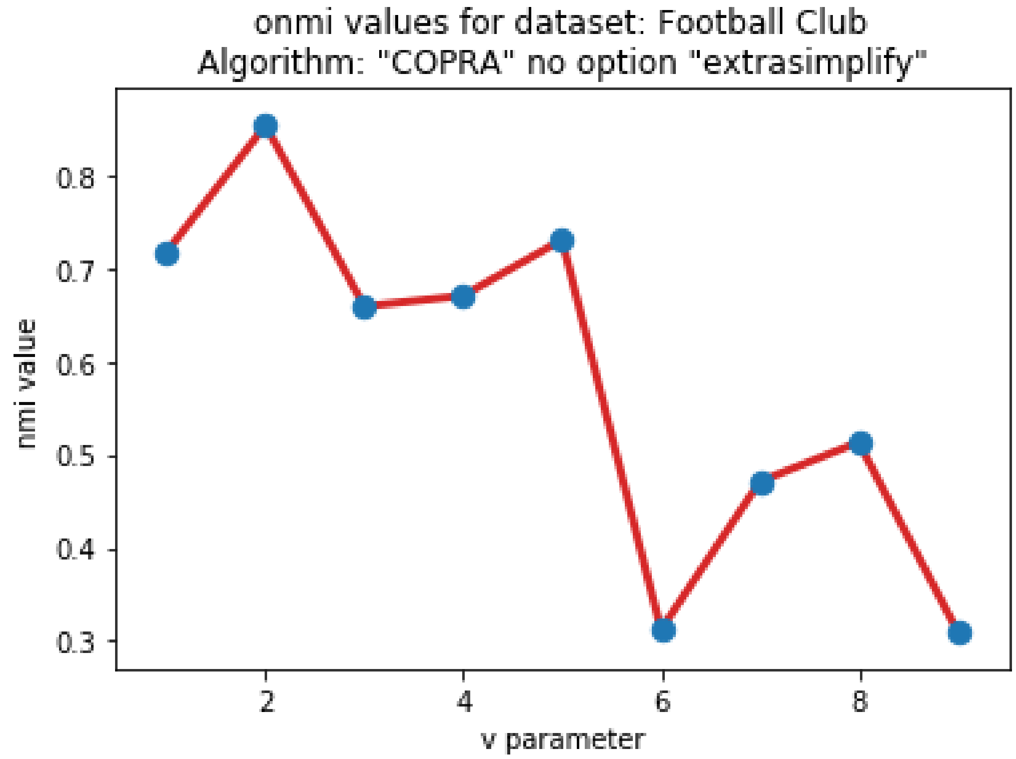

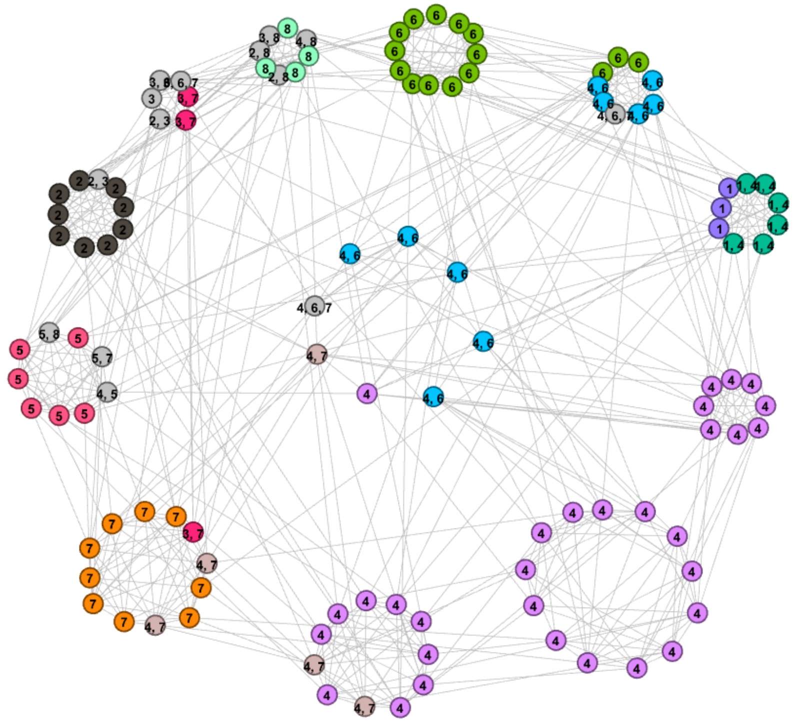

American Football League. Algoritm - COPRA

6 Political Books

```
In [171]: inputFile = "../datasets/polbooks/polbooks.dat"
          groundTruth = "../datasets/polbooks/truth_polbooks.dat"
          all_results = copra_experiment(inputFile, groundTruth, params, vertexNumerationShift=0)
```

```
Output dir name: ../Results/COPRA_polbooks
Output file name: ../Results/COPRA_polbooks/clusters-polbooks.dat
mkdir: cannot create directory '../Results/COPRA_polbooks': File exists
```

```
HBox(children=(IntProgress(value=0, max=9), HTML(value='')))
```

```
Best ONMI: 0.409727 params: '-v 2'
```

```
In [172]: plot_graph_for_all_results(all_results, "Politics Book")
```

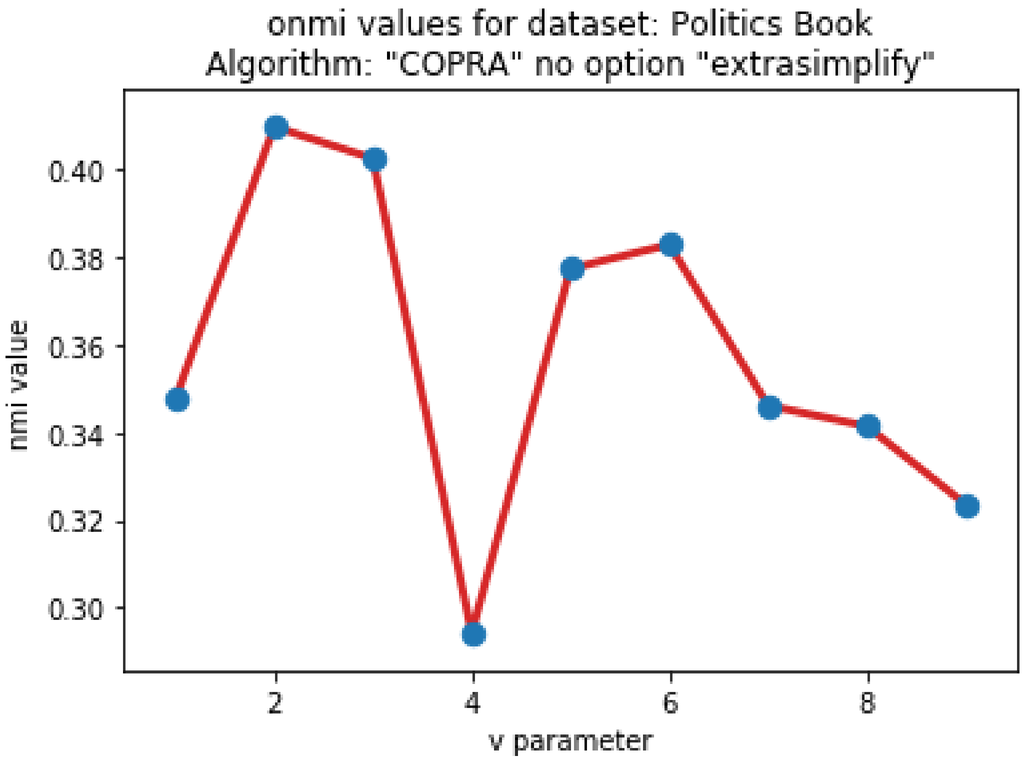

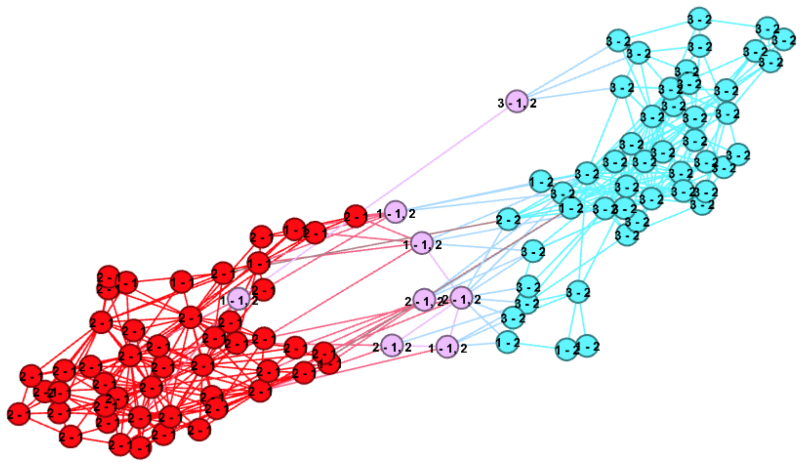

Political Books. Algorithm - COPRA

7 Syntetic Datasets

7.1 bench_30

```
In [155]: inputFile = "../datasets/bench_30/bench_30_network.dat"
          groundTruth = "../datasets/bench_30/bench_30_truth.dat"
          all_results = copra_experiment(inputFile, groundTruth, params, vertexNumerationShift=0)
```

```
Output dir name: ../Results/COPRA_bench_30
Output file name: ../Results/COPRA_bench_30/clusters-bench_30_network.dat
```

```
HBox(children=(IntProgress(value=0, max=9), HTML(value='')))
```

```
Best ONMI: 0.703095 params: '-v 2'
Avg ONMI: 0.26570044444444446
Reading  GroundTruth from file: ../datasets/bench_30/bench_30_truth.dat
Reading  ClusteringResult from file: ../Results/COPRA_bench_30/clusters-
bench_30_network.dat_best
```

```
In [156]: plot_graph_for_all_results(all_results, "bench_30")
```

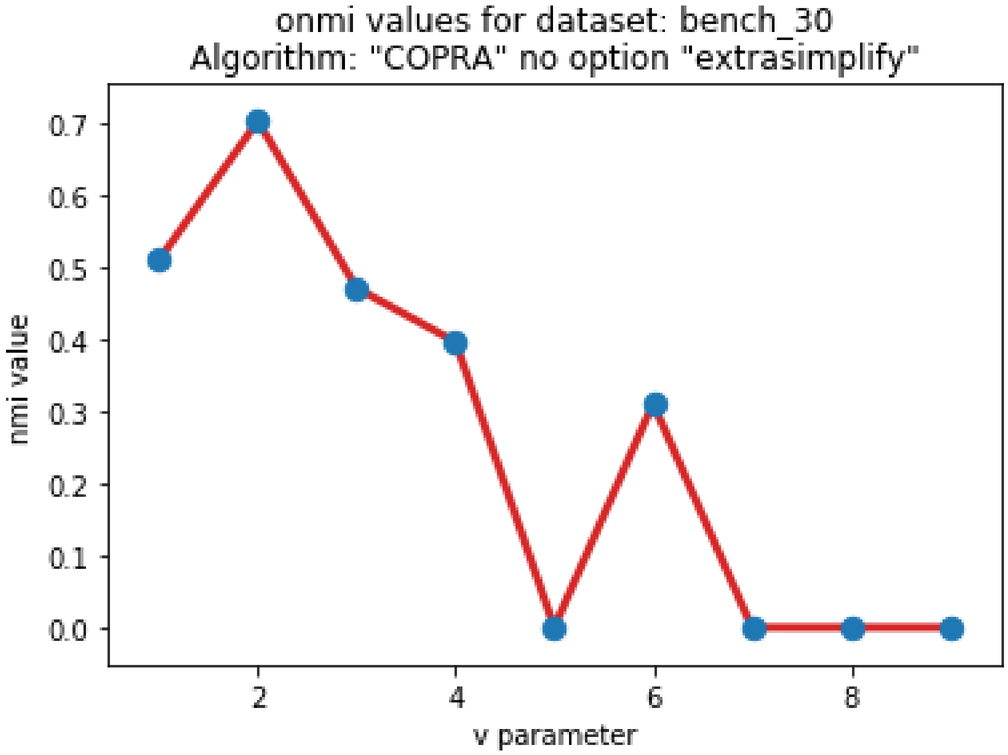
onmi values for dataset: bench_30
Algorithm: "COPRA" no option "extrasimplify"
nmi value
0.0
0.1
0.2
0.3
0.4
0.5
0.6
0.7
v parameter
2
4
6
8

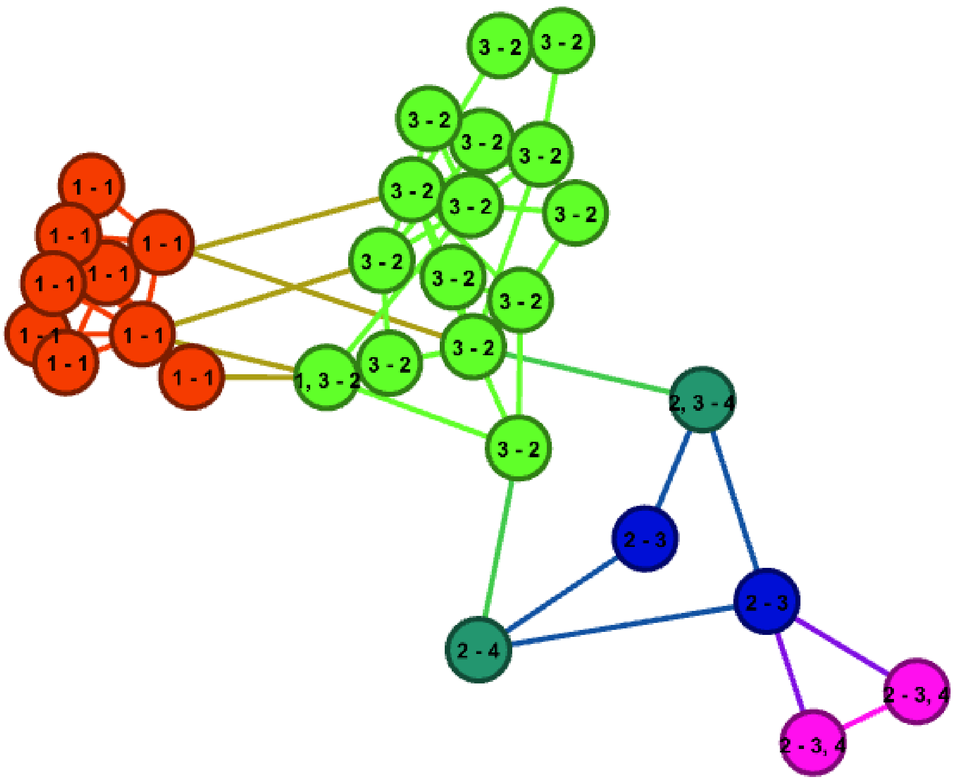

bench_30

7.2 bench_40

```
In [157]: inputFile = "../datasets/bench_40/bench_40_network.dat"
          groundTruth = "../datasets/bench_40/bench_40_truth.dat"
          all_results = copra_experiment(inputFile, groundTruth, params, vertexNumerationShift=0)
```

```
Output dir name: ../Results/COPRA_bench_40
Output file name: ../Results/COPRA_bench_40/clusters-bench_40_network.dat
```

```
HBox(children=(IntProgress(value=0, max=9), HTML(value='')))
```

```
Best ONMI: 0.381153 params: '-v 3'
Avg ONMI: 0.10586444444444444
Reading  GroundTruth from file: ../datasets/bench_40/bench_40_truth.dat
Reading  ClusteringResult from file: ../Results/COPRA_bench_40/clusters-
bench_40_network.dat_best
```

```
In [158]: plot_graph_for_all_results(all_results, "bench_40")
```

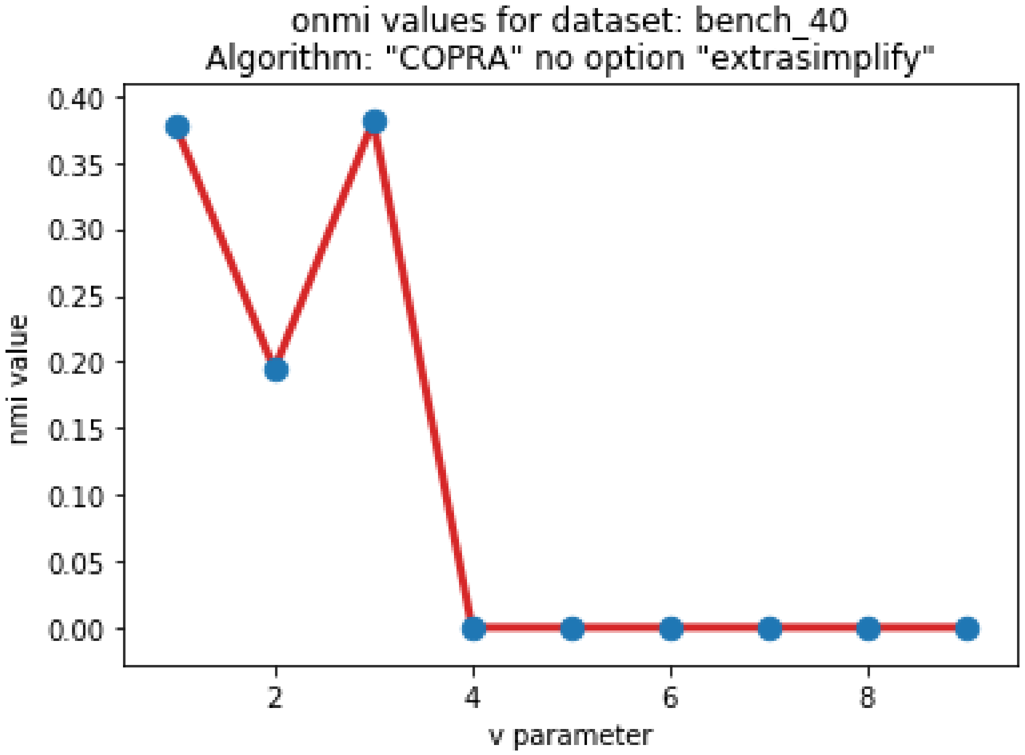
onmi values for dataset: bench_40
Algorithm: "COPRA" no option "extrasimplify"
0.40
0.35
0.30
0.25
0.20
0.15
0.10
0.05
0.00
nmi value
2
4
6
8
v parameter

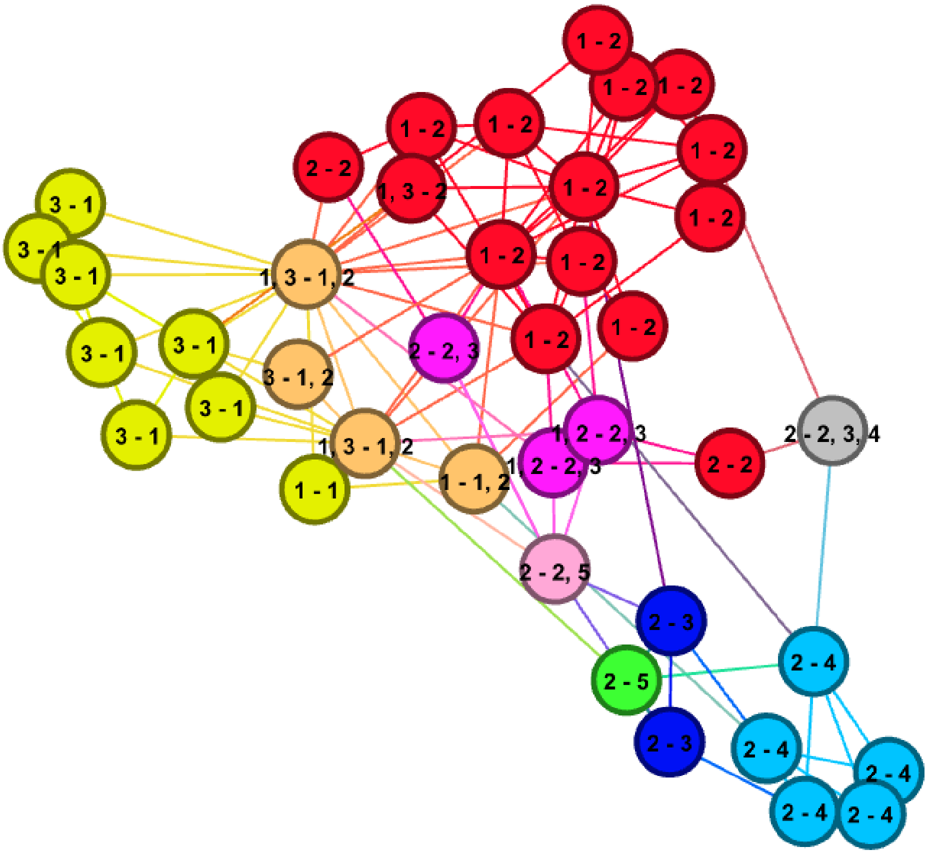

Bench_40. Algorith - COPRA

7.3 bench_50

```
In [159]: inputFile = "../datasets/bench_50/bench_50_network.dat"
          groundTruth = "../datasets/bench_50/bench_50_truth.dat"
          all_results = copra_experiment(inputFile, groundTruth, params, vertexNumerationShift=0)
```

```
Output dir name: ../Results/COPRA_bench_50
Output file name: ../Results/COPRA_bench_50/clusters-bench_50_network.dat
```

```
HBox(children=(IntProgress(value=0, max=9), HTML(value='')))
```

```
Best ONMI: 0.509912 params: '-v 1'
Avg ONMI: 0.24592011111111114
Reading  GroundTruth from file: ../datasets/bench_50/bench_50_truth.dat
Reading  ClusteringResult from file: ../Results/COPRA_bench_50/clusters-
bench_50_network.dat_best
```

```
In [160]: plot_graph_for_all_results(all_results, "bench_50")
```

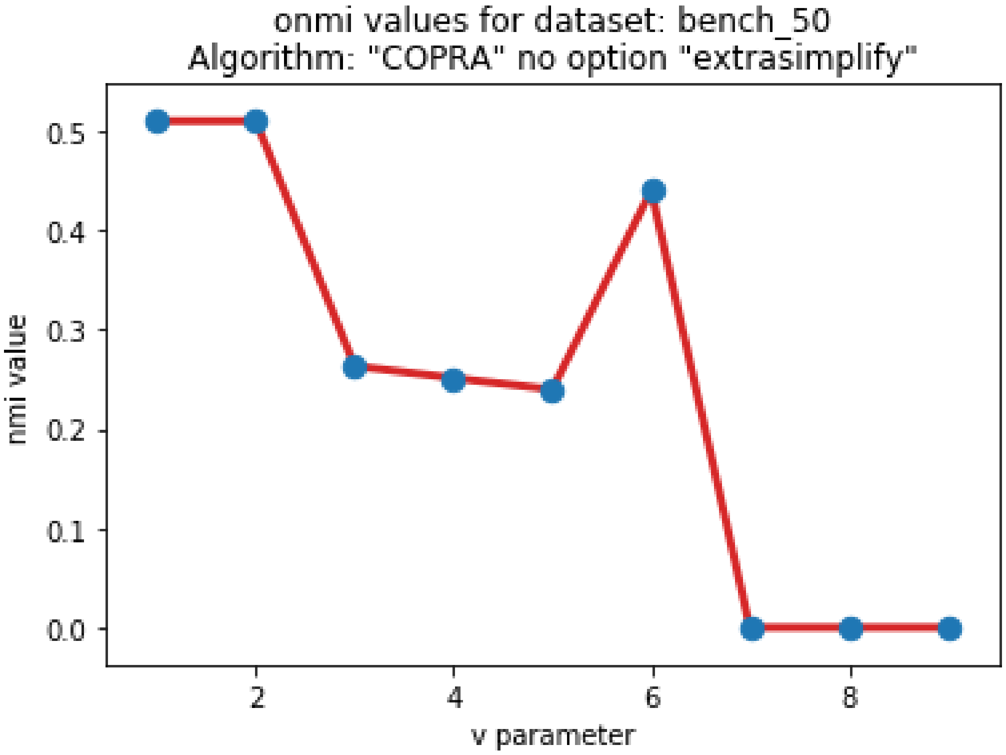

onmi values for dataset: bench_50
Algorithm: "COPRA" no option "extrasimplify"
nmi value
v parameter
0.0
0.1
0.2
0.3
0.4
0.5
2
4
6
8

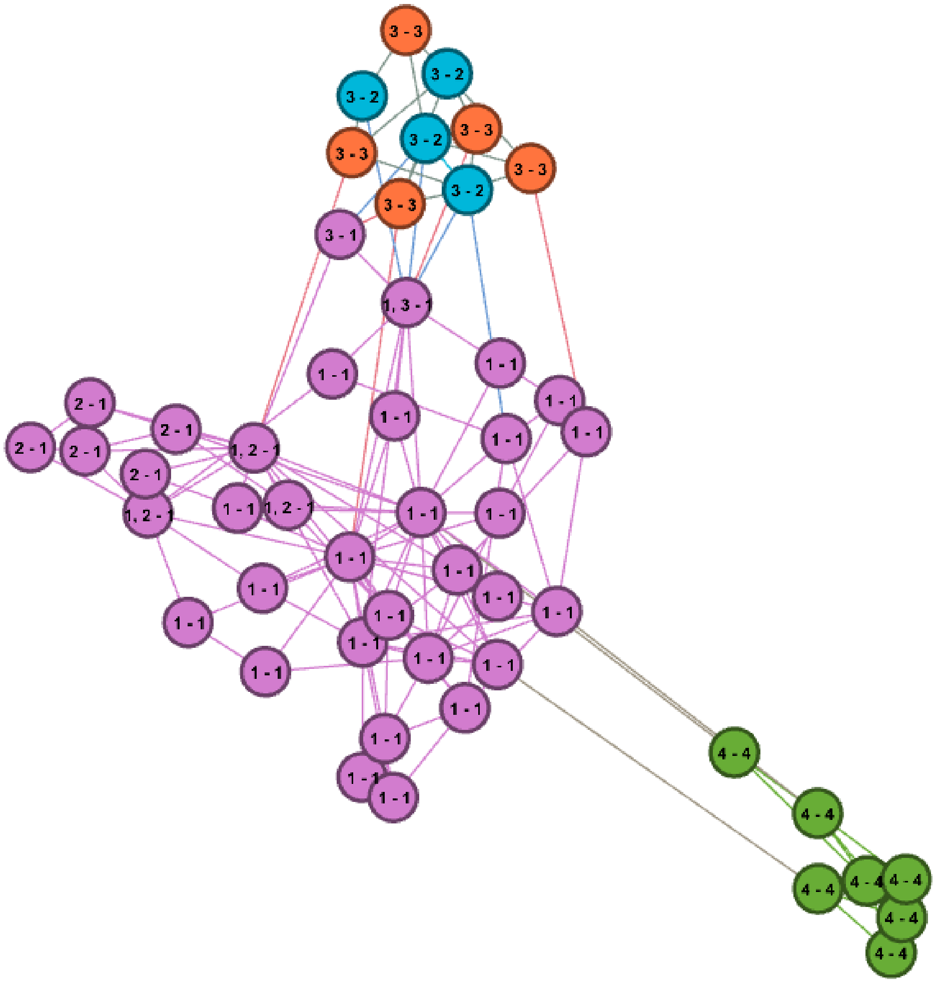

Bench_50. Algorithm - COPRA

7.4 bench_60

```
In [17]: inputFile = "../datasets/bench_60/bench_60_network.dat"
         groundTruth = "../datasets/bench_60/bench_60_truth.dat"
         all_results = copra_experiment(inputFile, groundTruth, params, vertexNumerationShift=0)
```

```
Output dir name: ../Results/COPRA_bench_60
Output file name: ../Results/COPRA_bench_60/clusters-bench_60_network.dat
mkdir: cannot create directory '../Results/COPRA_bench_60': File exists
```

```
HBox(children=(IntProgress(value=0, max=9), HTML(value='')))
```

```
Best ONMI: 0.51006 params: '-v 7'
```

```
In [18]: plot_graph_for_all_results(all_results, "bench_60")
```

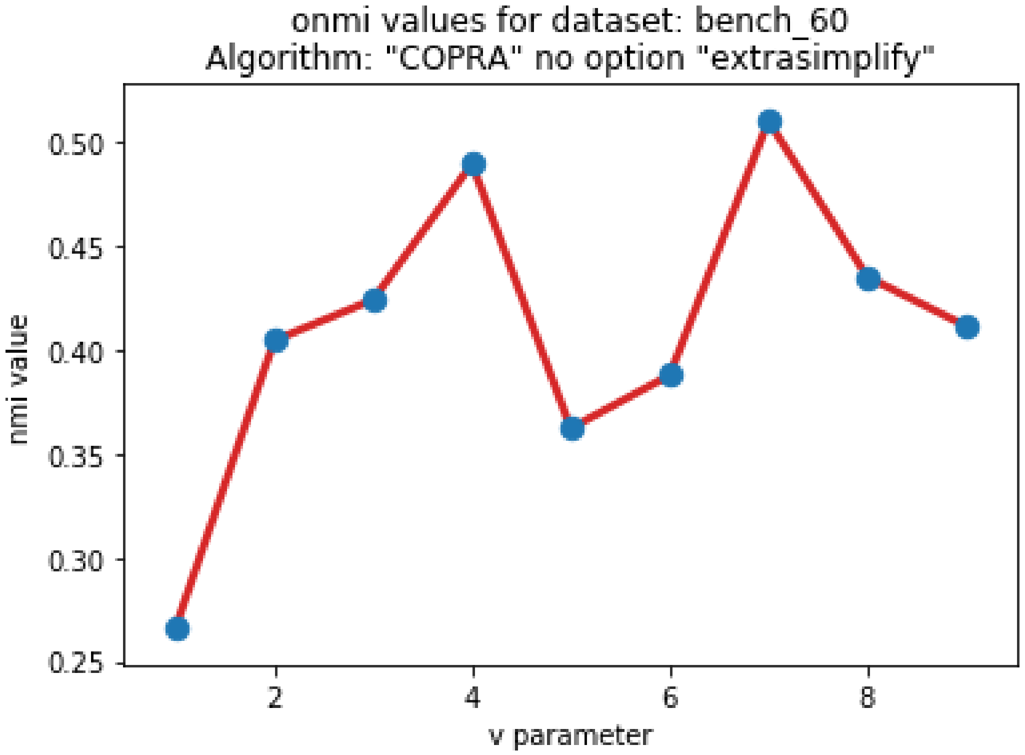

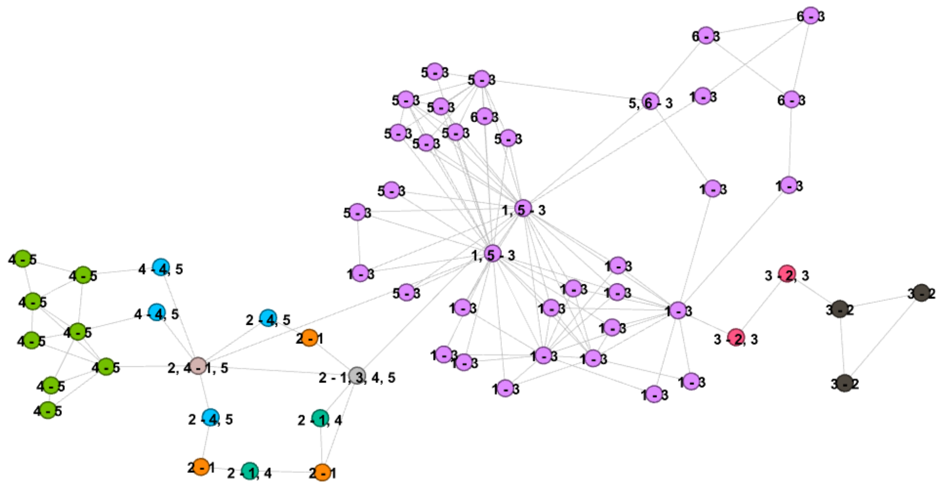

bench_60. Algorithm - COPRA

7.5 bench_60_dense

```
In [15]: inputFile = "../datasets/bench_60_dense/bench_60_dense_network.dat"
         groundTruth = "../datasets/bench_60_dense/bench_60_dense_truth.dat"
         all_results = copra_experiment(inputFile, groundTruth, params, vertexNumerationShift=0)
```

```
Output dir name: ../Results/COPRA_bench_60_dense
Output file name: ../Results/COPRA_bench_60_dense/clusters-bench_60_dense_network.dat
mkdir: cannot create directory '../Results/COPRA_bench_60_dense': File exists
```

```
HBox(children=(IntProgress(value=0, max=9), HTML(value='')))
```

```
Best ONMI: 0.30359 params: '-v 1'
```

```
In [16]: plot_graph_for_all_results(all_results, "bench_60_dense")
```

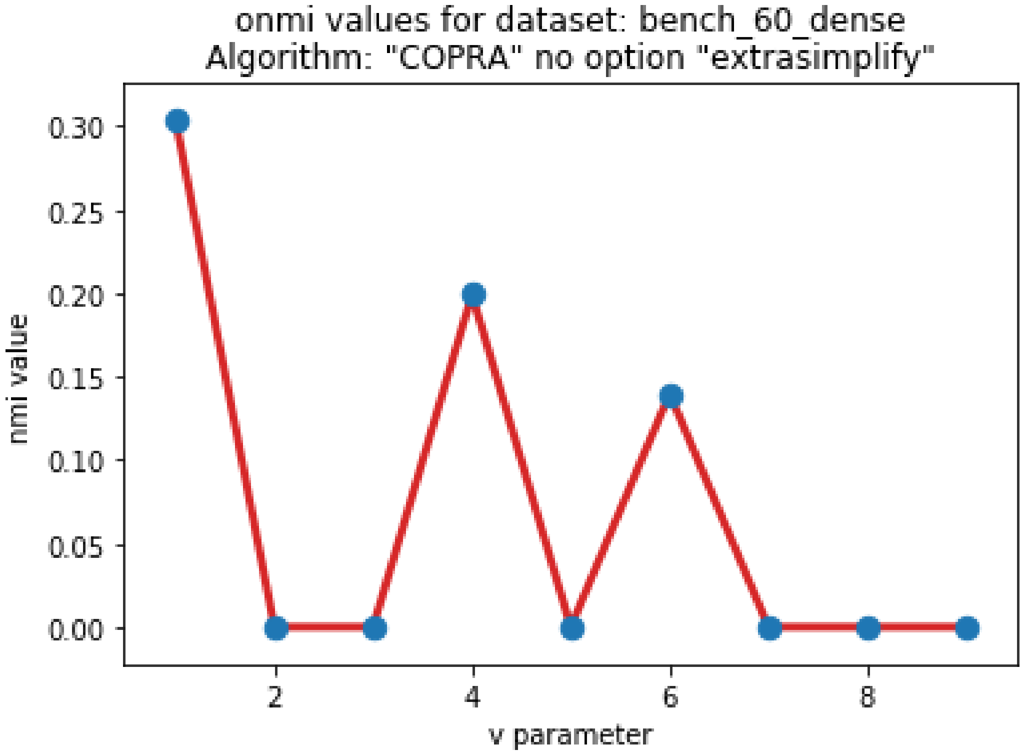

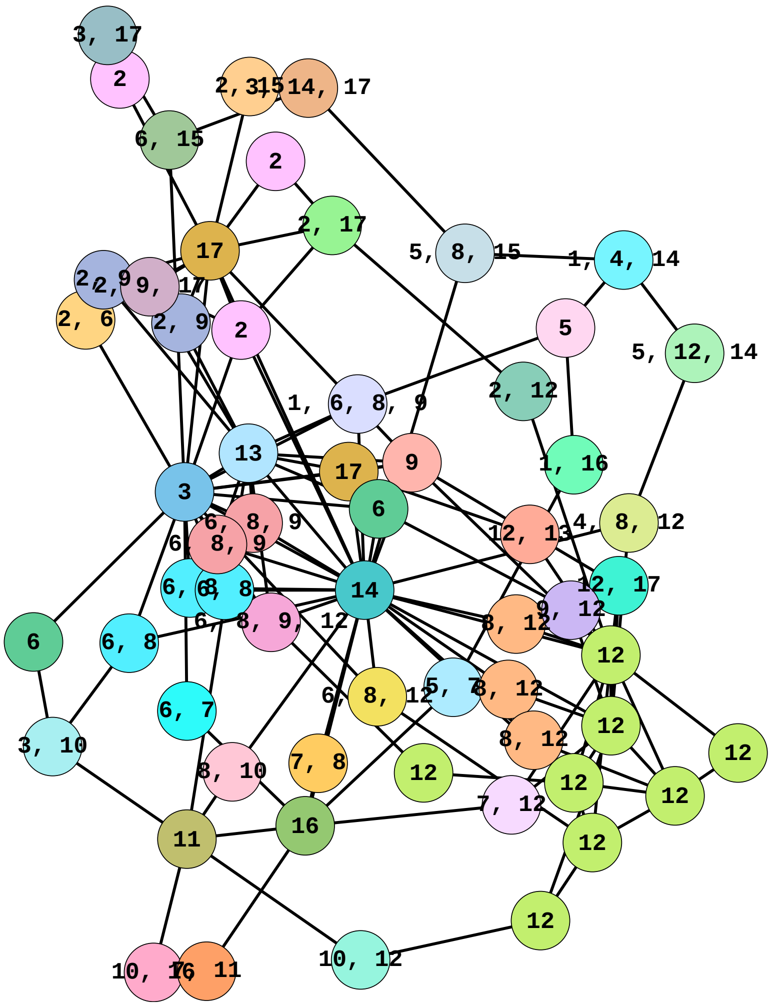

Bench_60_dense. Algorithm - COPRA

8 FARZ benchmarks

8.1 FARZ_n_200_m_5_k_5_beta_1

```
In [77]: inputFile = "../datasets/FARZ_n_200_m_5_k_5_beta_1/network.dat"
         groundTruth = "../datasets/FARZ_n_200_m_5_k_5_beta_1/network.lgt"
         all_results = copra_experiment(inputFile, groundTruth, params, vertexNumerationShift=0)
```

```
Output dir name: ../Results/COPRA_FARZ_n_200_m_5_k_5_beta_1
Output file name: ../Results/COPRA_FARZ_n_200_m_5_k_5_beta_1/clusters-network.dat
mkdir: cannot create directory ‘../Results/COPRA_FARZ_n_200_m_5_k_5_beta_1’: File
exists
```

```
HBox(children=(IntProgress(value=0, max=9), HTML(value='')))
```

```
Best ONMI: 0.964209 params: '-v 6'
Avg ONMI: 0.9062872222222222
```

```
In [78]: plot_graph_for_all_results(all_results, "FARZ_n_200_m_5_k_5_beta_1")
```

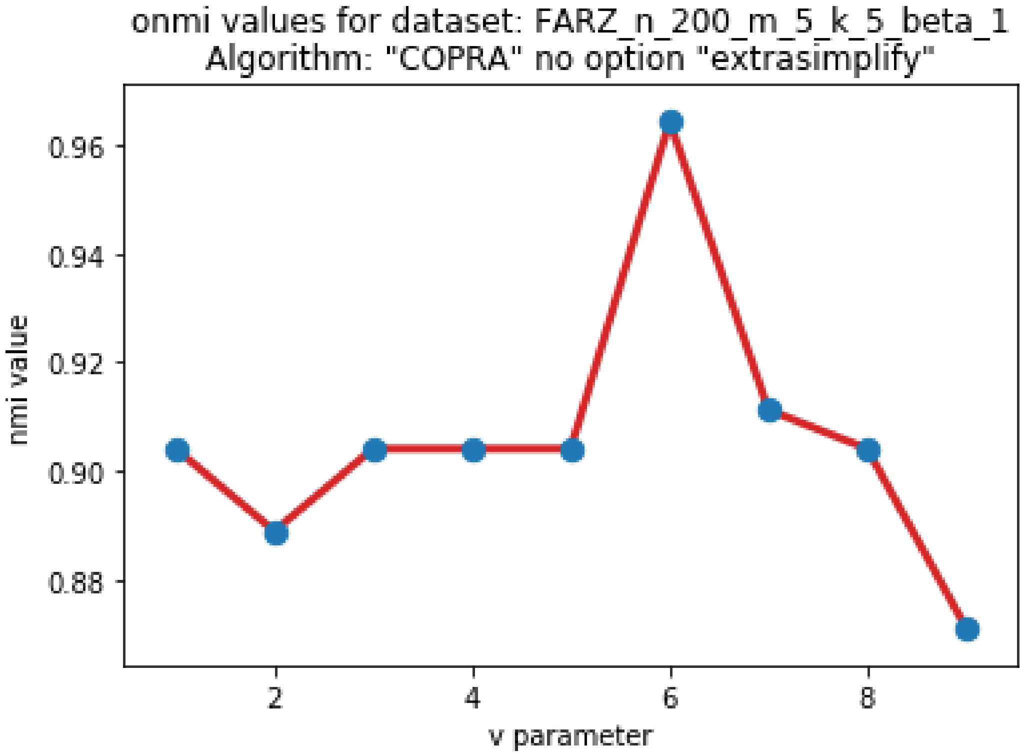


```
In [32]: plot_graph_for_all_results(all_results, "FARZ_n_200_m_5_k_5_beta_1")
```

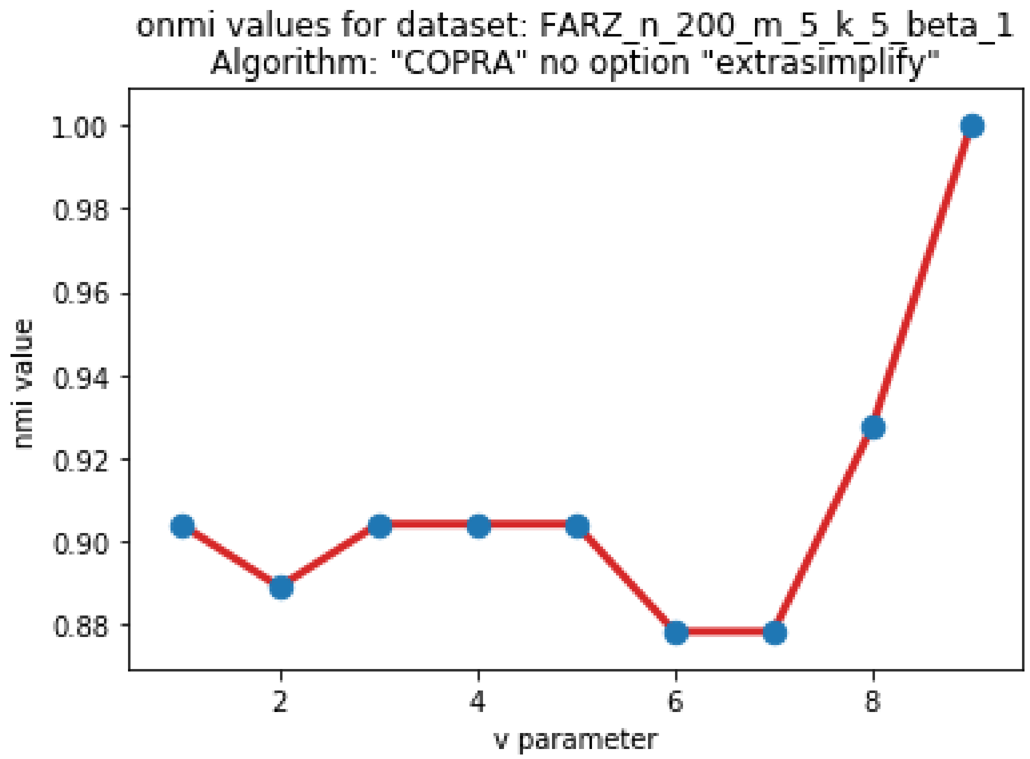

8.2 FARZ_n_200_m_5_k_5_beta_0.95

```
In [79]: inputFile = "../datasets/FARZ_n_200_m_5_k_5_beta_0.95/network.dat"
         groundTruth = "../datasets/FARZ_n_200_m_5_k_5_beta_0.95/network.lgt"
         all_results = copra_experiment(inputFile, groundTruth, params, vertexNumerationShift=0)
```

```
Output dir name: ../Results/COPRA_FARZ_n_200_m_5_k_5_beta_0.95
Output file name: ../Results/COPRA_FARZ_n_200_m_5_k_5_beta_0.95/clusters-network.dat
mkdir: cannot create directory ‘../Results/COPRA_FARZ_n_200_m_5_k_5_beta_0.95’: File
exists
```

```
HBox(children=(IntProgress(value=0, max=9), HTML(value='')))
```

```
Best ONMI: 0.777524 params: '-v 7'
Avg ONMI: 0.7499637777777778
```

```
In [80]: plot_graph_for_all_results(all_results, "FARZ_n_200_m_5_k_5_beta_0.95")
```

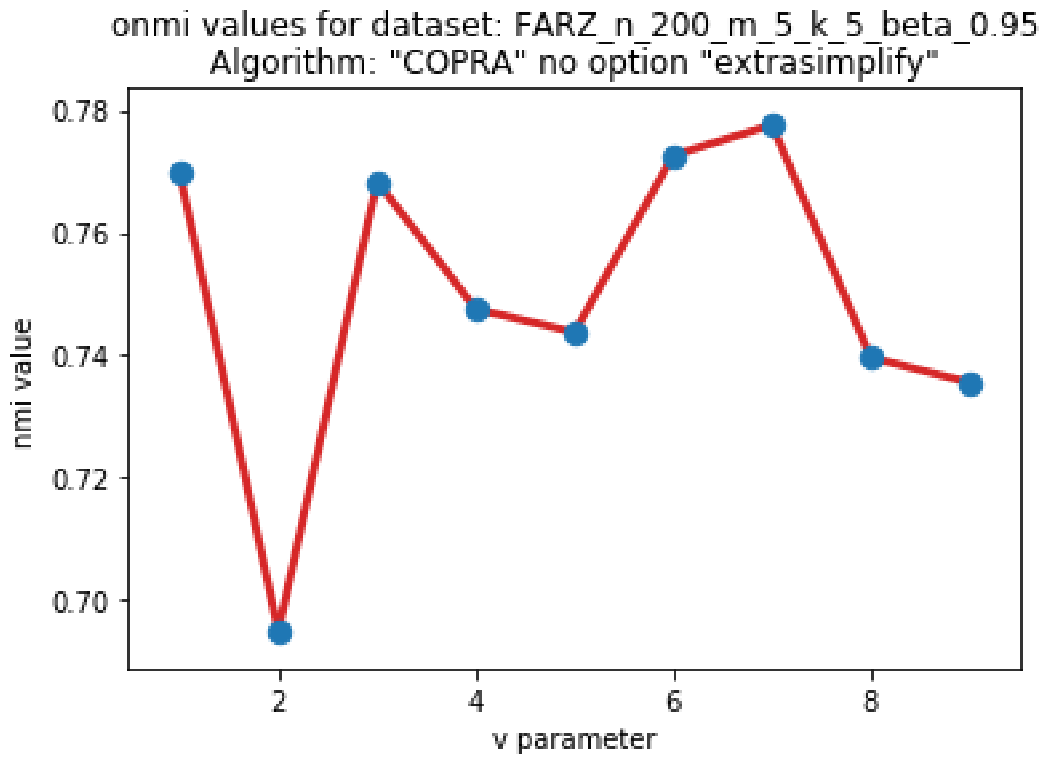


```
In [34]: plot_graph_for_all_results(all_results, "FARZ_n_200_m_5_k_5_beta_0.95")
```

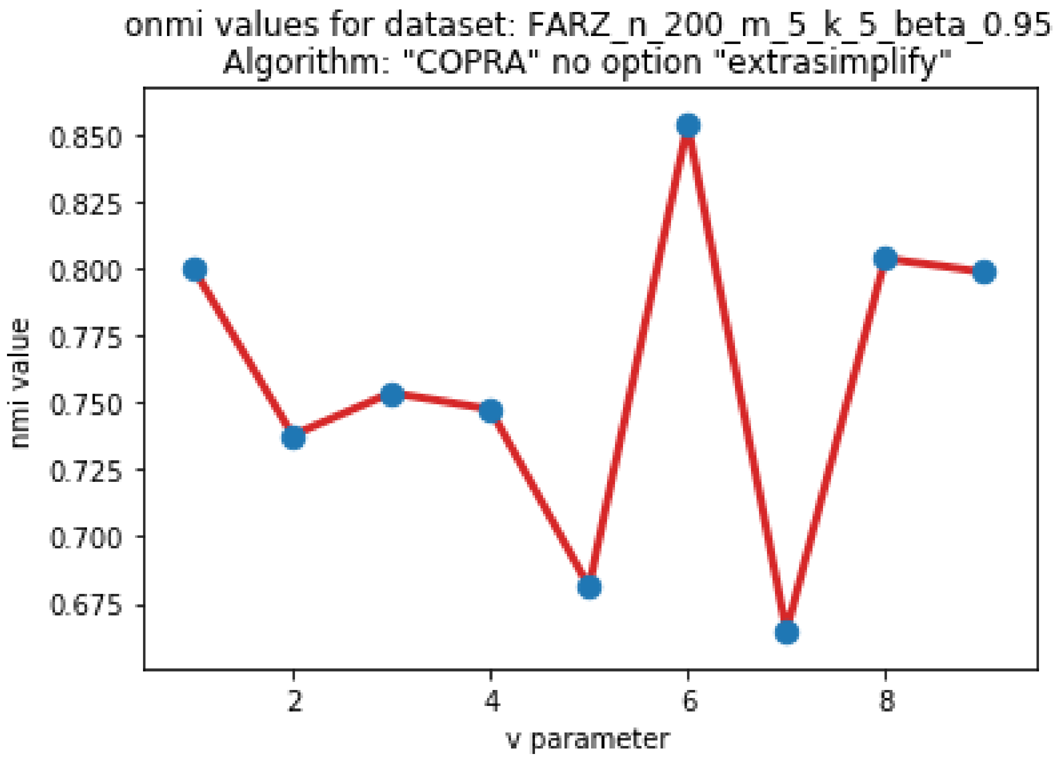

8.3 FARZ_n_200_m_5_k_5_beta_0.9

```
In [81]: inputFile = "../datasets/FARZ_n_200_m_5_k_5_beta_0.9/network.dat"
         groundTruth = "../datasets/FARZ_n_200_m_5_k_5_beta_0.9/network.lgt"
         all_results = copra_experiment(inputFile, groundTruth, params, vertexNumerationShift=0)
```

```
Output dir name: ../Results/COPRA_FARZ_n_200_m_5_k_5_beta_0.9
Output file name: ../Results/COPRA_FARZ_n_200_m_5_k_5_beta_0.9/clusters-network.dat
mkdir: cannot create directory '../Results/COPRA_FARZ_n_200_m_5_k_5_beta_0.9': File
exists
```

```
HBox(children=(IntProgress(value=0, max=9), HTML(value='')))
```

```
Best ONMI: 0.817856 params: '-v 2'
Avg ONMI: 0.6824643333333333
```

```
In [82]: plot_graph_for_all_results(all_results, "FARZ_n_200_m_5_k_5_beta_0.9")
```

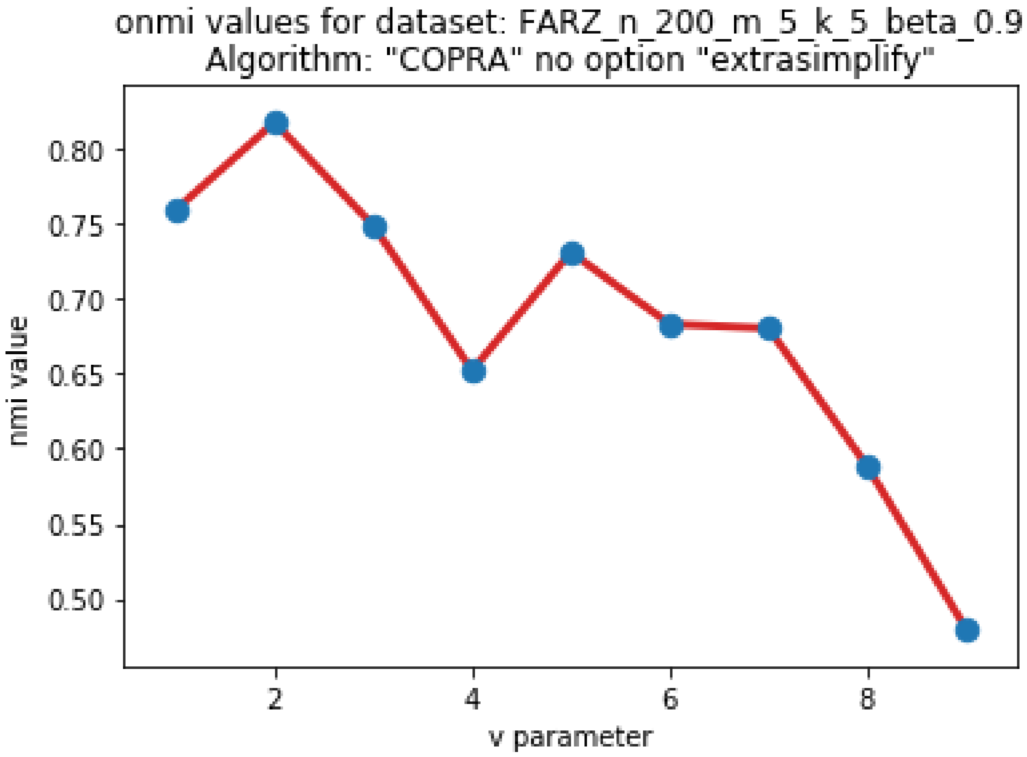


```
In [36]: plot_graph_for_all_results(all_results, "FARZ_n_200_m_5_k_5_beta_0.9")
```

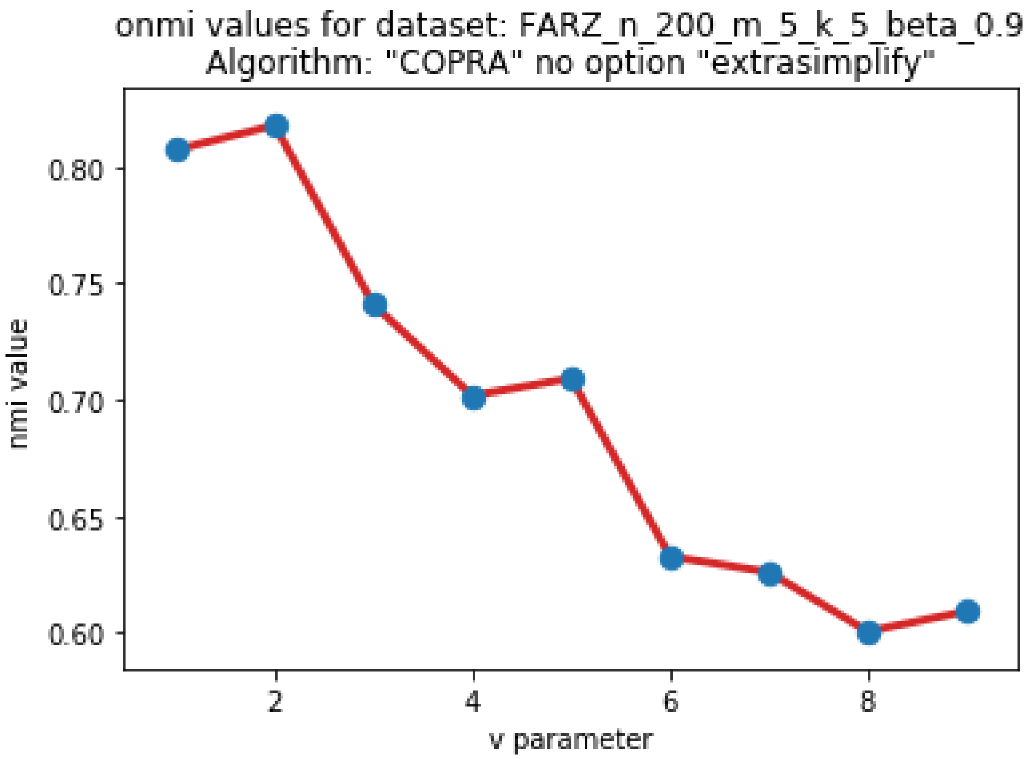

8.4 FARZ_n_200_m_5_k_5_beta_0.85

```
In [83]: inputFile = "../datasets/FARZ_n_200_m_5_k_5_beta_0.85/network.dat"
         groundTruth = "../datasets/FARZ_n_200_m_5_k_5_beta_0.85/network.lgt"
         all_results = copra_experiment(inputFile, groundTruth, params, vertexNumerationShift=0)
```

```
Output dir name: ../Results/COPRA_FARZ_n_200_m_5_k_5_beta_0.85
Output file name: ../Results/COPRA_FARZ_n_200_m_5_k_5_beta_0.85/clusters-network.dat
mkdir: cannot create directory '../Results/COPRA_FARZ_n_200_m_5_k_5_beta_0.85': File
exists
```

```
HBox(children=(IntProgress(value=0, max=9), HTML(value='')))
```

```
Best ONMI: 0.730298 params: '-v 4'
Avg ONMI: 0.6015953333333334
```

```
In [84]: plot_graph_for_all_results(all_results, "FARZ_n_200_m_5_k_5_beta_0.85")
```

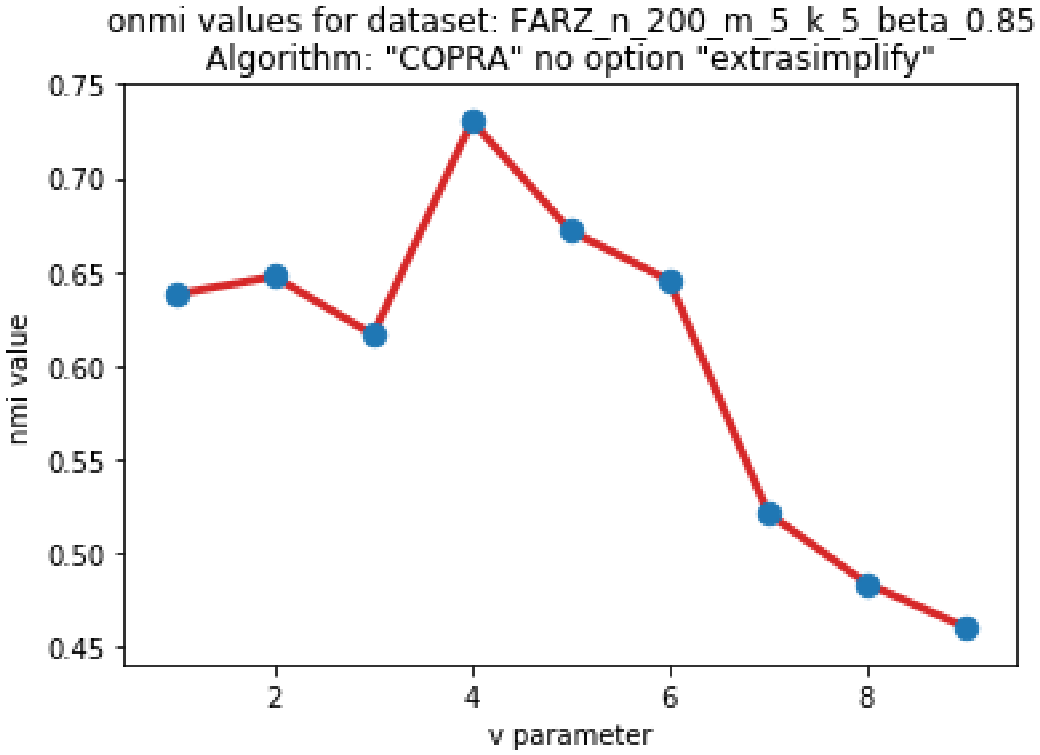


```
In [38]: plot_graph_for_all_results(all_results, "FARZ_n_200_m_5_k_5_beta_0.85")
```

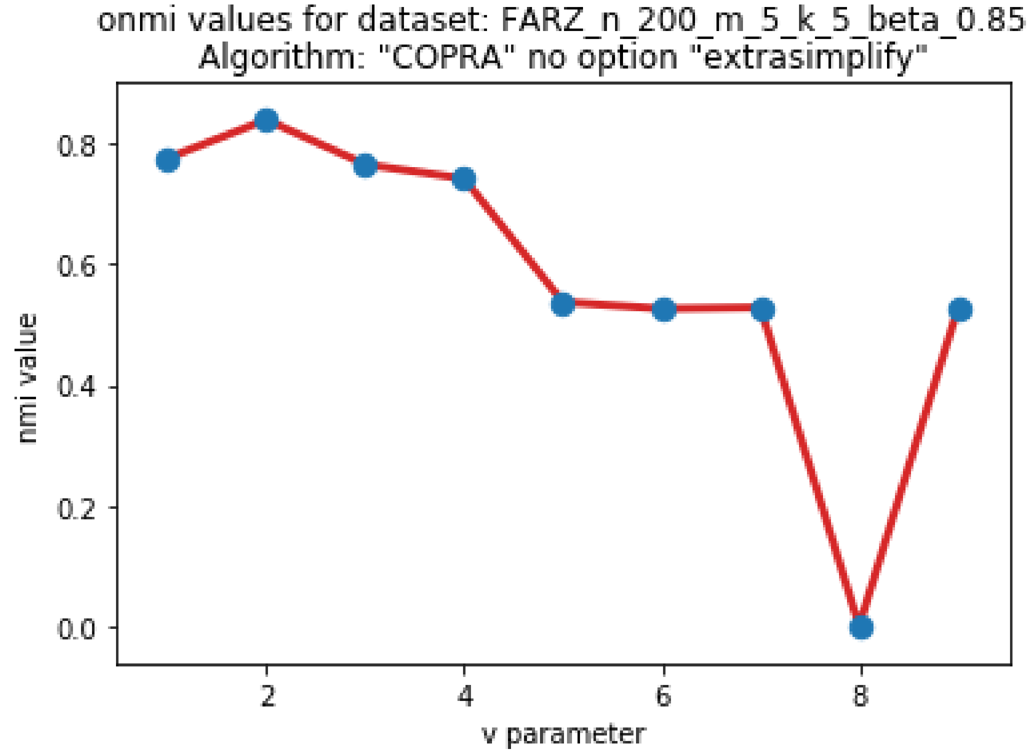

8.5 FARZ_n_200_m_5_k_5_beta_0.8

```
In [85]: inputFile = "../datasets/FARZ_n_200_m_5_k_5_beta_0.8/network.dat"
         groundTruth = "../datasets/FARZ_n_200_m_5_k_5_beta_0.8/network.lgt"
         all_results = copra_experiment(inputFile, groundTruth, params, vertexNumerationShift=0)
```

```
Output dir name: ../Results/COPRA_FARZ_n_200_m_5_k_5_beta_0.8
Output file name: ../Results/COPRA_FARZ_n_200_m_5_k_5_beta_0.8/clusters-network.dat
mkdir: cannot create directory '../Results/COPRA_FARZ_n_200_m_5_k_5_beta_0.8': File
exists
```

```
HBox(children=(IntProgress(value=0, max=9), HTML(value='')))
```

```
Best ONMI: 0.674966 params: '-v 2'
Avg ONMI: 0.48841055555555557
```

```
In [86]: plot_graph_for_all_results(all_results, "FARZ_n_200_m_5_k_5_beta_0.8")
```

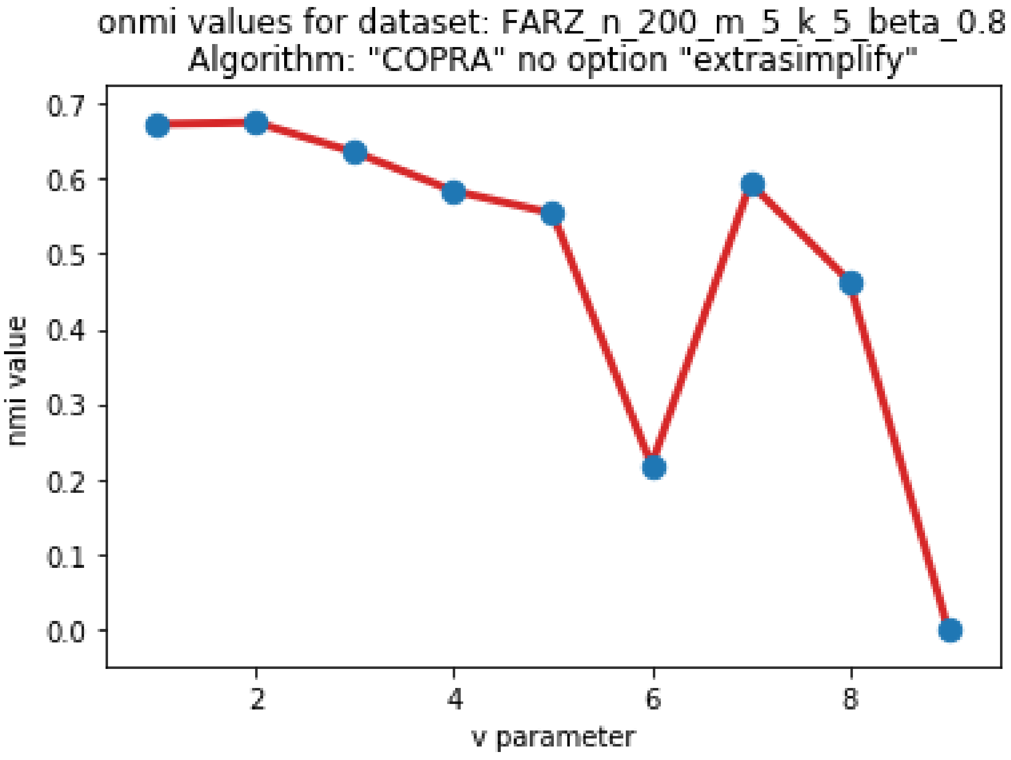


```
In [40]: plot_graph_for_all_results(all_results, "FARZ_n_200_m_5_k_5_beta_0.8")
```

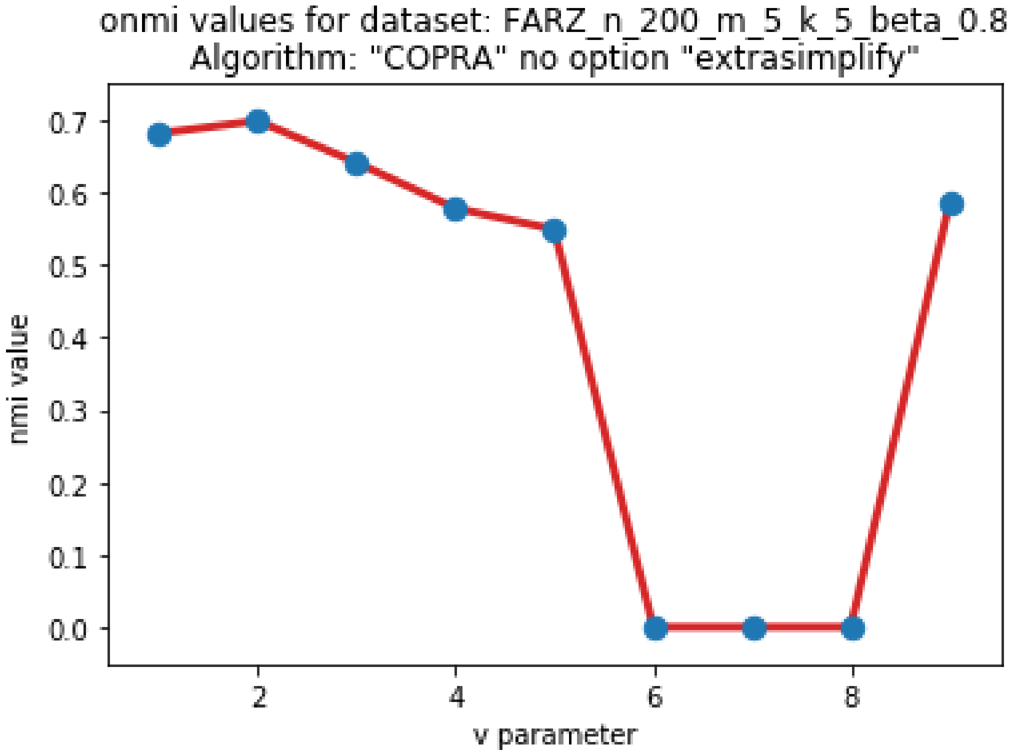


```
In [40]: plot_graph_for_all_results(all_results, "FARZ_n_200_m_5_k_5_beta_0.8")
```

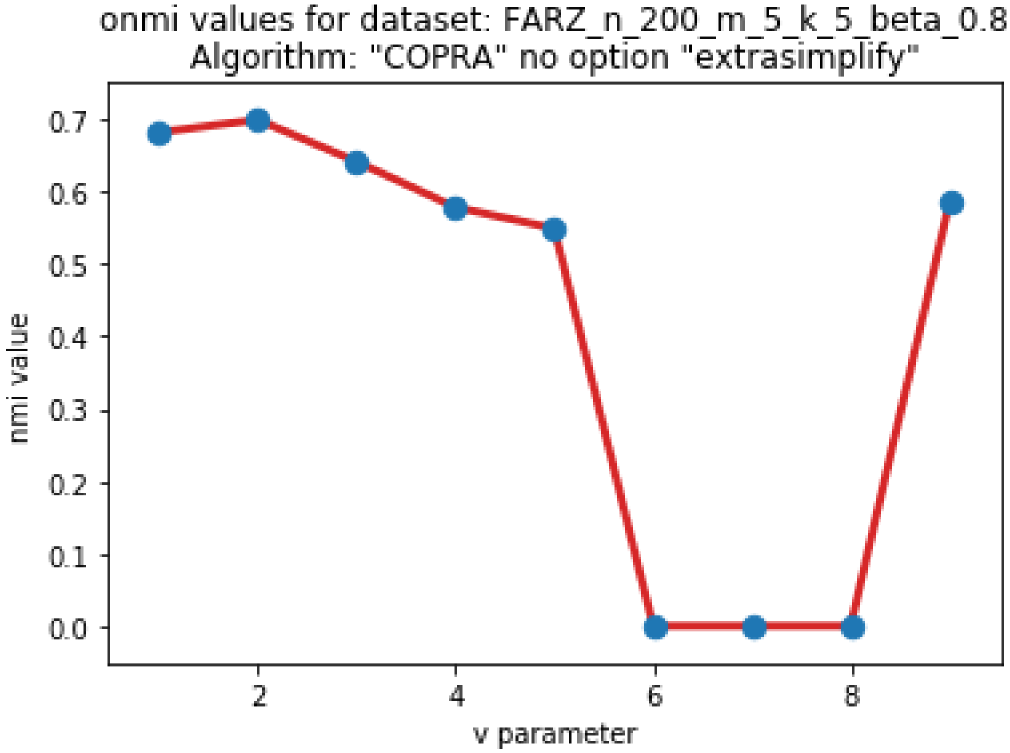

8.6 FARZ_n_200_m_5_k_5_beta_0.75

```
In [87]: inputFile = "../datasets/FARZ_n_200_m_5_k_5_beta_0.75/network.dat"
         groundTruth = "../datasets/FARZ_n_200_m_5_k_5_beta_0.75/network.lgt"
         all_results = copra_experiment(inputFile, groundTruth, params, vertexNumerationShift=0)
```

```
Output dir name: ../Results/COPRA_FARZ_n_200_m_5_k_5_beta_0.75
Output file name: ../Results/COPRA_FARZ_n_200_m_5_k_5_beta_0.75/clusters-network.dat
mkdir: cannot create directory '../Results/COPRA_FARZ_n_200_m_5_k_5_beta_0.75': File
exists
```

```
HBox(children=(IntProgress(value=0, max=9), HTML(value='')))
```

```
Best ONMI: 0.630062 params: '-v 1'
Avg ONMI: 0.2363482222222222
```

```
In [88]: plot_graph_for_all_results(all_results, "FARZ_n_200_m_5_k_5_beta_0.75")
```

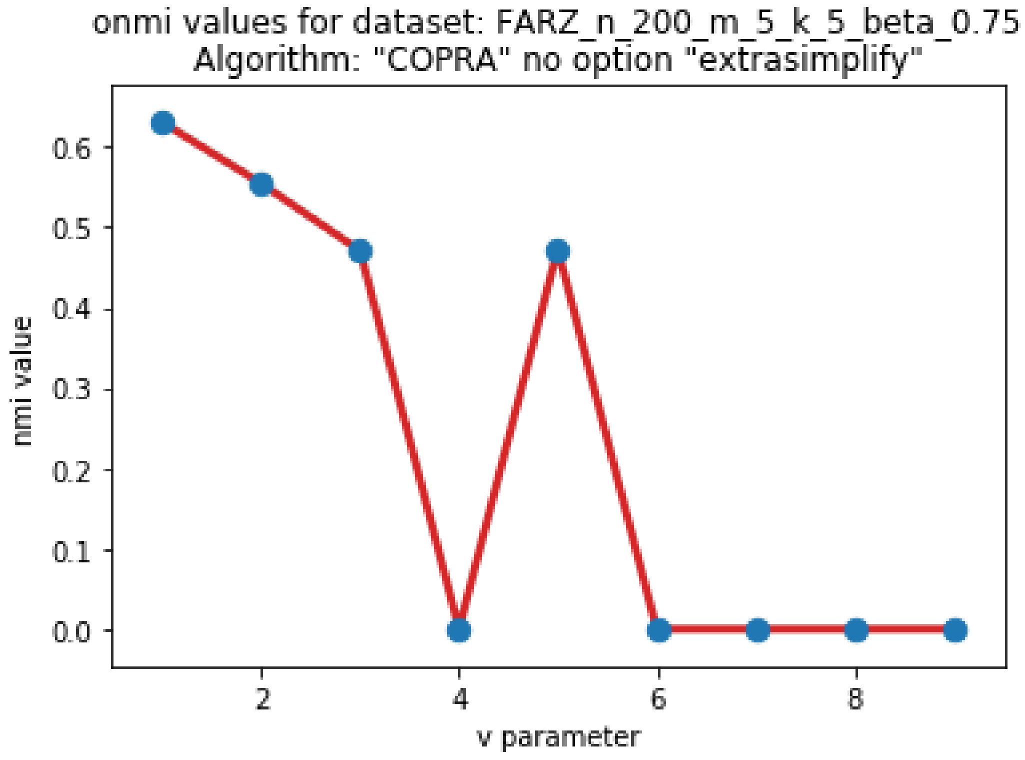


```
In [42]: plot_graph_for_all_results(all_results, "FARZ_n_200_m_5_k_5_beta_0.75")
```

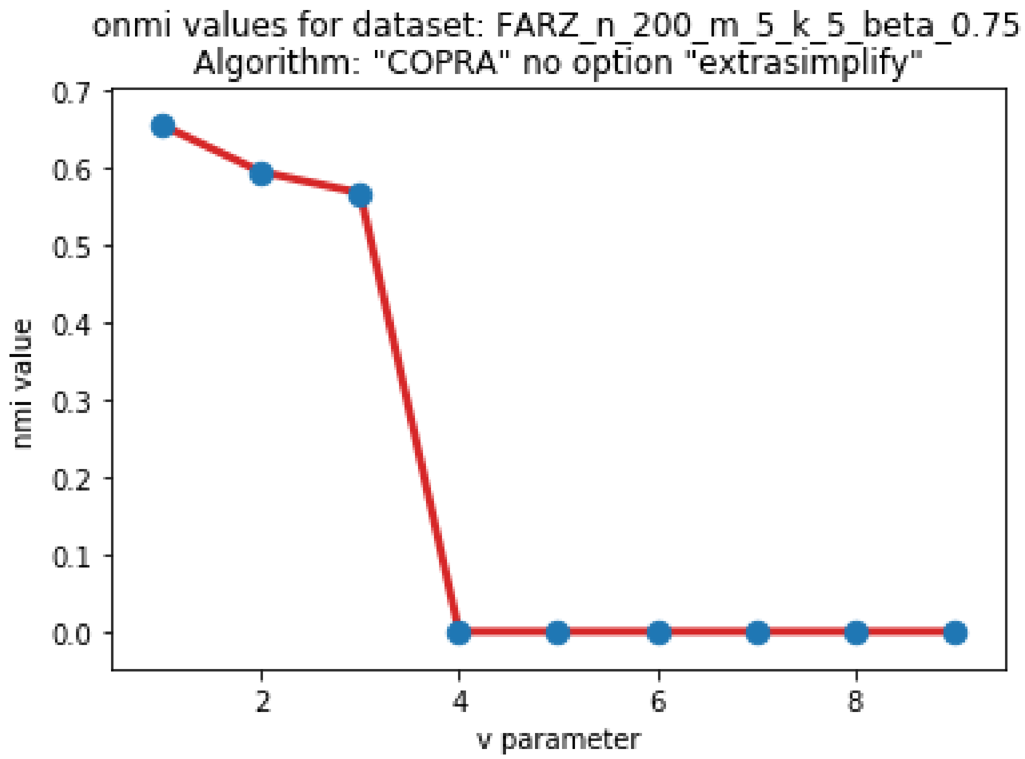

8.7 FARZ_n_200_m_5_k_5_beta_0.7

```
In [89]: inputFile = "../datasets/FARZ_n_200_m_5_k_5_beta_0.7/network.dat"
         groundTruth = "../datasets/FARZ_n_200_m_5_k_5_beta_0.7/network.lgt"
         all_results = copra_experiment(inputFile, groundTruth, params, vertexNumerationShift=0)
```

```
Output dir name: ../Results/COPRA_FARZ_n_200_m_5_k_5_beta_0.7
Output file name: ../Results/COPRA_FARZ_n_200_m_5_k_5_beta_0.7/clusters-network.dat
mkdir: cannot create directory ‘../Results/COPRA_FARZ_n_200_m_5_k_5_beta_0.7’: File
exists
```

```
HBox(children=(IntProgress(value=0, max=9), HTML(value='')))
```

```
Best ONMI: 0.641744 params: '-v 1'
Avg ONMI: 0.07130488888888889
```

```
In [90]: plot_graph_for_all_results(all_results, "FARZ_n_200_m_5_k_5_beta_0.7")
```

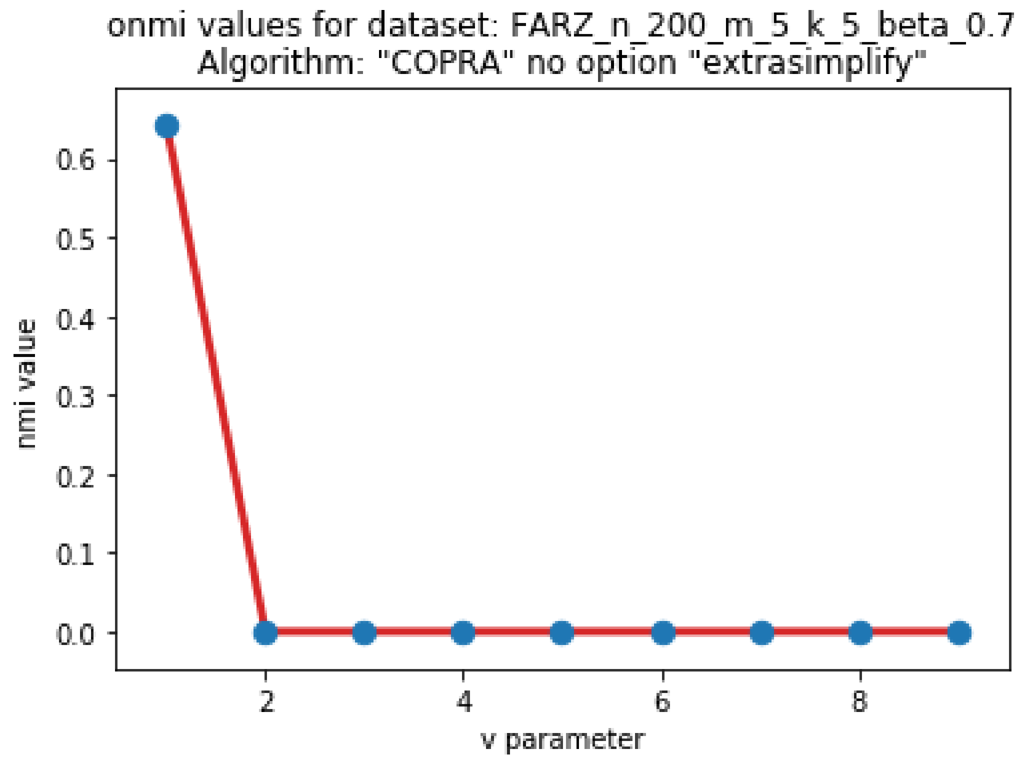


```
In [44]: plot_graph_for_all_results(all_results, "FARZ_n_200_m_5_k_5_beta_0.7")
```

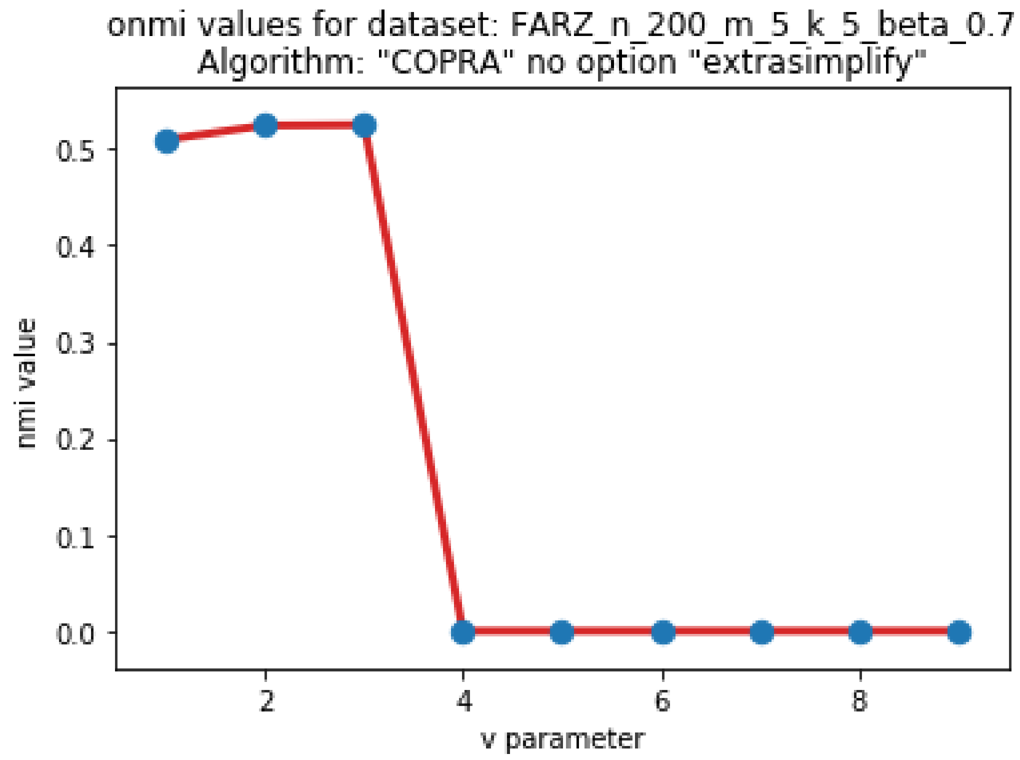

8.8 FARZ_n_200_m_5_k_5_beta_0.65

```
In [91]: inputFile = "../datasets/FARZ_n_200_m_5_k_5_beta_0.65/network.dat"
         groundTruth = "../datasets/FARZ_n_200_m_5_k_5_beta_0.65/network.lgt"
         all_results = copra_experiment(inputFile, groundTruth, params, vertexNumerationShift=0)
```

```
Output dir name: ../Results/COPRA_FARZ_n_200_m_5_k_5_beta_0.65
Output file name: ../Results/COPRA_FARZ_n_200_m_5_k_5_beta_0.65/clusters-network.dat
mkdir: cannot create directory ‘../Results/COPRA_FARZ_n_200_m_5_k_5_beta_0.65’: File
exists
```

```
HBox(children=(IntProgress(value=0, max=9), HTML(value='')))
```

```
Best ONMI: 0.369598 params: '-v 1'
Avg ONMI: 0.10466955555555556
```

```
In [92]: plot_graph_for_all_results(all_results, "FARZ_n_200_m_5_k_5_beta_0.65")
```

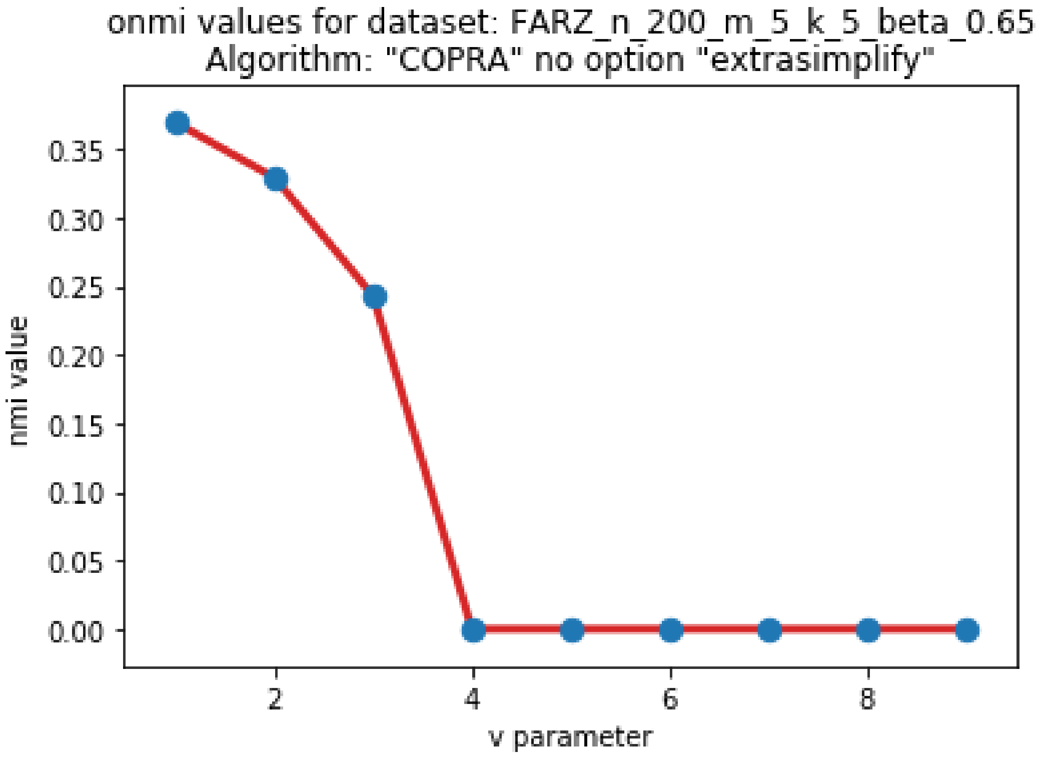


```
In [46]: plot_graph_for_all_results(all_results, "FARZ_n_200_m_5_k_5_beta_0.65")
```

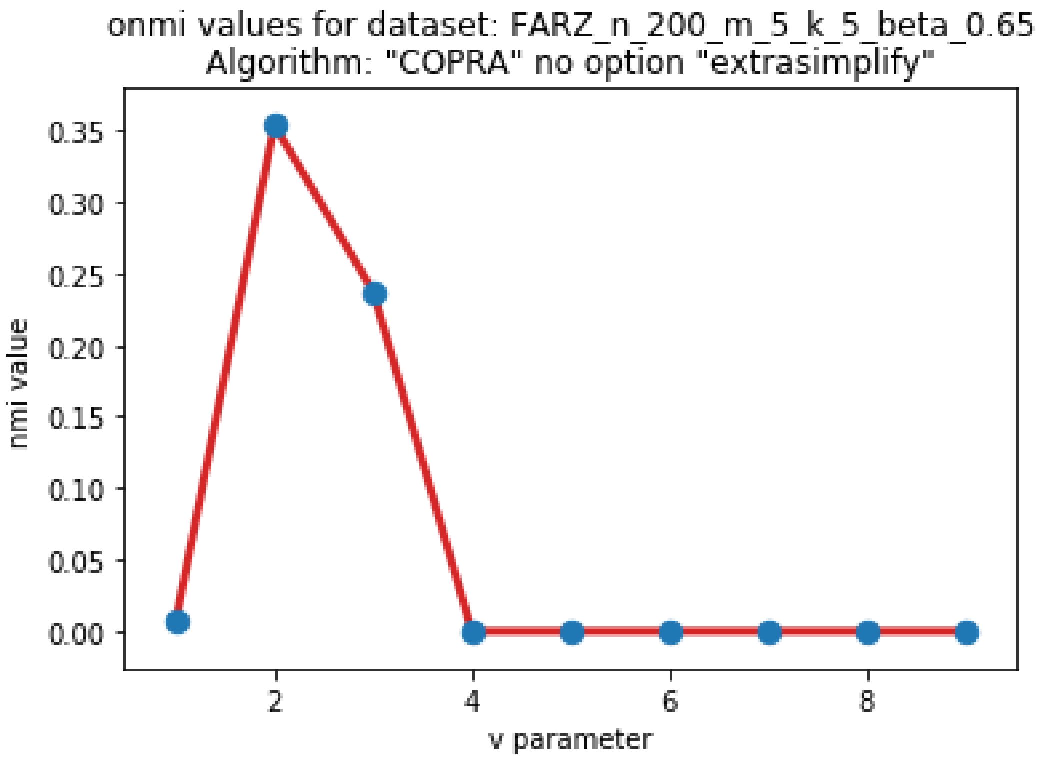

8.9 FARZ_n_200_m_5_k_5_beta_0.6

```
In [93]: inputFile = "../datasets/FARZ_n_200_m_5_k_5_beta_0.6/network.dat"
         groundTruth = "../datasets/FARZ_n_200_m_5_k_5_beta_0.6/network.lgt"
         all_results = copra_experiment(inputFile, groundTruth, params, vertexNumerationShift=0)
```

```
Output dir name: ../Results/COPRA_FARZ_n_200_m_5_k_5_beta_0.6
Output file name: ../Results/COPRA_FARZ_n_200_m_5_k_5_beta_0.6/clusters-network.dat
mkdir: cannot create directory ‘../Results/COPRA_FARZ_n_200_m_5_k_5_beta_0.6’: File
exists
```

```
HBox(children=(IntProgress(value=0, max=9), HTML(value='')))
```

```
Best ONMI: 0.0071265 params: '-v 1'
Avg ONMI: 0.0007918333333333333
```

```
In [94]: plot_graph_for_all_results(all_results, "FARZ_n_200_m_5_k_5_beta_0.6")
```

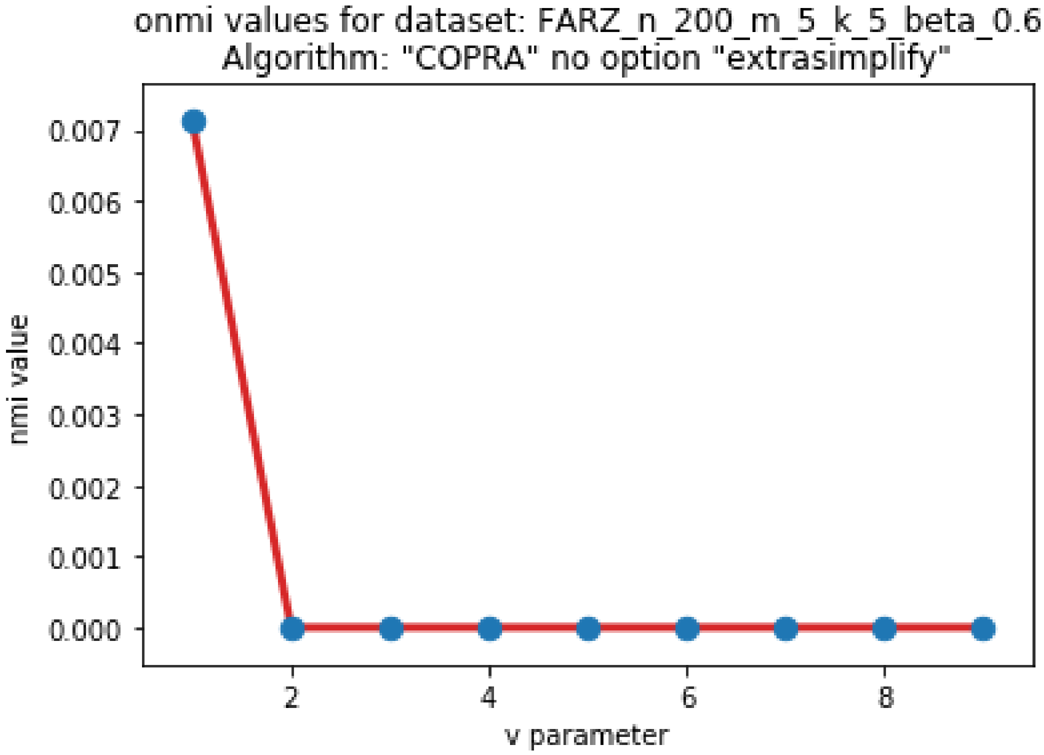

8.10 FARZ_n_200_m_5_k_5_beta_0.55

In [95]:
```
inputFile = "../datasets/FARZ_n_200_m_5_k_5_beta_0.55/network.dat"
groundTruth = "../datasets/FARZ_n_200_m_5_k_5_beta_0.55/network.lgt"
all_results = copra_experiment(inputFile, groundTruth, params, vertexNumerationShift=0)
```

```
Output dir name: ../Results/COPRA_FARZ_n_200_m_5_k_5_beta_0.55
Output file name: ../Results/COPRA_FARZ_n_200_m_5_k_5_beta_0.55/clusters-network.dat
mkdir: cannot create directory '../Results/COPRA_FARZ_n_200_m_5_k_5_beta_0.55': File
exists
```

```
HBox(children=(IntProgress(value=0, max=9), HTML(value='')))
```

```
Best ONMI: 0.274164 params: '-v 2'
Avg ONMI: 0.030462666666666667
```

In [96]:
```
plot_graph_for_all_results(all_results, "FARZ_n_200_m_5_k_5_beta_0.55")
```

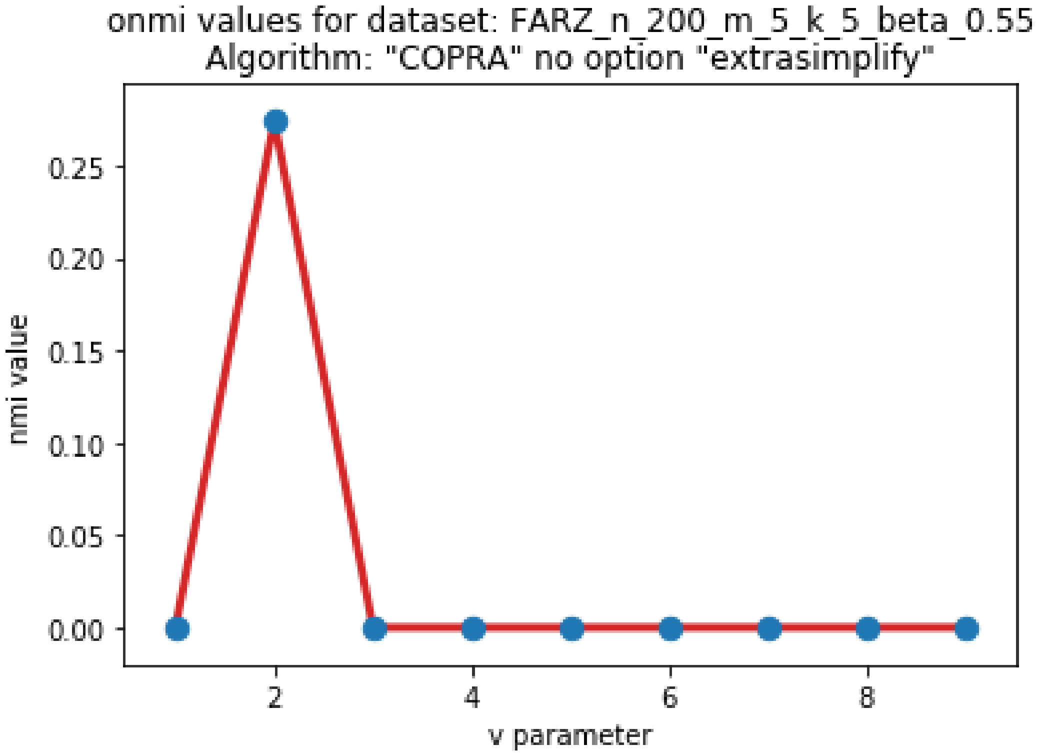


```
In [49]: plot_graph_for_all_results(all_results, "FARZ_n_200_m_5_k_5_beta_0.55")
```

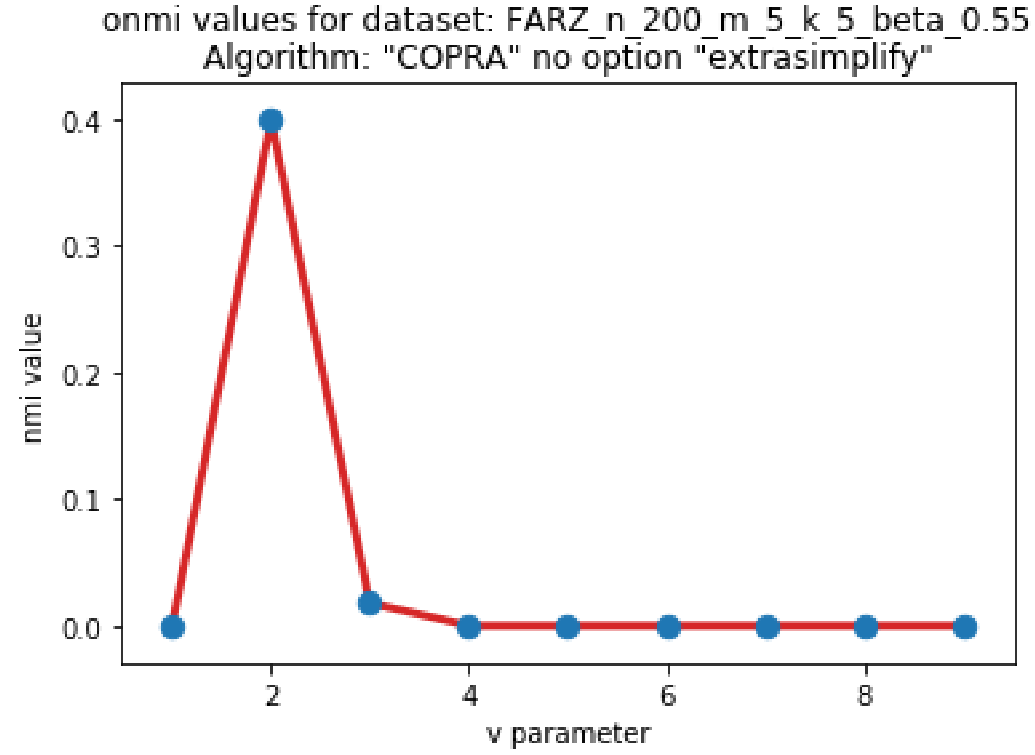

8.11 FARZ_n_200_m_5_k_5_beta_0.5

```
In [97]: inputFile = "../datasets/FARZ_n_200_m_5_k_5_beta_0.5/network.dat"
         groundTruth = "../datasets/FARZ_n_200_m_5_k_5_beta_0.5/network.lgt"
         all_results = copra_experiment(inputFile, groundTruth, params, vertexNumerationShift=0)
```

```
Output dir name: ../Results/COPRA_FARZ_n_200_m_5_k_5_beta_0.5
Output file name: ../Results/COPRA_FARZ_n_200_m_5_k_5_beta_0.5/clusters-network.dat
mkdir: cannot create directory ‘../Results/COPRA_FARZ_n_200_m_5_k_5_beta_0.5’: File
exists
```

```
HBox(children=(IntProgress(value=0, max=9), HTML(value='')))
```

```
Best ONMI: 0.00343204 params: '-v 1'
Avg ONMI: 0.00038133777777777777
```

```
In [98]: plot_graph_for_all_results(all_results, "FARZ_n_200_m_5_k_5_beta_0.5")
```

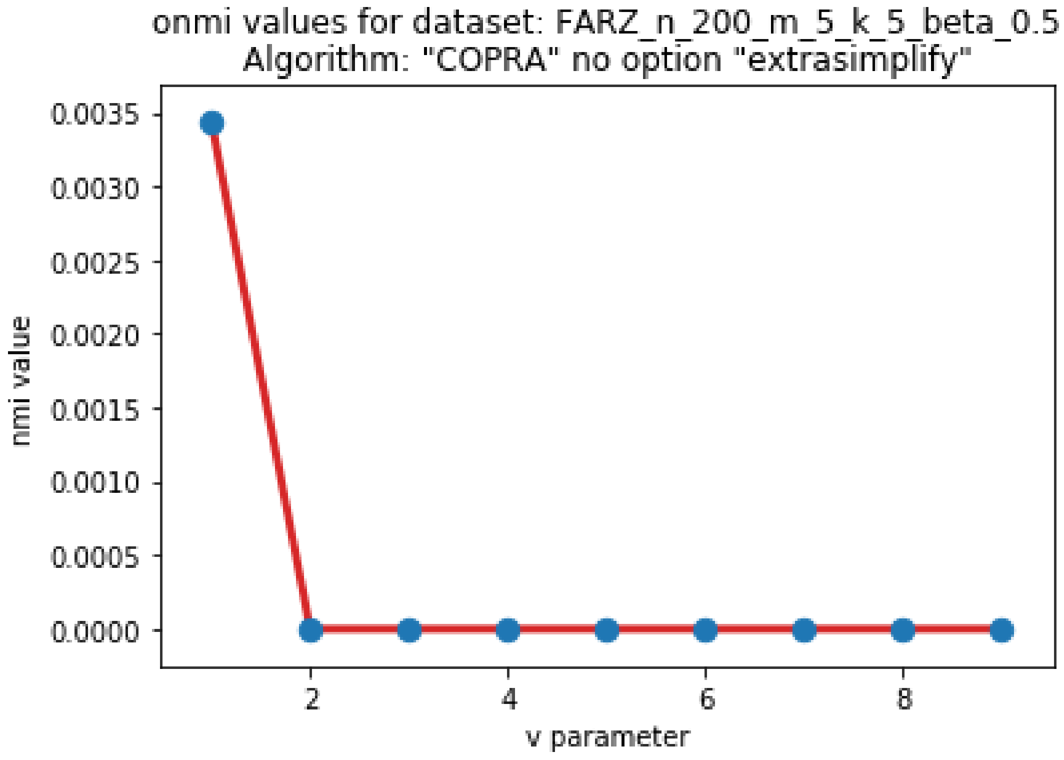


```
In [51]: plot_graph_for_all_results(all_results, "FARZ_n_200_m_5_k_5_beta_0.5")
```

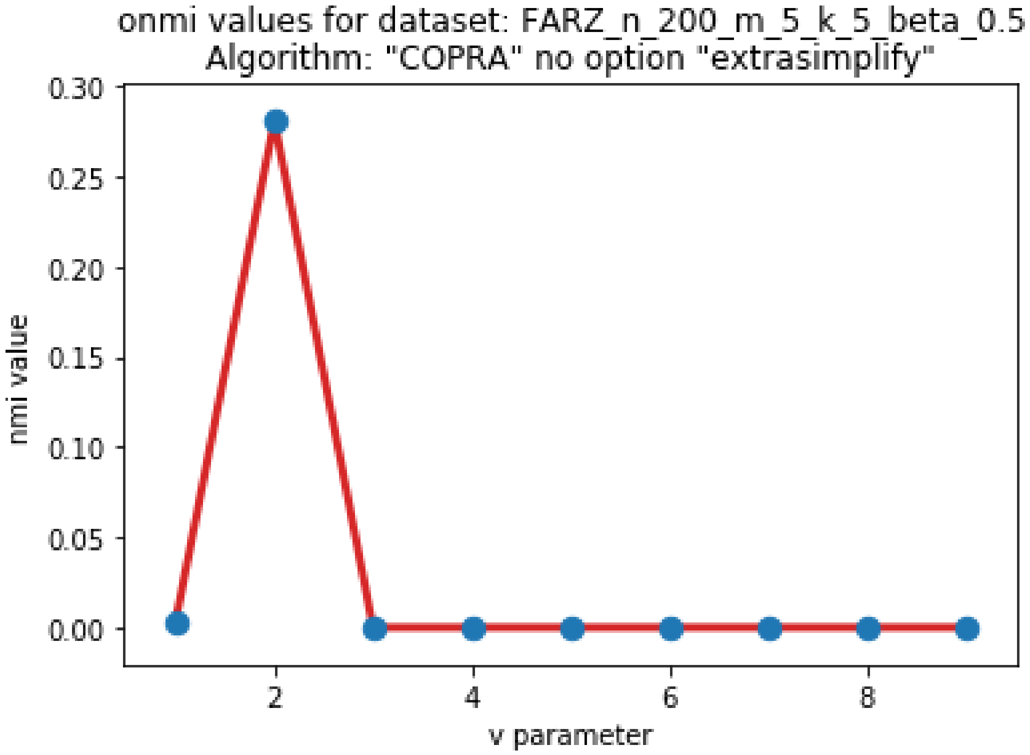

8.12 FARZ_n_1000_m_7_k_20_beta_0.9

```
In [59]: inputFile = "../datasets/FARZ_n_1000_m_7_k_20_beta_0.9/network.dat"
         groundTruth = "../datasets/ FARZ_n_1000_m_7_k_20_beta_0.9/network.lgt"
         all_results = copra_experiment(inputFile, groundTruth, params, vertexNumerationShift=0)
```

```
Output dir name: ../Results/COPRA_FARZ_n_1000_m_7_k_20_beta_0.9
Output file name: ../Results/COPRA_FARZ_n_1000_m_7_k_20_beta_0.9/clusters-network.dat
```

```
HBox(children=(IntProgress(value=0, max=9), HTML(value='')))
```

```
Best ONMI: 0.1 params: '-v 1'
```

```
In [60]: plot_graph_for_all_results(all_results, " FARZ_n_1000_m_7_k_20_beta_0.9")
```

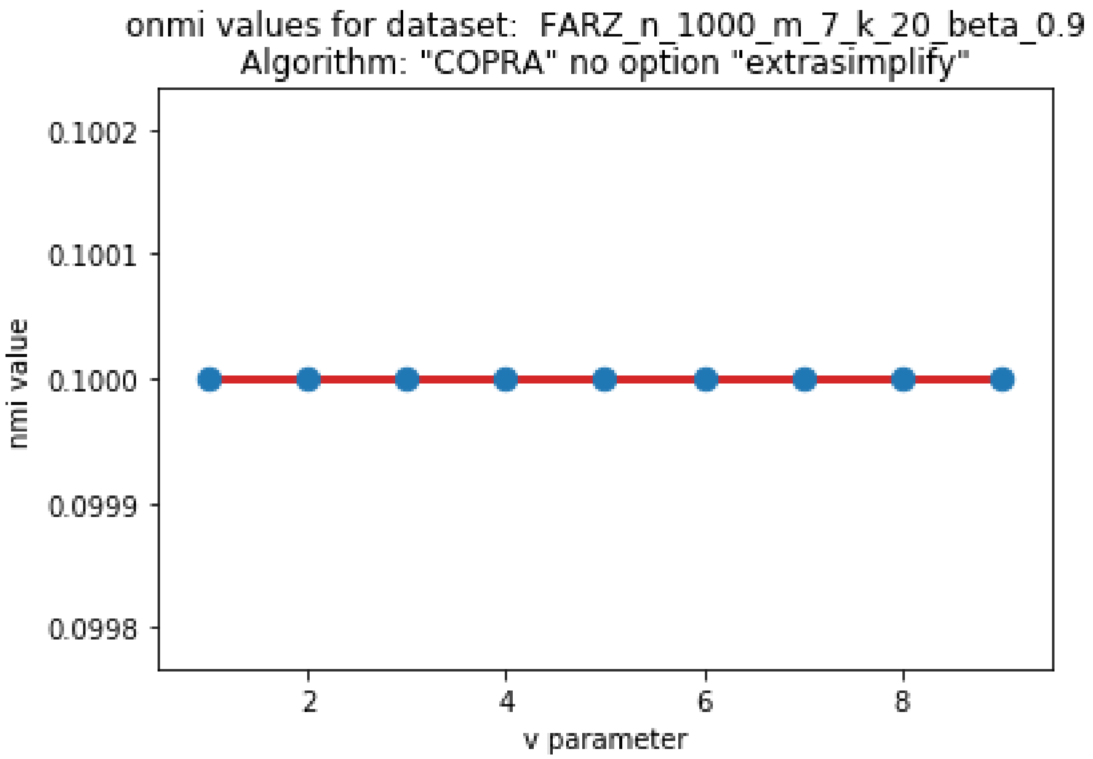

9 CKB and CKB-t

9.1 CKB_n_200

```
In [52]: inputFile = "../datasets/CKB_n_200/dl_edges_tabs.txt"
         groundTruth = "../datasets/CKB_n_200/dl_coms.txt"
         all_results = copra_experiment(inputFile, groundTruth, params, vertexNumerationShift=0)
```

```
Output dir name: ../Results/COPRA_CKB_n_200
Output file name: ../Results/COPRA_CKB_n_200/clusters-dl_edges_tabs.txt
```

```
HBox(children=(IntProgress(value=0, max=9), HTML(value='')))
```

```
Best ONMI: 0.0195658 params: '-v 1'
```

```
In [53]: plot_graph_for_all_results(all_results, "CKB_n_200")
```

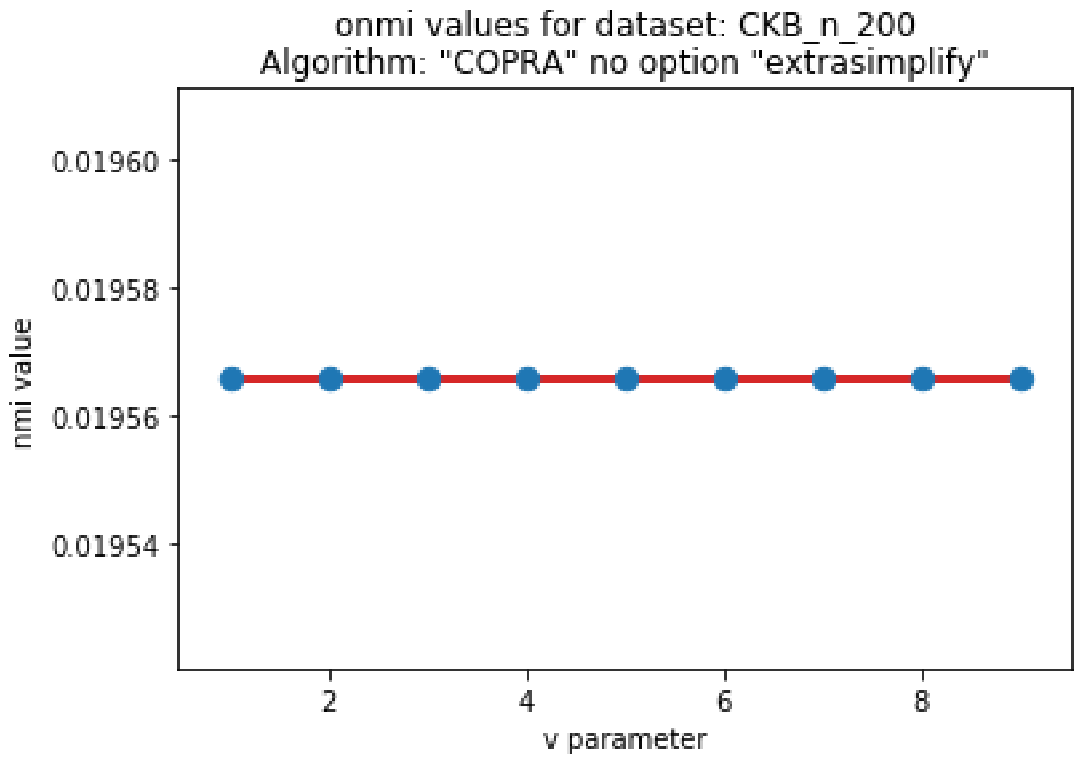

9.2 CKB-t_n_200_alfa_0.1_gamma_0.5

```
In [54]: inputFile = "../datasets/CKB-t_n_200_alfa_0.1_gamma_0.5/dl_edges_tabs.txt"
         groundTruth = "../datasets/CKB-t_n_200_alfa_0.1_gamma_0.5/dl_coms.txt"
         all_results = copra_experiment(inputFile, groundTruth, params, vertexNumerationShift=0)
```

```
Output dir name: ../Results/COPRA_CKB-t_n_200_alfa_0.1_gamma_0.5
Output file name: ../Results/COPRA_CKB-t_n_200_alfa_0.1_gamma_0.5/clusters-
dl_edges_tabs.txt
```

```
HBox(children=(IntProgress(value=0, max=9), HTML(value='')))
```

```
Best ONMI: 0.0299701 params: '-v 7'
```

```
In [55]: plot_graph_for_all_results(all_results, "CKB-t_n_200_alfa_0.1_gamma_0.5")
```

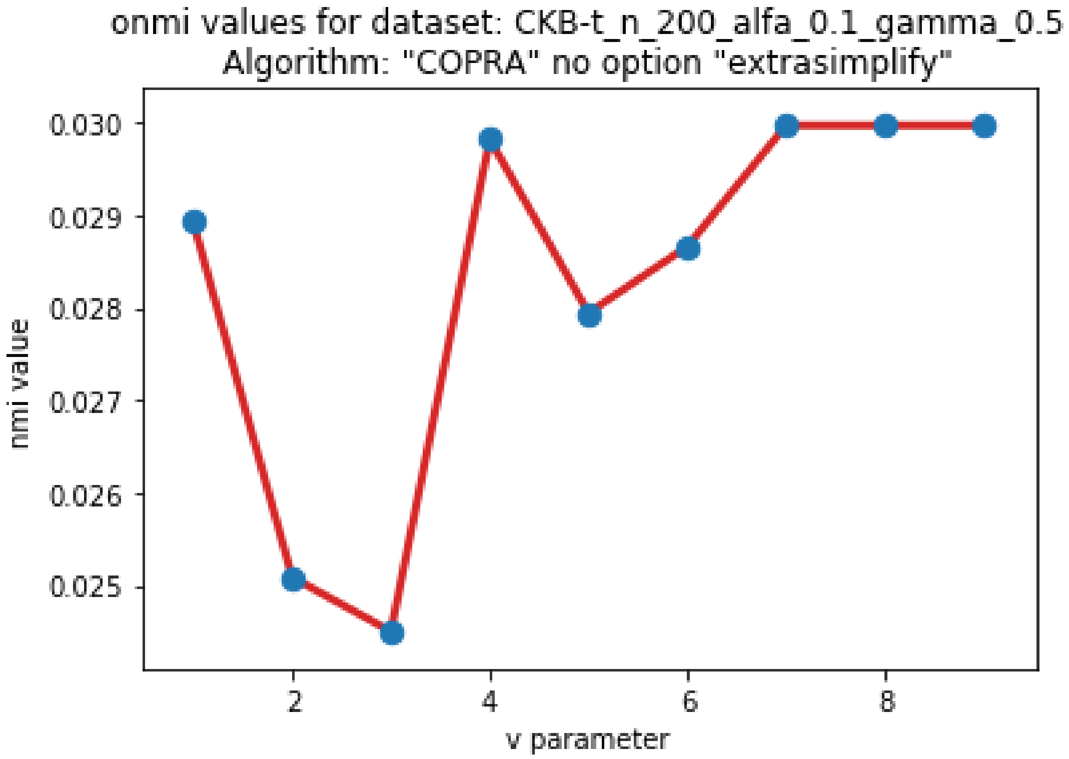

9.3 CKB-t_n_1000_alfa_0.1_gamma_0.5_max_memb_20_max_com_size_200

```
In [100]: inputFile = "../datasets/CKB-
          t_n_1000_alfa_0.1_gamma_0.5_max_memb_20_max_com_size_200/dl_edges_tabs.txt"
          groundTruth = "../datasets/CKB-
          t_n_1000_alfa_0.1_gamma_0.5_max_memb_20_max_com_size_200/dl_coms.txt"
          all_results = copra_experiment(inputFile, groundTruth, params, vertexNumerationShift=0)
```

```
Output dir name: ../Results/COPRA_CKB-
t_n_1000_alfa_0.1_gamma_0.5_max_memb_20_max_com_size_200
Output file name: ../Results/COPRA_CKB-
t_n_1000_alfa_0.1_gamma_0.5_max_memb_20_max_com_size_200/clusters-dl_edges_tabs.txt
mkdir: cannot create directory ‘../Results/COPRA_CKB-
t_n_1000_alfa_0.1_gamma_0.5_max_memb_20_max_com_size_200’: File exists
```

```
HBox(children=(IntProgress(value=0, max=9), HTML(value='')))
```

```
Best ONMI: 0.0112189 params: '-v 2'
Avg ONMI: 0.011039899999999998
```

```
In [102]: plot_graph_for_all_results(all_results, "CKB-
          t_n_1000_alfa_0.1_gamma_0.5_max_memb_20_max_com_size_200 ")
```

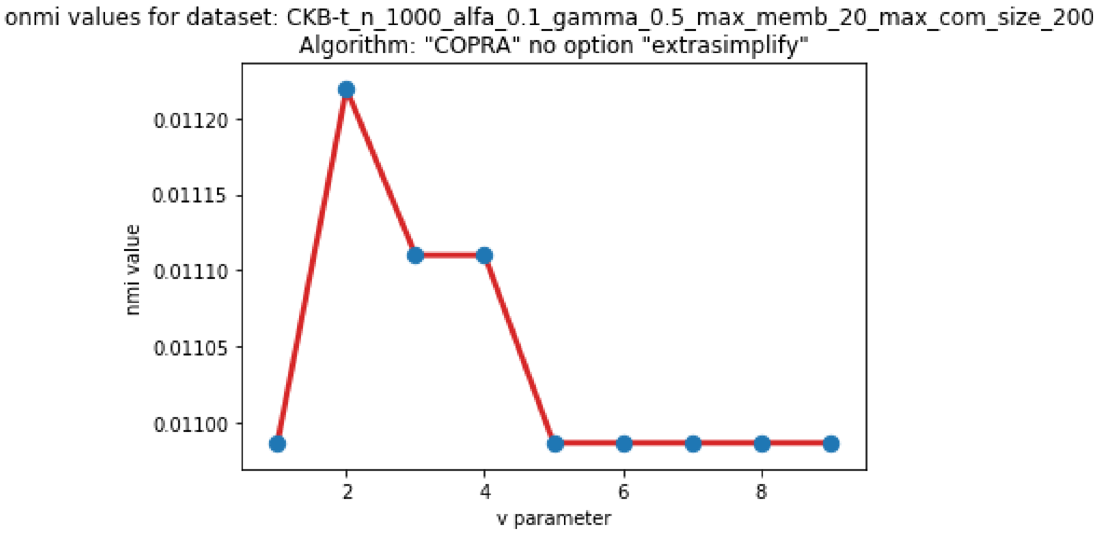

onmi values for dataset: CKB-t_n_1000_alfa_0.1_gamma_0.5_max_memb_20_max_com_size_200
Algorithm: "COPRA" no option "extrasimplify"
0.01120
0.01115
0.01110
0.01105
0.01100
nmi value
2
4
6
8
v parameter


```
In [ ]:
```
