# Supplementary material for: Overlapping community detection in networks based on link partitioning and partitioning around medoids
Source: PLoS One. 2021 Aug 25;16(8):e0255717. doi: 10.1371/journal.pone.0255717 (PMC8386890; doi:10.1371/journal.pone.0255717)
Supplement: S8 Appendix — The set of flags used for the benchmark networks generator by Andrea Lancichinetti and Santo Fortunato. (PDF) [file pone.0255717.s008.pdf]

Flags for Network Benchmarks Generator by Andrea Lancichinetti and Santo Fortunato

	bench_30	bench_40	bench_50	bench_60	bench_60_dense
-N	30	40	50	60	60
-k	4	6	6	5	5
-maxk	12	20	20	30	30
-mu	0.1	0.2	0.1	0.05	0.25
-t1	2	2	2	2	2
-t2	1	1	1	1	1
-minc	5	5	5	5	5
-maxc	20	25	25	30	30
-on	2	5	4	4	5
-om	2	2	2	2	2
timeseed	21111986	21111984	21111984	21111984	21111984
